# Chiral Heptagon‐Embedded Double [6]Helicenes via Scholl Reaction

**DOI:** 10.1002/anie.8159257

**Published:** 2026-03-13

**Authors:** Dan Wang, Philipp Penert, Lars Schneider, Moritz P. Schuldt, Frank Rominger, Felix Deschler, Michael Mastalerz

**Affiliations:** ^1^ Organisch‐Chemisches Institut Ruprecht‐Karls‐Universität Heidelberg Heidelberg Germany; ^2^ Physikalisch‐Chemisches Institut Ruprecht‐Karls‐Universität Heidelberg Heidelberg Germany

**Keywords:** chiral PAH, heptagons, multiple helicenes, Scholl reaction

## Abstract

A series of heptagon‐embedded multiple helicenes was synthesized in which the heptagon subunit was fused to the π‐framework through Knoevenagel condensation reactions. By controlling the conditions of the cyclodehydrogenation (Scholl) reactions, different fused chiral and twisted PAHs were accessible. The optical and electronic properties of all products were investigated by UV–vis, fluorescence spectroscopy, and cyclic voltammetry. One member of the series displays an unusual anti‐Kasha‐like fluorescence emission. In addition, all the enantiopure [6]helicenes were separated by chiral HPLC and characterized by circular dichroism spectroscopy.

## Graphical abstract

One bond makes the difference–two chiral and twisted polycyclic aromatic hydrocarbons (PAHs) show different emission behavior. The more flexible one (top) shows a double emission with one having a large anti‐Stokes shift. The two peaks are assigned to different conformers of one and the same compound.

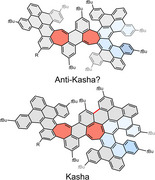

## 1 Introduction

Nonplanar polycyclic aromatic hydrocarbons (PAHs) are, in contrast to structurally comparable planar PAHs, more soluble due to less efficient π‐stacking [1, 2, 3, 4, 5]. The improved solubility allows better processability, photophysical studies, or follow‐up chemistry in solution. Furthermore, nonplanar PAHs can be chiral and thus have exceptional chiroptical properties, if these are enantio‐ or diastereopure [6, 7, 8]. To introduce nonplanarity, several strategies have been used. One is to embed rings with a smaller (e.g., tetragons, pentagons) or larger (e.g., heptagons, octagons) number of ring members than six (hexagons) [2, 9, 10, 11]. PAHs containing heptagons have been synthesized more often than those with octagons [2, 12, 13, 14, 15, 16, 17, 18, 19, 20, 21, 22, 23, 24, 25, 26, 27, 28, 29, 30, 31, 32, 33, 34, 35, 36, 37]. In most cases, the PAHs have been generated by Scholl‐type oxidations, and compounds with up to four [38, 39, 40, 41], five [10, 37] or even six heptagons [40] have been realized.

Besides Scholl‐type oxidations, other strategies to make PAHs with seven‐membered rings have recently been introduced. For instance, the Würthner group used the approach to doubly cross‐couple a cyclic borinic acid with ortho‐dibromides to generate the seven‐membered ring PAHs [35, 42, 43, 44]. Our group used the approach to make larger PAHs by a sequence of Suzuki–Miyaura cross‐coupling, followed by condensation reactions of aldehyde or ketone groups with (doubly activated) acidic benzylic positions [21, 45, 46, 47, 48, 49, 50, 51]. Depending on the substrate, PAHs with six [45, 46, 47, 48, 49], seven [50], or eight‐membered [51] rings have been generated by this method.

Another subclass of nonplanar PAHs is the one of the helicenes, which exhibit inherent helical chirality [52, 53, 54, 55, 56, 57, 58, 59, 60, 61, 62, 63, 64, 65, 66, 67, 68, 69]. Helicenes are known to have unique chiroptical properties, and helicenes, extended helicenes, and similar compounds have been synthesized longer and longer to fine‐tune these properties [70, 71, 72, 73, 74, 75, 76]. Incorporation of heptagons as defects into the helical system is expected to enhance nonlinear optics and chiroptical properties [6, 77, 78, 79]. For instance, in 2020 Narita's group reported the synthesis of negatively curved nanographene with two heptagons and one [5]helicene substructure by an unexpected aryl rearrangement [52]. Most recently, Yang's group successfully synthesized a double helicene bearing two NBN‐doped heptagons, which shows excellent photoluminescence quantum yields [80]. However, despite these few intriguing examples, embedding heptagons into π‐extended multiple helicenes has still been rarely exploited [81].

Here, we report the synthesis of a series of multiple helicenes containing a highly twisted backbone with two heptagons. One member of the series shows unusual and strong anti‐Kasha‐like photophysical properties [81, 82, 83], which have been further investigated by experimental and computational studies.

## 2 Results and Discussion

### 2.1 Synthesis and Characterization

**SCHEME 1:**
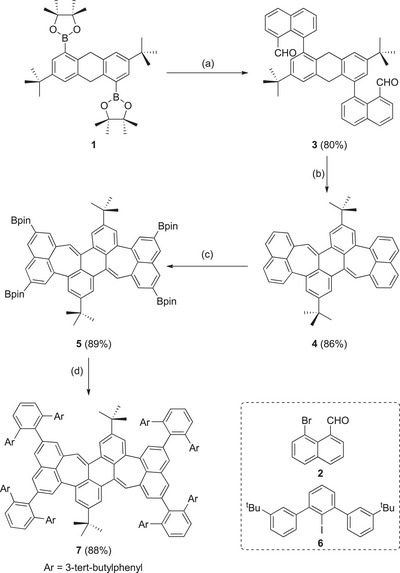
Synthesis of precursor **7**. (a) **2** (3.5 equiv.), Pd(PPh_3_)_4_ (10 mol%), K_2_CO_3_ (1 M), DME, 80°C, 16 h; (b) KO*t*Bu (6.0 equiv.), THF, 80°C, 72 h; (c) B_2_pin_2_ (7.0 equiv.), [Ir(COD)OMe]_2_ (0.1 equiv.), TMPhen (0.2 equiv.), THF, 80°C, 72 h; (d) **6** (6.0 equiv.), Pd(PPh_3_)_4_ (10 mol%), Cs_2_CO_3_ (10 equiv.), toluene, 120°C, 16 h.

The synthesis of the large PAHs is based on diboronic ester **1** (Scheme 1) [36, 46, 47, 84, 85, 86]. The first step is a twofold palladium‐catalyzed cross‐coupling with naphtylbromo aldehyde **2** to give bisaldehyde **3** in 88% yield. By subsequent base‐mediated cyclization [50] with KOtBu, two heptagons are formed, giving the twisted PAH **4** in a yield of 86% (see also X‐ray structure). PAH **4** was regioselectively four‐fold borylated by iridium‐catalysis to give PAH **5** in 89% yield. Next step was the fourfold Suzuki–Miyaura cross‐coupling with iodo terphenyl **6**, resulting in sterically crowded PAH **7** (88% yield) [38]. All compounds have been fully characterized (for details, see Supporting Information), and for PAHs **4** and **5,** we were able to grow single crystals for X‐ray diffraction analysis (see also discussion below).

**SCHEME 2:**
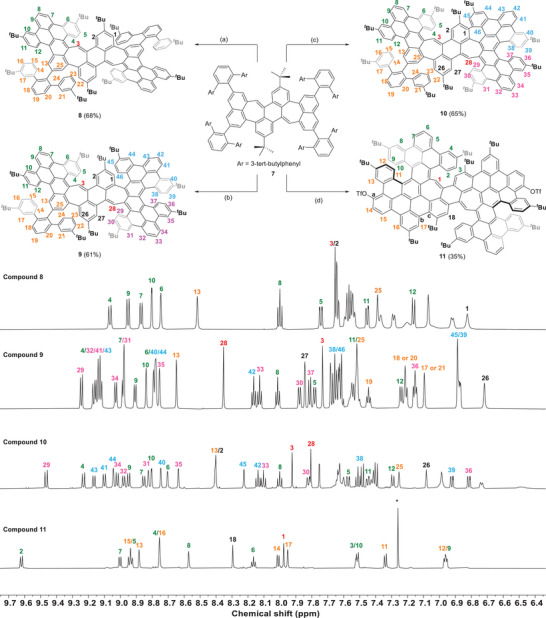
Top: Cyclodehydrogenation reactions of precursor **7**. Reagents and conditions: (a) DDQ (16 equiv.) CH_3_SO_3_H (280 equiv.), Ar, DCM, rt, 90 min; (b) DDQ (16 equiv.), TfOH (1:200), DCM, 0°C, 5 min; (c) FeCl_3_ (80 equiv.), MeNO_2_, Ar, DCM, 0–40°C, 60 min; (d) DDQ (16 equiv.), TfOH (1:60), DCM, 0°C–rt, 60 min. Bottom: Partial ^1^H NMR spectra of **8** (THF‐*d_8_*, 600 MHz, 298 K), **9** (THF‐*d_8_*_,_ 600 MHz, 298 K), **10** (THF‐*d_8_*_,_ 600 MHz, 298 K), and **11** (CDCl_3_, 600 MHz, 298 K). *: CDCl_3_. Proposed assignments for signals corresponding to multiple nuclei are separated by a slash. For full spectra, see the Supporting Information.

The key reaction is the Scholl‐type oxidation to transform **7** to **8–11** [9, 87]. The first conditions applied were typical conditions with DDQ and CH_3_SO_3_H to facilitate the cyclodehydrogenation reaction. After 1.5 h at room temperature, *C*_2_‐symmetric fourfold cyclized product **8** was obtained as a purple solid in 68% yield (Scheme 2). Well‐resolved signals of the ^1^H NMR spectra were observed in THF‐*d_8_*, allowing to clearly elucidate the structure and the sites where cyclodehydrogenation occurred. By cyclodehydrogenation, two dibenzopyrene units were generated. The protons of these subunits (green labeled signals 4–12) could be assigned by 2D NMR spectroscopy (for details, see Figures S29–S34). For instance, the outmost protons of these units (H8) appeared as a triplet at *δ* = 8.00 ppm (*J* = 7.8 Hz), coupling to protons H7 (*δ* = 8.95 ppm, *J* = 8.0 Hz) and H9 (*δ* = 8.87 ppm, *J* = 7.8 Hz). A characteristic signal is that of the methine proton H3 of the heptagon ring, which resonates as a singlet at *δ* = 8.52 ppm. Single crystals of PAH **8** of suitably quality for X‐ray diffraction were grown by diffusion of MeOH into a hexane solution of **8**, unambiguously confirming the molecular structure elucidated by NMR spectroscopy (see discussion below).

When the cyclodehydrogenation was performed with DDQ in the presence of stronger Brønsted acid TfOH with a concentration of 1/200 (v/v), after stirring at 0°C for 5 min, a green solid was obtained in 61% yield. The MALDI‐TOF mass spectrum indicated a loss of twelve protons with an exact mass of *m/z* 1914.0710 (calculated *m/z* 1914.0676), which is related to a six‐fold cyclized product (Figure S68). This suggests that PAH **9** has a less symmetric structure. And indeed, the ^1^H NMR spectrum is more complex than that for more symmetric PAH **8**. Again, with the aid of 2D NMR spectroscopy, all peaks in the ^1^H NMR spectrum were assigned (for details, see Supporting Information). There are three sets of dibenzopyrene units formed in PAH **9**, as for instance is reflected by three similar triplets of the protons of the dibenzopyrene tips at *δ* = 8.01 ppm (H8), *δ* = 8.13 ppm (H33), and *δ* = 8.16 ppm (H42). By NOESY NMR, cross‐correlation between H12 and H13 was found (see Figure S38), confirming the cyclodehydrogenation sites and supporting the proposed structure as drawn. It should be noted that the formation of 7‐fold and 8‐fold cyclodehydrogenation products was also observed by MALDI‐TOF mass spectrometry under these conditions. However, these formed only in traces (<1%) and could not be isolated in reasonable amounts to be characterized.

Nevertheless, the 7‐fold cyclodehydrogenation product could be selectively obtained by using Lewis acid FeCl_3_ in CH_3_NO_2_ instead of DDQ in combination with a Brønsted acid, resulting in PAH **10** as a dark purple solid with a yield of 65%. The MALDI‐TOF mass spectrum indicated the formation of seven C─C bonds with the observed exact mass of *m/z* 1912.0526 (Figure S69), matching the calculated *m/z* of 1912.0519. As PAH **9**, PAH **10** shows in the ^1^H NMR spectrum three formed dibenzopyrene units, H42 (*δ* = 8.14 ppm), H33 (*δ* = 8.00 ppm), and H8 (*δ* = 8.10 ppm). Nuclear Overhauser correlation (NOE) between H12 and H13 was found, and H25 appeared at *δ* = 7.25 ppm, showing a ^1^H,^1^H‐COSY correlation with H13 (Figures S43 and S44). These indicate that the seventh C─C bond was not formed at either C13 or C25. Accordingly, the cyclization is restricted to take place between C1 and C46. The result suggests that cyclization is likely promoted by the formation of an extended π‐conjugated system and may account for the difficulty in achieving the more symmetric 8‐fold cyclized product.

When the Scholl reaction was conducted with twelve equivalents of DDQ together with TfOH at a concentration of 1/30 (v/v) in dry DCM, a brown solid was separated after column chromatography in 35% yield (Scheme 2). MALDI‐TOF mass spectrometry revealed an exact mass of *m/z* 2201.8935 (Figure S70), suggesting that the product **11** (calc. *m/z* 2201.8934) was generated by a 10‐fold cyclodehydrogenation of precursor **7**, accompanied by an additional two‐fold oxytriflation. [36, 86, 88, 89] The introduction of oxytriflate moieties was supported by ^19^F NMR spectroscopy, where the CF_3_ signals appeared at *δ* = −72.34 ppm (Figure S53). The ^1^H NMR spectrum of PAH **11** in CDCl_3_ is much simpler compared to the other ones, reflecting its higher symmetry by having all 10 C─C bonds closed. There are five singlets at *δ* = 1.47, 1.42, 1.34, 1.32, and 1.26 ppm in the aliphatic region for the five different tert‐butyl groups (Figure S47), which is consistent with the pattern observed for compound **8**, suggesting that **11** also possesses *C_2_* symmetry. Only one triplet (H6) was observed, implying that one of the protons of the two symmetrically inequivalent dibenzopyrene units has been substituted by a triflate group at the tips or adjacent to it. The absence of an NOE between H13 and protons of the adjacent benzene ring (Figure S50) supports that the OTf groups are located at carbon *a* as shown in Scheme 2.

### 2.2 Single‐Crystal X‐Ray Structure Analysis

**FIGURE 1:**
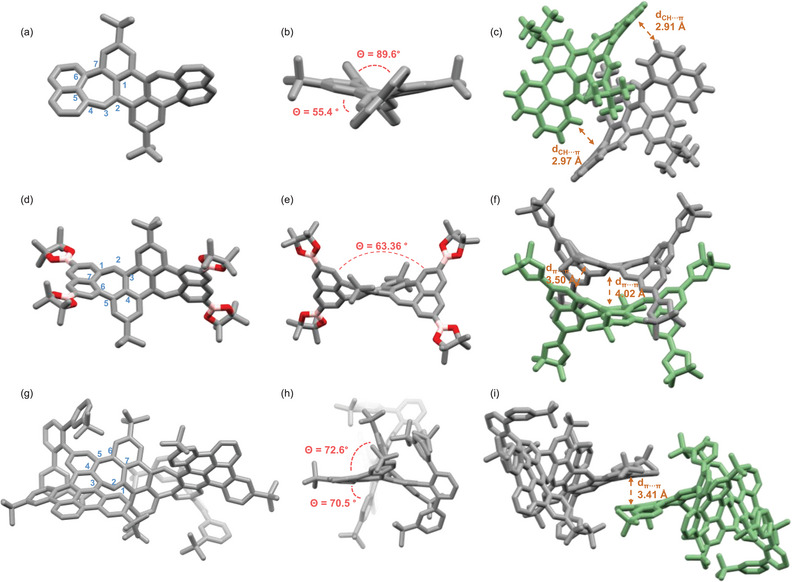
Single‐crystal X‐ray structures of (a–c) PAH **4**, (d–f) tetraboronic ester **5**, and (g–h) [4]helicene **8** as ORTEP drawing with probability of ellipsoids of 25%. (c, f, i) Packing of PAHs **4**, **5**, and **8** in the unit cell. (*P*,*P*)‐conformers are displayed in grey, and (*M*,*M*)‐conformers are colored in green. Except for (c), all hydrogens are omitted for clarity.

From PAH **4**, tetraboronic ester **5,** and PAH **8** single crystals were grown that were sufficient for X‐ray diffraction analysis. Due to the embedded heptagons, all have a twisted saddle‐shaped backbone (Figure 1). The naphthalene units of PAH **4** are significantly distorted from the mean planes of the anthracene core with a dihedral angle of 55.4°. The two naphthalene units form a dihedral angle of 89.5° (Figure 1b). It is clearly visible that borylation did not change the twist of the system but formed a wider twisted angle of 63.4° between the mean planes of two naphthalene units as a result of π extension, as shown in Figure 1e. The perylene units of PAH **8** also show strong twisting to the anthracene core with angles of 70.5° and 72.7°, respectively (Figure 1h). Analysis of bond length in the heptagonal ring reveals that the C2─C3 bond in PAH **4**, the C2─C3 bond in tetraboronic ester **5**, and the C1─C2 bond in PAH **8** are the shortest bonds with 1.35, 1.38, and 1.35 Å, respectively, revealing some olefinic character (Figure 1a,d,g). It is worth noting that this corroborates with calculations of nucleus‐independent chemical shifts (NICS) and induced current density (ACID) calculations (see Table S1).

All compounds are chiral and crystallize as racemates. (*M*)‐**4** and (*P*)‐**4** show weak CH···π interactions between two naphthalene units with a closest distance of 2.91 Å (Figure 1c). For the tetraboronic ester **5**, a pair of enantiomers, (*M*)‐**5** and (*P*)‐**5**, was found to pack as dimers in the asymmetric unit. The two molecules arrange face to face with an interplanar distance varying slightly from 3.50 Å between the naphthalene units to 4.02 Å between the central anthracene fragments (Figure 1f). The π─π stacking between two (*M*)‐**8** and (*P*)‐**8** was observed with a distance of 3.41 Å between dibenzopyrene units (Figure 1i). The weak π‐stacking also explains the excellent solubility in most organic solvents [90, 91]. There are no *meso*‐configurated compounds observed in the single crystal structures.

### 2.3 Electrochemical and Optoelectronic Properties

The electrochemical properties of compounds **8–11** were studied by cyclic voltammetry (CV) and differential pulse voltammograms (DPV) in dichloromethane using *n*‐Bu_4_NPF_6_ as the supporting electrolyte and calibrated by ferrocenium/ferrocene (Fc^+^/Fc) (Figures S83–S86). All the PAHs exhibit multiple reversible electrochemical oxidations. For PAH **8**, the first oxidation/reduction potentials were determined to be 0.35 and −1.79 V, respectively. Accordingly, the electrochemical band gap was estimated to be 2.1 eV (Table 1). For the [6]helicenes, quite similar potentials of the first oxidation peaks were observed (0.08 V for **9**, 0.09 V for **10**, and 0.07 V for **11**). The reduction potentials are shifted toward more negative values (−1.91 V for **9**, −2.03 V for **10**, −2.02 V for **11**) compared to that of PAH **8**. This decreased electron affinity is likely due to the structural distortion caused by the [6]helicene unit, which reduces the orbital overlap. The molecular orbital analysis reveals that the HOMOs of **8–11** are primarily delocalized in the central backbone of the molecules (Figure S97), which may account for the relatively small difference in oxidation potentials.

**TABLE 1:** Summary of the photophysical and electrochemical properties of PAHs **8–11**.

Compound	*λ*_max_ ^a^ [nm]	*λ*_em_(λ_ex_) [nm]	E_gap,opt_ ^b^ [eV]	E_IP,CV_ ^c^ [eV]	E_EA,CV_ ^c^ [eV]	E_gap,CV_ ^c^ [eV]	E_HOMO,DFT_ ^d^ [eV]	E_LUMO,DFT_ ^d^ [eV]	E_diff,DFT_ ^d^ [eV]	*ṽ* [cm^−1^]	*g*_abs_
**8**	541	605 (540)	2.0	−5.1	−3.0	2.1	−4.43	−2.09	2.3	1955	—
**9**	589	548 (450)	1.7	−4.9	−2.9	2.0	−4.30	−2.28	2.0	−1279	6.19 × 10^−4^ (454 nm)
**10**	581	604, 619 (510)	2.0	−4.6	−2.7	2.2	−4.37	−2.16	2.2	655	1.89 × 10^−3^ (574 nm)
**11**	583	604 (490)	1.7	−4.7	−2.8	2.0	−4.59	−2.65	1.9	596	2.42 × 10^−3^ (589 nm)

^a^ Measured in CH_2_Cl_2_ at room temperature.

^b^ Estimated from the absorption onset.

^c^ E^CV^ = −(E_red/Ox_ + 4.8 eV), estimated from the difference between half‐wave oxidation potential and reduction potential.

^d^ Calculated by DFT at the B3LYP/6‐31G^*^ level of theory.

**FIGURE 2:**
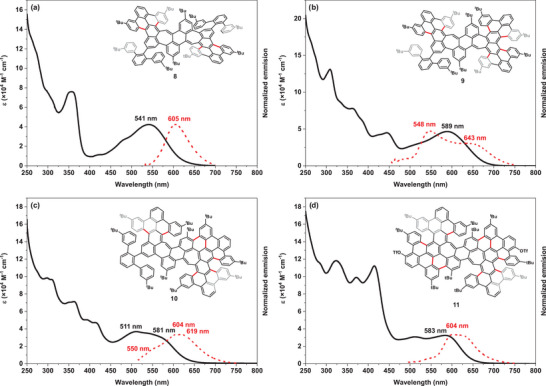
UV–vis absorption (solid) and normalized fluorescence spectra (dash) of helicenes **8–11** in CH_2_Cl_2_.

Optoelectronic properties of all compounds have been investigated using UV–vis absorption and emission spectroscopy (Table 1 and Figure 2). All absorption and emission spectra were recorded in dichloromethane solution. The main absorption band of PAH **8**, with its absorption maximum at *λ* = 541 nm, is bathochromically shifted in comparison to that of precursor **7** (*λ* = 427 nm, Figure S74), attributed to the extension of π‐conjugation. PAH **10** features broad absorption bands with a dominant peak at *λ* = 511 nm and a lowest‐energy maximum at *λ* = 581 nm, suggesting overlapping electronic transitions due to enhanced conjugation and a lower symmetry. For PAH **11**, a further red‐shifted absorption band with a maximum at *λ* = 583 nm was observed. The general trend of a decreasing optical band gap with increasing conjugation is disrupted by the highly flexible structure **9**, which exhibits the most red‐shifted absorption maximum at *λ* = 589 nm, r. To investigate the absorption phenomenon, time‐dependent density functional theory (TD‐DFT) calculations were performed at the CAM‐B3LYP/6‐31G* (SCRF, CH_2_Cl_2_) level of theory (Figures S101–S104). According to the results, the broad absorption bands of all PAHs in the visible light region are primarily ascribed to the transitions from the highest occupied molecular orbital (HOMO) to the lowest unoccupied molecular orbital (LUMO). Attention was then focused on the emission behavior. Upon excitation, PAH **8** displays photoluminescence with an emission maximum at *λ*_em_ = 605 nm with a Stokes‐shift of *ṽ* = 1955 cm^−1^. PAH **10** shows a broad and unresolved emission peak at *λ*_em_ = 604 nm, with a Stokes shift of *ṽ* = 655 cm^−1^. Similarly, PAH **11** shows an unresolved emission peak with a narrower Stokes shift of *ṽ* = 596 cm^−1^. In contrast to PAH **8**, **10**, and **11**, product **9** presents an exceptional emission spectrum with a peak maximum at *λ*_em_ = 548 nm and a shoulder around *λ*_em_ = 650 nm when excited at 450 nm. This extraordinary blue‐shifted emission relative to its absorption maximum at *λ*_abs_ = 589 nm leads to an unusual anti‐Stokes shift of *ṽ* = −1279 cm^−1^ [92]. To exclude any aggregation effects, the excitation‐dependent emission spectra of PAH **9** were subsequently recorded at low concentrations (absorbance <0.1, Figure S76). Excitation at *λ*_ex_ = 530 nm resulted in emission only at longer wavelengths (*λ*_em,2_ = 643 nm), whereas excitation at *λ*_ex_ = 510 nm led to the emergence of a higher‐energy emission peak (*λ*_em,1_ = 548 nm). This observation suggests the existence of two independent excited states, with the higher‐energy excited state being predominant.

To investigate this intriguing phenomenon, photoluminescence (PL) and time correlated single‐photon counting (TCSPC) were further examined for PAHs **8–10** (Figures S79 and S80). PAHs **8** and **10** show similar PL spectral shapes, while PAH **9** shows larger shifts upon changing the excitation wavelength, with the band at *λ* = 650 nm being greatly reduced in intensity in comparison to the main band at *λ* = 550 nm. The kinetic traces at different emission wavelengths within the photoluminescence spectra (Figure S79d–f) were used to determine emission lifetimes with biexponential fits. For PAH **9** (Figure S79e), two types of photoluminescence decay kinetics are found at detection wavelengths of 500 nm (blue side of the PL spectrum) and 670 nm (red side of the PL spectrum). This agrees with a process in which the total PL emission results from two different excited state populations. Notably, the high‐energy PL kinetics show longer lifetimes than the low‐energy ones. In addition, there is no increase in the intensity of the low‐energy species on the studied time scales.

### 2.4 Computational Studies

**FIGURE 3:**
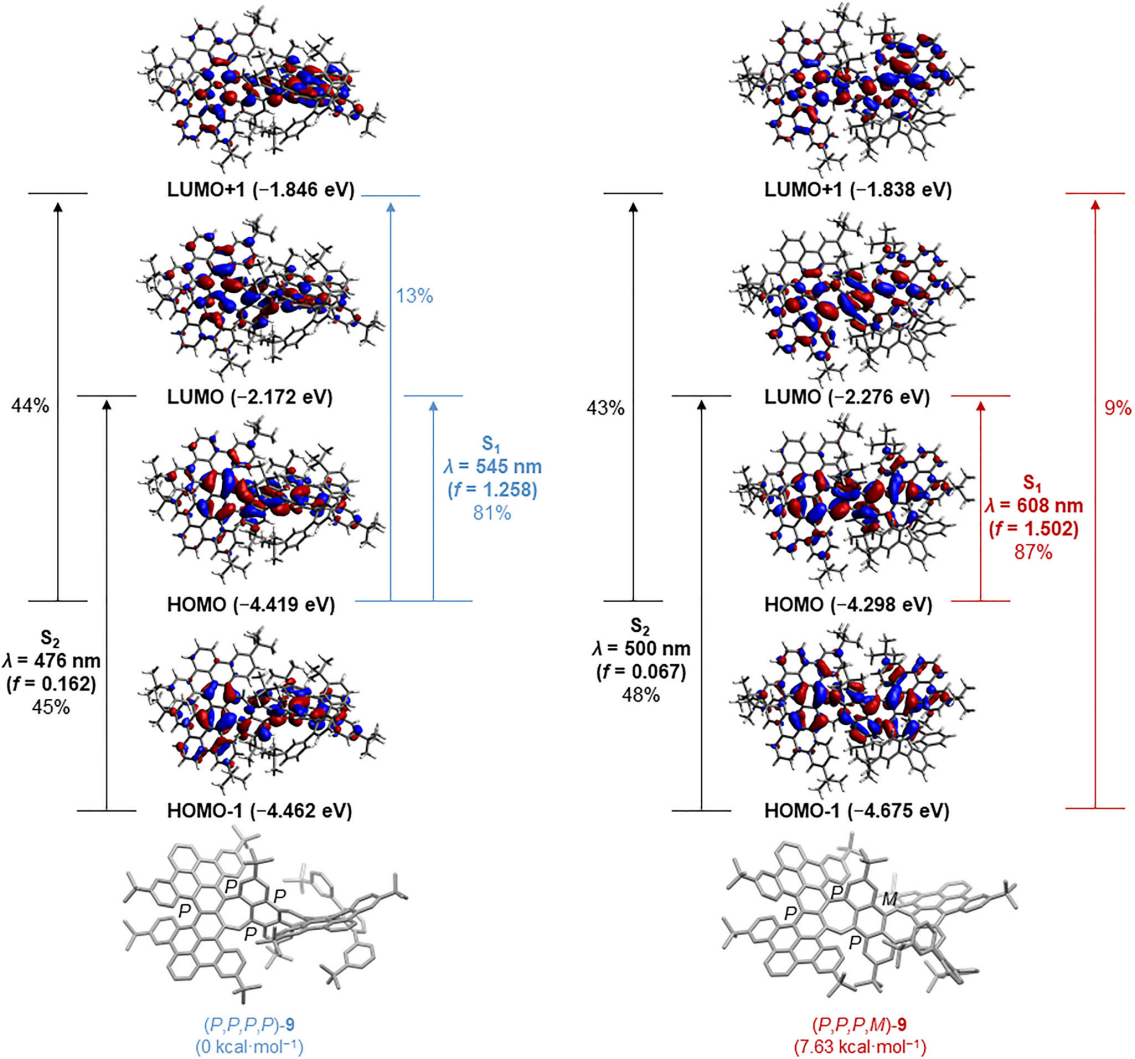
DFT‐calculated frontier molecular orbitals (B3LYP/6‐31G*) and TD‐DFT‐calculated electronic transitions (CAM‐B3LYP/6‐31G*, SCRF = CH_2_Cl_2_) for two suggested conformers of compound **9**. The S_0_→S_1_ and S_0_→S_2_ transitions and their corresponding oscillator strengths (*f*) are presented. The percentage values represent the contributions of specific orbital transitions to each excited state.

To gain deeper insight into this unusual phenomenon, DFT calculations were performed. A notable discrepancy was observed between the calculated HOMO–LUMO gap of (*P,P,P,P*)‐**9** (2.2 eV) and the experimentally determined optical band gap (1.7 eV). This inconsistency indicates that the all‐*P* conformer is not likely to fully account for the observed absorption behavior, implying the involvement of other conformers. From the emission spectrum of PAH **9** (Figure 2b), two emission processes correspond to two excited states with transition energies of 2.26 eV (*λ*_em_ = 548 nm) and 1.93 eV (*λ*_em_ = 643 nm). Thus, based on these results, we propose that two different conformers are involved in this emission process, (*P,P,P,P*)‐**9** and (*P,P,P,M*)‐**9** (Figure 3). According to calculations, the (*P,P,P,P*) conformer is the energetically most favored, and (*P,P,P,M*)‐**9** is 7.63 kcal∙mol^−1^ higher in energy than (*P,P,P,P*)‐**9**. The corresponding HOMO‐LUMO gap of (*P,P,P,M*)‐**9** was calculated to be 2.05 eV, which is in good agreement with the observed lower‐energy emission process (1.93 eV). Based on the TD‐DFT results, (*P,P,P,M*)‐**9** shows a lower vertical excitation energy ($\Delta E_{S_{0}-S_{1}}^{\left(P,P,P,M\right)}$ S_1_ = 2.04 eV, *λ*_abs_ = 608 nm) and higher oscillator strength ($f_{S_{0}-S_{1}}^{\left(P,P,P,M\right)}$ = 1.50) compared to (*P,P,P,P*)‐**9** ($\Delta E_{S_{0}-S_{1}}^{\left(P,P,P,P\right)}$ = 2.27 eV, *λ*_abs_ = 545 nm, $f_{S_{0}-S_{1}}^{\left(P,P,P,P\right)}$ = 1.26). This suggests that absorption from S_0_ to S_1_ is more likely to occur in (*P,P,P,M*)**‐9**, resulting in a dominant absorption band at roughly *λ*_abs_ = 589 nm. However, (*P,P,P,P*)‐**9** possesses a 2.3 kcal∙mol^−1^ lower electronic energy at the S_1_ state than that of (*P,P,P,M*)‐**9**, suggesting that in the first excited‐state at room temperature, inversions of (*P,P,P,M*)‐**9** to (*P,P,P,P*)‐**9** are possible. In addition, the transitions from S_0_ to S_2_ of both conformers give relatively high emission energies with relatively low oscillator strength compared to that of the observed emission: $\Delta E_{S_{0}-S_{2}}^{\left(P,P,P,P\right)}$ = 2.60 eV, $f_{S_{0}-S_{2}}^{\left(P,P,P,P\right)}$= 0.06, $\Delta E_{S_{0}-S_{2}}^{\left(P,P,P,M\right)}$= 2.48 eV and $f_{S_{0}-S_{2}}^{\left(P,P,P,M\right)}$ = 0.07), which excludes the possibility that this observed main emission originates from a higher energetic state S_n_. Therefore, the main origin of the dual emission appears to arise from two conformers with distinct photophysics. We hypothesized that an additional conformational inversion during the excitation may account for some of the observed anti‐Kasha‐ like behavior of PAH **9**. The estimated energy barrier for the isomerization process is calculated to be about 14 kcal/mol (see Figures S97–S99).

### 2.5 Chiroptical Properties

**FIGURE 4:**
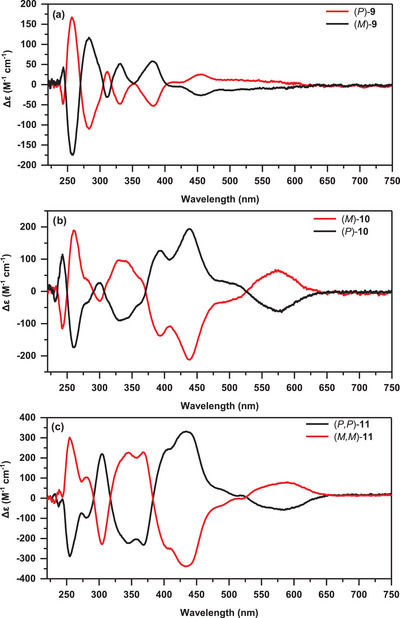
CD spectra of the separated enantiomers of helicenes **9–11**.

To our delight, the enantiomers of helicenes **9**, **10**, and **11** were successfully separated using HPLC with a chiral stationary phase (Figures S88–S93). The circular dichroism (CD) spectra (Figure 4) reveal that each pair of enantiomers for all the PAHs exhibits nearly perfect mirror‐symmetric behavior with opposite Cotton effects. It should be noted here that the chiral descriptors (*P*/*M*) used for chiral resolution and CD spectra exclusively refer to the [6]helicene units. The absolute configurations were assigned on the basis of the TD‐DFT simulated CD spectra, as shown in Figures S105–S107. PAH **9** shows the lowest Δε value of ±25 M^−1^·cm^−1^ at *λ* = 454 nm with an absorption dissymmetry factor of *g*_abs_ = 6.19 × 10^−4^ at the same wavelength, which is comparable with that of single [6]helicene [93]. The π‐extended structure **10** shows an increased Δε value of ± 194 M^−1^·cm^−1^ at *λ* = 437 nm, and PAH **11** has the highest Δε value of ±330 M^−1^·cm^−1^ at *λ* = 433 nm among all the PAHs. The *g*_abs_ values of the longest‐wavelength absorptions are 1.89 × 10^−3^(*λ* = 574 nm) for **10** and 2.42 × 10^−3^(*λ* = 589 nm) for **11**, which are more than twice as large as that of the parent [6]helicene **9**, and are in good agreement with the reported double carbo[6]helicenes and π‐extended [6]helicenes, whose *g*_abs_ values are generally in the order of 10^−3^ [53].

## 3 Conclusions

In conclusion, we presented a series of multiple helicenes with a highly contorted backbone bearing two heptagons. The heptagonal units were constructed via Knoevenagel condensation, while helicene moieties were formed by fine‐tuning the conditions of the Scholl reaction. With DDQ/MeSO_3_H, a fourfold cyclization occurred, giving symmetric PAH **8**. Changing the acid to TfOH resulted in a six‐fold cyclization, leaving one terphenyl moiety unreacted to give PAH **9**. Increasing the amount of TfOH sixtyfold gave a 10‐fold cyclodeydrogenation, accompanied by oxytriflation (PAH **11**). Using FeCl_3_ as the oxidant leads to an unusual unsymmetric structure (PAH **10**). The optical and electronic properties were investigated by UV–vis absorption, fluorescence spectroscopy, and cyclic voltammetry. In addition, one member of the series, PAH **9**, which possesses a high conformational flexibility, exhibits unusual anti‐Kasha‐like behavior. Assisted by DFT calculations, we conclude that this unusual phenomenon is likely associated with conformational conversion upon excitation. Moreover, for PAH **9**, **10**, and **11**, a pair of enantiomers for each product was separated by chiral HPLC and characterized by circular dichroism spectroscopy. Further and more detailed investigations into this anti‐Kasha‐like behavior of compound **9** are ongoing and will be reported in due course.

## Conflicts of Interest

The authors declare no conflicts of interest.

## Supporting information




**Supporting File 1**: anie71781‐sup‐0001‐SuppMat.pdf.

## Data Availability

The data that support the findings of this study are available from the corresponding author upon reasonable request.
