# Supplementary material for: Chiral Heptagon‐Embedded Double [6]Helicenes via Scholl Reaction
Source: Angew Chem Int Ed Engl. 2026 Mar 13;65(16):e8159257. doi: 10.1002/anie.8159257 (PMC13080427; doi:10.1002/anie.8159257)
Supplement: Supplementary file 1 — Supporting File 1: anie71781‐sup‐0001‐SuppMat.pdf. [file ANIE-65-e8159257-s001.pdf]

Chiral Heptagon-Embedded Double [6]Helicenes via Scholl Reaction

Dan Wang, [a] Philipp Penert, [a] Lars Schneider, [b] Moritz P. Schuldt, [a] Frank Rominger, [a] Felix Deschler, [b] and Michael Mastalerz*[a]

[a] D. Wang, P. Penert, M. P. Schuldt, Dr. F. Rominger, Prof. Dr. M. Mastalerz
Organisch-Chemisches Institut, Ruprecht-Karls-Universität Heidelberg, Im Neuenheimer Feld 272, 69120, Heidelberg (Germany)
E-mail: michael.mastalerz@oci.uni-heidelberg.de

[b] L. Schneider, Prof. Dr. F. Deschler
Physikalisch-Chemisches Institut, Ruprecht-Karls-Universität Heidelberg, Im Neuenheimer Feld 229, 69120, Heidelberg (Germany)

Supporting Information

Table of Contents

1. General remarks S3

2. Experimental Procedures S4

3. NMR Spectra S11

4. FT-IR Spectra S38

5. Mass Spectra S42

6. UV-vis and Fluorescence Spectra S46

7. Cyclic Voltammograms and Differential pulse voltammograms S51

8. Chromatograms S57

9. Computational Details S60

9.1. Conformational Studies S60

9.2. Frontier Molecular Orbitals S64

9.3. TD-DFT Calculations S64

9.4. Nucleus-independent Chemical Shift (NICS) S67

9.5. Anisotropy of the Induced Current Density (AICD) S72

10. Crystallographic Data S75

11. Reference S78

1. General remarks

Materials: All the reagents and solvents were purchased from Sigma Aldrich, Acros Organics, BLDPharm, Carbolution, Fisher Scientific, Thermo Fisher, Honeywell, Grüssing, Merck, TCI, Eurisotop or VWR Chemicals and used without further purification, if not mentioned otherwise. Dry DCM and THF were obtained from Honeywell and purified by a solvent purification system MB SPS-800.

Thin layer and flash column chromatography: Thin layer chromatography was performed on fluorescent-labeled silica gel 60 coated aluminum plates from Macherey Nagel. Spots were detected with UV-light irradiation (λ_{ex} = 254, 365 nm). Flash column chromatography was carried out with silica gel 60 (particle size: 0.04–0.063 mm) purchased from Macherey Nagel.

High-performance liquid chromatography (HPLC): Analytical chiral HPLC was performed on a Shimadzu LC-40 Nexera HPLC-System and semi-preparative chiral HPLC was performed on a Shimadzu LC-20A Prominance recycling HPLC-System. Both systems use Chiralpak® IA and IC columns from Daicel. Detection was accomplished at 254 nm.

Melting point (m.p.): The non-corrected melting points were determined with a Büchi Melting Point B-545 with a maximum measurement temperature of 400 °C.

Nuclear magnetic resonance (NMR): NMR spectra were recorded on Bruker Avance III 400 (400 MHz), Bruker Avance III 600 (600 MHz) or Bruker Avance Neo 700 (700 MHz) spectrometer. Abbreviations: s = singlet, d = doublet, t = triplet, m = multiplet. Chemical shifts (δ) are reported in parts per million (ppm) relative to traces of $CHCl_3$ (δ_H = 7.26 ppm, δ_C = 77.0 ppm) or tetrahydrofuran (δ_H = 1.72, 3.58 ppm, δ_C = 67.21, 25.31 ppm) in the corresponding deuterated solvent.[S1] Signals were assigned by 2D NMR spectra (^{1}H,^{1}H-COSY, ^{1}H,^{1}H-NOESY, ^{1}H,^{13}C-HSQC, ^{1}H,^{13}C-HMBC). Proposed assignments for signals corresponding to multiple atoms are separated by a slash.

Mass spectrometry (MS): Mass spectra were recorded on a Bruker timsTOFfleX spectrometer or a Bruker Autoflex speed MALDI-TOF spectrometer. DCTB (trans-2-[3-(4-tert-Butylphenyl)-2-methylpropenylidene)malononitrile) was used as matrix. The mass peaks for all compounds are the most abundant ion peaks.

Infrared spectroscopy (IR): Fourier transform infrared spectroscopy (FT-IR) spectra were recorded on Bruker Tensor 27 spectrometer equipped with a ZnSe ATR crystal. Absorption bands $\tilde{\nu}$ were reported in cm^{-1}, and the signal intensities were described as s = strong, m = medium, w = weak. v = very.

UV-vis and fluorescence spectroscopy: Absorption spectra were recorded on a Jasco UV-VIS V-730. Emission spectra were recorded on a Jasco FP-8300.

Cyclic voltammetry (CV): Cyclic voltammetry and differential pulse voltammetry (DPV) were carried out with a Metrohm Autolab PGSTAT101 potentiostat using a glassy carbon (disc geometry, 3.14 mm^2) working electrode, a Pt counter electrode and an Ag/Ag^+ pseudo reference electrode. Ferrocene/ferrocenium was used as an internal standard for calibration. The measurements were performed in a 0.1 M tetrabutylammonium hexafluorophosphate (NBu_4PF_6) solution in degassed HPLC-grade dichloromethane and a ~1 mM concentration of the analyte. Cyclic voltammograms were obtained at a scan rate of 100 mV·s^{-1}. Differential pulse voltammograms were recorded with a step size of 0.005 V, a modulation amplitude of 0.025 V, a modulation time of 0.05 s and an interval time of 0.5 s. All measurements were performed at room temperature.

Circular dichroism (CD) spectroscopy: CD spectra were recorded with a Jasco J-1500 CD spectrometer.

Elemental analysis: Elemental analysis was performed by the Microanalytical Laboratory of the University of Heidelberg with an Elementar vario Micro Cube Element Analyzer.

X-ray crystal structure analysis: The X-ray structure analysis was accomplished on a STOE Stadivari using Cu-Kα radiation (λ = 1.54178 Å) or a Bruker APEX II Quazar diffractometer using Mo-Kα radiation (λ = 0.71073 Å). Intensities were corrected for Lorentz and polarization effects. An empirical scaling and absorption correction was applied using X-Area LANA 2.7.12 (STOE, 2023), or 2.8.4 (STOE, 2024) based on the Laue symmetry of the reciprocal space, mu = 0.57 mm^{-1}, T_{min} = 0.89, T_{max} = 0.94. The structures were solved with SHELXT-2014[S2] and refined refined against F^2 with a Full-matrix least-squares algorithm using the SHELXL-2019/2 software[S3].

Computational Details: All quantumchemical calculations were performed by employing the Gaussian16 program packages.[S4] Structure optimizations and frequency calculations were performed at the B3LYP/6-31G* level of theory.[S5-8] The ground state exhibited no imaginary frequency. The mechanistic calculations for the inversion of the backbone in compound **9** and a simplified version of it were performed at PM6 level of theory.[S9] The transition states were confirmed by frequency calculations and exhibit exactly one imaginary frequency. For isomerizations, where no exact transition state was found, the maximum energy of a scan corresponding to this isomerization is reported as an upper bound for the transition state energy. Time-dependent DFT (TDDFT) calculation was done with the range-separated hybrid functional CAM-B3LYP/6-31G*.[S10] The calculated absorption and CD spectra were evaluated in Avogadro with Orca 4.1 support, exported, and plotted in OriginPro. Nucleus-independent chemical shift (NICS)[S11] were calculated using the gauge-independent atomic orbital (GIAO)[S12-16] method. Anisotropy of the induced current density (ACID) plots were calculated by Herges's method[S17-18]. The calculations only included the contributions from occupied π-orbitals by analyzing the compositions of the molecular orbitals using the Multiwfn 3.6 software.[S19]

Time-correlated single photon counting (TCSPC): The excitation-dependent photoluminescence spectra and time-resolved photoluminescence measurements were measured on a commercial TCSPC setup (*Lifespec II* by *Edinburgh Instruments*) with the samples dissolved in dichloromethane in a 10 mm quartz cuvette. The samples were excited by pulsed diode lasers (*EPL* by *Edinburgh Instruments*) at 375 nm, 475 nm and 635 nm, and a pulse period of 100 ns. The time-resolved measurements were taken at multiple wavelengths within the PL spectra with a $\Delta\lambda$ cutout of 5 nm. The lifetimes were derived from bi-exponential fit.

2. Experimental Procedures

Boronic acid pinacol ester **1**[S20] and 8-bromo-1-naphthaldehyde **2**[S21-22] were synthesized according to the literature.

Compound 3

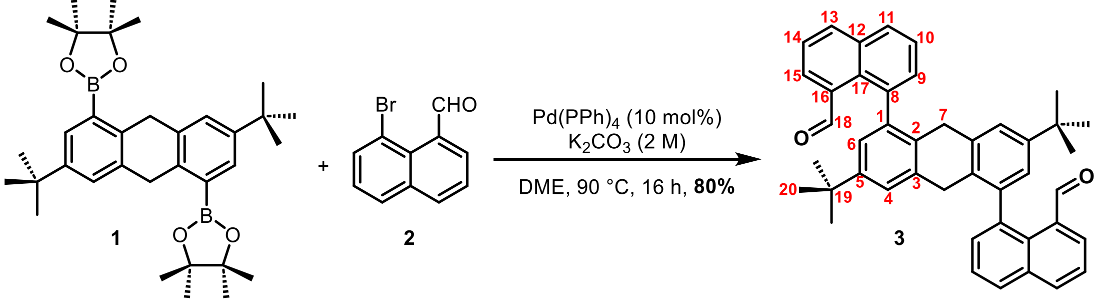

In a 25 mL screw-capped vial under an argon atmosphere, 544 mg (0.50 mmol) boronic acid pinacol ester **1** and 823 mg (1.75 mol) of 8-bromo-1-naphthaldehyde were dissolved in degassed DME (3 mL). Degassed aqueous 2M K_2CO_3 solution (1 mL) was added, followed by the addition of 58 mg (50 μmmol) of $Pd(PPh_3)_4$. The reaction mixture was further degassed

with argon for 5 minutes. The vial was sealed and the reaction mixture was stirred at 90 °C overnight. After cooling down to room temperature, the aqueous phase was extracted with DCM (3 × 10 mL) and the extracts combined. The organic layer was dried over $MgSO_4$ and the solvent was removed by rotary evaporation. The crude product was purified by flash column chromatography (SiO_2, PE/EA 20/1) to obtain **3** as a slightly yellow solid (480 mg, 80%, mixture of atropisomers). ***R*f** = 0.18, 0.12 (PE/EA 10:1). **m.p.** 300 °C (decomp.).**^{1}H NMR** (600 MHz, $CDCl_3$) *δ* 9.25 (s, 2H, H-18), 8.17 (dd, *J* = 8.3, 1.7 Hz, 2H, H-13), 8.03 (d, *J* = 1.3 Hz, 2H, H-11), 7.90 (d, *J* = 1.4 Hz, 2H, H-15), 7.71 (dd, *J* = 8.2, 7.0 Hz, 1H, H-10), 7.68 (dd, *J* = 8.2, 6.9 Hz, 1H, H-10), 7.64 (dd, *J* = 7.0, 1.4 Hz, 1H), 7.62 – 7.58 (m, 2H), 7.54 (dd, *J* = 6.9, 1.3 Hz, 1H), 7.22 (d, *J* = 2.1 Hz, 1H), 7.15 (d, *J* = 2.0 Hz, 1H), 7.05 (d, *J* = 2.0 Hz, 2H, H-4), 3.69 (s, 2H, H-6), 3.61 – 3.49 (m, 2H, H-6), 1.22 (s, 9H, H-20), 1.20 (s, 9H, H-20).**^{13}C NMR** (151 MHz, $CDCl_3$) δ 192.28 (C-18), 192.00 (C-18), 150.08 (C-5), 149.90 (C-5), 140.89 (C-16), 140.85 (C-16), 138.32 (C-8), 138.25 (C-8), 137.94 (C-3), 137.68 (C-3), 136.39 (C-1), 136.26 (C-1), 134.87 (C-12), 134.73 (C-12), 134.44 (C-13), 134.31 (C-13), 131.81 (C-2), 131.52 (C-2), 131.46 (C-17), 130.71 (C-17), 130.22 (C-9), 129.58 (C-9), 129.33 (C-15), 128.86 (C-15), 128.81 (C-11), 126.39 (C-11), 126.26 (C-10), 125.80 (C-10), 125.67 (C-14), 125.63 (C-14), 125.54 (C-6), 125.04 (C-6), 125.01 (C-4), 34.73 (C-19), 34.69 (C-19), 34.62 (C-7), 34.41 (C-7), 31.59 (C-20), 31.54 (C-20).**IR (RT, ATR)** $\tilde{\nu}$ (cm^{-1}): 2959 (w), 2912 (vw), 2866 (w), 1732 (w), 1680 (s), 1605 (w), 1574 (w), 1504 (w), 1474 (w), 1464 (w), 1418 (w), 1398 (w), 1362 (w), 1231 (w), 1167 (w), 1101 (w), 930 (w), 831 (m), 775 (vs), 717 (w), 650 (w).**UV-vis** (CH_2Cl_2) λ_{max} (lg ε) 323 (4.21) nm. **Fluorescence** (CH_2Cl_2) λ_{em} (λ_{ex}): 375 (330) nm. **MALDI HRMS** (DCTB) (*m*/*z*): calcd. for $[M+Na]^+$: 623.2926; found: 623.2918.

Compound 4

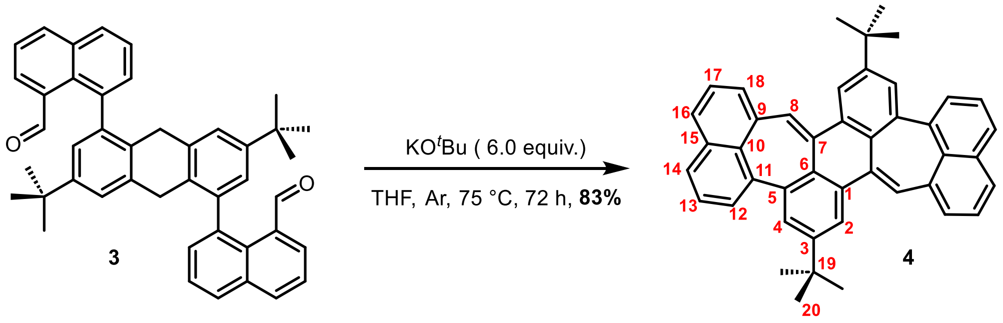

In a glovebox, the aldehyde **2** (430 mg, 0.72 mmol) was dissolved in dry THF (10 mL) in a 25 mL vial. A solution of KO*t*Bu (482 mg, 4.3 mmol) in dry THF (2 mL) was added. The vial was then sealed, and the reaction mixture was stirred at 75 °C overnight. After cooling down to room temperature, the reaction was quenched with water (15 mL). The aqueous phase was extracted with DCM (3 × 15 mL) and the combined organic layers were dried over anhydrous $MgSO_4$. The solvent was removed under reduced pressure, and the crude product was purified via flash column chromatography (SiO_2, PE/EA, 50/1) to obtain **4** as a yellow solid (335 mg, 83%). ***R*f** = 0.40 (PE/EA 10:1). **m.p.** 375–380 °C. **^{1}H NMR** (600 MHz, $CDCl_3$) *δ* 7.74-7.71 (m, 4H, H-2, H-14), 7.66-7.63 (m, 4H, H-12, H-17), 7.53 (d, *J* = 7.7 Hz, 2H, H-13), 7.41-7.38 (m, 4H, H-16, H-18), 7.18 (d, *J* = 1.9 Hz, 2H, H-4), 7.13 (s, 2H, H-8), 1.35 (s, 18H, H-20). **^{13}C NMR** (151 MHz, $CDCl_3$) *δ* 152.32 (C-3), 139.51 (C-11), 139.31 (C_q), 138.08 (C-10), 136.45 (C_q), 135.88 (C_q), 135.63 (C_q), 134.51 (C-15), 134.14 (C-8), 131.64 (C-4), 130.44 (C-16 or C-18), 130.12(C_q), 128.46 (C-12 or C-17), 128.04 (C-14), 127.12 (C-12 or C-17), 126.68 (C-13), 125.90 (C-16 or C-18), 122.04 (C-2), 35.04 (C-19), 31.43 (C-20). **IR (RT, ATR)** $\tilde{\nu}$ (cm^{-1}): 3049 (vw), 2957 (w), 2866 (w), 1587 (w), 1462 (w), 1366 (w), 1250 (w), 1188 (vw), 1094 (vw), 1049 (vw), 962 (vw), 891 (w), 866 (m), 820 (m), 766 (vs), 731 (w), 654 (w).**UV-vis** (CH_2Cl_2) λ_{max} (lg ε) 321 (4.32), 423 (4.52) nm. **Fluorescence** (CH_2Cl_2) λ_{em} (λ_{ex}): 542 (410) nm. **CV (**0.1 M TBAPF, DCM, RT): $E_{1/2}^{Ox}$ = 0.5 eV. **MALDI HRMS** (DCTB) (*m*/*z*): calcd. for $[M]^+$: 564.2817; found: 564.2814. **Elemental Analysis** [%] calculated for $C_{44}H_{36}\cdot 3H_2O$: C 85.40, H 6.84, found: C 85.80, H 6.66.

Compound 5

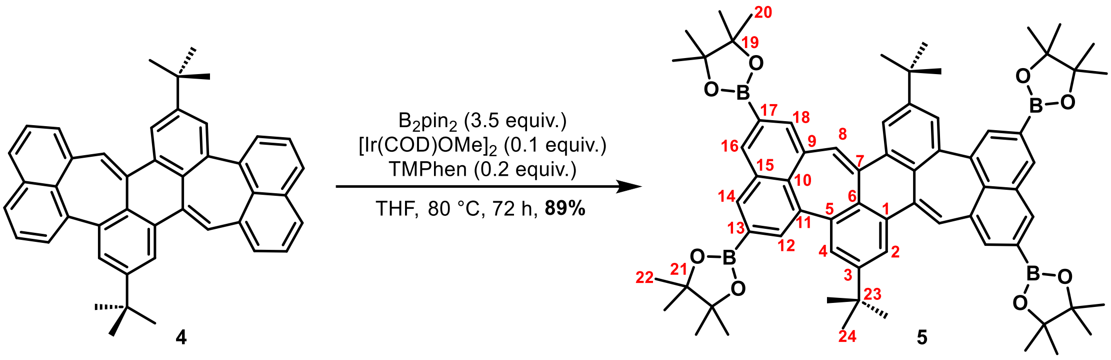

In a glovebox, $[Ir(COD)OMe]_2$ (47 mg, 71 μmol), 3,4,7,8-tetramethyl-1,10-phenanthroline (33.5 mg, 68 μmol) and B_2pin_2 (54 mg 0.2 mmol) were weighed in a 20 mL screw capped vial. THF (16 mL) was added and the mixture was stirred vigorously for 20 minutes. The catalyst solution was then added to a mixture of PAH **4 (**400 mg, 0.7 mmol) and B_2pin_2 (1.0 g, 4.0 mmol) in a screw-capped vial. The reaction mixture was stirred at 80 °C for 72 h. After cooling down to room temperature, the solvent was removed under reduced pressure using a rotary evaporator. The crude product was dissolved in DCM (5 mL) and added dropwise to MeOH (50 mL) with stirring to precipitate PAH **5** (670 mg, 89%) as a yellow solid. **m.p.** 375–378 °C (decomp.).**^{1}H NMR** (600 MHz, $CDCl_3$) *δ* 8.26 (s, 2H, H-14), 8.17 (s, 2H, H-16), 7.98 (s, 2H, H-12), 7.72 (s, 2H, H-18), 7.66 (d, *J* = 1.9 Hz, 2H, H-2), 7.15 (d, *J* = 1.9 Hz, 2H, H-4), 7.14 (s, 2H, H-8), 1.39 (s, 48H, $CH_{3,\ H20/21}$), 1.35 (s, 18H, $CH_{3,\ tBu}$).**^{13}C NMR** (151 MHz, $CDCl_3$) *δ* 152.08 (C-3), 140.97 (Cq), 139.07 (C-5), 138.59 (C-11), 136.99 (C-14), 136.50 (C_q), 135.96 (C-16), 135.92 (C-18), 134.83 (Cq), 134.01 (C-12), 133.84 (C-8), 133.33 (C_q), 131.52 (C-4), 130.16 (C_q), 127.20 (Bpin-C_q), 126.10 (Bpin-C_q), 122.06 (C-2), 84.14 (C-21/19), 84.05 (C-21/19), 35.01 (C-23), 31.46 (C-24), 25.07 (C-22/20), 25.04 (C-22/20). **IR (RT, ATR)** $\tilde{\nu}$ (cm^{-1}): 2974 (w), 2874 (vw), 1614 (w), 1591 (vw), 1468 (w), 1414 (s), 1381 (s), 1300 (s), 1246 (m), 1207 (w), 1142 (vs), 1115 (m), 1005 (vw), 968 (m), 903 (w), 856 (m), 833 (w), 797 (vw), 770 (vw), 689 (m), 658 (w). **UV-vis** (CH_2Cl_2) λ_{max} (lg ε) 323 (4.28), 431 (4.47) nm; **Fluorescence** (CH_2Cl_2) λ_{em} (λ_{ex}): 488 (420) nm. **CV (**0.1 M TBAPF, DCM, RT): $E_{1/2}^{Ox}$ = 0.4 eV. **MALDI HRMS** (DCTB) (*m*/*z*): calcd. for $[M]^+$: 1068.6225, found: 1068.6247. **Elemental Analysis** [%] calcd. for $C_{68}H_{80}B_4O_8{\cdot}3H_2O$: C 72.75, H 7.72, found: C 72.47, H 7.66.

Compound 6

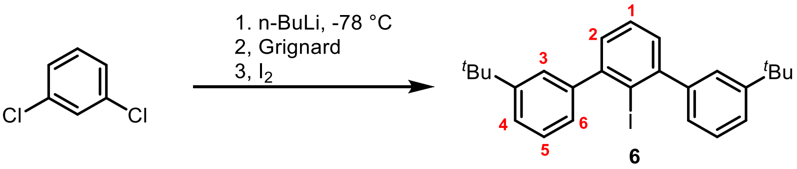

To a stirred solution of 1,3-dichlorobenzene (1.47 g, 10 mmol) in THF (25 mL) at –78 °C (EtOAc with liquid nitrogen), *n*-BuLi (4 mL, 2.5 M in hexane) was slowly added. The reaction mixture was stirred at –78 °C for 1.5 h, to give a white slurry. In another 100 mL three-necked flask flushed with argon, 1-bromo-3-tert-butylbenzene (6.39 g, 30 mmol), magnesium (860 mg, 36 mmol), a piece of iodine and THF (15 mL) were added. The flask was heated in an oil bath at 40–50 °C without stirring. After the evolution of first bubbles was observed, the stirring was started until the solution was clear. The freshly prepared Grignard reagent was transferred via cannula to the reaction mixture at –78 °C. The resulting mixture was allowed to warm to room temperature and stirred overnight. The reaction slurry was cooled in an ice bath, and a solution of I_2 (7.61 g, 30 mmol) in THF (15 mL) was added dropwise with vigorously stirring. The reaction mixture was then warmed to rt and stirred for another 1 h. The mixture was washed with an aqueous solution of sq. Na_2SO_3 (50 mL) and extracted with MTBE (3 x 30 mL). The combined organic layer was dried with $MgSO_4$ and concentrated under reduce pressure. The residue was purified by column chromatography (SiO_2, PE) to give the product as a colorless solid (1.7 g, 36%). R_f = 0.2 (PE). **m.p.** 94 °C. **^{1}H NMR** (600 MHz, $CDCl_3$) *δ* 7.44 – 7.41 (m, 4H, H-3/4), 7.40 (d, *J* = 7.7 Hz, 1H, H-1), 7.37 (t, *J* = 7.6 Hz, 2H, H-5), 7.28 (d, *J* = 7.5 Hz, 2H, H-2), 7.20 – 7.17 (m, 2H, H-6), 1.37 (s, 18H, H-*t*Bu). **^{13}C NMR** (151 MHz, $CDCl_3$) *δ* 150.65, 148.70, 145.37, 128.69 (C-2), 127.70 (C-5), 127.66 (C-1), 127.31 (C-3 or C-4), 126.46 (C-6), 124.40 (C-3 or C-4), 104.27, 34.96, 31.50. **IR (RT, ATR)** $\tilde{\nu}$ (cm^{-1}): 2958.75 (m), 2900.90 (w), 2865.22 (w), 1601.11 (w),

1570.50 (w), 1474.79 (w), 1452.14 (w),1410.19 (w), 1386.33 (w), 1360.53 (w), 1255.67 (w), 1180.22 (w), 1091.75 (w), 1004.73 (w), 998.70 (w), 902.76 (w), 896.25 (w), 807.06 (w), 796.46 (s), 784.89 (s), 729.68 (m), 703.65 (s) ,697.38 (vs), 668.70 (w), 667.01 (w), 636.64 (w), 620.00 (m), 598.55 (w). **EI HRMS** (DCTB) (*m*/*z*): calcd. for $[M]^+$: 468.1314, found: 468.1273. **Elemental Analysis** [%] calculated for $C_{26}H_{29}I$: C 66.67, H 6.24, found: C 66.64 H 5.91.

Compound 7

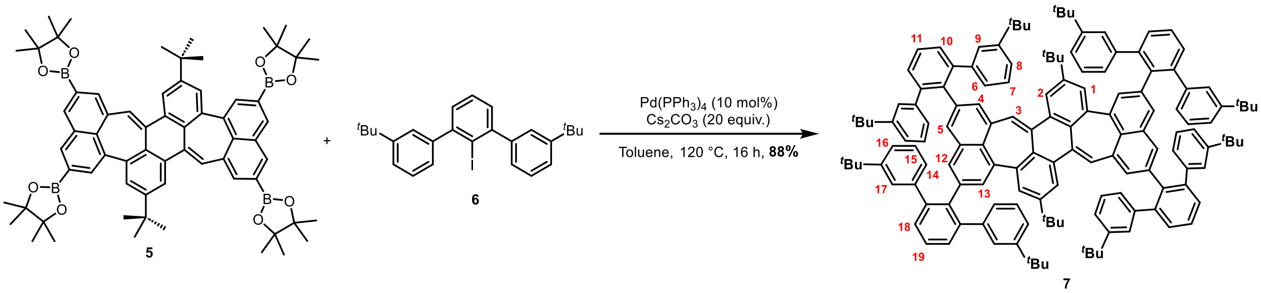

In a 25 mL vial purged with argon, **5** (100 mg, 94.0 μmol), iodoterphenyl **6** (438 mg, 0.84 mmol) and Cs_2CO_3 (609 mg, 1.9 mmol) were mixed in degassed toluene (4 mL). Pd $(PPh_3)_4$ (10.8 mg, 14.0 μmol) was added under argon atmosphere and the mixture was further bubbled with argon for 5 minutes. The vial was sealed and the reaction mixture was refluxed overnight. After cooling to room temperature, the solvent was removed under reduced pressure using a rotary evaporator. The residue was dissolved in CH_2Cl_2 (10 mL) and washed with brine (10 mL). The organic layer was dried with anhydrous $MgSO_4$ and concentrated under reduced pressure. The crude mixture was further purified by column chromatography (SiO_2, PE/EA, 20/1) to give **7** (158 mg, 88%) as a yellow solid. ***R*$_f$** = 0.45 (PE/EA 10:1). **m.p.** 175°C (decomp.).**^{1}H NMR** (400 MHz, THF-*d*$_8$) ***δ*** 7.49 – 7.36 (m, 10H, H-6/14), 7.36 – 7.33 (m, 4H, H-7), 7.33 – 7.31 (m, 2H, H-2), 7.20 – 7.09 (m, 20H), 6.98 (t, *J* = 7.7 Hz, 8H, H-10/18), 6.83 – 6.81 (m, 2H, H-8), 6.79–6.72 (m, 8H, H-11/19/9/17), 6.46 (s, 2H, H-3), 6.45 – 6.43 (m, 2H, H-1), 1.23 (s, 18H, H-*t*Bu), 1.09 (s, 36H, H-*t*Bu), 0.95 (s, 36H, H-*t*Bu). **^{13}C NMR** (101 MHz, THF-*D*$_8$) ***δ*** 152.01, 151.01, 150.65, 143.60, 142.48, 142.30, 139.80, 139.36, 139.27, 138.84, 138.64, 137.85, 137.24, 136.41, 136.12, 134.99, 134.66, 134.33, 134.05, 132.01, 131.44, 131.23, 131.01, 130.50, 130.35, 128.86, 128.67, 128.26, 128.23, 128.07, 127.63, 127.17, 123.79, 121.66, 35.44, 35.12, 35.07, 32.81, 32.02, 31.77, 31.75. **IR (RT, ATR)** $\tilde{\nu}$ (cm^{-1}): 2961(w), 1601 (w), 1410 (w), 1362 (m), 1340 (w), 1315 (w), 1288 (m), 1256 (m), 1204 (w), 1177 (w), 1032 (w), 897 (m), 879 (w), 866 (w), 789 (vs), 760 (m), 706 (vs), 667 (w), 654 (w), 621(w). **UV-vis** (CH_2Cl_2) λ_{max} (lg ε) 427 (4.26) nm. **Fluorescence** (CH_2Cl_2) λ_{em} (λ_{ex}): 535 (360) nm. **CV (**0.1 M TBAPF, DCM, RT): $E_{1/2}^{Ox}$ = 0.4 eV. **MALDI HRMS** (DCTB) (*m*/*z*): calcd. for $[M]^+$: 1926.1615, found: 1926.1610. **Elemental Analysis** [%] calculated for $C_{148}H_{148}$: C 92.26, H 7.74, found: C 91.69, H 7.64.

Compound 8

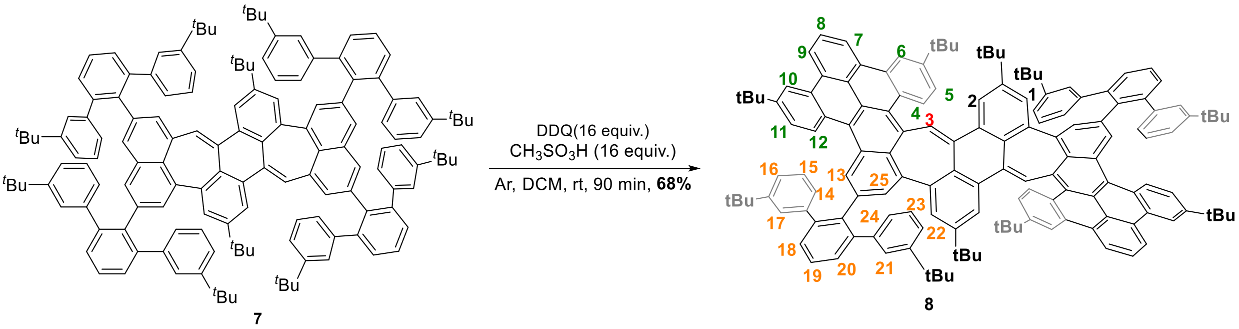

In a 50 mL round-bottom flask purged with argon, **7** (36.6 mg, 19 μmol) was dissolved in dry CH_2Cl_2 (20 mL). 2,3-Dichloro-5,6-dicyanobenzoquinone (42.4 mg, 187 μmol) was added in one portion. Then methanesulfonic acid (0.35 mL) was added. The mixture was stirred at room temperature for 90 min. The reaction was quenched by pouring into saturated aqueous $NaHCO_3$ solution (50 mL) and the mixture was stirred vigorously for 15 minutes. After phase separation, the organic layer was washed with water (50 mL), brine (50 mL) and dried over anhydrous $MgSO_4$. The solvent was removed under reduced pressure and the crude product was purified by column chromatography (SiO_2, hexane/dichloromethane 5:1) to give PAH **8** (24.5 mg, 68%) as purple solid. ***R*$_f$** = 0.35 (PE/DCM 3:1). **m.p.** 330 °C (decomp.).**^{1}H NMR** (600 MHz,

THF-d_8) δ 9.07 (d, J = 8.7 Hz, 2H, H-4), 8.95 (d, J = 8.0 Hz, 2H, H-9), 8.87 (d, J = 7.8 Hz, 2H, H-7), 8.80 (d, J = 2.1 Hz, 2H, H-10), 8.75 (s, 2H, H-6), 8.52 (s, 2H, H-13), 8.00 (t, J = 7.8 Hz, 2H, H-8), 7.74 (d, J = 8.7 Hz, 2H, H-5), 7.67 – 7.62 (m, 6H, H-3/2), 7.60 – 7.52 (m, 8H), 7.45 (d, J = 8.4 Hz, 2H, H-11), 7.39 (s, 2H, H-25), 7.37 (s, 2H), 7.29 (d, J = 7.8 Hz, 2H), 7.24 – 7.18 (m, 2H), 7.16 (d, J = 8.5 Hz, 2H, H-12), 7.11 – 6.98 (m, 4H), 6.92 (d, J = 7.7 Hz, 2H), 6.83 (s, 2H, H-1), 1.58 (s, 18H, H-*t*Bu), 1.44 (s, 18H, H-*t*Bu), 1.25 (s, 18H, H-*t*Bu), 0.79 (s, 18H, H-*t*Bu), 0.74 (s, 18H, H-*t*Bu). **^{13}C NMR** (151 MHz, THF-d_8) δ 152.76 (C_q), 151.93 (C_q), 151.15 (C_q), 151.07 (C_q), 150.50 (C_q), 145.05 (C_q), 142.73 (C_q), 142.62 (C_q), 142.40 (C_q), 140.82 (C_q), 140.48 (C_q), 139.98 (C_q), 139.21 (C_q), 138.64 (C_q), 137.70, 136.47 (C_q), 134.05 (C_q), 133.03 (C_q), 133.00 (C-25), 132.23 (C_q), 131.81 (C_q), 131.52 (C_q), 131.36 (C_q), 131.25 (C-4), 131.11 (C-13), 131.03 (C_q), 130.64, 130.56 (C-1), 130.41, 130.15 (C-12), 129.98, 129.78, 129.66 (C_q), 129.44, 128.89, 128.76 (C_q), 128.56, 127.74 (C-8), 127.70, 127.58, 125.87 (C_q), 125.75 (C-5), 125.60 (C-11), 125.19 (C_q), 124.95 (C_q), 124.21, 124.09, 122.58 (C-9), 122.28 (C-7), 122.12 (C-3), 121.20 (C-6), 120.46 (C-10), 67.57, 35.98 (C_q), 35.96 (C_q), 35.81 (C_q), 35.20 (C_q), 35.16 (C_q), 32.11 (C_{Me}), 32.05 (C_{Me}), 31.86 (C_{Me}), 31.55 (C_{Me}), 31.52 (C_{Me}). **IR (RT, ATR)** $\tilde{\nu}$ (cm^{-1}): 2964 (m), 1406 (m), 1362 (m), 1285 (m), 1256 (s), 1067 (m), 1043 (m), 789 (vs), 762 (vs), 706 (s), 627 (m). **UV-vis** (CH_2Cl_2) λ_{max} (lg ε) 541 (4.70) nm. **Fluorescence** (CH_2Cl_2) λ_{em} (λ_{ex}): 605 (541) nm. **CV (**0.1 M TBAPF, DCM, RT): $E_{1/2}^{Ox}$ = 0.3 eV; $E_{1/2}^{Red}$ = −1.8 eV. **MALDI HRMS** (DCTB) (*m/z*): [calcd. for M]$^+$: 1918.0989, found: 1918.0966. **Elemental Analysis** [%] calcd. for $C_{148}H_{140}$·$1.5CH_2Cl_2$: C 87.76, H 7.04, found: C 87.83, H 7.29.

Compound 9

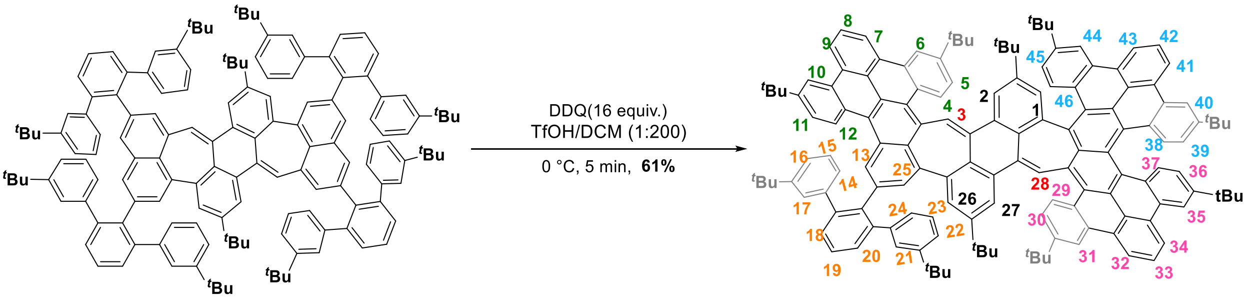

7 9

In a 50 mL round-bottom flask purged with argon, **7** (27.0 mg, 14 µmol) was dissolved in dry dichloromethane (20 mL), and the mixture was cooled to 0 °C in an ice bath. 2,3-Dichloro-5,6-dicyano-1,4-benzoquinone (DDQ, 95.3 mg, 0.42 mmol) was added in one portion under argon. After dropwise addition of trifluoromethanesulfonic acid (0.1 mL), the reaction mixture was stirred at 0 °C for an additional 5 minutes. The reaction was quenched by pouring into saturated aqueous $NaHCO_3$ (50 mL) and stirred vigorously for 15 minutes. After phase separation, the organic layer was washed with brine (50 mL) and dried over anhydrous $MgSO_4$. After removal of the solvent by rotary evaporation, the crude product was purified by silica gel column chromatography (petroleum ether/dichloromethane 10:1) to give PAH **9** (16.4 mg, 61%) as dark green solid. ***R*$_f$** = 0.40 (PE/DCM 3:1). **m.p.** > 400 °C. **^{1}H NMR** (700 MHz, THF-d_8) δ 9.24 (d, J = 8.6 Hz, 1H, H-29), 9.19 – 9.10 (m, 4H, H-4/32/41/43), 9.03 (d, J = 7.8 Hz, 1H, H-34), 9.00 – 8.96 (m, 2H, H-7/31), 8.91 (d, J = 7.7 Hz, 1H, H-9), 8.84 (s, 1H, H-10), 8.81 – 8.76 (m, 3H, H-6/40/44), 8.75 (s, 1H, H-35), 8.65 (s, 1H, H-13), 8.35 (s, 1H, H-28), 8.16 (t, J = 7.7 Hz, 1H, H-42), 8.13 (t, J = 7.7 Hz, 1H, H-33), 8.01 (t, J = 7.7 Hz, 1H, H-8), 7.88 (d, J = 8.6 Hz, 1H, H-30), 7.84 (s, 1H, H-27), 7.81 (d, J = 8.6 Hz, 1H, H-37), 7.78 (d, J = 8.6, 1.9 Hz, 1H, H-5), 7.73 (s, 1H, H-3), 7.70 – 7.60 (m, 6H, H-38/46), 7.58 – 7.49 (m, 5H, H-18 or 20/11/25), 7.44 (t, J = 7.6 Hz, 1H, H-19), 7.24 (d, J = 8.3 Hz, 1H, H-12), 7.23 – 7.19 (m, 2H, H-18 or 20), 7.18 – 7.13 (m, 2H, H-36), 7.09 (s, 1H, H-17 or H-21), 6.90 – 6.85 (m, 3H, H-45/39), 6.72 (s, 1H, H-26), 1.60 (s, 9H), 1.51 (s, 9H), 1.45 (s, 9H), 1.40 (s, 10H), 1.35 (d, J = 4.6 Hz, 19H, H-*t*Bu), 1.19 (s, 9H, H-*t*Bu), 0.92 (s, 9H, H-*t*Bu), 0.78 (s, 9H, H-*t*Bu), 0.70 (s, 9H, H-*t*Bu). **^{13}C NMR** (176 MHz, THF-d_8) δ 153.23 (C_q), 152.84 (C_q), 152.00 (C_q), 151.75 (C_q), 150.97 (C_q), 150.57 (C_q), 150.46 (C_q), 150.36 (C_q), 144.54 (C_q), 142.85 (C_q), 142.83 (C_q), 142.53 (C_q), 142.20 (C_q), 140.64 (C_q), 140.39 (C_q), 139.46 (C_q), 139.05 (C-28), 138.99 (C-3), 138.59 (C_q), 137.19, 136.79 (C_q), 136.60 (C_q), 136.06 (C_q), 135.91 (C_q), 135.39 (C_q), 134.69 (C_q), 133.71 (C_q), 133.29 (C_q), 133.19 (C-25), 132.87 (C_q), 132.12 (C-37), 132.07 (C_q), 131.98 (C_q), 131.90 (C_q), 131.87 (C_q), 131.86 (C_q), 131.72 (C_q), 131.69 (C_q), 131.66 (C_q), 131.64 (C-29), 131.38 (C_q), 131.31 (C_q), 131.29 (C_q), 131.16 (C-13), 131.13 (C-26), 131.07 (C_q), 130.87 (C-32), 130.54 (C), 130.50 (C_q),

130.45 (C_q), 130.31 (C_q), 130.25 (C_q), 130.21 (C), 130.14 (C), 129.90 (C_q), 129.85 (C), 129.66 (C-17 or 21), 129.60 (C-19), 129.40 (C_q), 128.95 (C_q), 128.89 (C_q), 128.87 (C), 128.77 (C), 128.53 (C_q), 128.49 (C), 128.46 (C_q), 128.42, 127.67 (Cq), 127.63 (C_q), 127.58 (C-8), 127.54 (C-42 or 43), 127.47 (C-42 or 43), 127.43 (C), 126.01 (C-30), 125.86 (C_q), 125.79 (C-5), 125.66 (C_q), 125.22 (C), 125.13 (C-36 or), 124.89 (C_q), 124.72 (C_q), 124.48 (C-45 or 39), 124.44 (C-45 or 39), 124.25 (C-36), 124.00 (C_q), 123.97 (C-12), 123.92 (C_q), 122.91 (C-41 or 43 or 32), 122.71 (C), 122.42 (C-7), 122.40 (C), 122.13 (C-34), 122.11 (C-9), 121.99 (C-27), 121.75 (C), 121.41 (Cq), 120.99 (C-31), 120.84 (C-6), 120.29 (C-10), 119.54 (C-35), 119.37 (C-44/40), 35.95 (C_q), 35.81 (Cq), 35.70 (C_q), 35.68 (C_q), 35.61 (C_q), 35.58 (C_q), 35.25 (C_q), 35.11 (C_q), 34.92 (C_q), 31.88 (C_{Me}), 31.76 (C_{Me}), 31.69 (C_{Me}), 31.65 (C_{Me}), 31.20 (C_{Me}), 31.13 (C_{Me}). **IR (RT, ATR)** $\tilde{\nu}$ (cm^{-1}): 2957 (vs), 2926 (s), 2862 (m), 1069 (m), 1576 (m), 1474 (s), 1460 (s), 1364 (s), 1259 (m), 841 (s), 793 (s), 752 (m), 708 (s), 627 (m). **UV-vis (CH_2Cl_2)** λ_{max} (lg ε) 308 (5.12), 589 (4.67) nm. **Fluorescence** (DCM) λ_{em} (λ_{ex}): 546 (450) nm. **CV (**0.1 M TBAPF, DCM, RT): $E_{1/2}^{Ox}$ = 0.1 eV; $E_{1/2}^{Red}$ = −1.9 eV. **MALDI HRMS** (DCTB) (*m/z*): calcd. for $[M]^+$: 1914.0676; found: 1914.0710. **Elemental Analysis** [%] calculated for $C_{148}H_{136}$: C 92.84, H 7.16; found: C 91.92, H 7.44. **Absorption Dissymmetry Factor (g_{abs}):** 6.19 × 10^{-4} at 454 nm.

Chiral resolution: The enantiomers of PAH **9** were separated by chiral HPLC (column: CHIRALPAK IC, particle size 5 µm, dimension 4.6 mm I.D. x 250 mL; eluent: *n*-heptane/DCM (95:5); flow rate: 10 mL/min; retention time: 12.0 and 14.3 min.). The ratio of enantiomers was: 48.7 : 51.3. The assignment of the absolute configuration was accomplished by comparing experimental and simulated spectra.

Compound 10

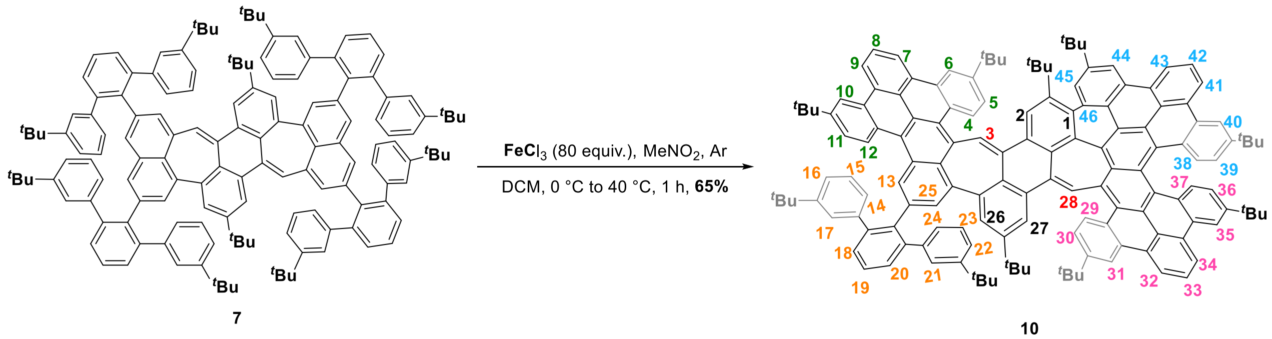

In a 50 mL round-bottom flask purged with argon, **7** (26.0 mg, 13 µmol) was dissolved in dry dichloromethane (12 mL), and the reaction mixture was cooled to 0 °C (ice bath). A solution of $FeCl_3$ (175 mg, 1.08 mmol)) in nitromethane (0.4 mL) was added dropwise. The reaction mixture was then heated to reflux for 1 hour. After cooling to room temperature, the reaction was quenched by adding methanol (5 mL). The resulting mixture was washed with water (3 × 20 mL). The separated organic layer was dried over $MgSO_4$. After removal of the solvent under reduced pressure using a rotary evaporator, the crude product was purified by silica gel column chromatography (petroleum ether/dichloromethane 5:1) to give PAH **10** (16.8 mg, 65%) as a dark purple solid. R_f = 0.40 (PE/DCM 3:1). **m.p.** > 400 °C. **^{1}H NMR** (600 MHz, THF-d_8) δ 9.46 (d, J = 8.7 Hz, 1H, H-29), 9.23 (d, J = 8.8 Hz, 1H, H-4), 9.17 (d, J = 7.8 Hz, 1H, H-43), 9.10 (d, J = 8.0 Hz, 1H, H-41), 9.04 (s, 1H, H-44), 9.02 (d, J = 7.9 Hz, 1H, H-34), 8.98 (d, J = 7.8 Hz, 1H, H-32), 8.95 (d, J = 8.2 Hz, 1H, H-9), 8.85 (d, J = 7.7 Hz, 1H, H-7), 8.83 (s 1H, H-31), 8.80 (s, 1H, H-10), 8.75 (d, J = 2.2 Hz, 1H, H-40), 8.70 (s, 1H, H-6), 8.64 (d, J = 2.2 Hz, 1H, H-35), 8.40 (d, J = 2.1 Hz, 2H, H-12/2), 8.23 (s, 1H, H-45), 8.14 (t, J = 7.8 Hz, 1H, H-42), 8.10 (t, J = 7.8 Hz, 1H, H-33), 8.00 (t, J = 7.8 Hz, 1H, H-8), 7.92 (s, 1H, H-3), 7.84 – 7.79 (m, 2H, H-30/28), 7.75 (s, 1H), 7.66 – 7.62 (m, 3H), 7.60 (s, 1H), 7.57 (d, J = 8.8 Hz, 1H, H-5), 7.51 (t, J = 7.6 Hz, 1H), 7.48 (d, J = 8.6 Hz, 1H, H-38), 7.45 (d, J = 8.4 Hz, 1H, H-11), 7.43 – 7.38 (m, 3H), 7.29 (d, J = 8.3 Hz, 1H, H-12), 7.25 (s, 1H, H-25), 7.08 (s, 1H), 7.01 – 6.96 (m, 2H, H-26), 6.92 (d, J = 8.7 Hz, 1H, H-39), 6.82 (d, J = 8.6 Hz, 1H, H-36), 6.74 (d, J = 7.7 Hz, 1H), 6.49 (s, 1H), 1.63 (s, 9H, H_{tBu}), 1.60 (s, 9H, H_{tBu}), 1.53 (s, 9H, H_{tBu}), 1.41 (s, 9H, H_{tBu}), 1.37 (s, 9H, H_{tBu}), 1.31 (d, J = 6.3 Hz, 18H, H_{tBu}), 1.19 (s, 9H, H_{tBu}), 1.01 (s, 9H, H_{tBu}), 0.51 (s, 9H, H_{tBu}). **^{13}C NMR** (151 MHz, THF-d_8) δ 152.23 (C_q), 151.17 (C_q), 151.02 (C_q), 150.41 (C_q), 150.24 (C_q), 149.66 (C_q), 149.47 (C_q), 149.37 (C_q), 146.96 (C_q), 146.37 (C_q), 144.70 (C_q), 141.77 (C_q), 141.66 (C_q), 141.48 (C_q), 140.93 (C_q), 139.74 (C_q), 139.50 (C_q), 137.91 (C_q), 137.51 (C_q), 137.44 (C_q), 135.50 (C-3), 135.40 (C-28), 133.37

(C_q), 132.40 (C_q), 132.24 (C_q), 132.00 (C-25), 131.64 (C_q), 131.61 (C_q), 131.52 (C_q), 131.50 (C_q), 131.42 (C_q), 131.35 (C_q), 131.30 (C_q), 130.88 (C_q), 130.81 (C_q), 130.75 (C_q), 130.73 (C-4), 130.36 (C-29), 130.29 (C_q), 130.21 (C_q), 130.17 (C-2 or 13), 129.70 (C_q), 129.67, 129.53, 129.48 (C_q), 129.42 (C_q), 129.33 (C-12), 129.18, 129.14, 129.09 (C_q), 128.89, 128.80, 128.61, 128.08 (C-25), 127.98 (C-45), 127.95 (C_q), 127.80, 127.78 (C_q), 127.68 (C_q), 127.47, 127.39 (C_q), 127.12 (C-42), 126.93 (C-33), 126.90 (C-8), 126.30, 125.60 (C_q), 125.06 (C_q), 125.01 (C-11), 124.92 (C_q), 124.73 (C_q), 124.69 (C_q), 124.67 (C_q), 124.58 (C-30), 124.50 (C-5), 124.29 (C-2 or 13), 124.04 (C_q), 124.01 (C-39), 123.78 (C-36), 123.62, 123.46 (C_q), 123.09, 122.29 (C_q), 122.26 (C_q), 122.18 (C-41), 122.06 (C-34), 122.00 (C-43), 121.79 (C-9), 121.45 (C-7), 121.27 (C-32), 120.89 (C-31), 120.70 (C_q), 120.67, 120.32 (C-6), 119.64 (C-10), 118.81 (C-35), 118.60 (C-40), 118.45 (C-44), 118.33 (C_q), 38.52 (C_q), 35.68 (C_q), 35.14 (C_q), 35.12 (C_q), 35.04 (C_q), 34.89 (C_q), 34.84 (C_q), 34.82 (C_q), 34.54 (C_q), 34.05 (C_q), 33.70 (C_{Me}), 31.46 (C_{Me}), 31.20 (C_{Me}), 31.01 (C_{Me}), 30.96 (C_{Me}), 30.91 (C_{Me}), 30.89 (C_{Me}), 30.59 (C_{Me}). **IR (RT, ATR)** $\tilde{\nu}$ (cm^{-1}): 2957 (m), 1464 (m), 1410 (m), 1367 (m), 1331 (m), 1285 (m), 1258 (s), 1236 (s), 1196 (m), 1159 (m), 1123 (m), 1103 (m), 1032 (s), 991 (m), 970 (m), 941 (m), 926 (s), 906 (s), 878 (s), 841 (s), 806 (s), 789 (vs), 762 (vs), 698 (s), 671 (m), 644 (m), 629 (m). **UV-vis (CH_2Cl_2)** λ_{max} (lg ε)) 363 (4.93), 511 (4.64) nm. **Fluorescence** (DCM) λ_{em} (λ_{ex}): 611 (511) nm. **CV (**0.1 M TBAPF, DCM, RT): $E_{1/2}^{Ox}$ = 0.2 eV; $E_{1/2}^{Red}$ = −2.1 eV. **MALDI HRMS** (DCTB) (*m/z*): calcd. for $[M]^+$: 1912.0519, found: 1912.0526. **Elemental Analysis** [%] calculated for $C_{148}H_{134}$: C 92.94, H 7.06; found: C 92.32, H 7.17. **Absorption Dissymmetry Factor (g_{abs}):** 6.61 × 10^{-3} at 437 nm.

Chiral resolution: the enantiomers of compound **10** were separated by chiral HPLC (column: CHIRALPAK IA, particle size 5 µm, dimension 4.6 mm I.D. x 250 mL; eluent: *n*-heptane/DCM/Et_3N (100:2:0.3); flow rate: 10 mL/min; retention time: 18.0 and 23.8 min.). The ratio of enantiomers was: 51.4:48.6. The assignment of the absolute configuration was accomplished by comparing experimental and simulated spectra.

Compound 11

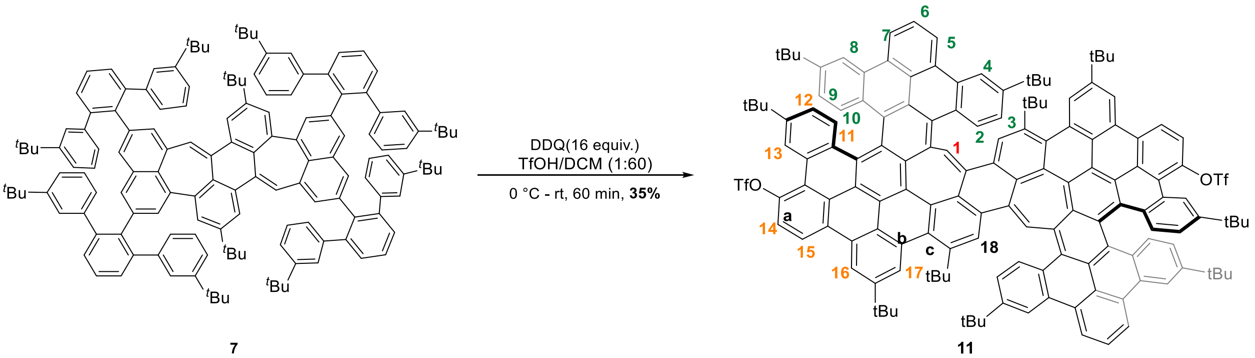

7 11

In a 50 mL round-bottom flask purged with argon, **7** (20.0 mg, 0.011 mmol) was dissolved in dry dichloromethane (12 mL) and the reaction mixture was cooled to 0 °C (ice bath). 2,3-Dichloro-5,6-dicyano-1,4-benzoquinone (DDQ, 37.7 mg, 0.18 mmol) was added in one portion under argon. Trifluoromethanesulfonic acid (0.12 mL) was added dropwise. The mixture was then allowed to warm to room temperature and stirred for another hour. The reaction was quenched by pouring into saturated aq. $NaHCO_3$ (30 mL) and stirred vigorously for 15 minutes. After phase separation, the organic layer was washed with brine (20 mL) and dried over anhydrous $MgSO_4$. After removal of the solvent by rotary evaporation, the crude product was purified by silica gel column chromatography (petroleum ether/dichloromethane 10:1) to give PAH **11** (8.1 mg, 35%) as a brown solid. $\boldsymbol{R_f}$ = 0.45 (PE/DCM 3:1). **m.p.** > 400 °C. **^{1}H NMR** (600 MHz, $CDCl_3$) δ 9.62 (d, *J* = 8.7 Hz, 2H, H-2), 9.00 (d, *J* = 8.1 Hz, 2H, H-7), 8.94 (dd, *J* = 8.6, 7.3 Hz, 4H, H-15/5), 8.88 (d, *J* = 2.1 Hz, 2H, H-13), 8.75 (s, 4H, H-4/16), 8.57 (s, 2H, H-8), 8.29 (s, 2H, H-18), 8.17 (t, *J* = 7.8 Hz, 2H, H-6), 8.01 (d, *J* = 8.3 Hz, 2H, H-14), 7.97 (s, 2H, H-1), 7.95 (s, 2H, H-17), 7.53 – 7.52 (m, 2H, H-3), 7.52 – 7.50 (m, 2H, H-10), 7.34 (d, *J* = 8.7 Hz, 2H, H-11), 6.98 – 6.94 (m, 4H, H-12/9), 1.47 (s, 18H), 1.42 (s, 18H, H_{tBu}), 1.34 (s, 18H, H_{tBu}), 1.32 (s, 18H, H_{tBu}), 1.26 (s, 18H, H_{tBu}). **^{13}C NMR** (151 MHz, $CDCl_3$) δ 151.05 (C_q), 149.90 (C_q), 149.11 (C_q), 147.07 (C_q), 147.00 (C_q), 145.59 (C_q), 141.21 (C_q), 136.89 (C_q), 134.86 (C-1), 132.71 (C_q), 132.13 (C_q), 131.33 (C_q), 131.28 (C_q), 131.14 (C_q), 131.05 (C_q), 130.97 (C_q), 130.82 (C_q), 130.21 (C-2), 129.96 (C_q), 129.90 (C_q), 129.80 (C_q), 129.50 (C-10), 129.36 (C_q), 129.35 (C_q), 128.83 (C_q), 128.66 (C-17), 128.42 (C-11), 128.32 (C_q), 128.06 (C_q), 127.88 (C_q), 127.39 (C_q), 127.15 (C-6), 126.58 (C_q), 125.78 (C-12), 125.14 (C-3), 125.05

(C_q), 124.97 (C_q), 124.93 (C_q), 124.76 (C-9), 123.81 (C-18), 123.64 (C_q), 123.20 (C-13), 122.09 (C-15), 121.96 (C-7), 121.90 (C_q), 121.83 (C_q), 121.09 (C-5), 120.99 (C-14), 120.61 (C-4), 120.17 (C_q), 119.76 (C_q), 118.47 (C-16), 118.41 (C-8), 118.16 (C_q), 38.59 (C_q), 35.60 (C_q), 35.18 (C_q), 35.12 (C_q), 35.00 (C_q), 34.22 (C_{Me}), 31.75 (C_{Me}), 31.42 (C_{Me}), 31.27 (C_{Me}), 31.16 (C_{Me}). **^{19}F NMR** (471 MHz, $CDCl_3$) δ -72.34. **IR (RT, ATR)** $\tilde{\nu}$ (cm^{-1}): 2962 (m), 2905 (m), 1423 (w), 1408 (w), 1366 (w), 1261 (s), 1207 (m), 1138 (m), 1095 (s), 1078 (s), 1022 (s), 866 (m), 839 (w), 800 (vs), 764 (w), 744 (m), 627 (w). **UV-vis (CH_2Cl_2)** λ_{max} (lg ε)) 319 (6.28), 373 (6.23), 415 (6.26), 511 (5.75), 583 (5.76) nm. **Fluorescence** (DCM) λ_{em} (λ_{ex}): 631 (490) nm. **CV (**0.1 M TBAPF, DCM, RT): $E_{1/2}^{Ox}$ = 0.1 eV; $E_{1/2}^{Red}$ = −2.0 eV. **MALDI HRMS** (DCTB) (*m*/*z*): calcd. for $[M]^+$: 2201.8934, found: 2201.8935. **Elemental Analysis** [%] calculated for $C_{150}H_{126}F_6O_6S_2{\cdot}2H_2O$: C 80.47, H 5.85; found: C 80.63, H 6.05. **Absorption Dissymmetry Factor (g_{abs}):** 4.85×10^{-3} at 433 nm.

The enantiomers of compound **11** were separated by chiral HPLC (column: CHIRALPAK IA, particle size 5 µm, dimension 4.6 mm I.D. x 250 mL; eluent: *n*-heptane/Et_3N (1000:3); flow rate: 10 mL/min; retention time: 15.5 and 21.0 min.). The ratio of enantiomers was: 50.7 : 49.3. The assignment of the absolute configuration was accomplished by comparing experimental and simulated spectra.

3. NMR Spectra

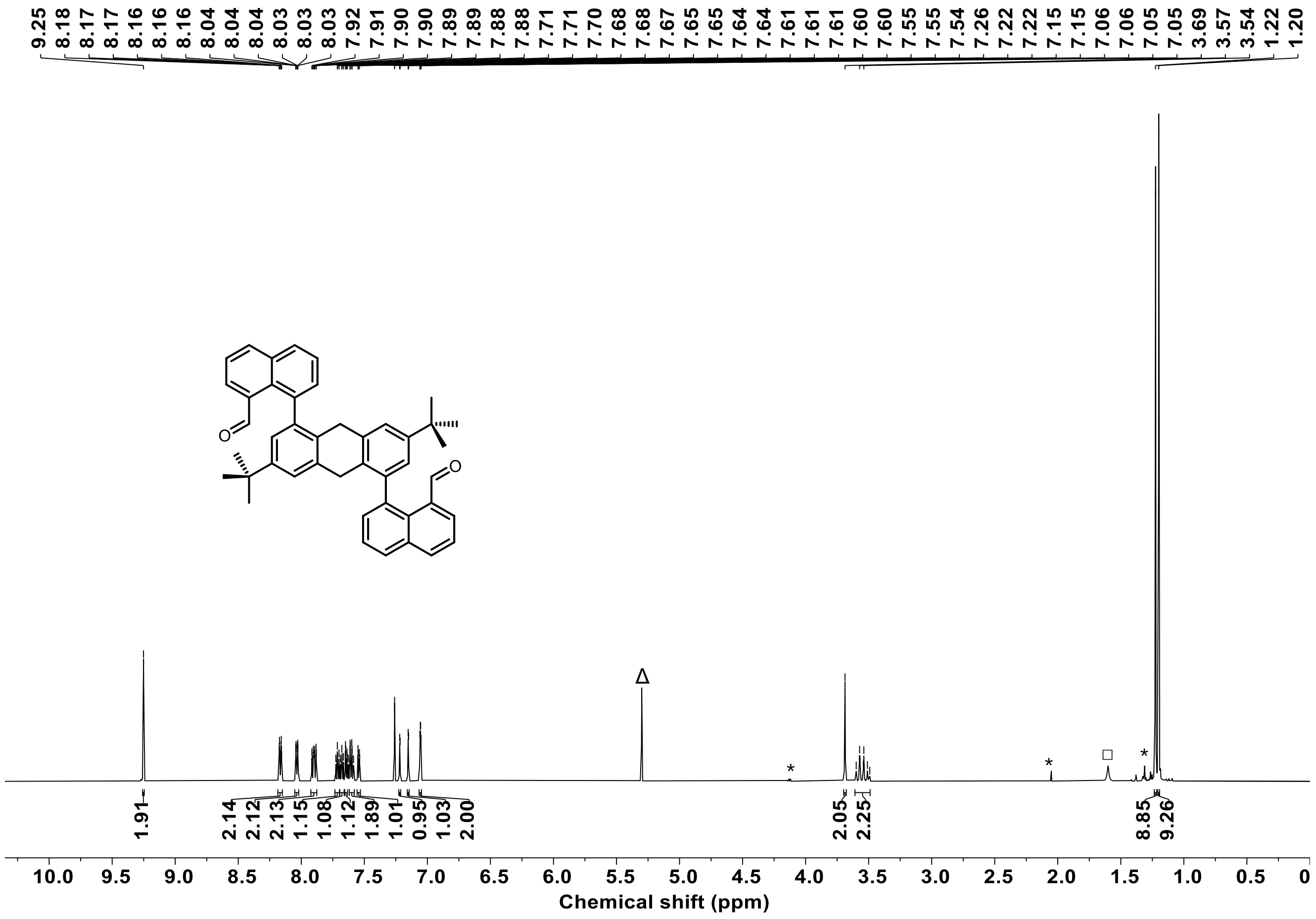

Figure S1. ^{1}H NMR spectrum (600 MHz, $CDCl_3$, 298 K) of compound **3**. Δ: DCM, *: Ethyl acetate, □: H_2O.

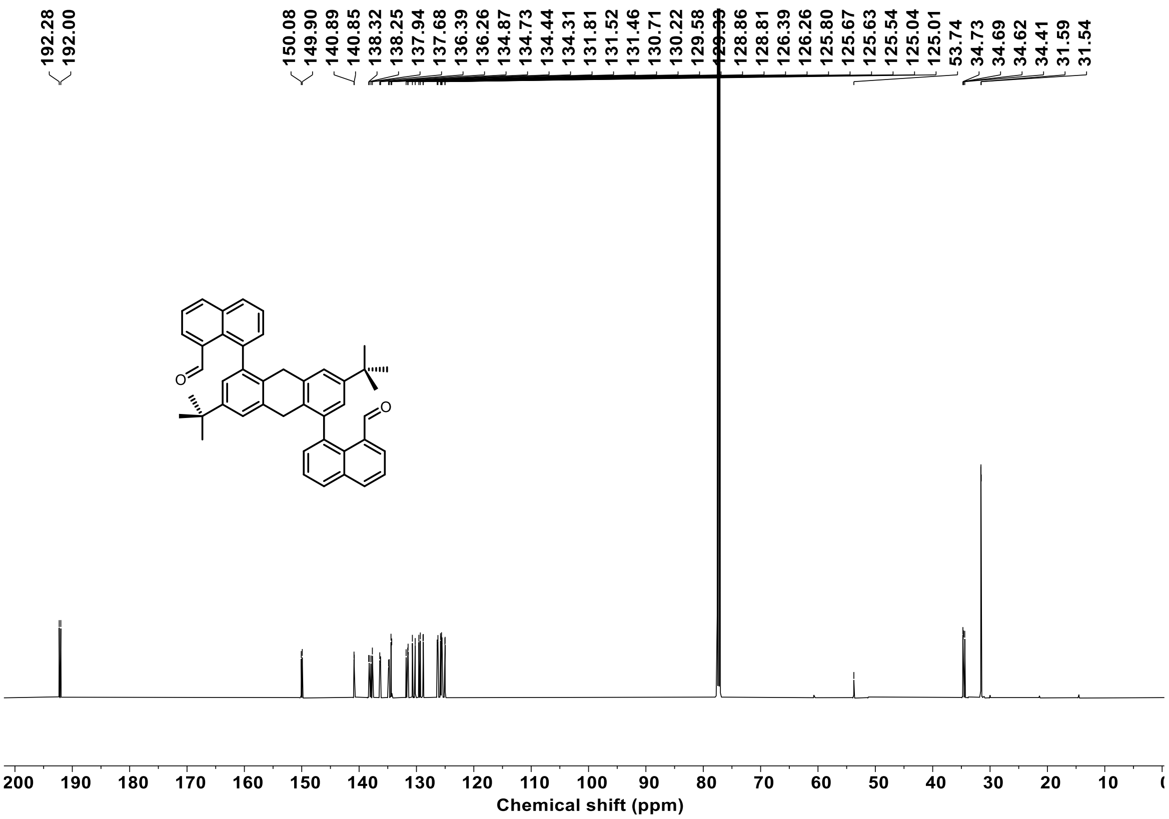

Figure S2. ^{13}C NMR spectrum (151 MHz, $CDCl_3$, 298 K) of compound **3**.

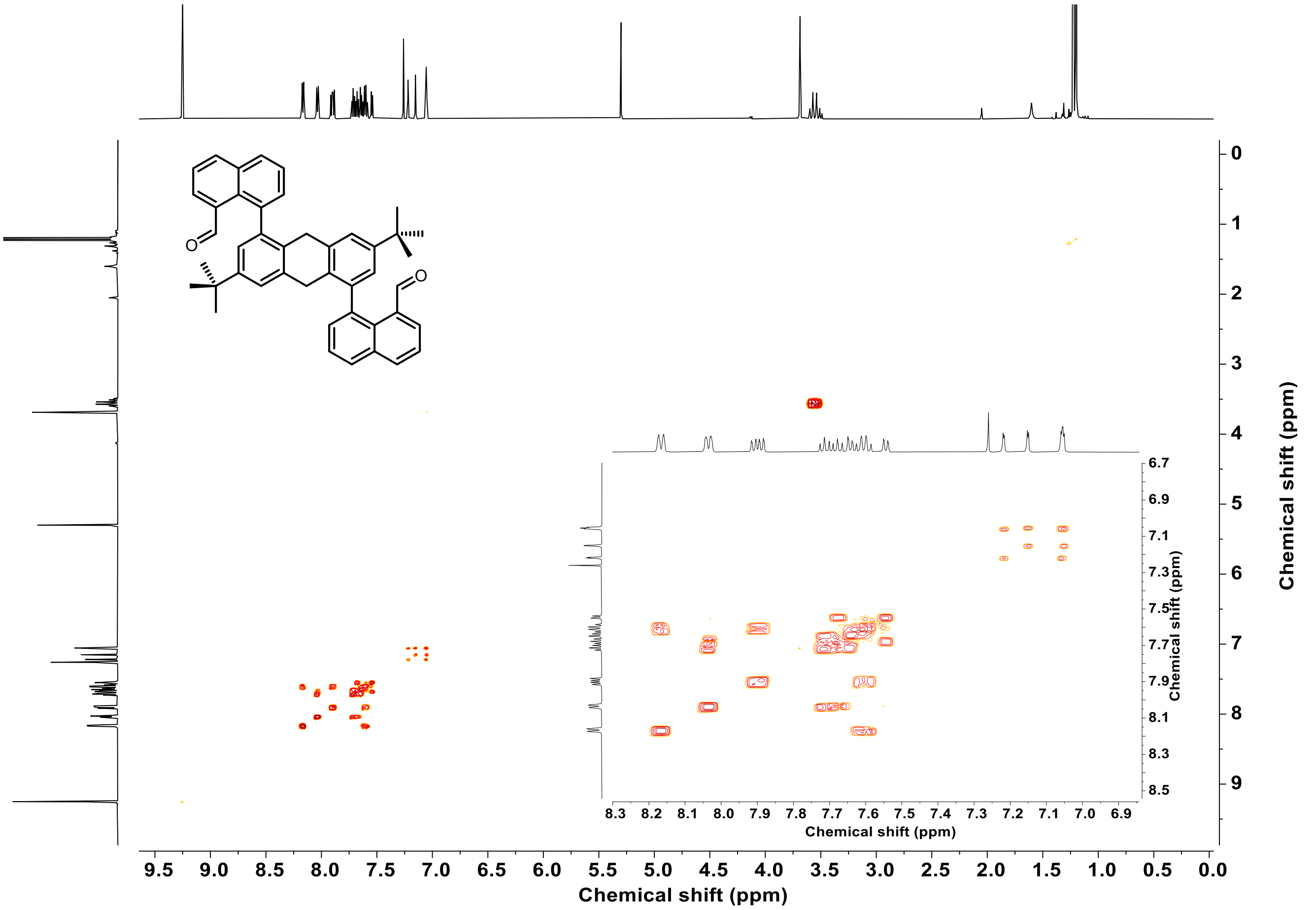

Figure S3. ^{1}H-^{1}H COSY NMR spectrum (600/600 MHz, $CDCl_3$, 298 K) of compound **3**.

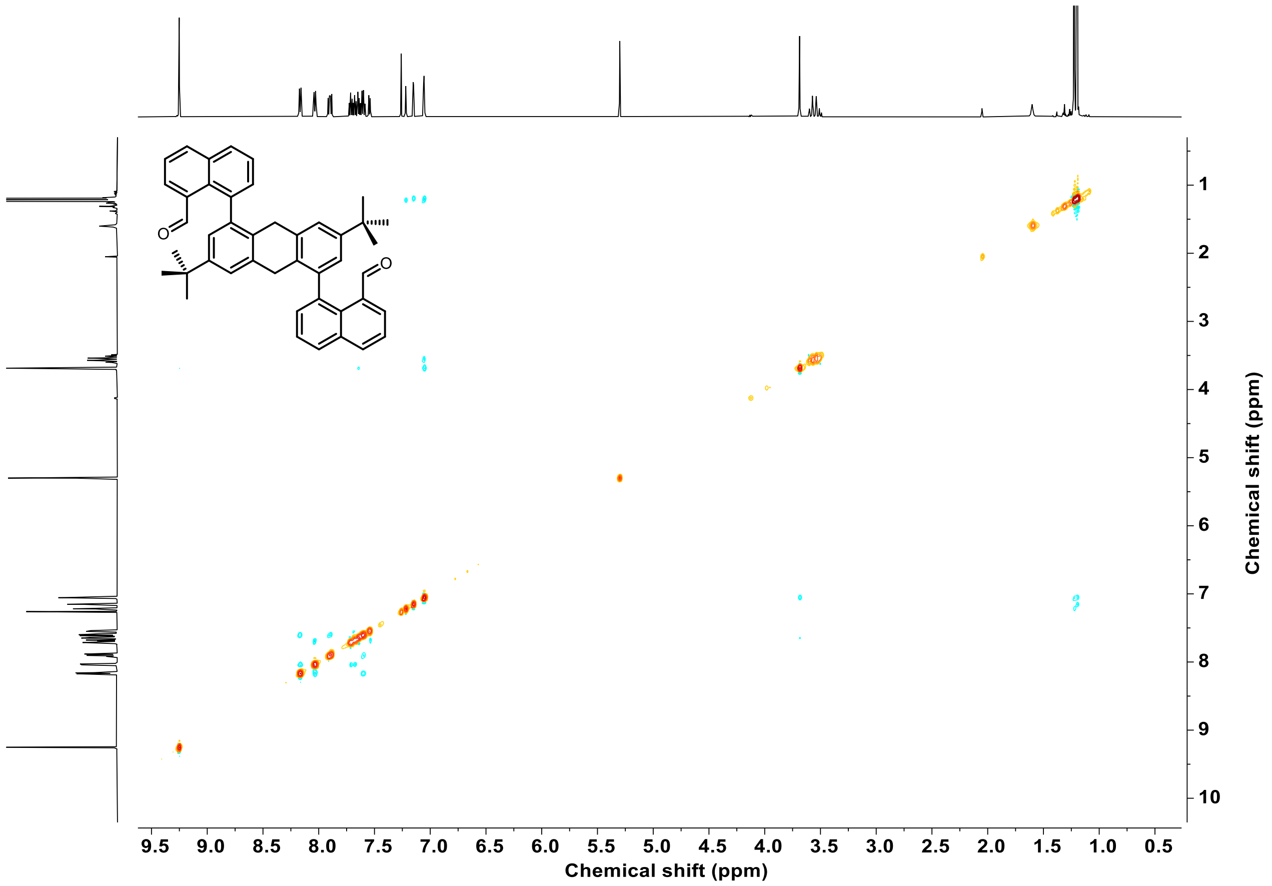

Figure S4. ^{1}H-^{1}H NOESY NMR spectrum (600/600 MHz, $CDCl_3$, 298 K) of compound **3**.

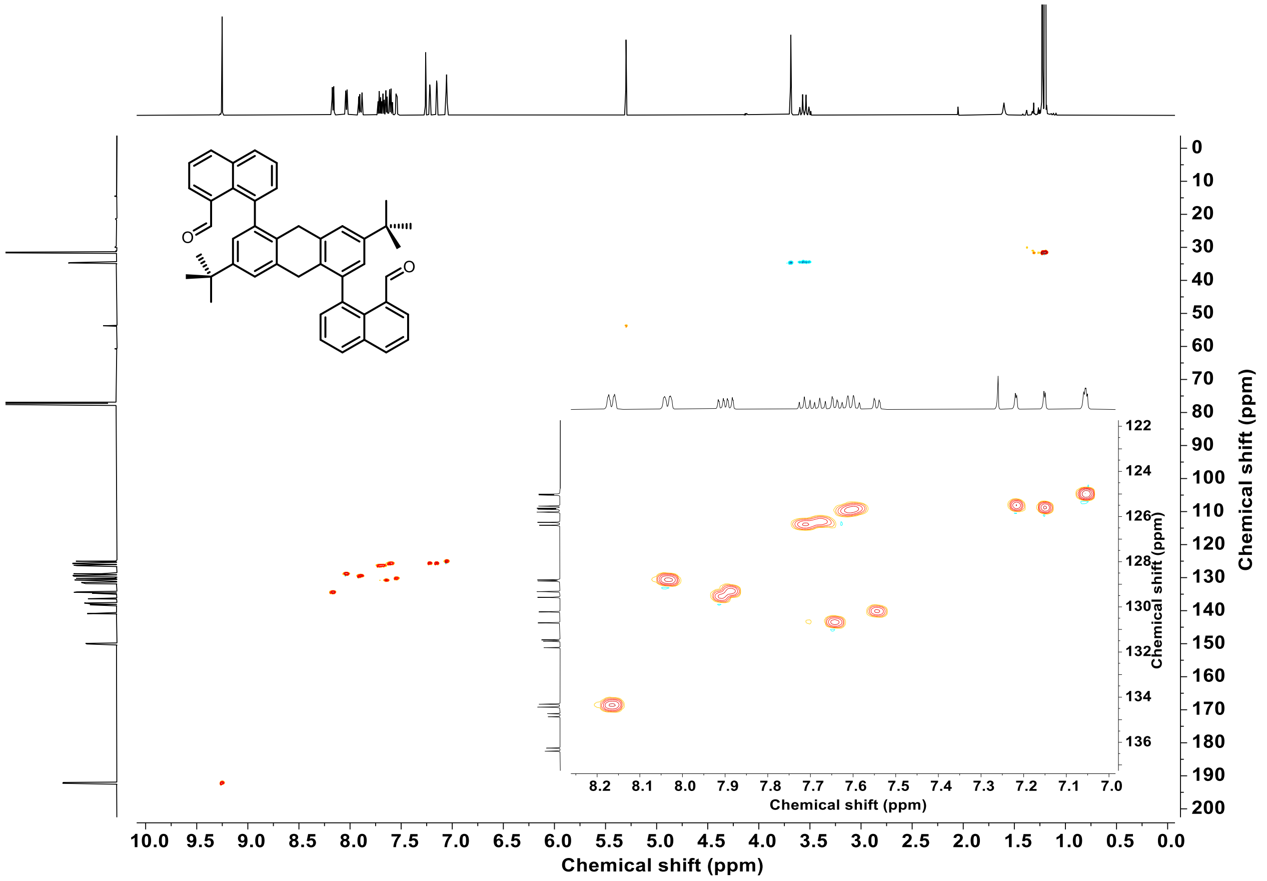

Figure S5. ^{1}H-^{13}C HSQC NMR spectrum (600/151 MHz, $CDCl_3$, 298 K) of compound **3**.

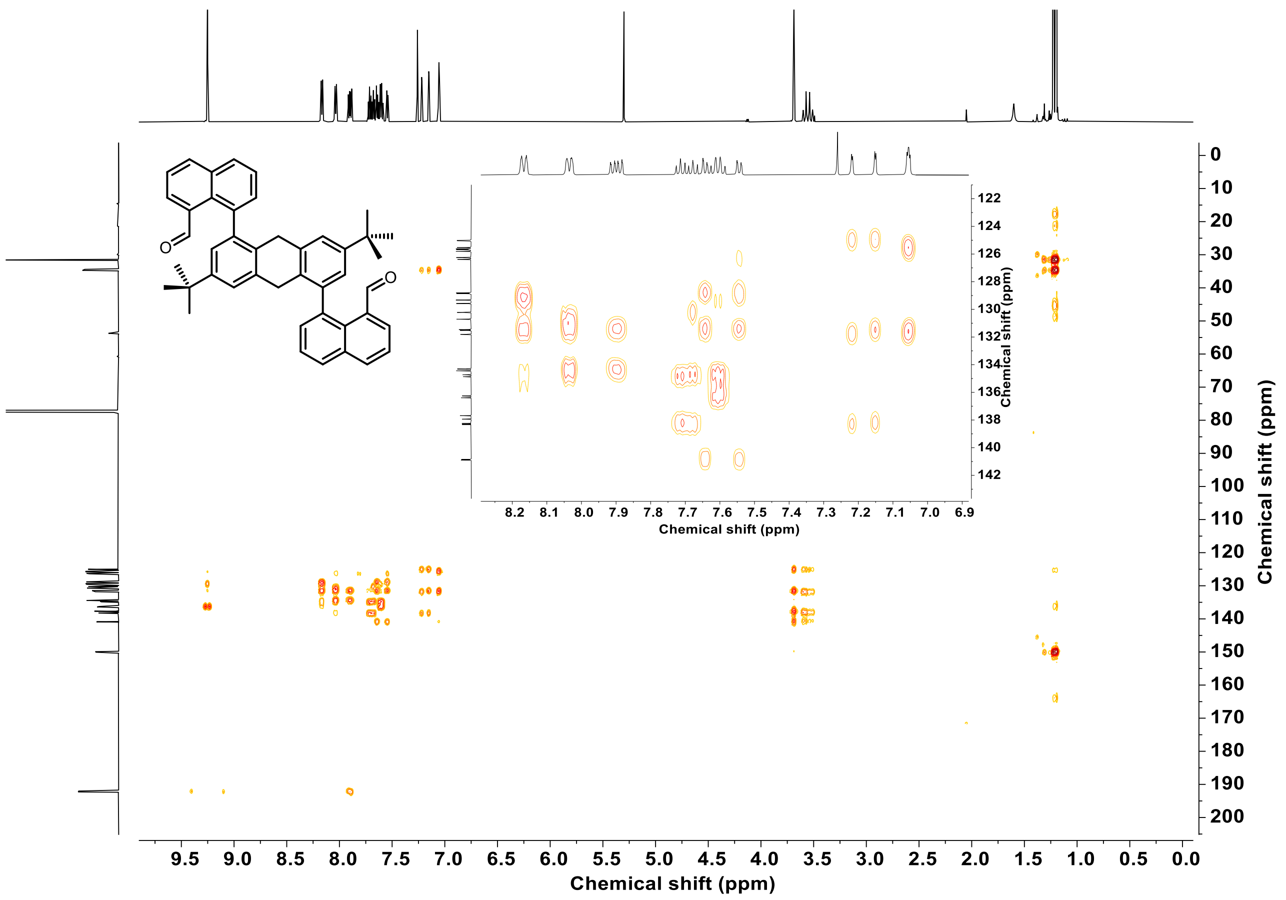

Figure S6. ^{1}H-^{13}C HMBC NMR spectrum (600/151 MHz, $CDCl_3$, 298 K) of compound **3**.

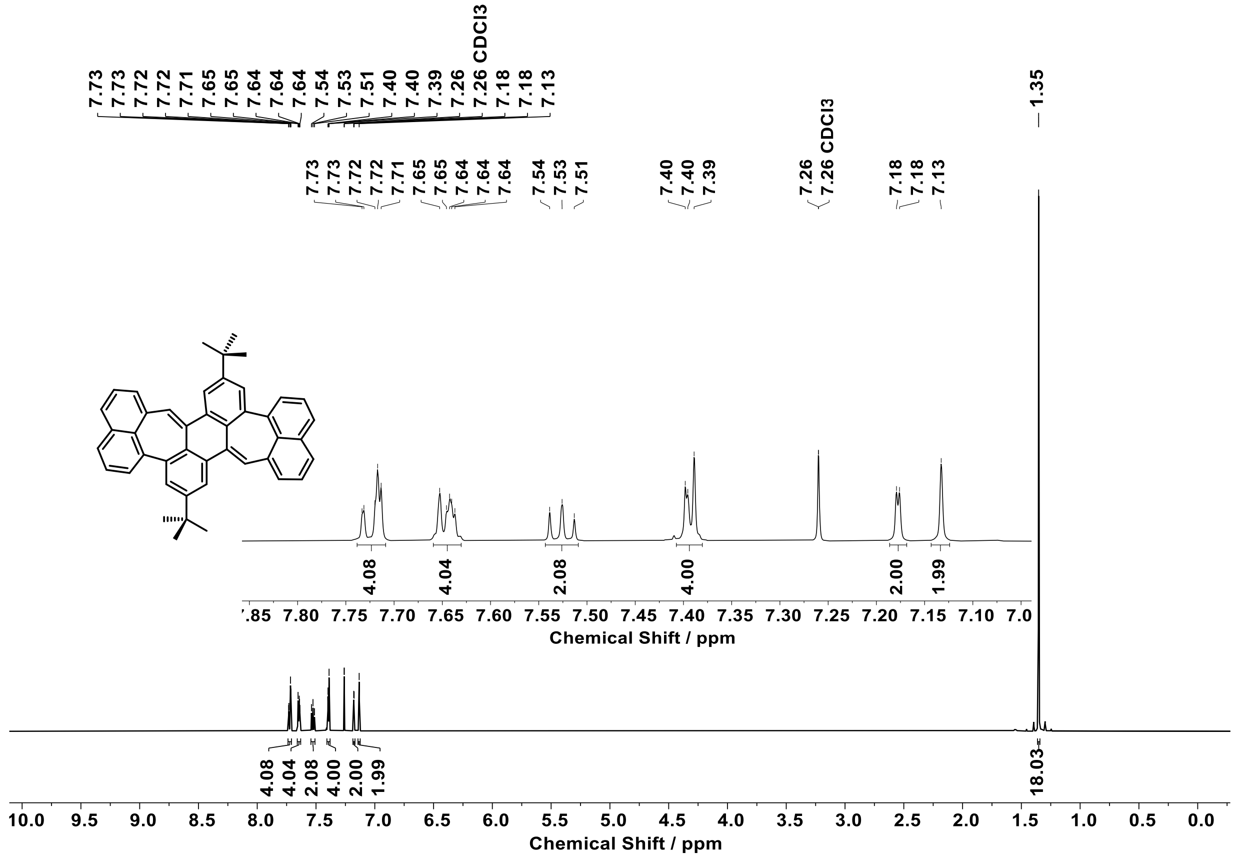

Figure S7. ^{1}H NMR spectrum (600 MHz, $CDCl_3$, 298 K) of compound **4**.

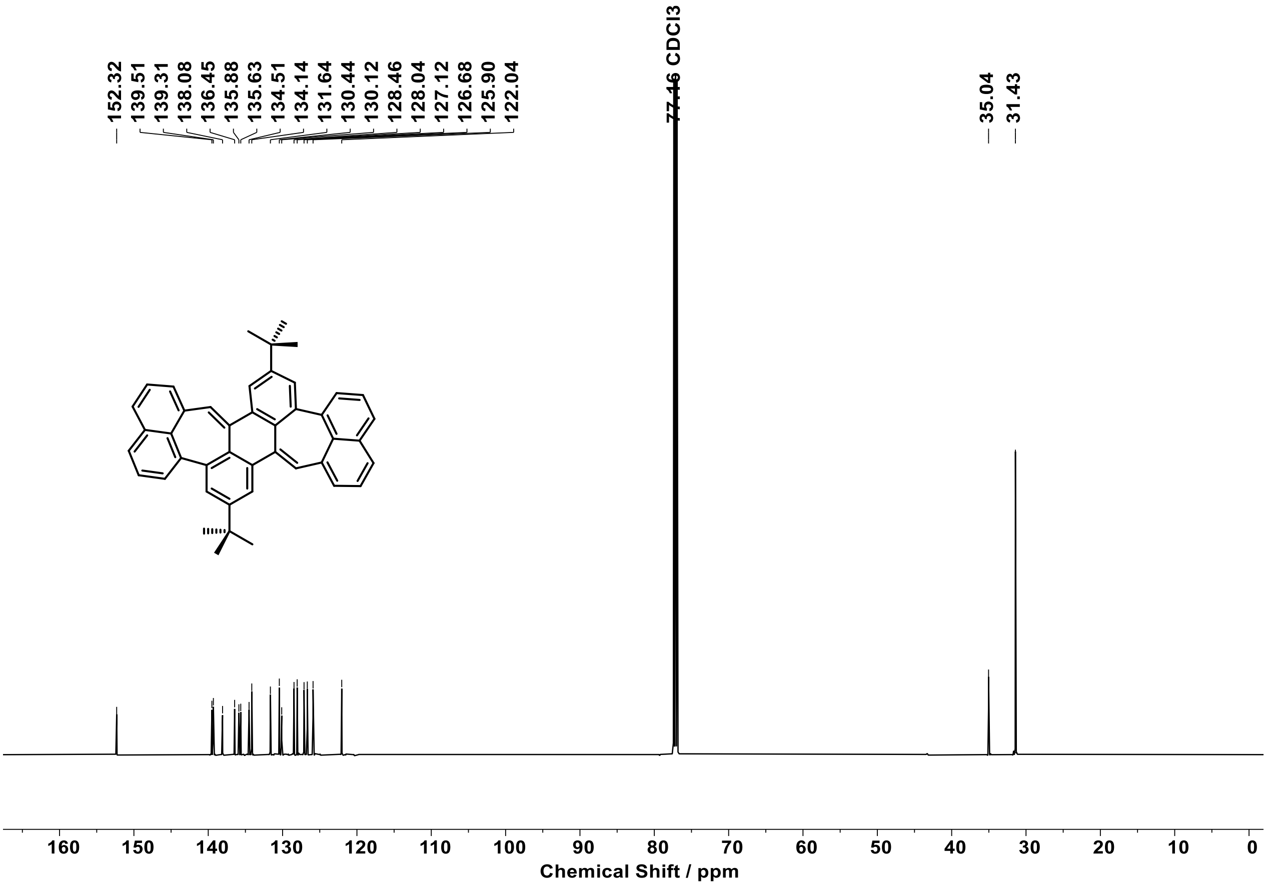

Figure S8. ^{13}C NMR spectrum (151 MHz, $CDCl_3$, 298 K) of compound **4**.

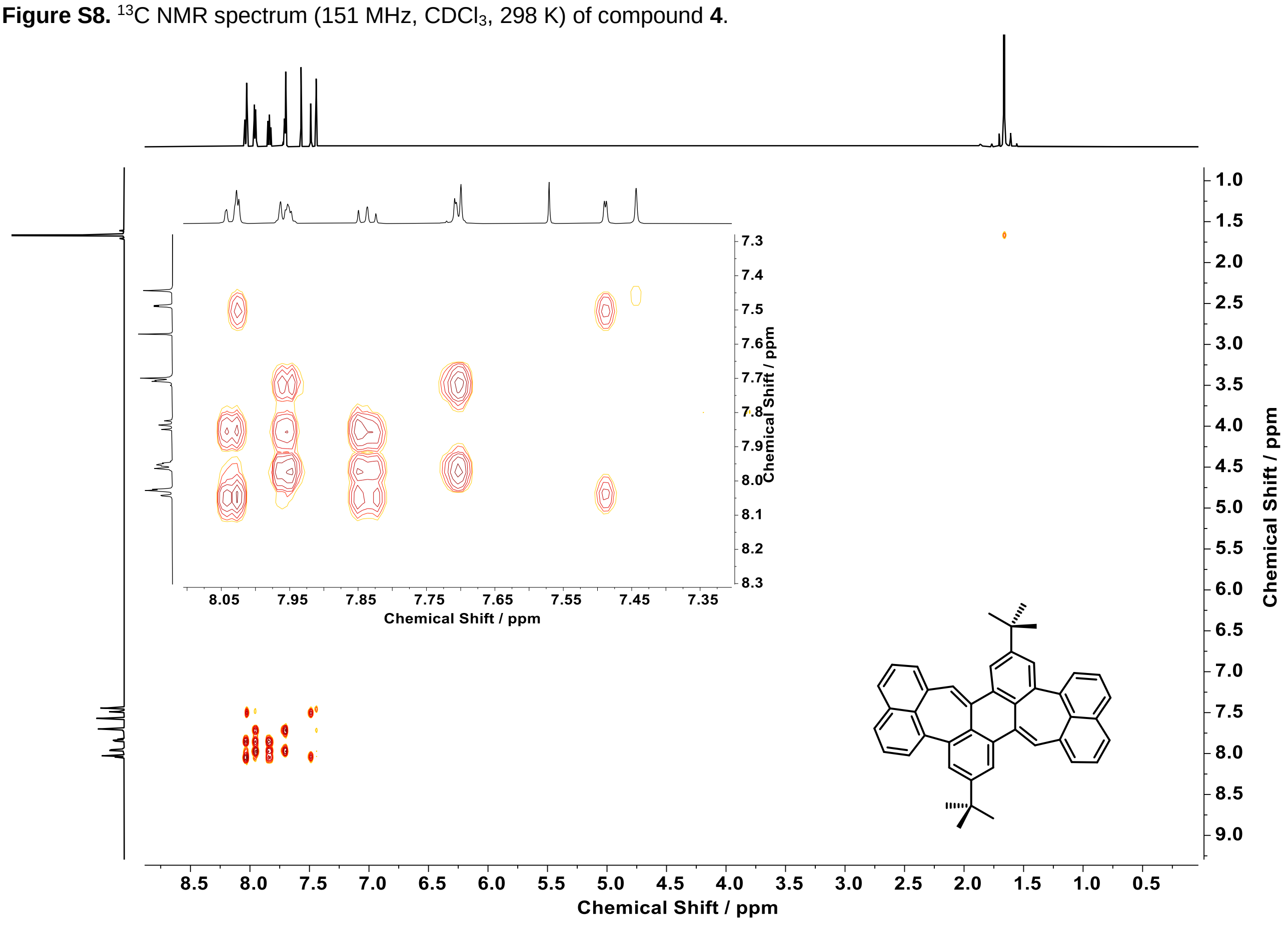

Figure S9. ^{1}H-^{1}H COSY NMR spectrum (600/600 MHz, $CDCl_3$, 298 K) of compound **4**.

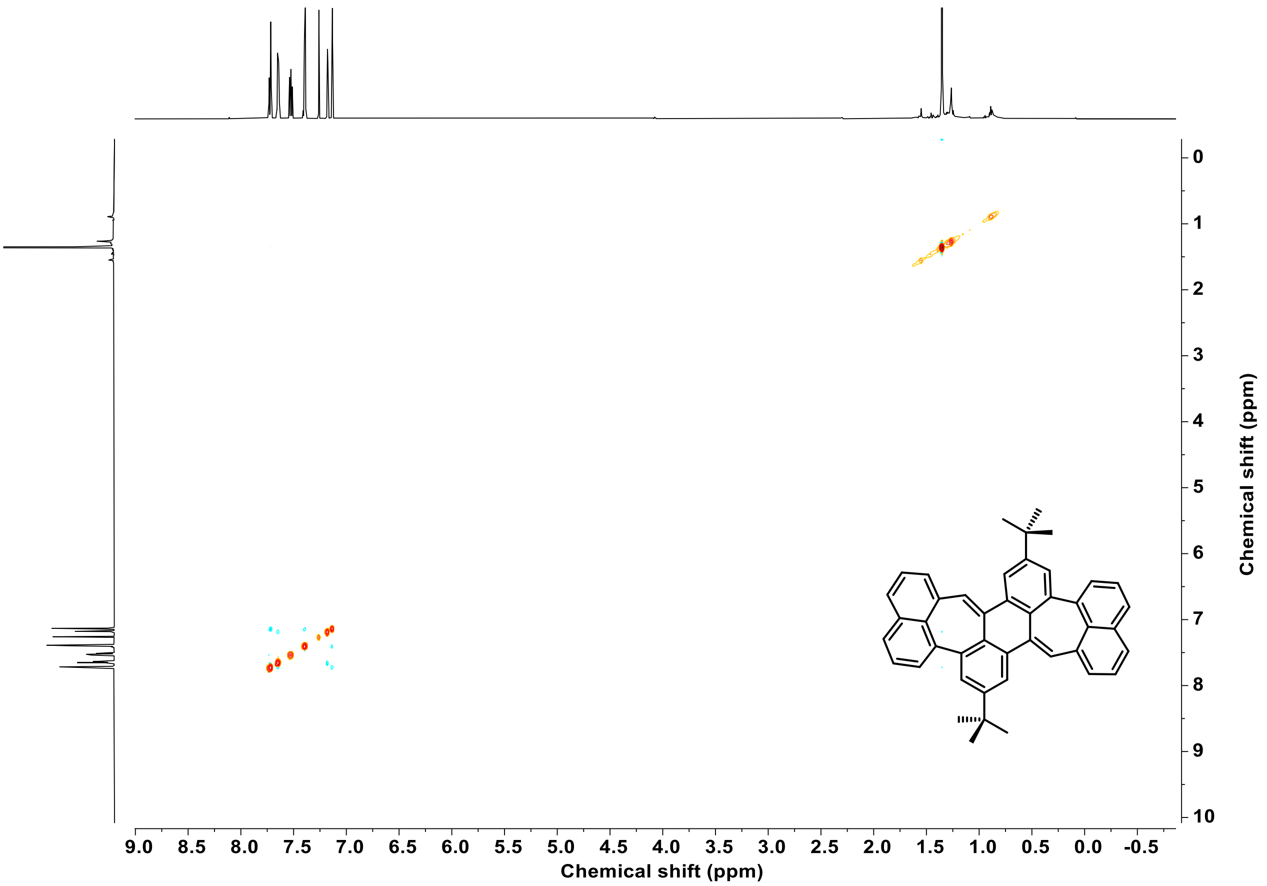

Figure S10. ^{1}H-^{1}H NOESY NMR spectrum (600/600 MHz, THF-d_8, 298 K) of compound **4**.

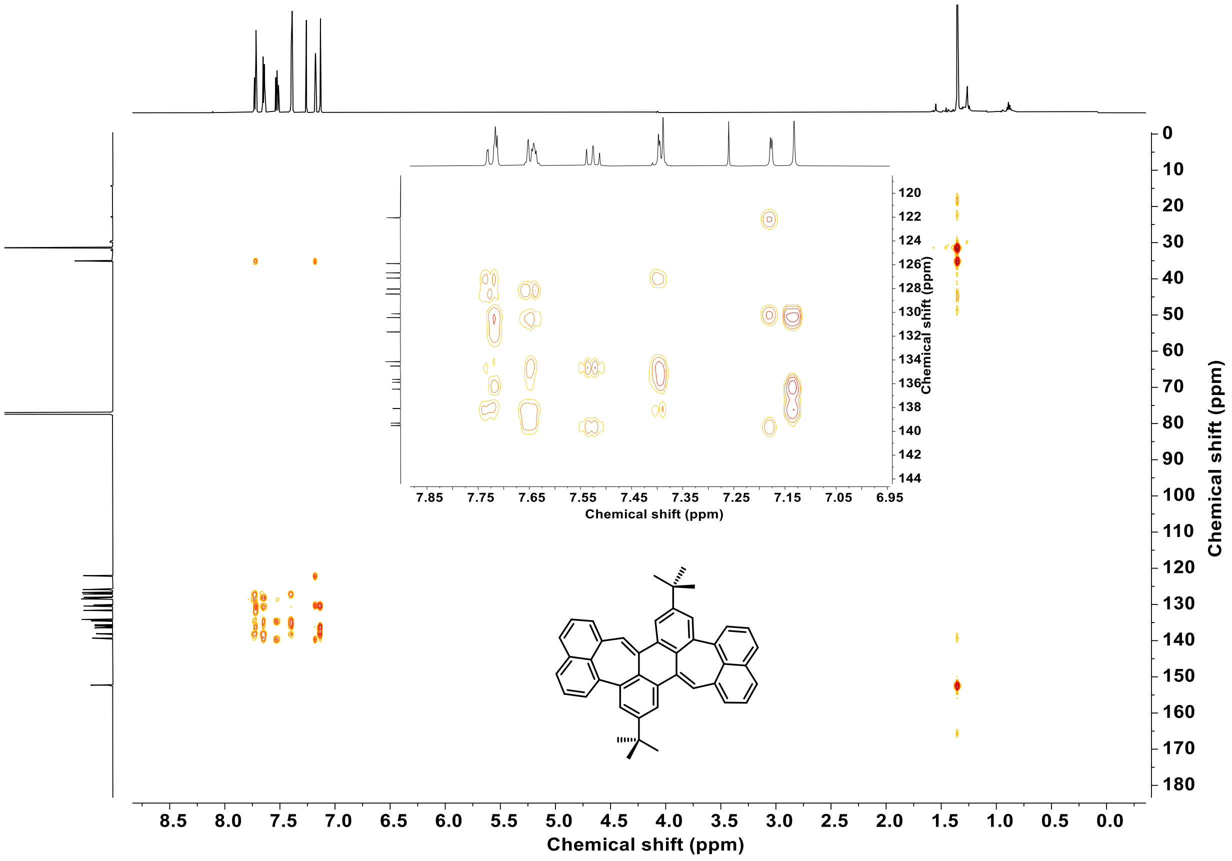

Figure S11. ^{1}H-^{13}C HSQC NMR spectrum (600/151 MHz, $CDCl_3$, 298 K) of compound **4**.

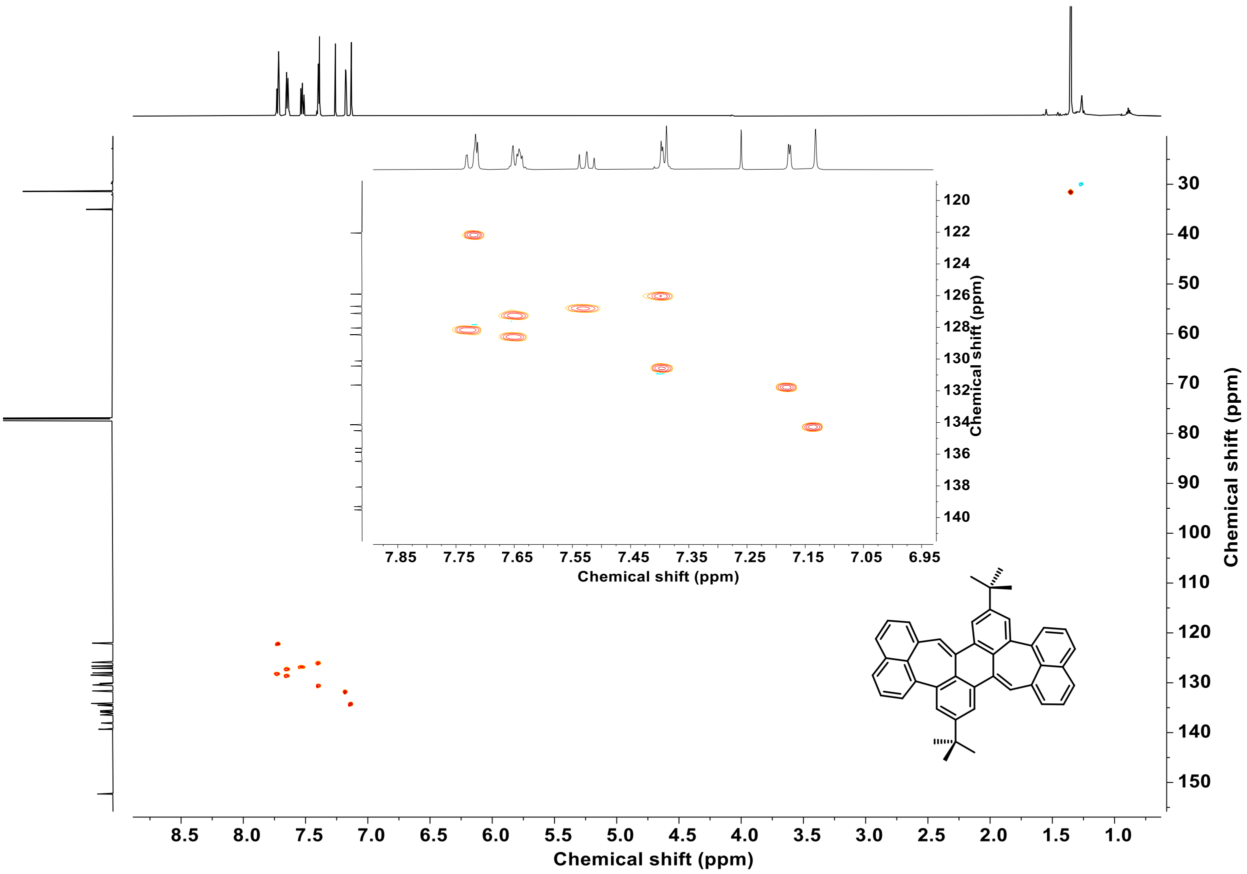

Figure S12. ^1H-^{13}C HMBC NMR spectrum (600/151 MHz, $CDCl_3$, 298 K) of compound **4**.

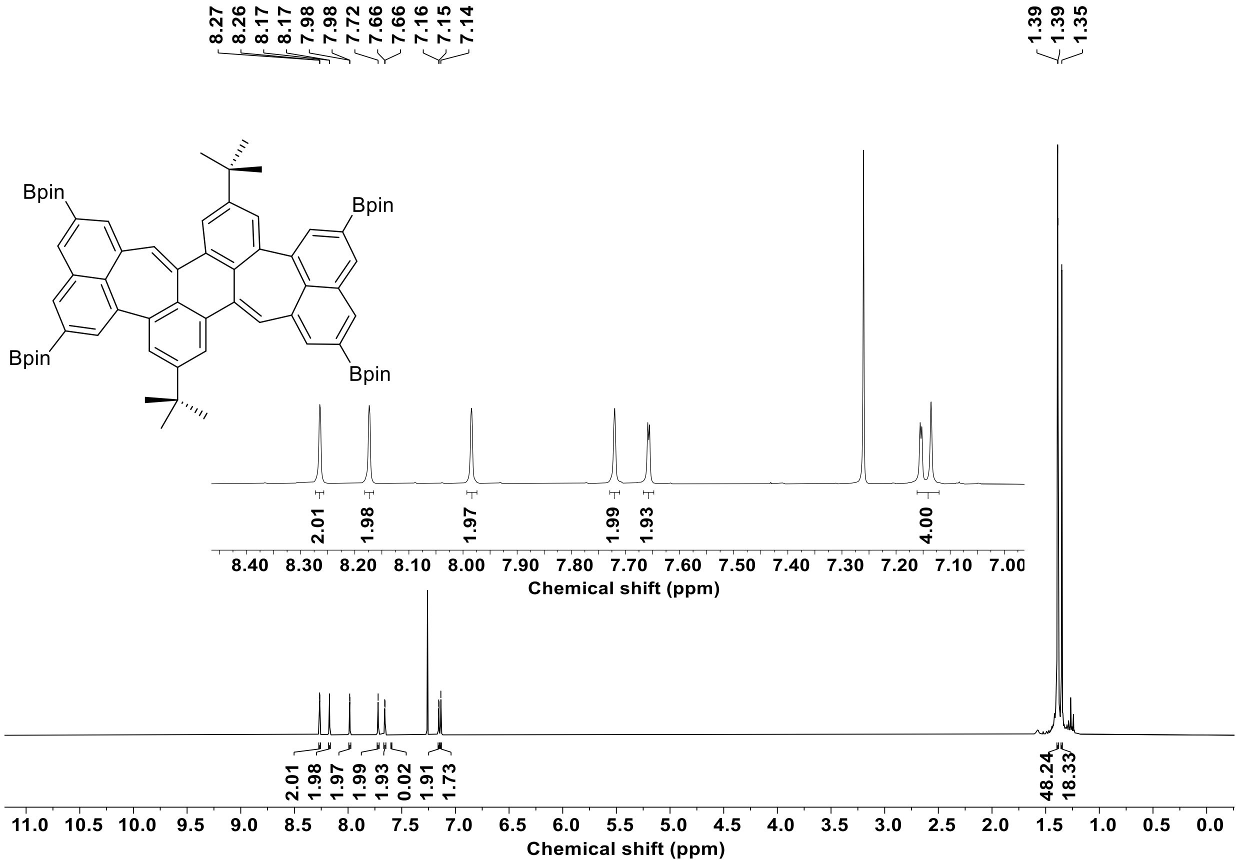

Figure S13. ^1H NMR spectrum (600 MHz, $CDCl_3$, 298 K) of compound **5**.

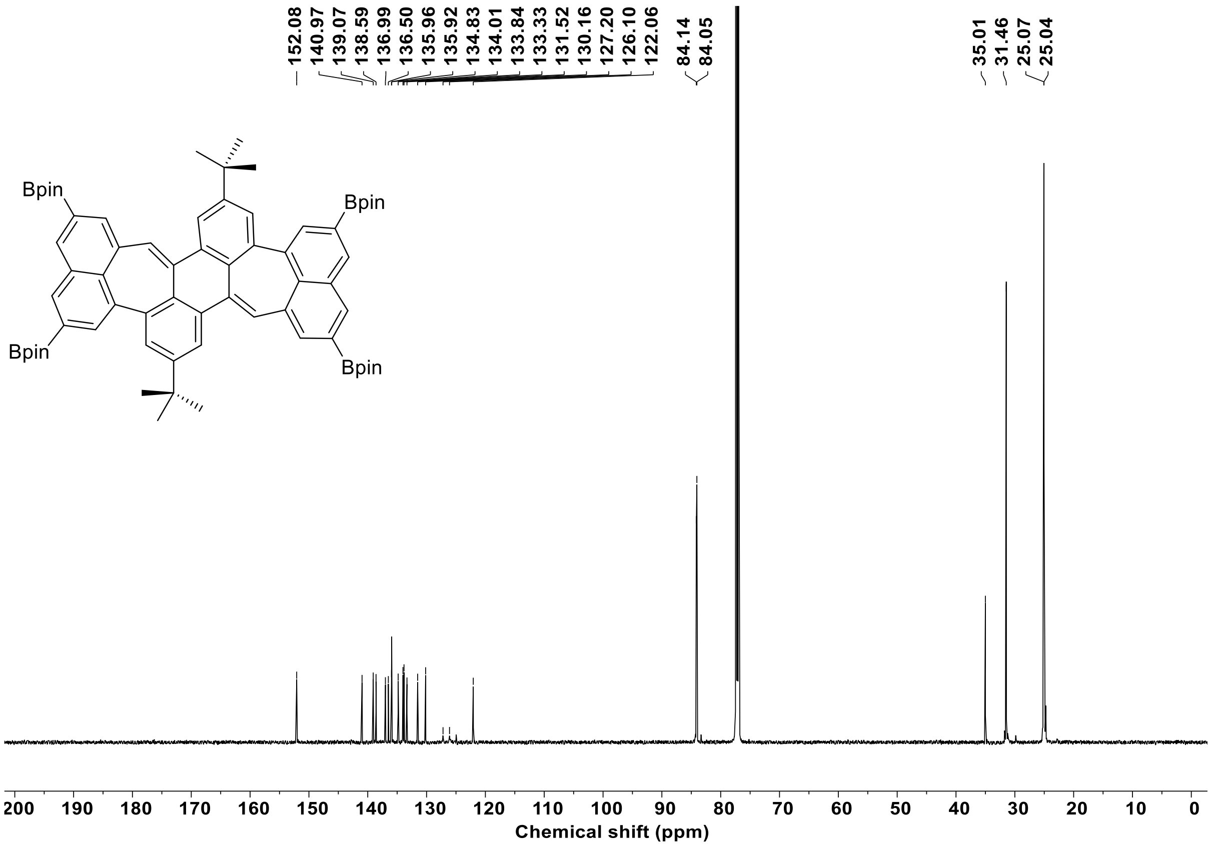

Figure S14. ^{13}C NMR spectrum (151 MHz, $CDCl_3$, 298 K) of compound **5**.

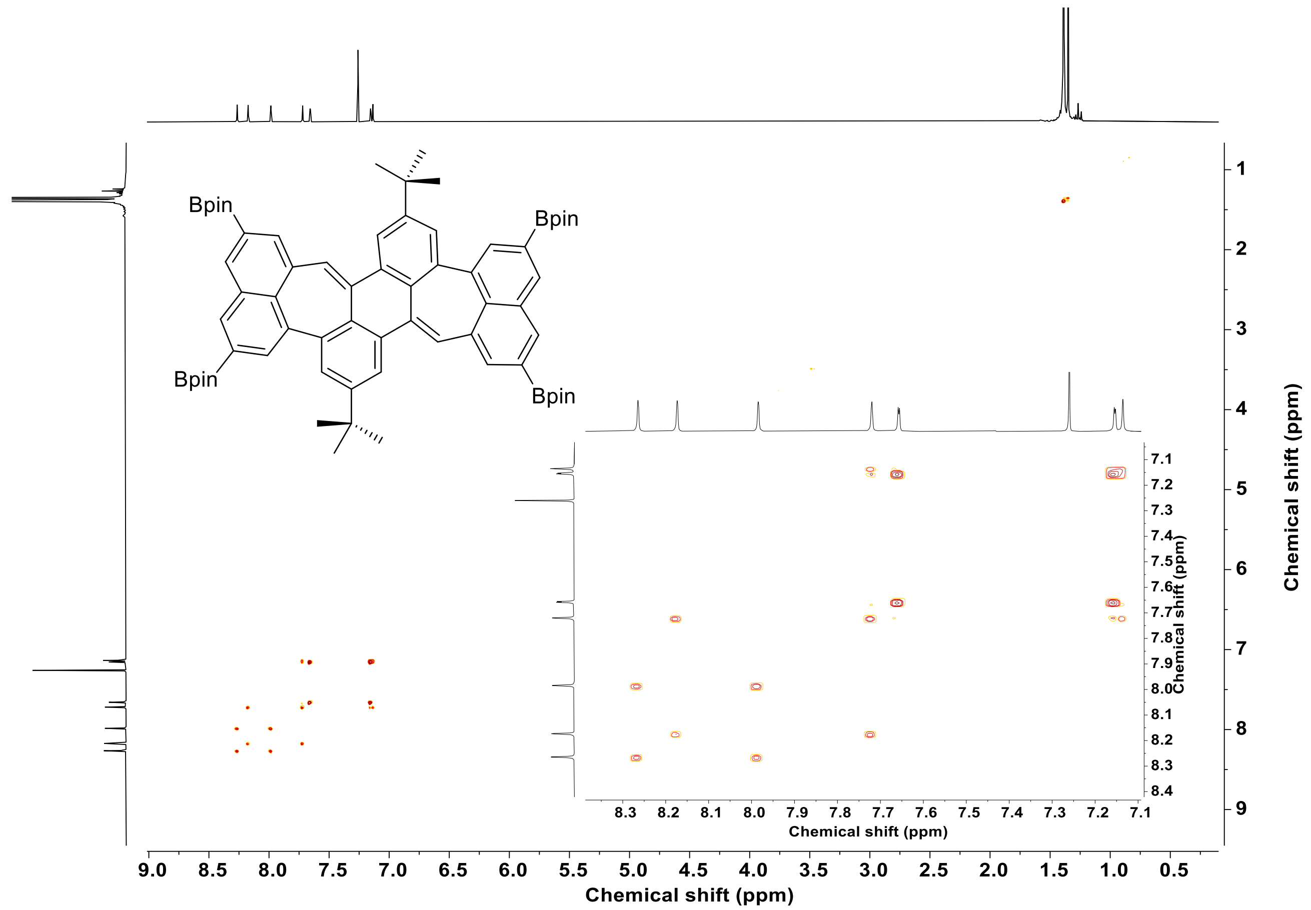

Figure S15. ^1H-^1H COSY NMR spectrum (600/600 MHz, $CDCl_3$, 298 K) of compound **5**.

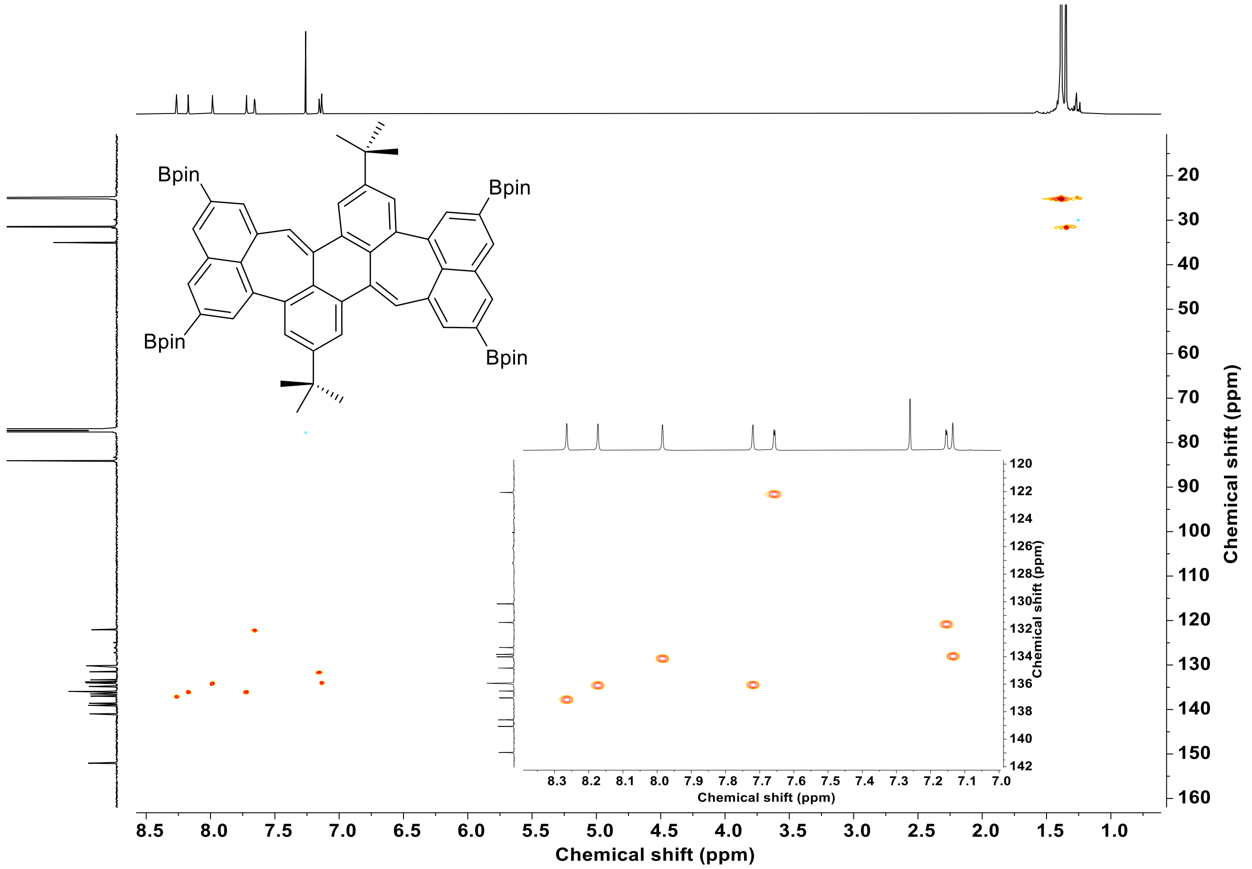

Figure S16. ^{1}H-^{13}C HSQC NMR spectrum (600/151 MHz, $CDCl_3$, 298 K) of compound **5**.

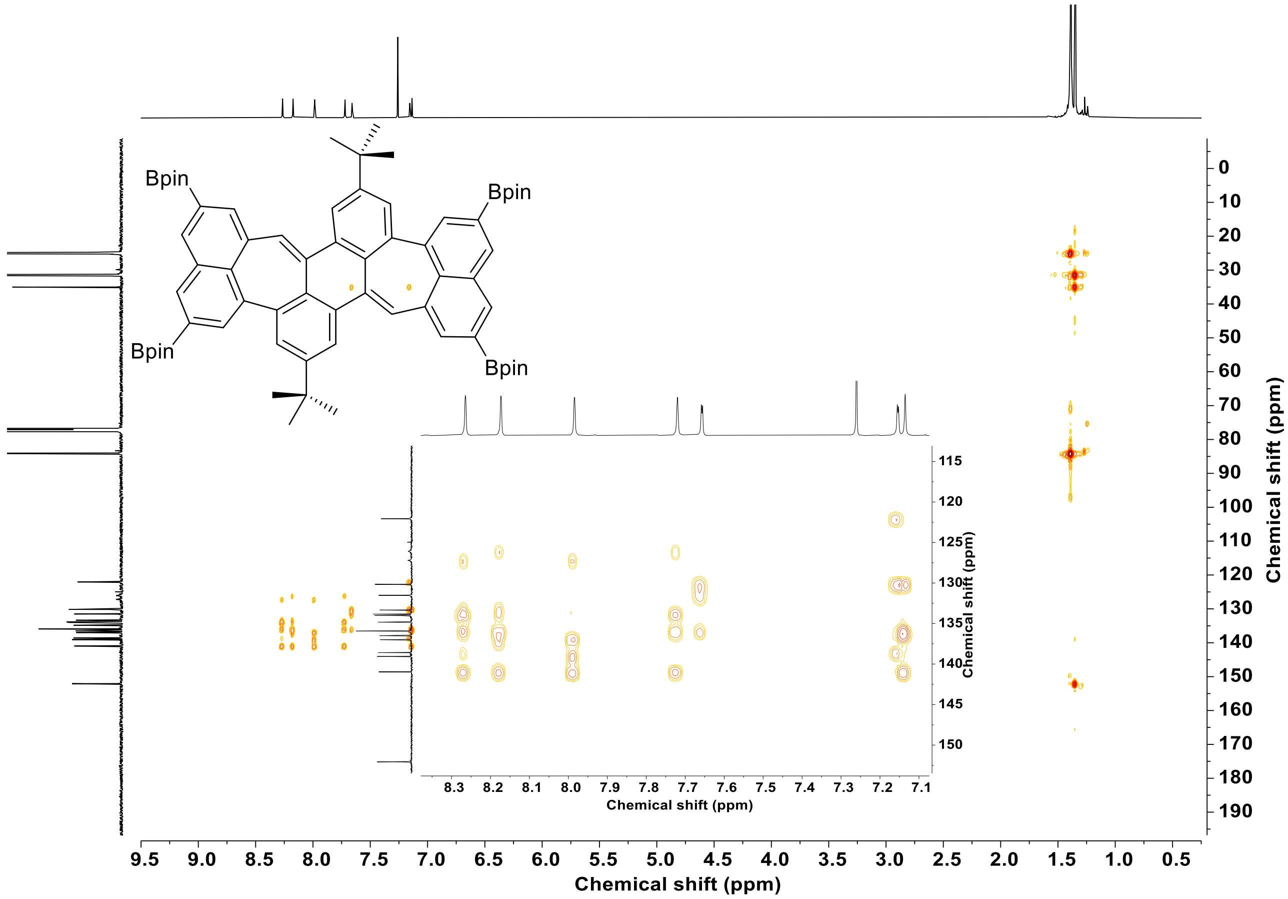

Figure S17. ^{1}H-^{13}C HMBC NMR spectrum (600/151 MHz, $CDCl_3$, 298 K) of compound **5**.

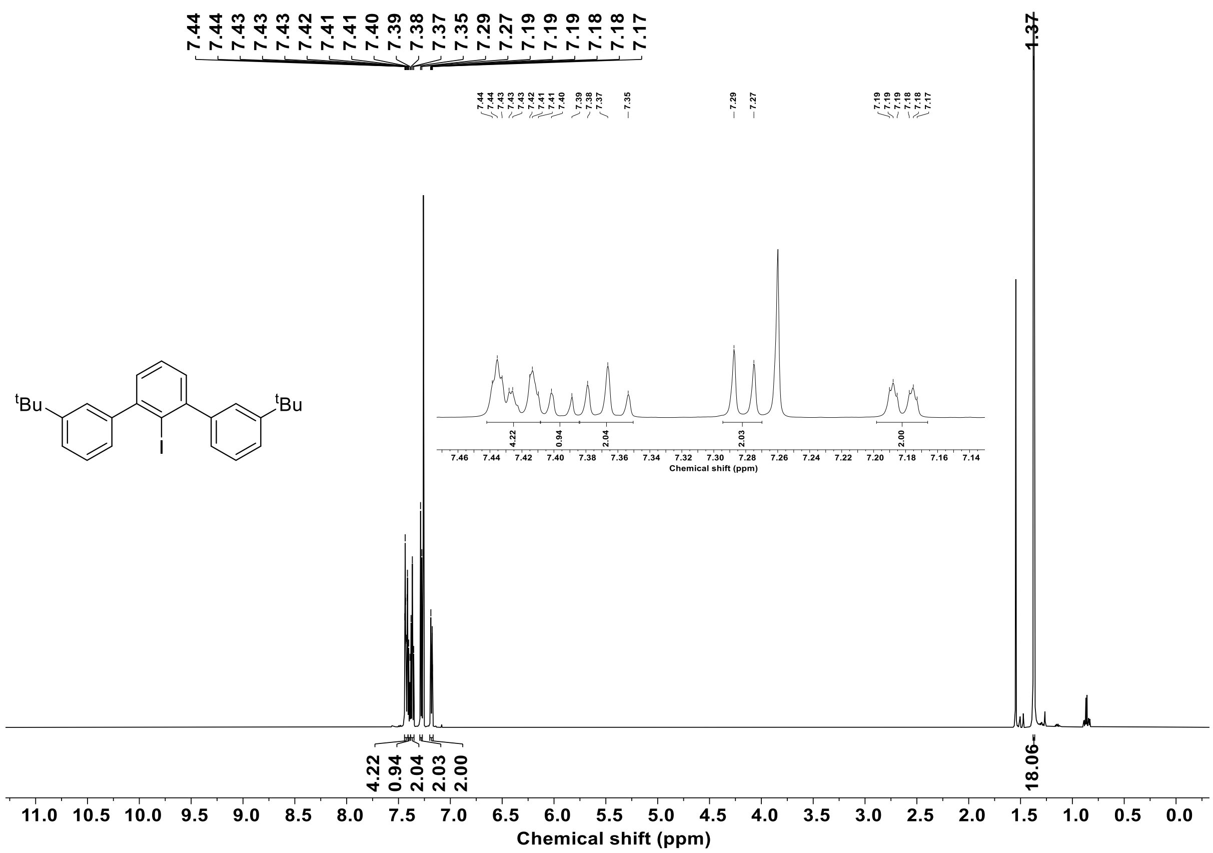

Figure S18. ^{1}H NMR spectrum (600 MHz, $CDCl_3$, 298 K) of compound **6**.

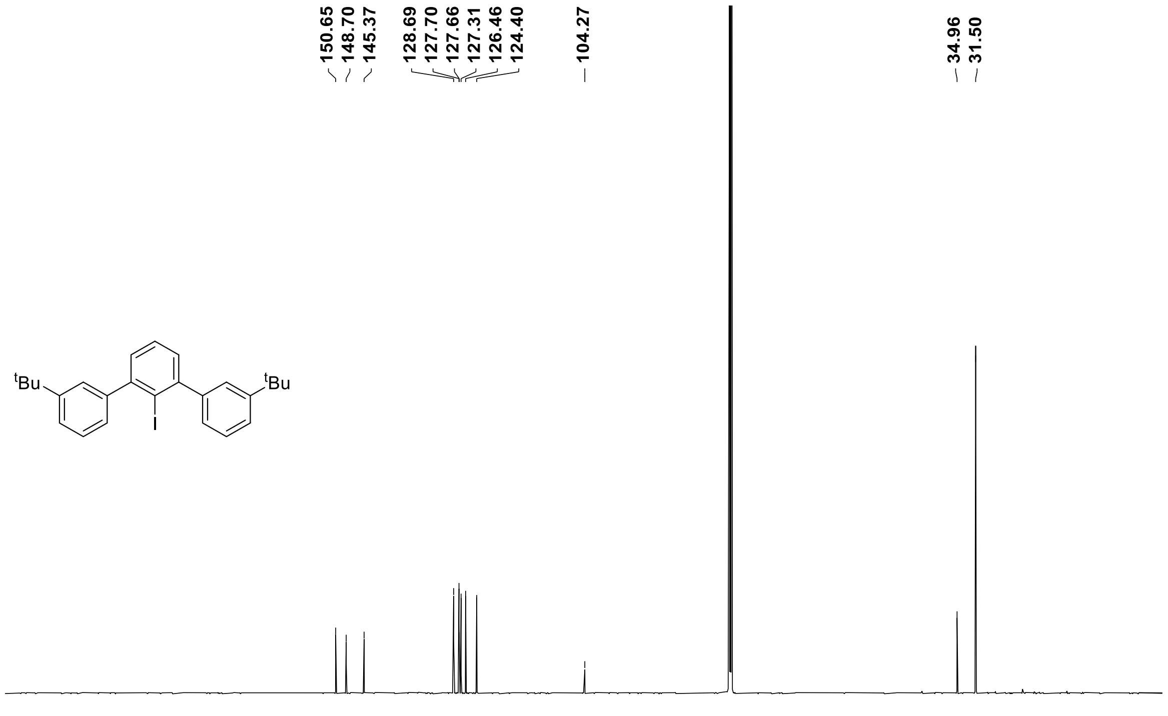

Figure S19. ^{13}C NMR spectrum (151 MHz, $CDCl_3$, 298 K) of compound **6**.

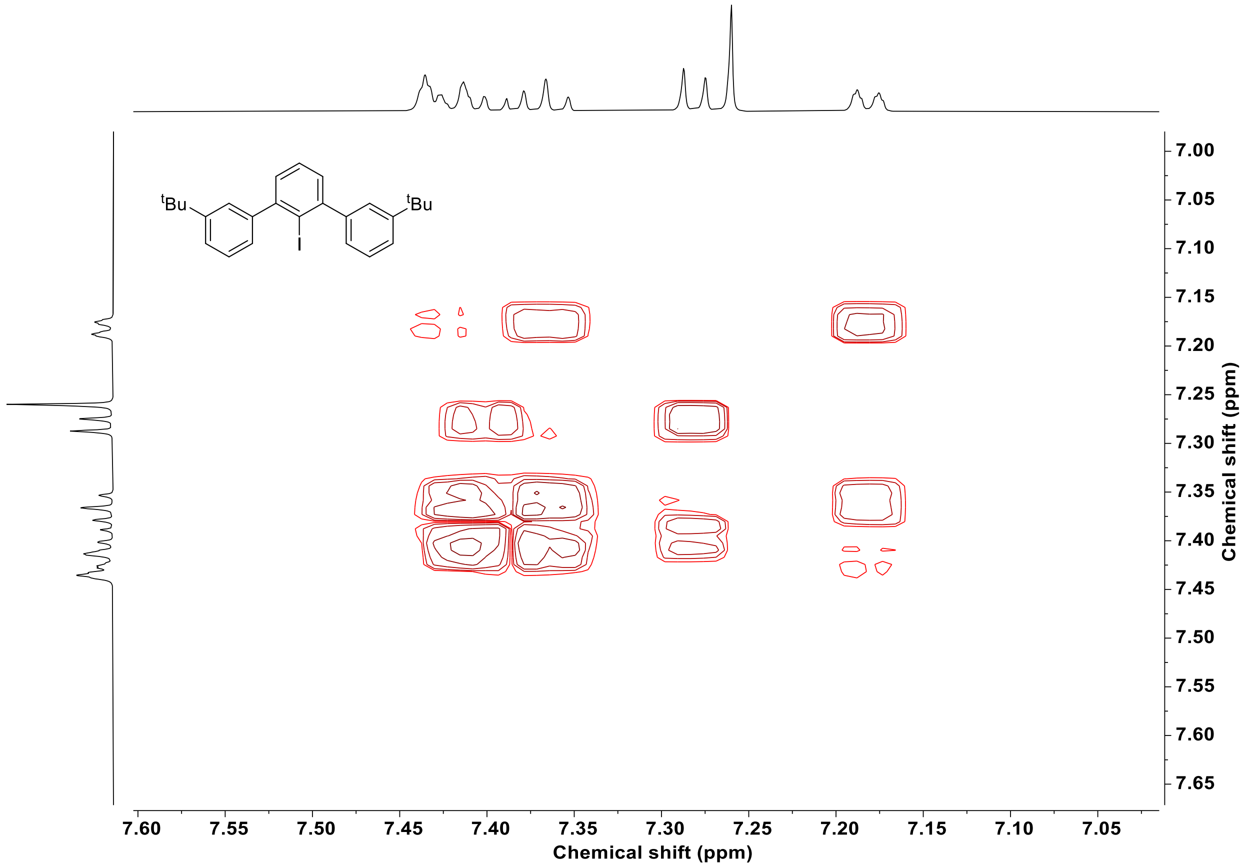

Figure S20. ^{1}H-^{1}H COSY NMR spectrum (600/600 MHz, $CDCl_3$, 298 K) of compound **6**.

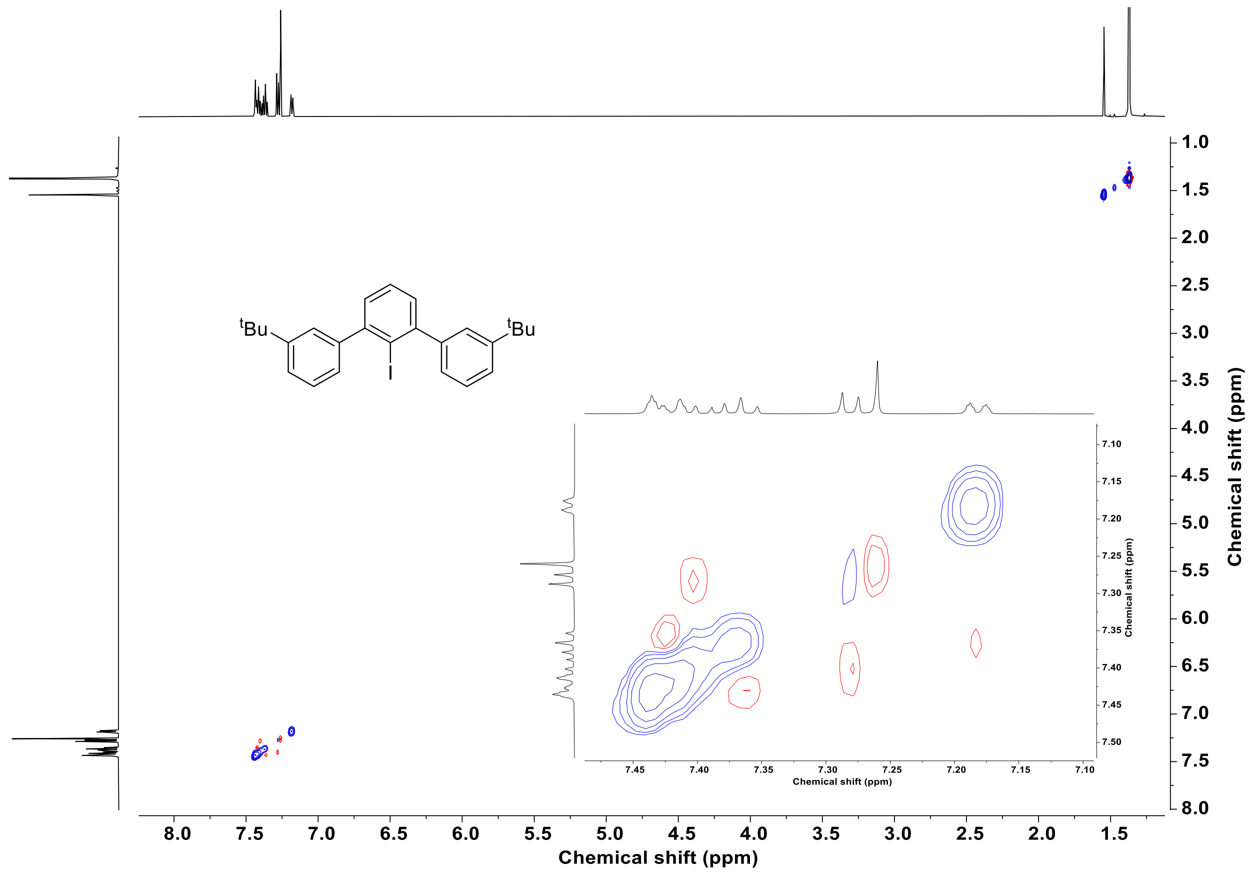

Figure S21. ^{1}H-^{1}H NOESY NMR spectrum (600/600 MHz, THF-d_8, 298 K) of compound **6**.

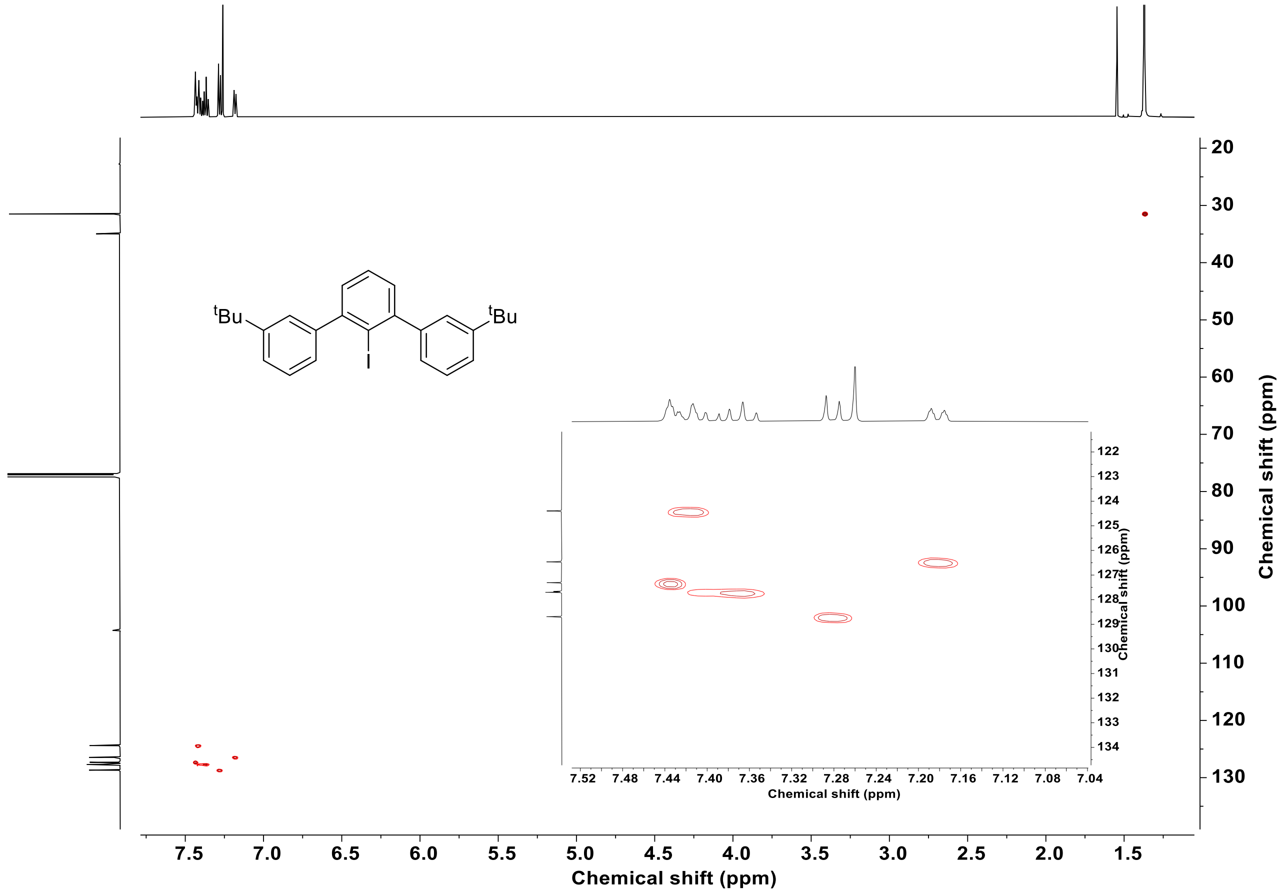

Figure S22. ^1H-^{13}C HSQC NMR spectrum (600/151 MHz, $CDCl_3$, 298 K) of compound **6**.

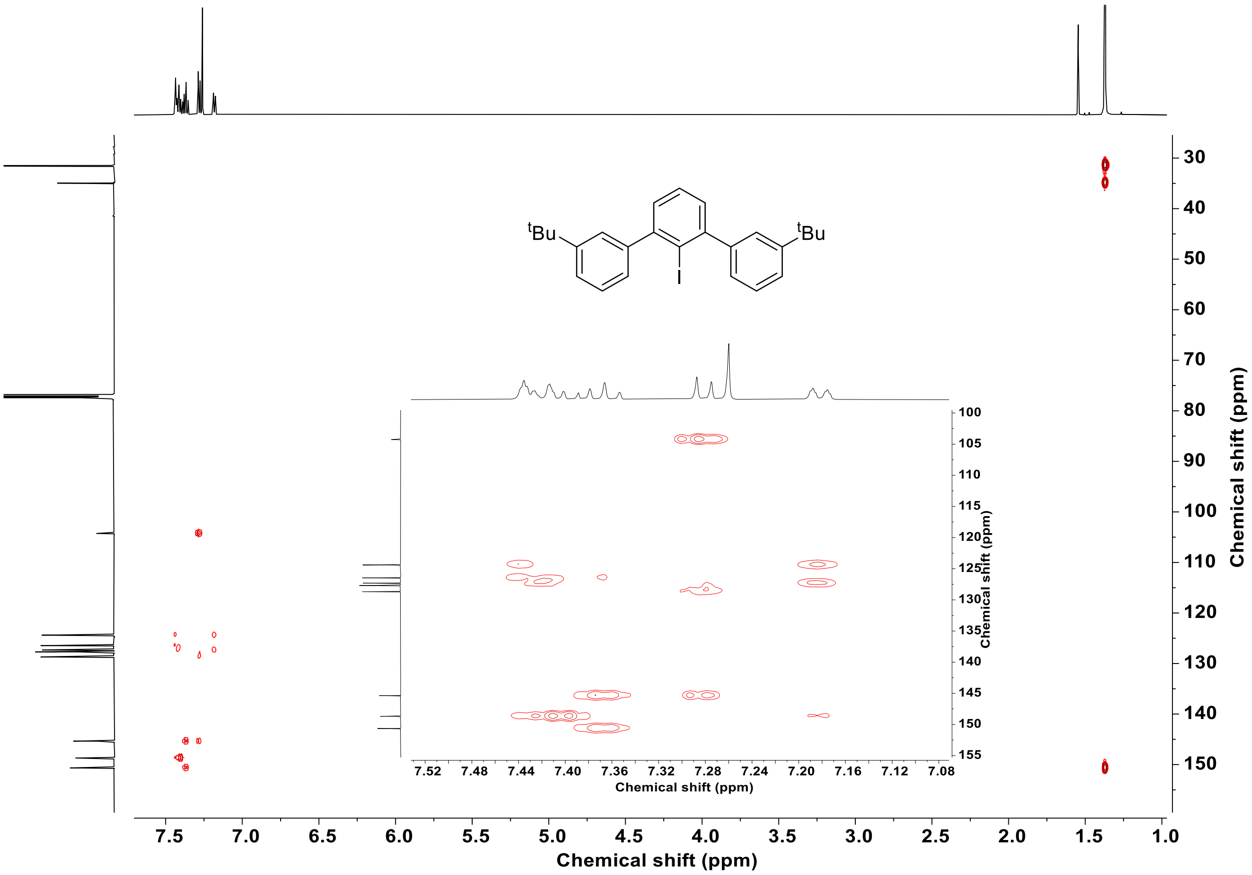

Figure S23. ^1H-^{13}C HMBC NMR spectrum (600/151 MHz, $CDCl_3$, 298 K) of compound **6**.

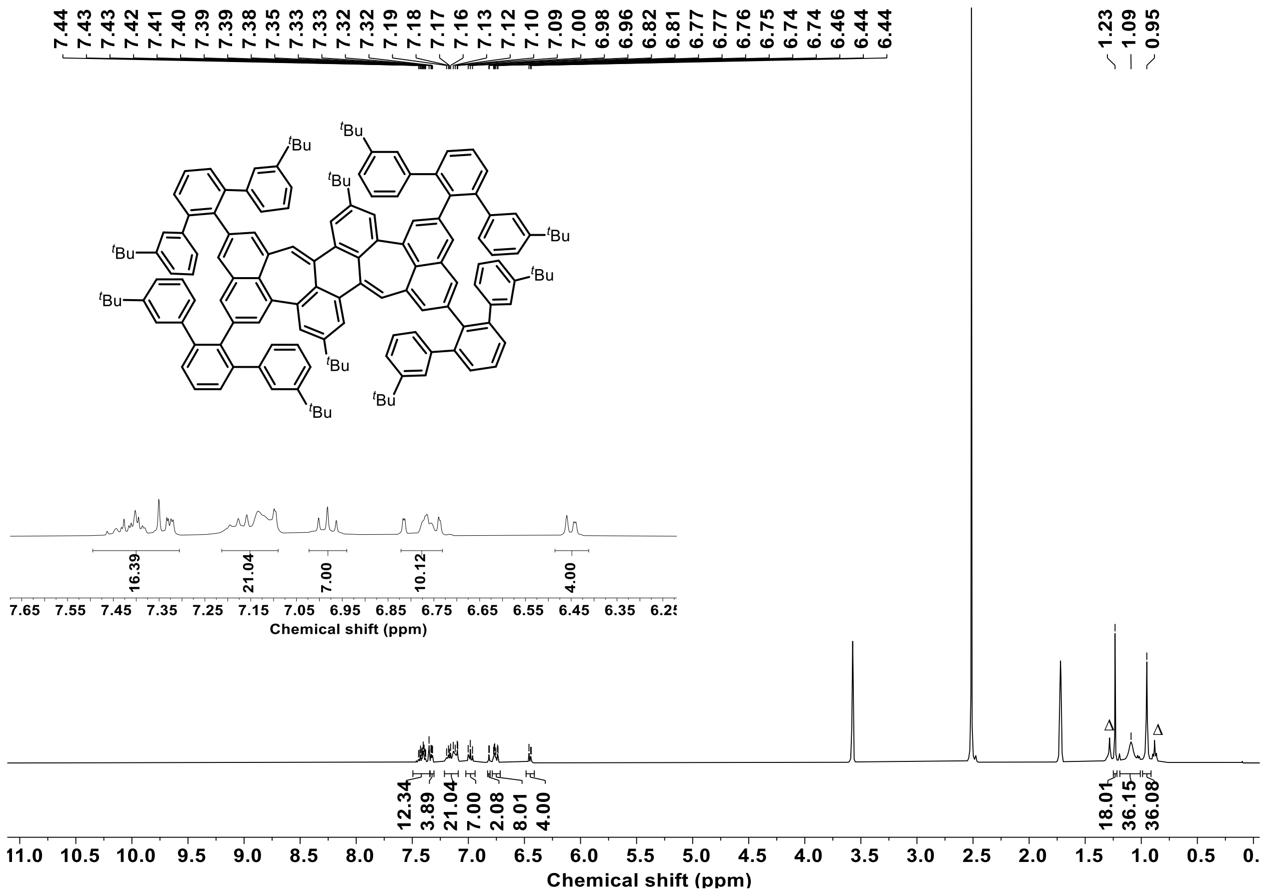

Figure S24. ^{1}H NMR spectrum (400 MHz, THF-d_8, 298 K) of compound **7**. Δ: heptane.

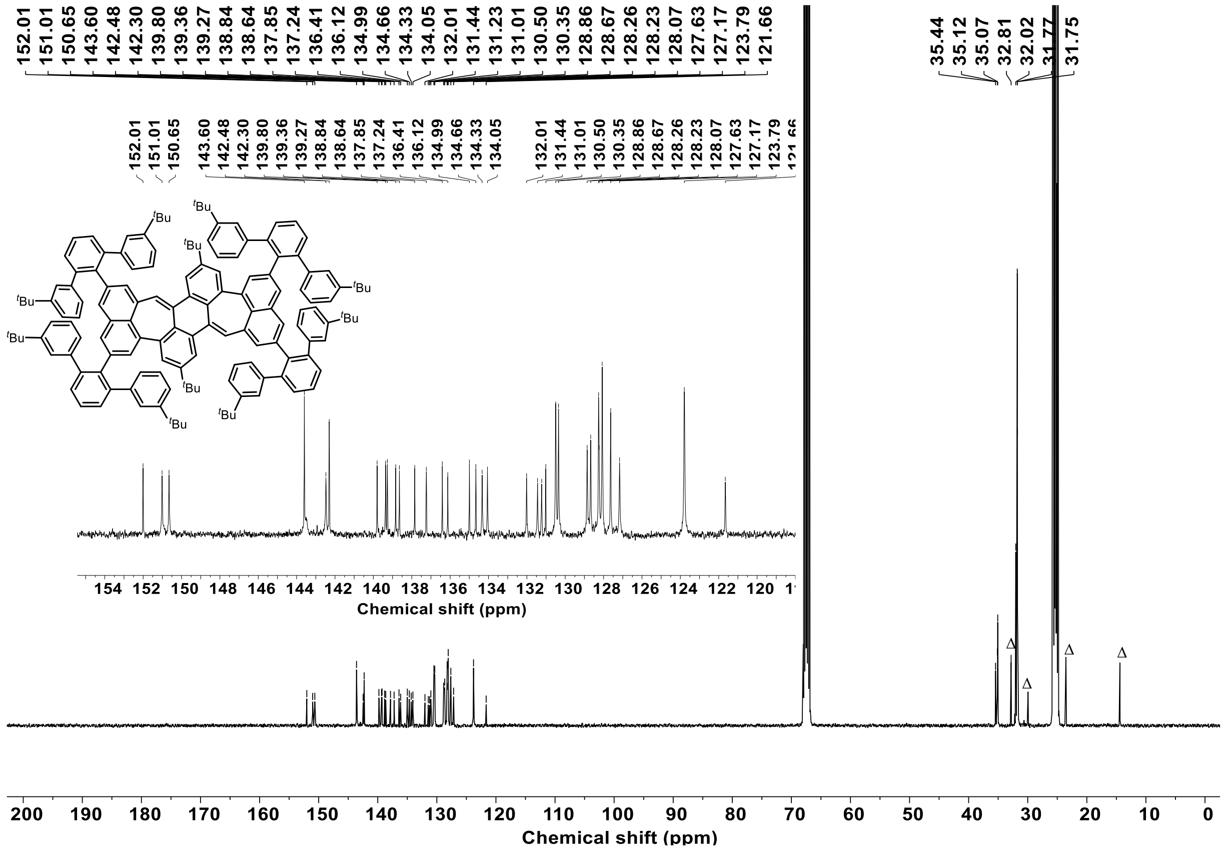

Figure S25. ^{13}C NMR spectrum (101 MHz, THF-d_8, 298 K) of compound **7**. Δ: heptane.

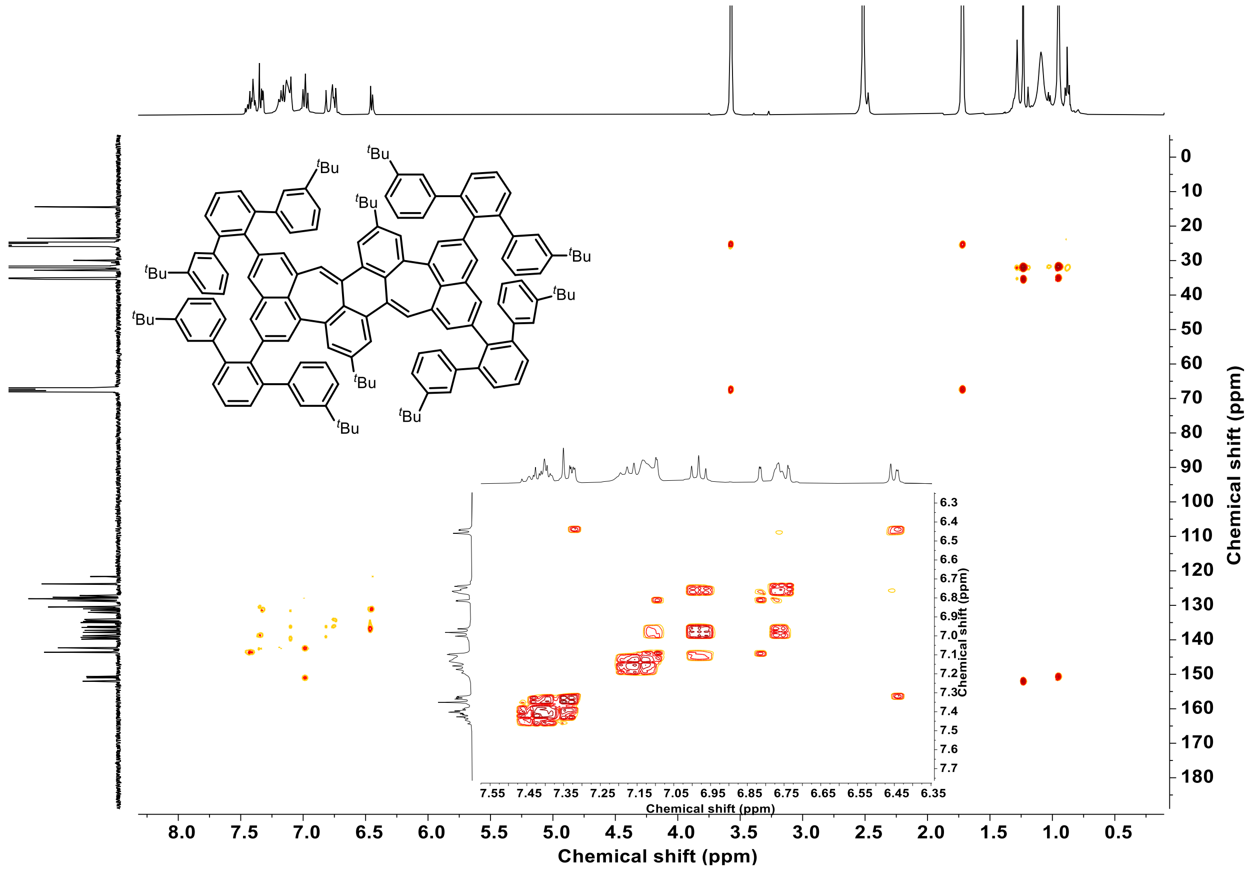

Figure S26. ^{1}H-^{1}H COSY NMR spectrum (400/400 MHz, THF-d_8, 298 K) of compound **7**.

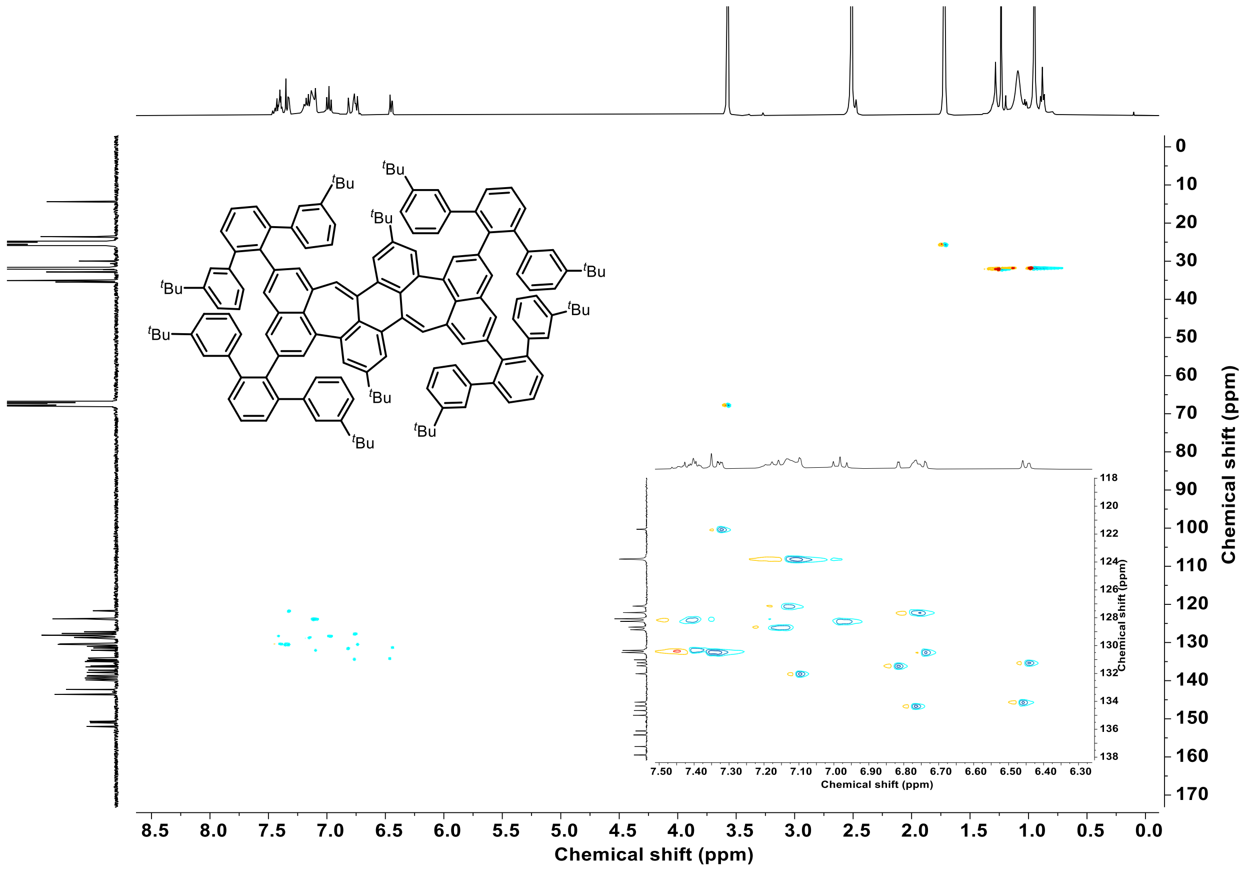

Figure S27. ^{1}H-^{13}C HSQC NMR spectrum (400/101 MHz, THF-d_8, 298 K) of compound **7**.

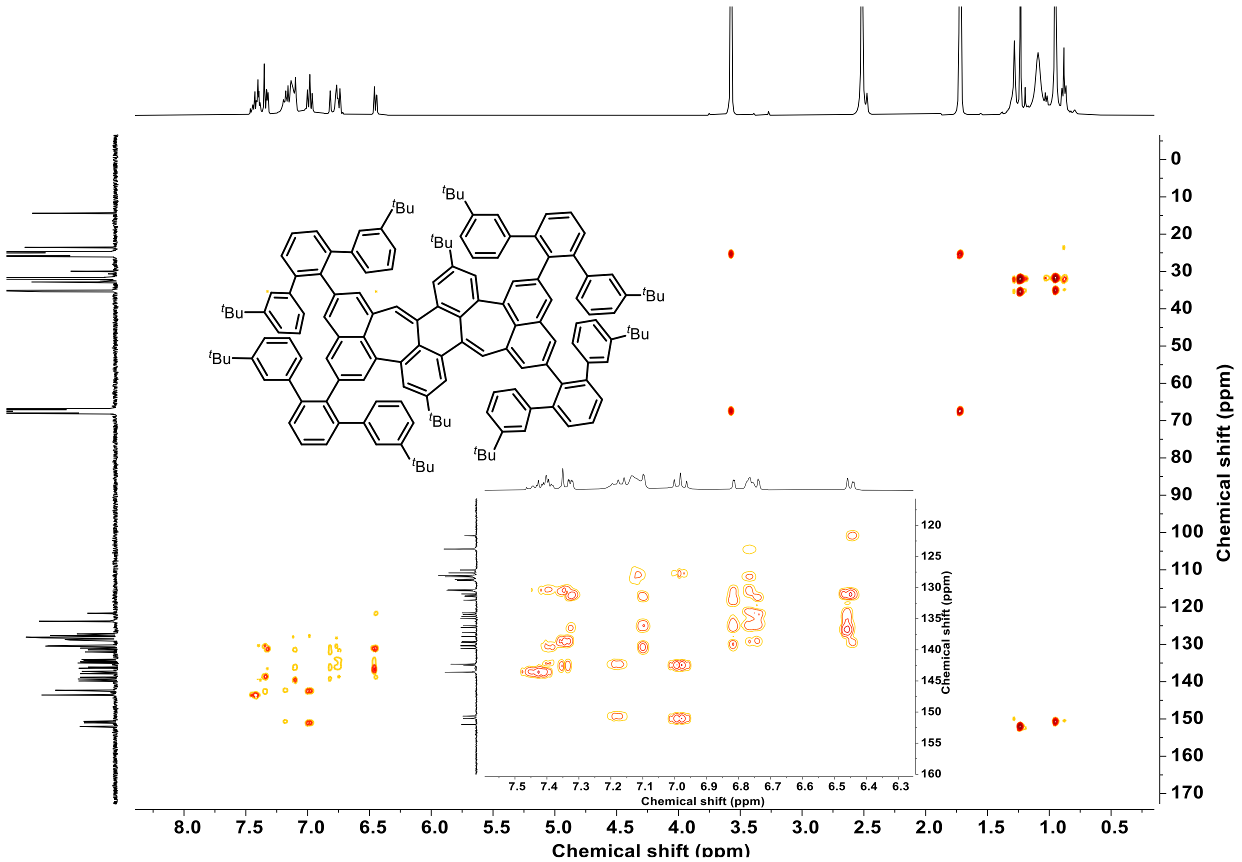

Figure S28. ^{1}H-^{13}C HMBC NMR spectrum (400/101 MHz, THF-d_8, 298 K) of compound **7**.

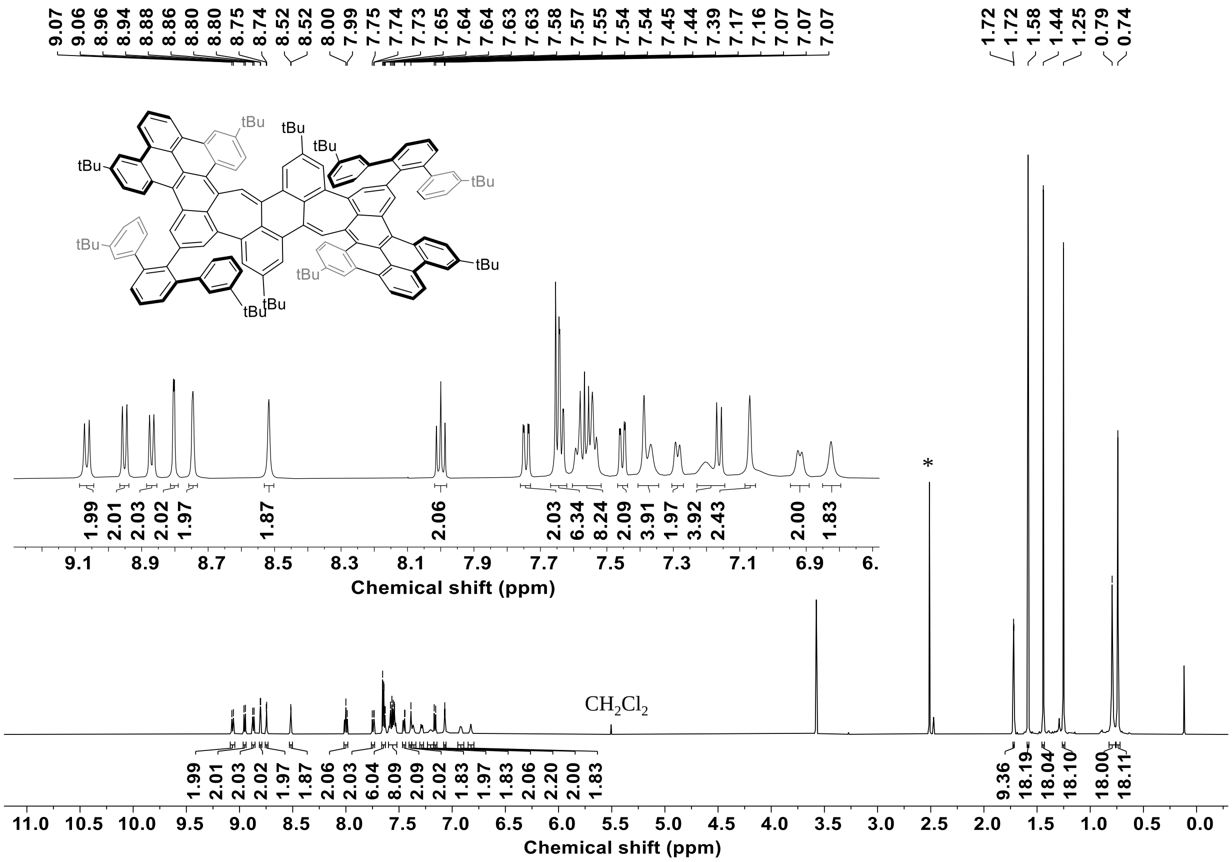

Figure S29. ^{1}H NMR spectrum (600 MHz, THF-d_8, 298 K) of compound **8**. * H_2O.

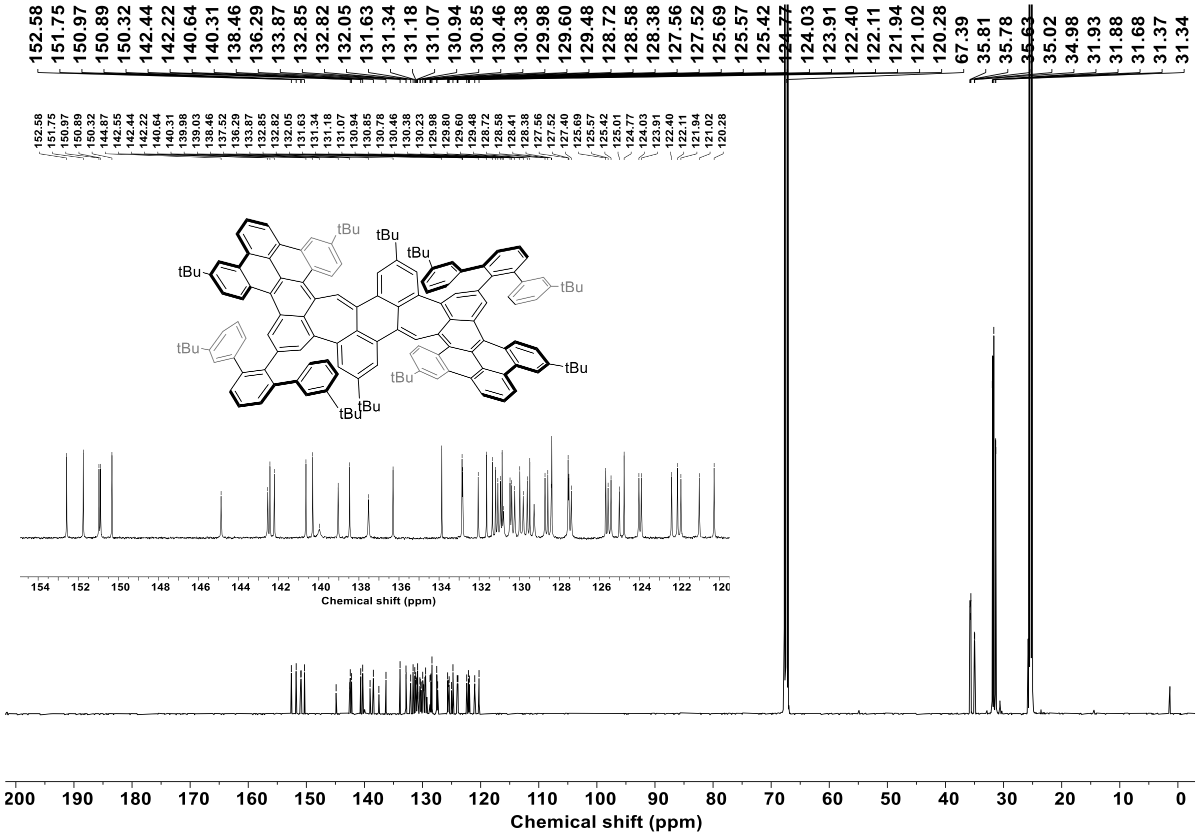

Figure S30. ^{13}C NMR spectrum (151 MHz, THF-d_8, 298 K) of compound **8**.

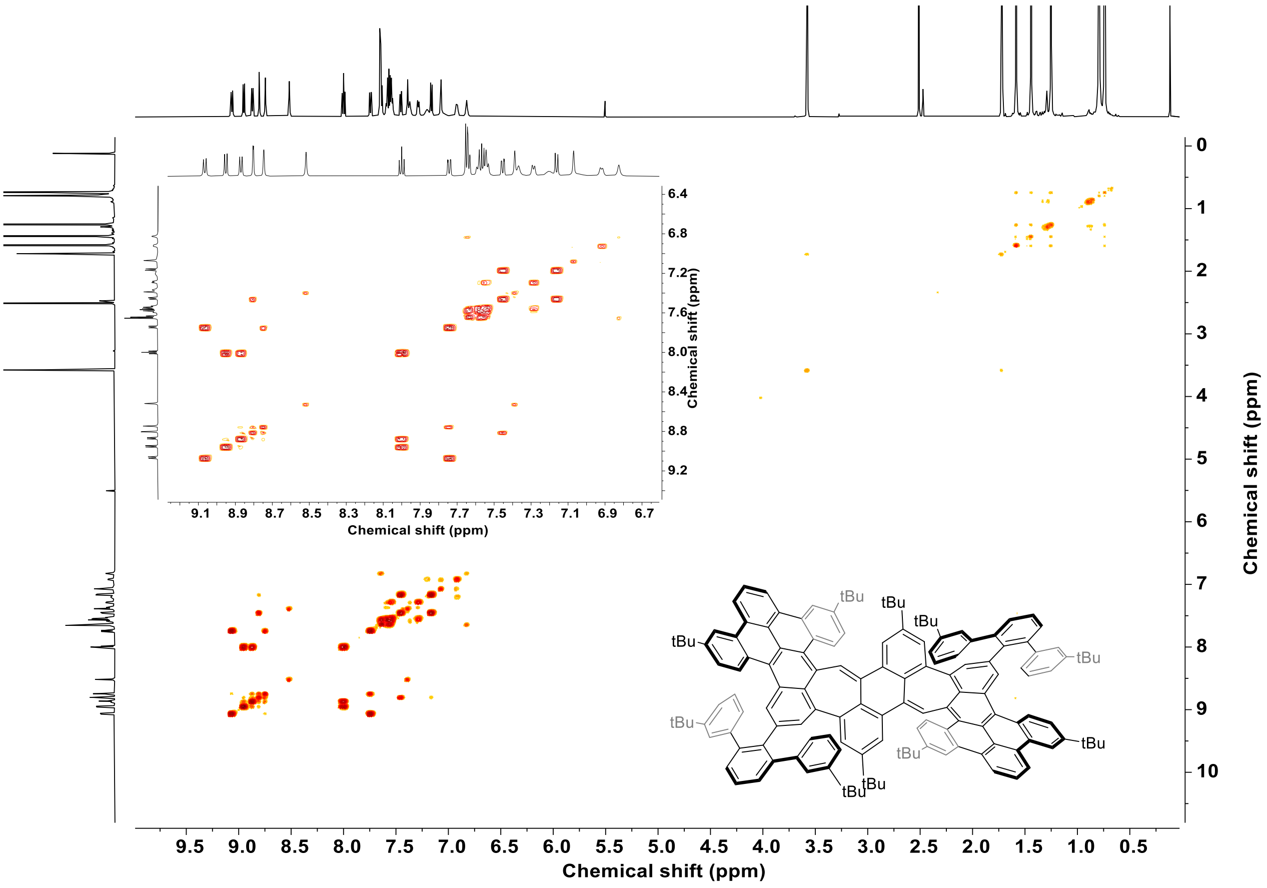

Figure S31. ^{1}H-^{1}H COSY NMR spectrum (600/600 MHz, THF-d_8, 298 K) of compound **8**.

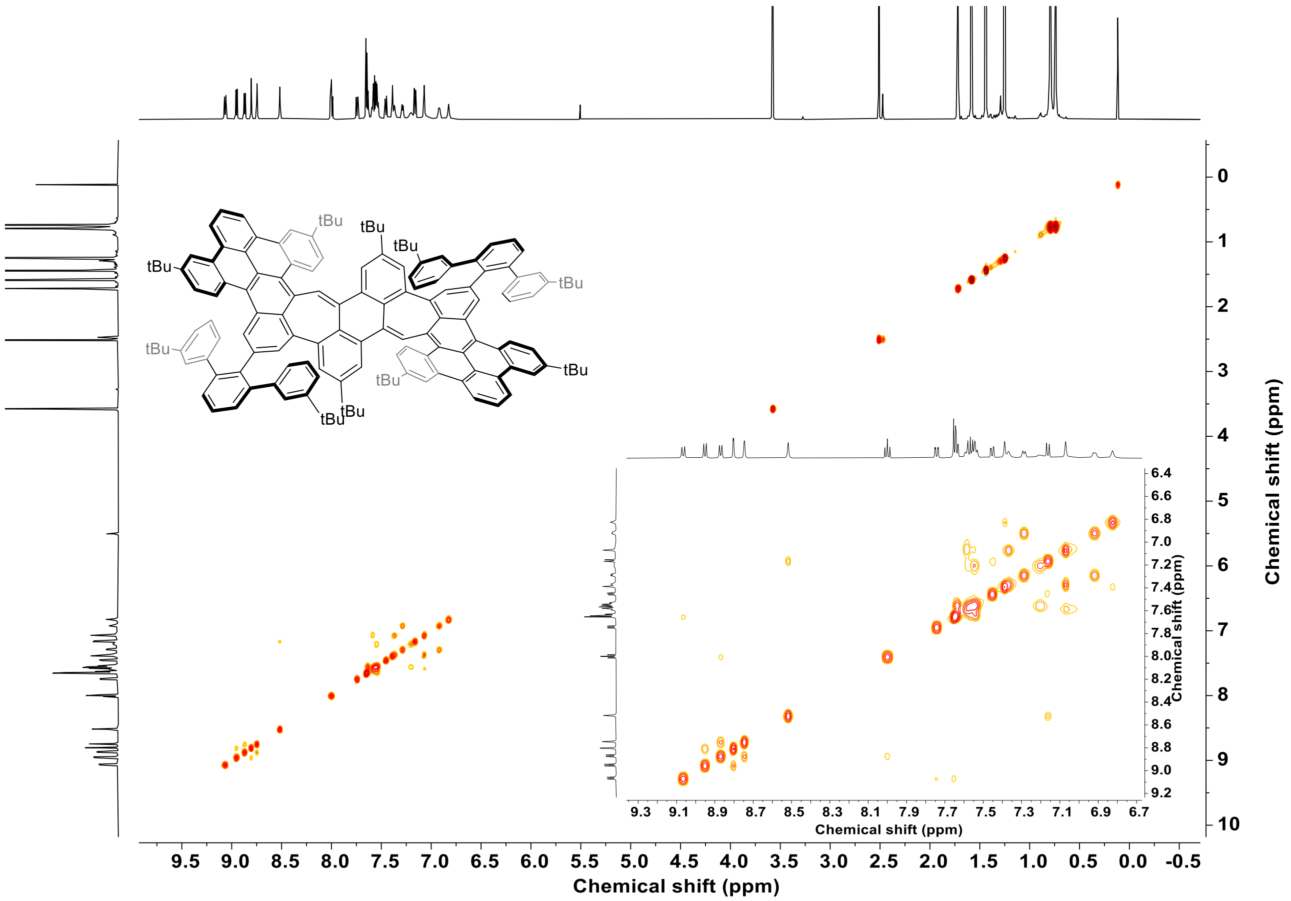

Figure S32. ^1H-^1H NOESY NMR spectrum (600/600 MHz, THF-d_8, 298 K) of compound **8**.

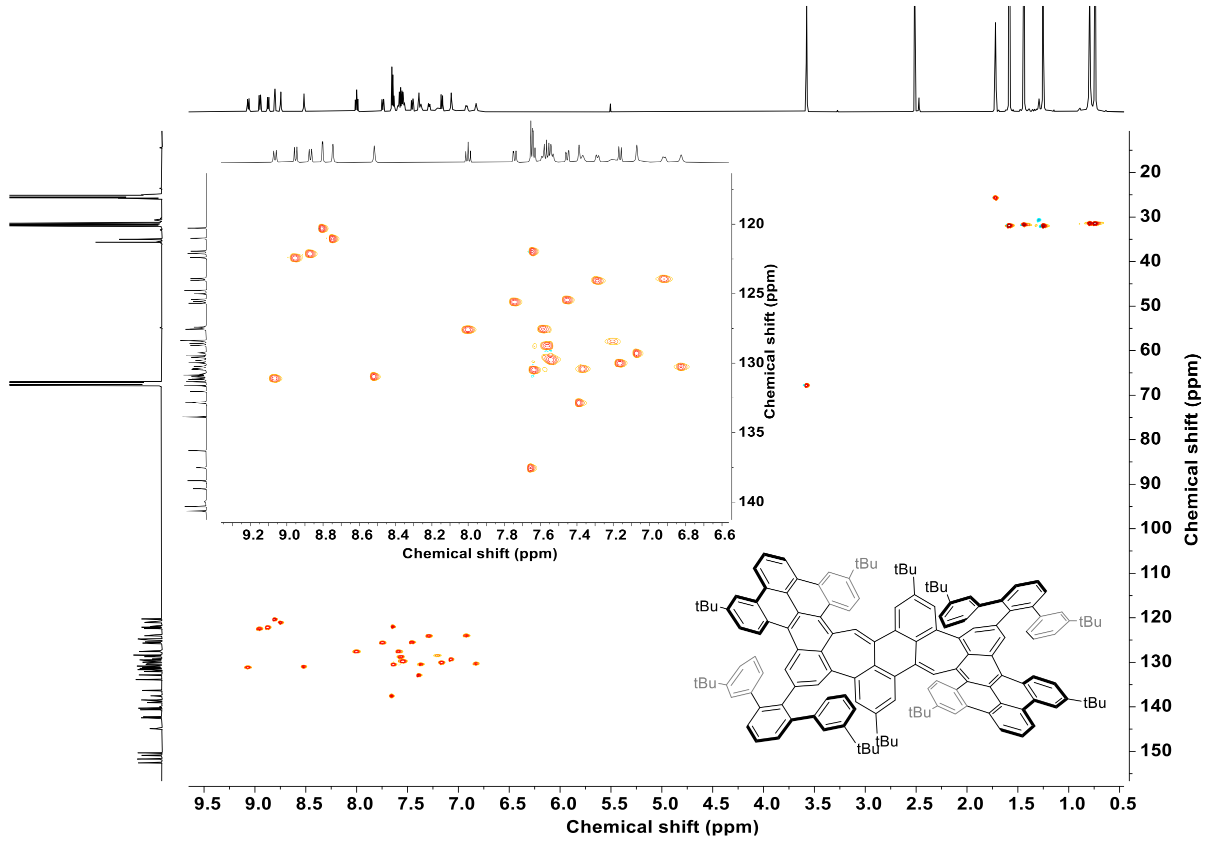

Figure S33. ^1H-^{13}C HSQC NMR spectrum (600/151 MHz, THF-d_8, 298 K) of compound **8**.

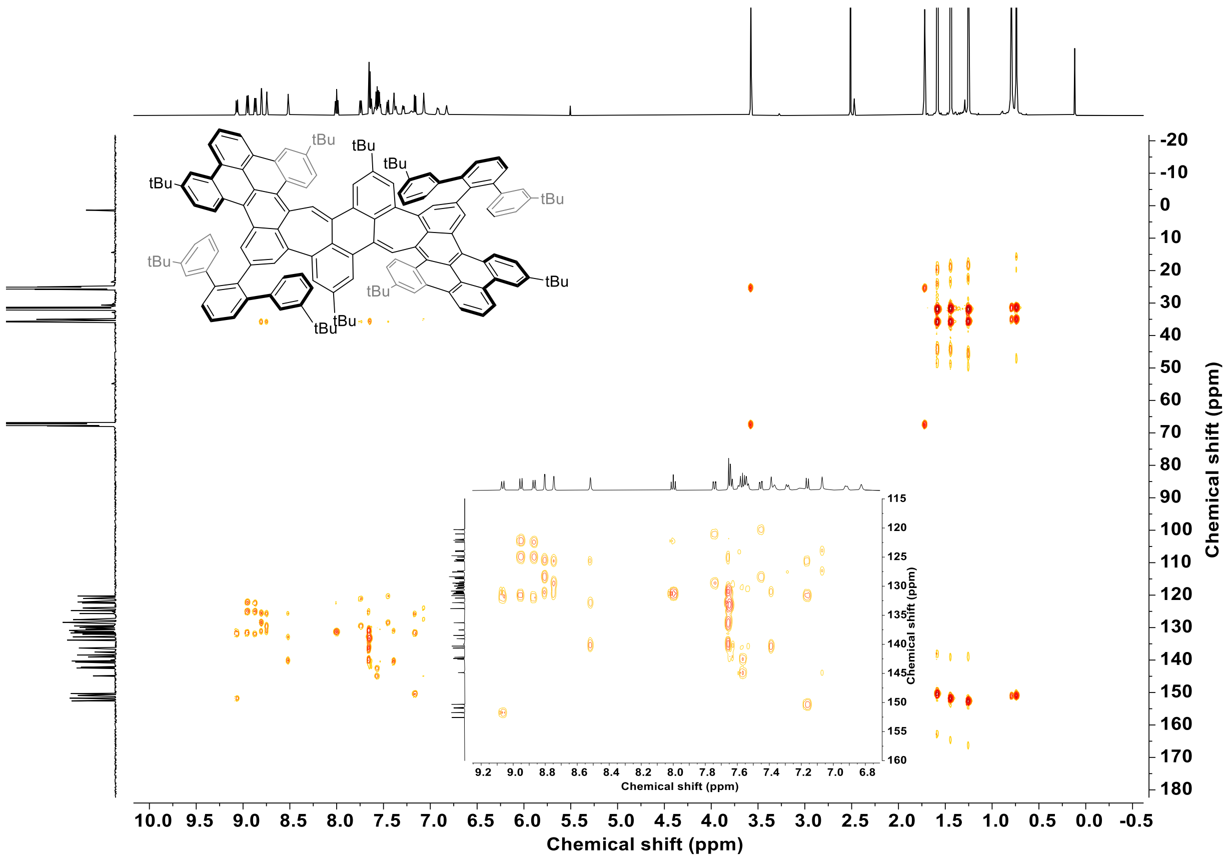

Figure S34. ^{1}H-^{13}C HMBC NMR spectrum (600/151 MHz, THF-d_8, 298 K) of compound **8**.

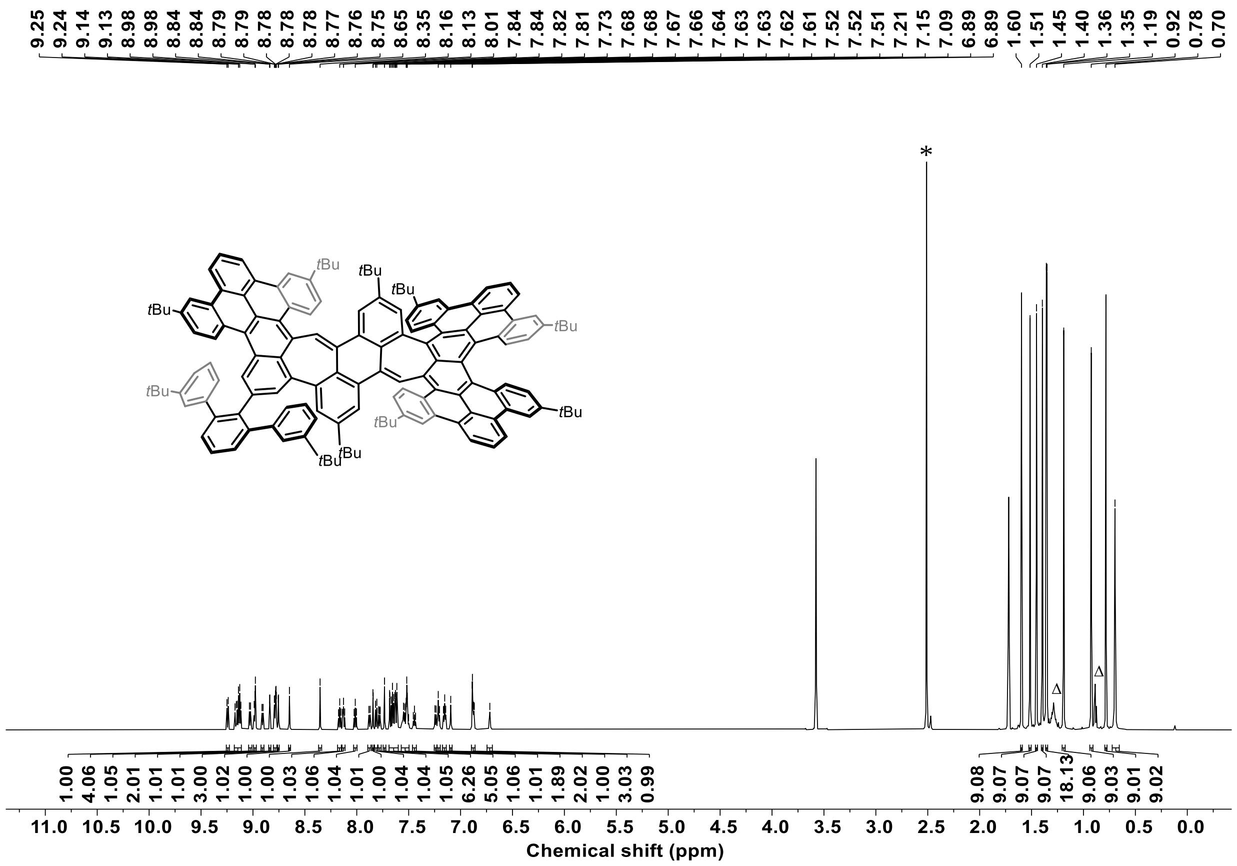

Figure S35. ^{1}H NMR spectrum (600 MHz, THF-d_8, 298 K) of compound **9**. *: H_2O. Δ: heptane.

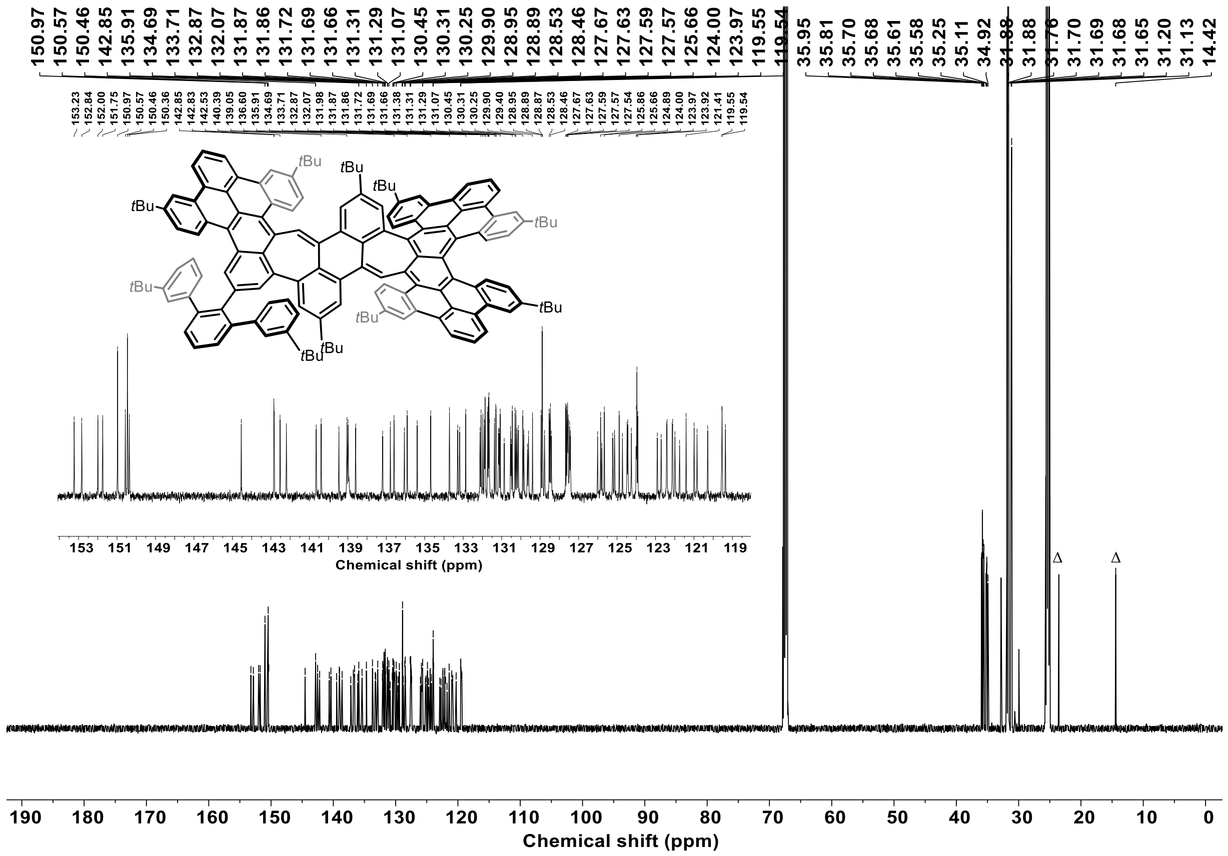

Figure S36. ^{13}C NMR spectrum (151 MHz, THF-d_8, 298 K) of compound **9**. Δ: heptane.

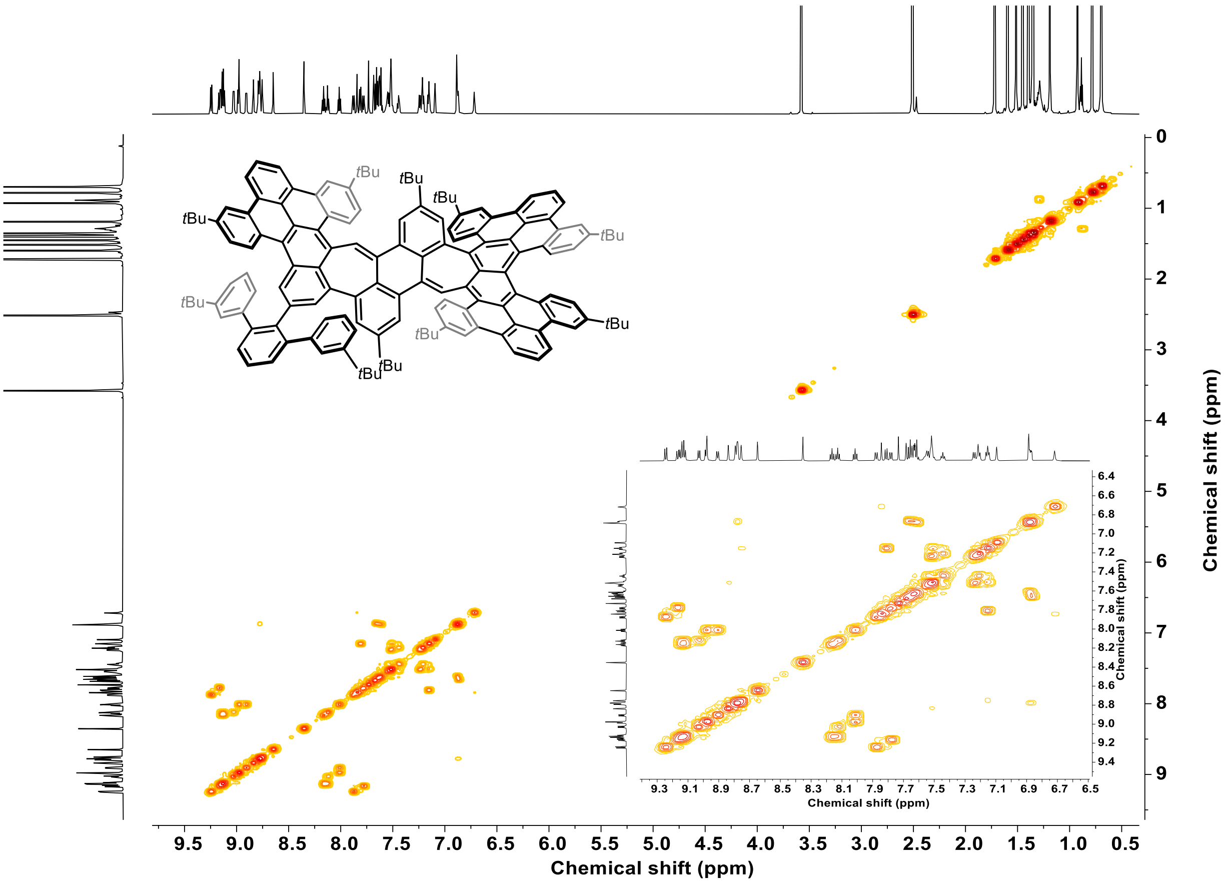

Figure S37. ^1H-^1H COSY NMR spectrum (600/600 MHz, THF-d_8, 298 K) of compound **9**.

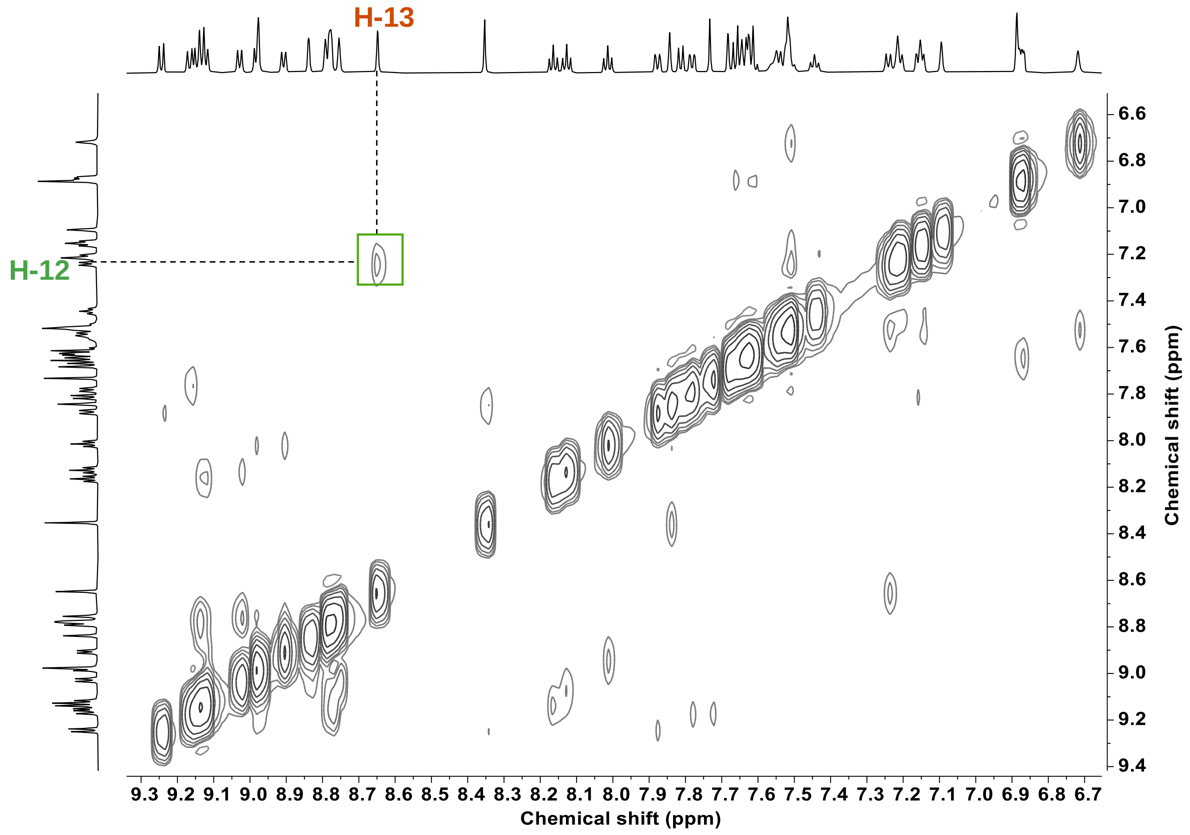

Figure S38. ^1H-^1H NOESY NMR spectrum (600/600 MHz, THF-d_8, 298 K) of compound **9**.

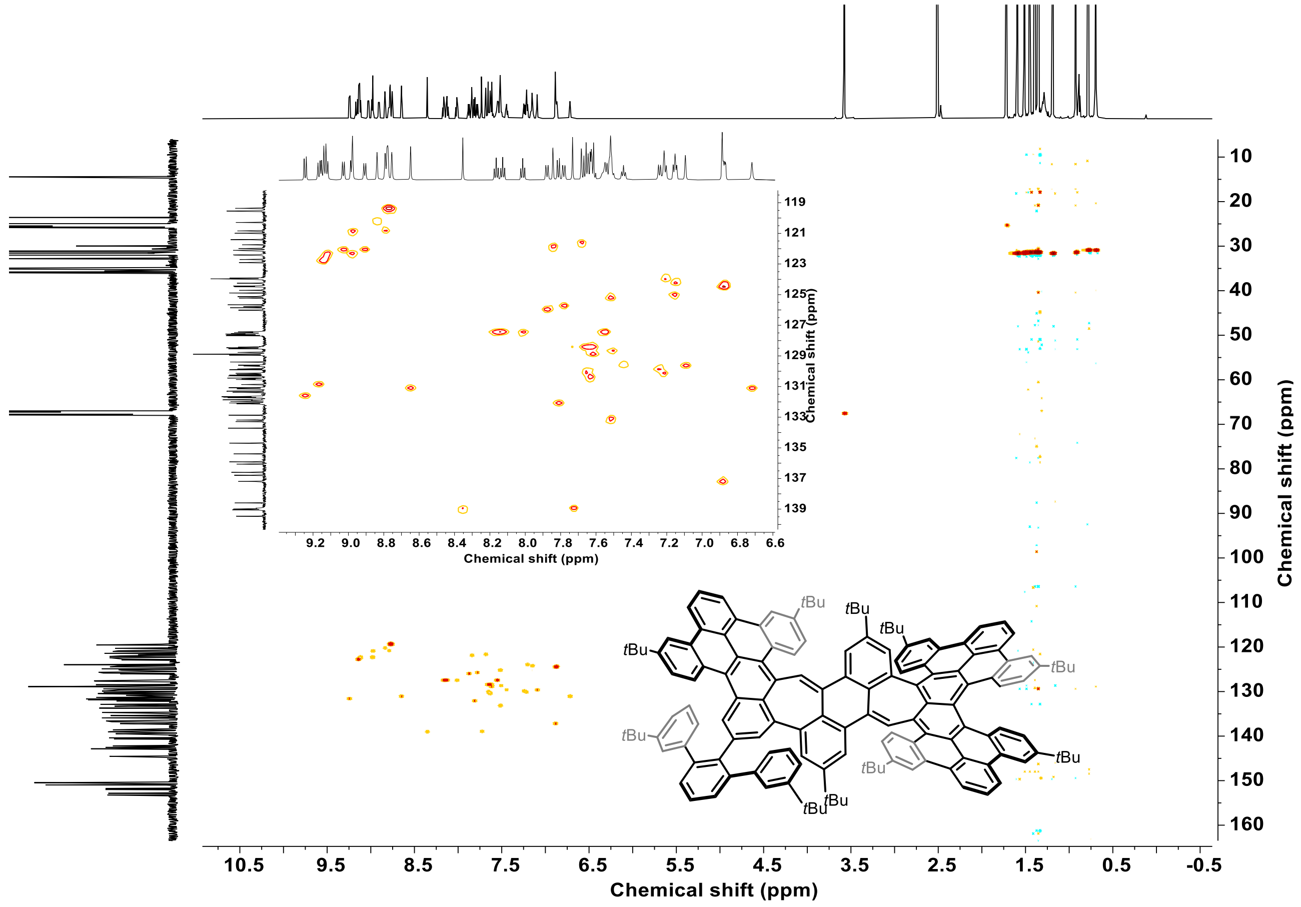

Figure S39. ^1H-^{13}C HSQC NMR spectrum (600/151 MHz, THF-d_8, 298 K) of compound **9**.

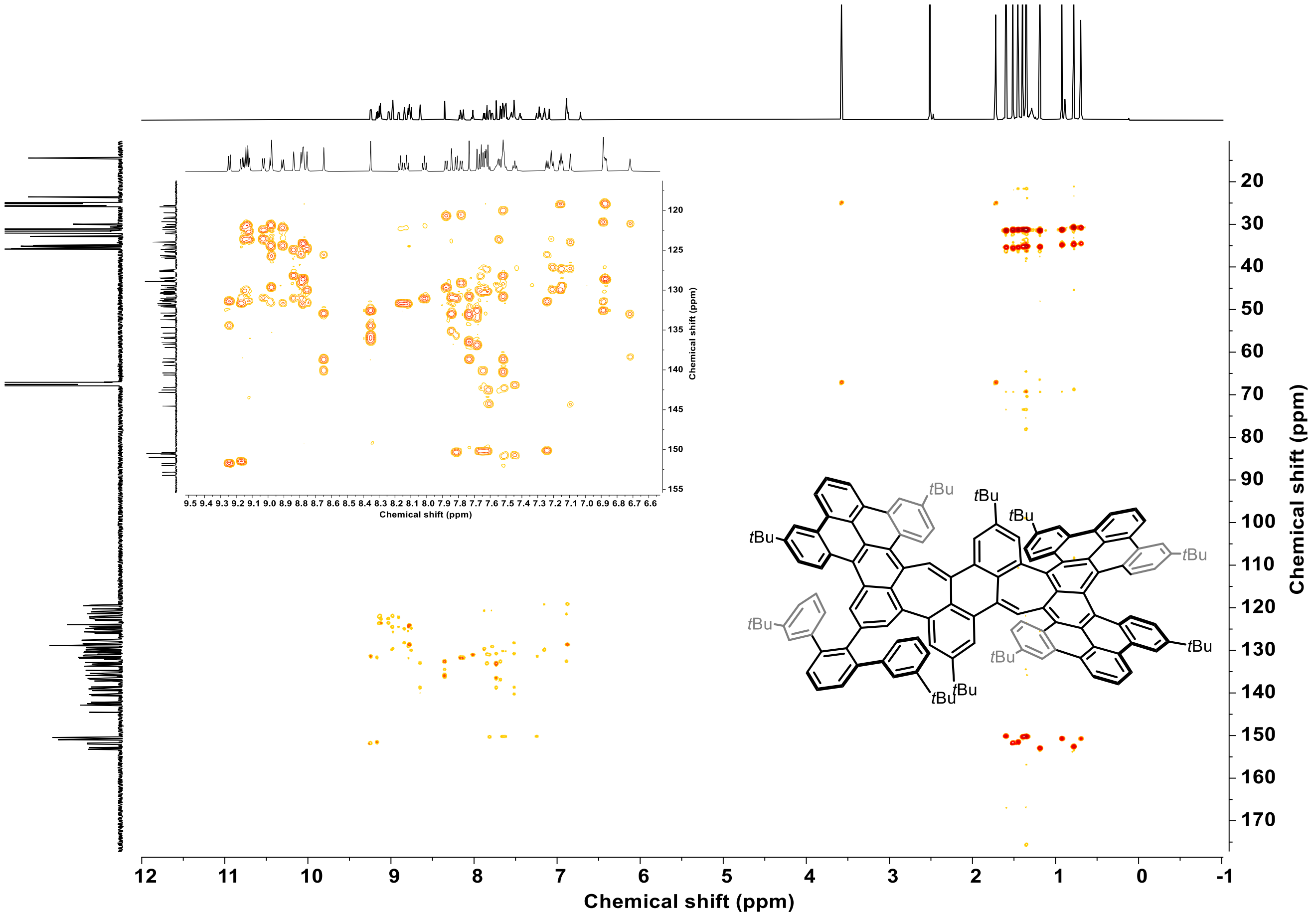

Figure S40. ^{1}H-^{13}C HMBC NMR spectrum (600/151 MHz, THF-d_8, 298 K) of compound **9**.

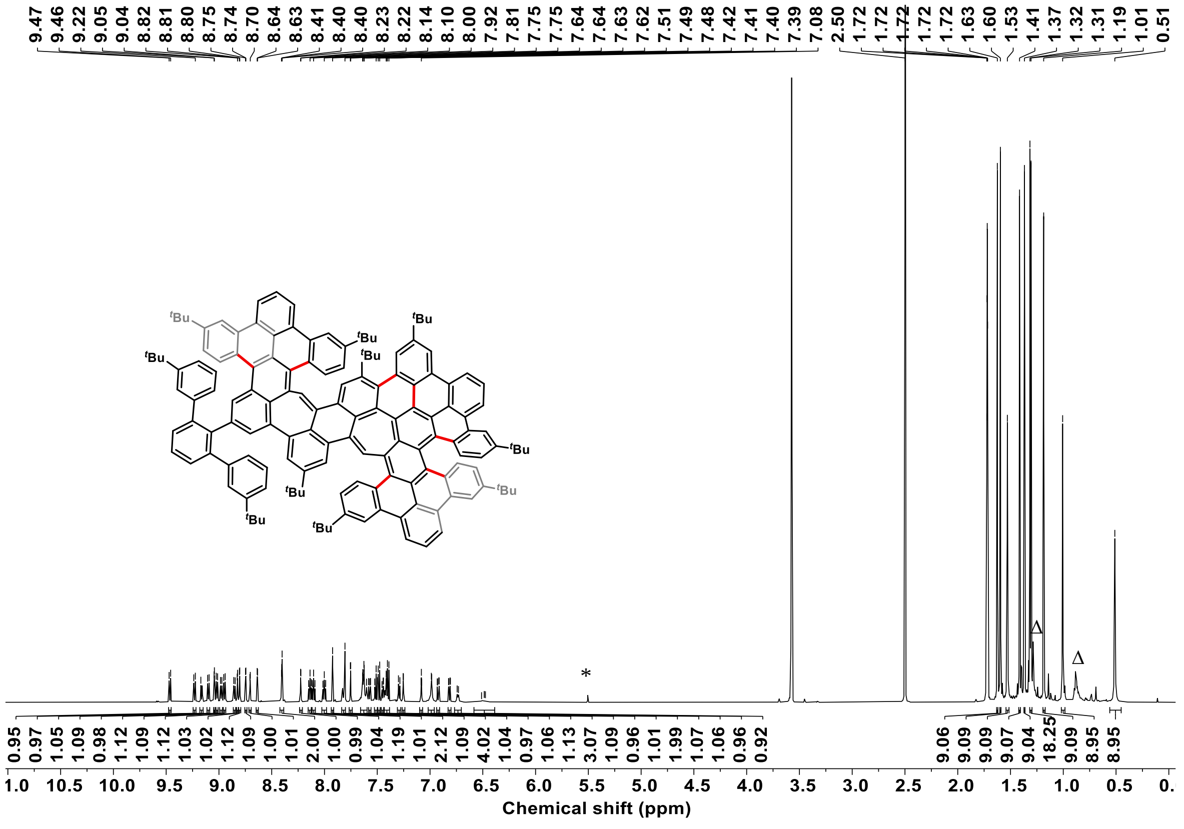

Figure S41. ^{1}H NMR spectrum (600 MHz, THF-d_8, 298 K) of compound **10**. *: CH_2Cl_2, Δ: hexane.

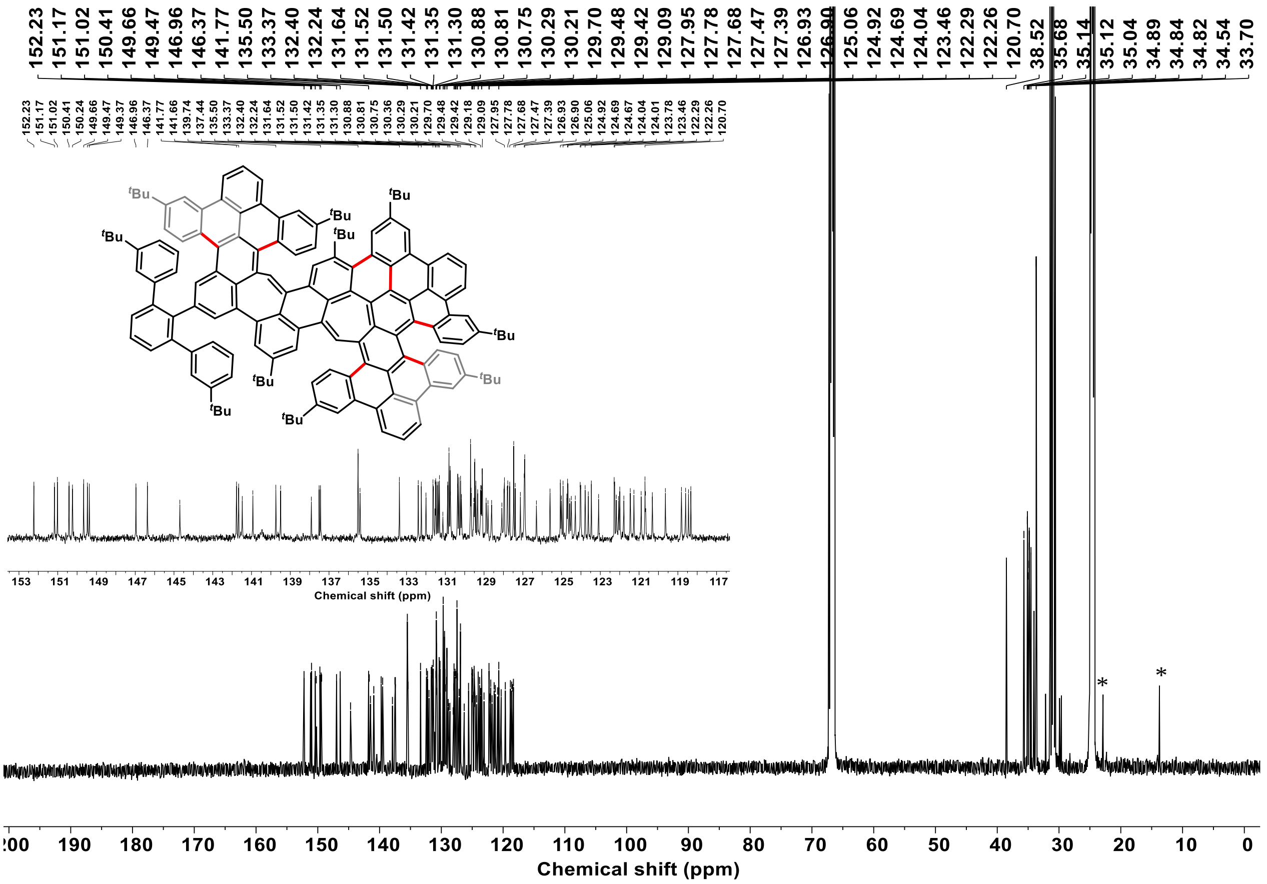

Figure S42. ^{13}C NMR spectrum (151 MHz, THF-d_8, 298 K) of compound **10**. *: hexane.

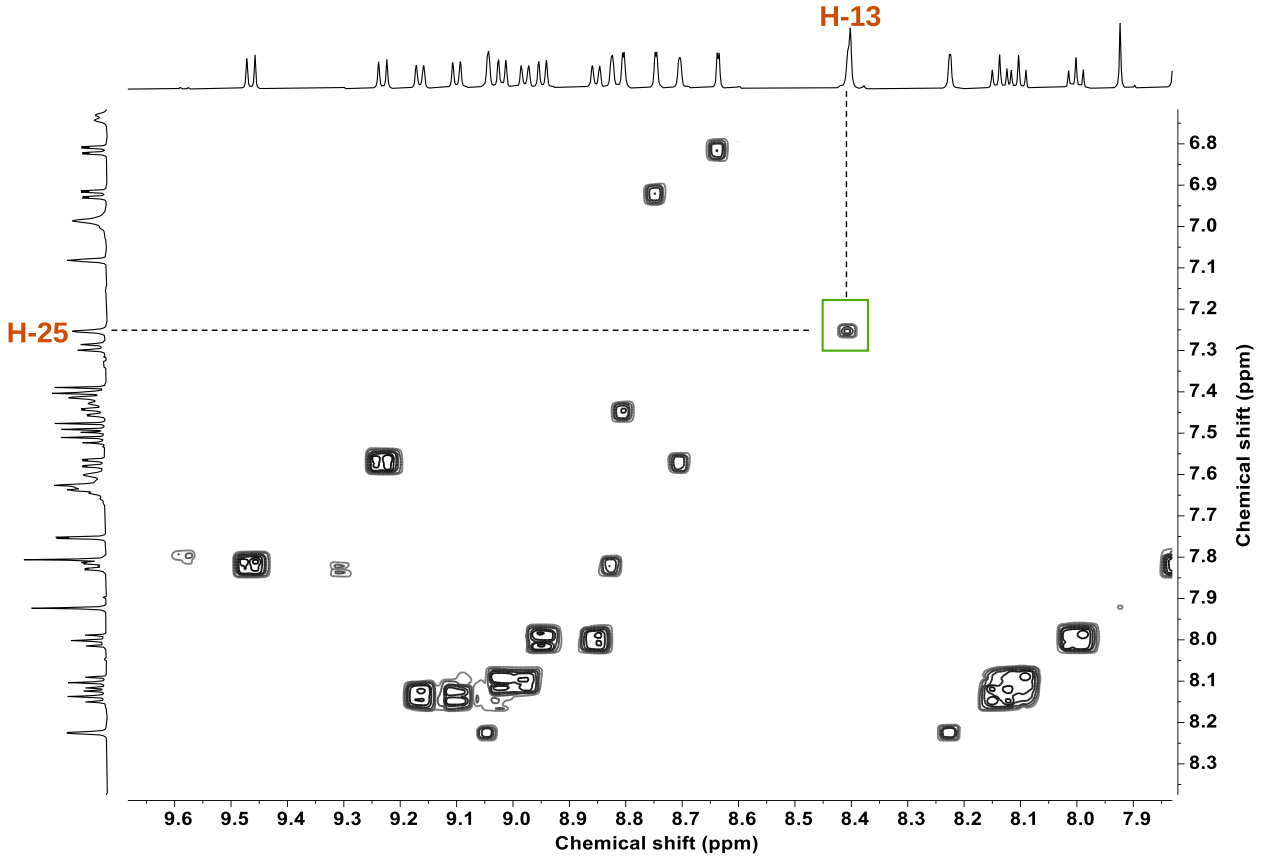

Figure S43. ^{1}H-^{1}H COSY NMR spectrum (600/600 MHz, THF-d_8, 298 K) of compound **10**.

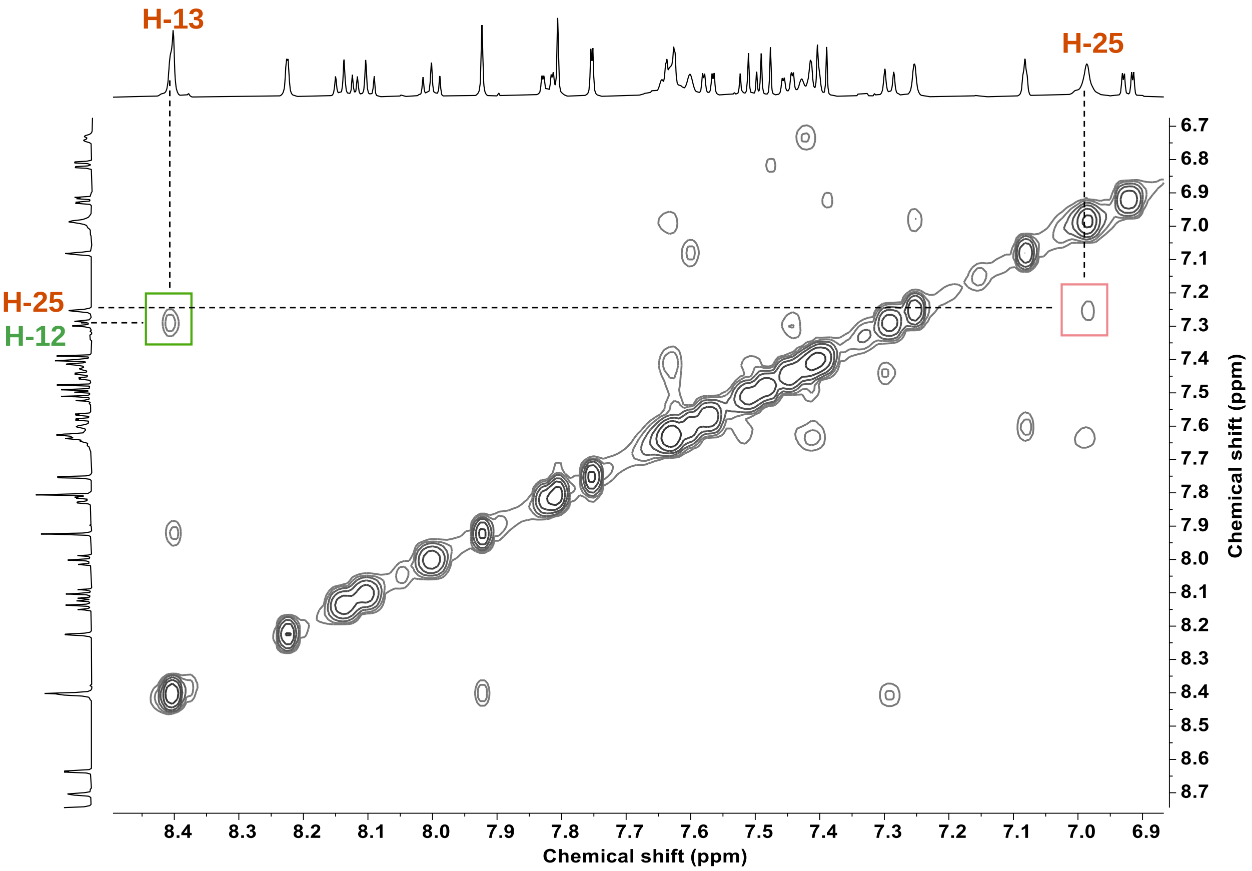

Figure S44. ^{1}H-^{1}H NOESY NMR spectrum (600/600 MHz, THF-d_8, 298 K) of compound **10**.

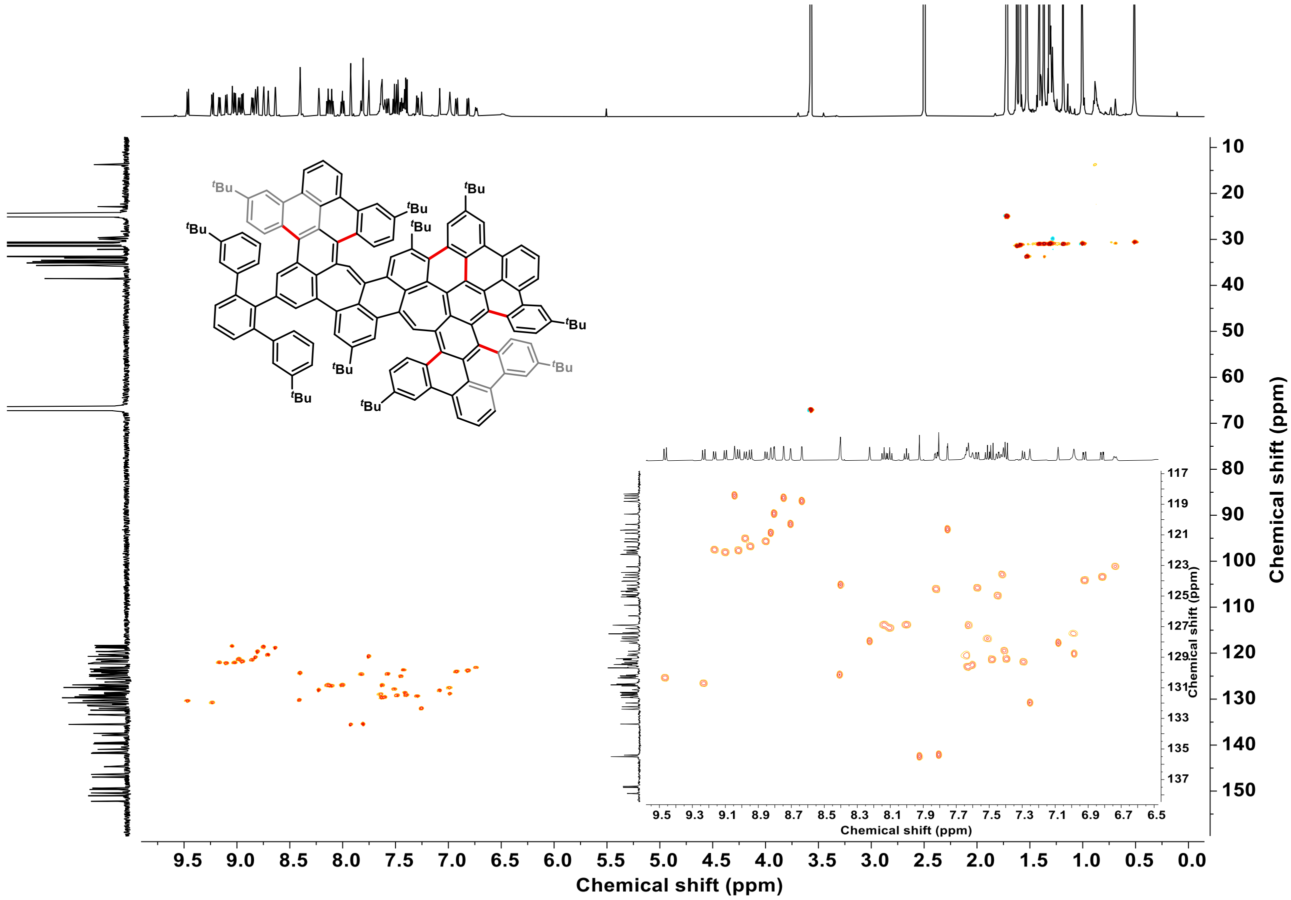

Figure S45. ^{1}H-^{13}C HSQC NMR spectrum (600/151 MHz, THF-d_8, 298 K) of compound **10**.

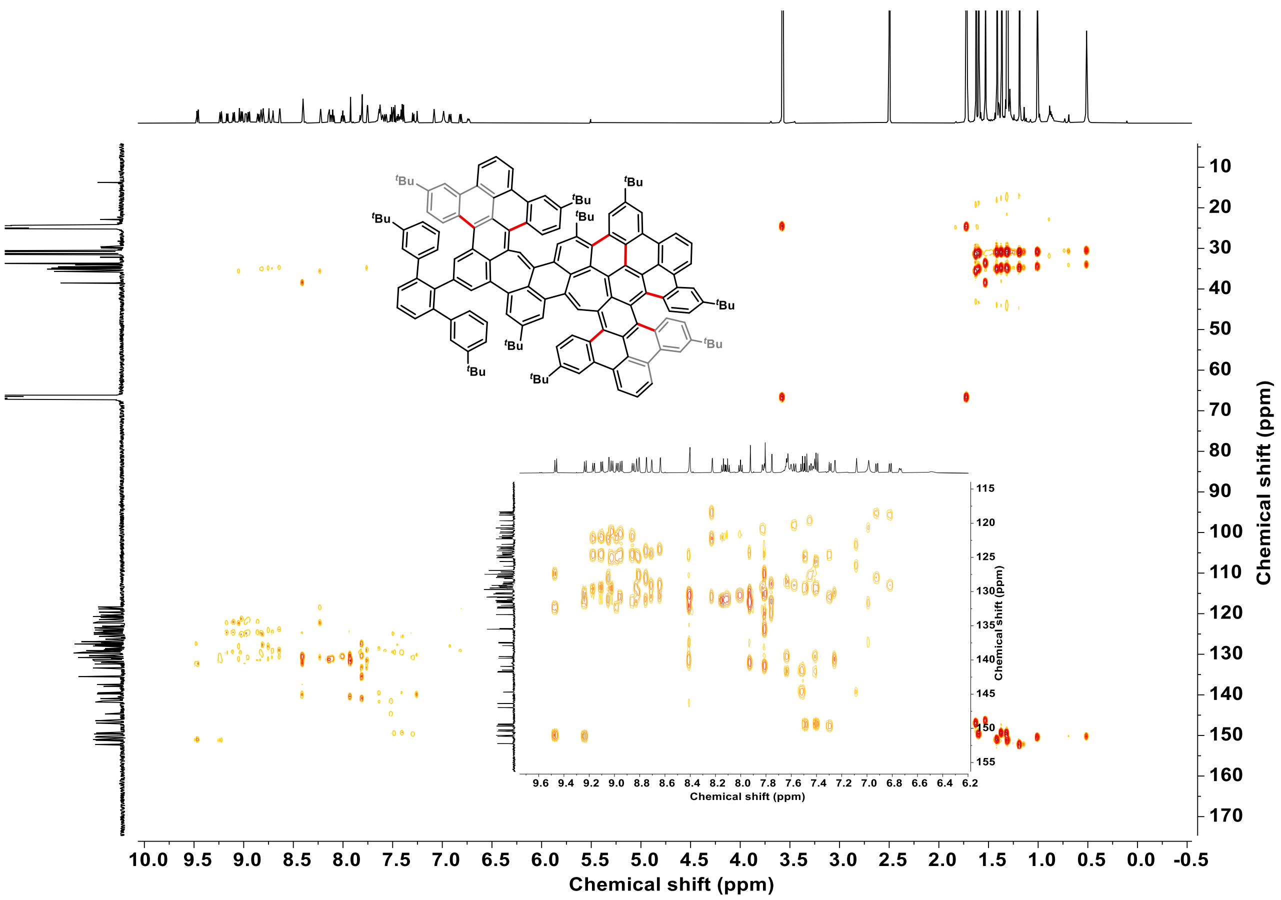

Figure S46. ^{1}H-^{13}C HMBC NMR spectrum (600/151 MHz, THF-d_8, 298 K) of compound **10**.

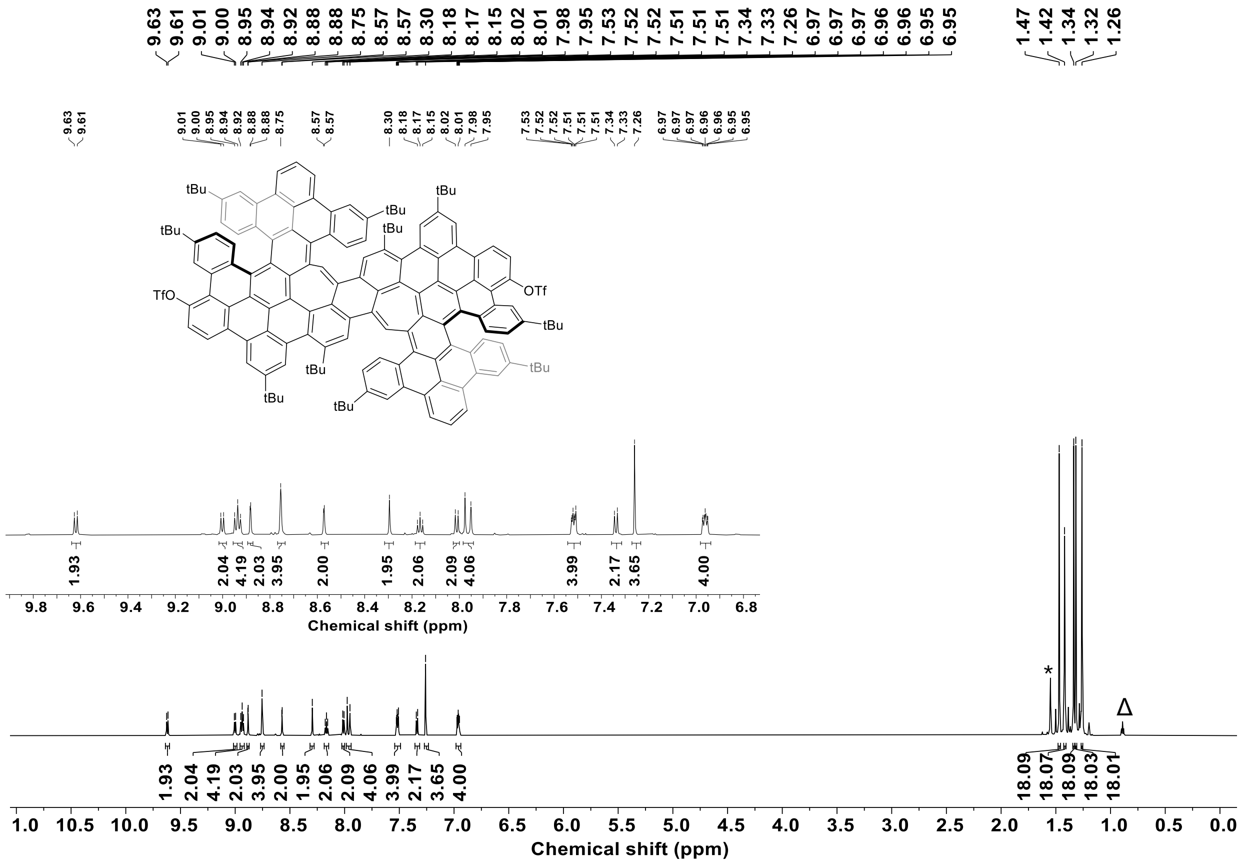

Figure S47. ^{1}H NMR spectrum (600 MHz, $CDCl_3$, 298 K) of compound **11**. *: H_2O, Δ: heptane.

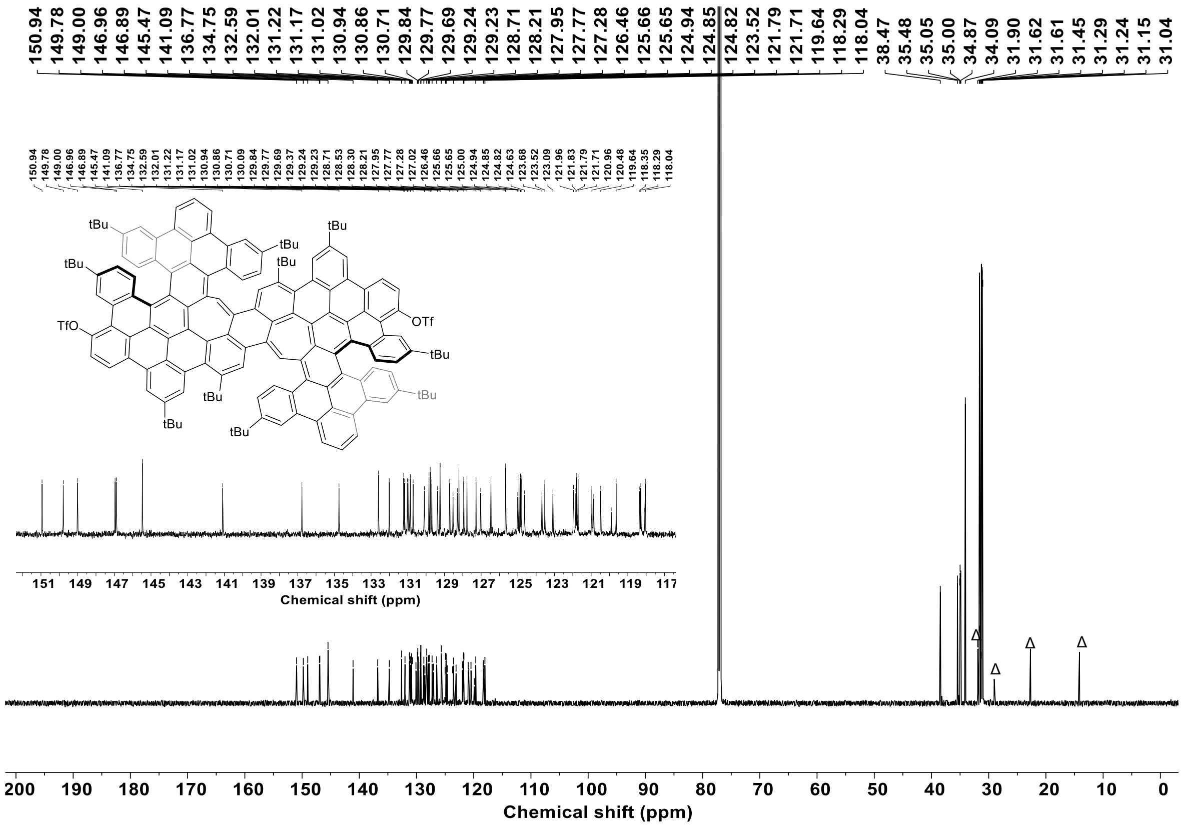

Figure S48. ^{13}C NMR spectrum (151 MHz, $CDCl_3$, 298 K) of compound **11**. Δ: heptane.

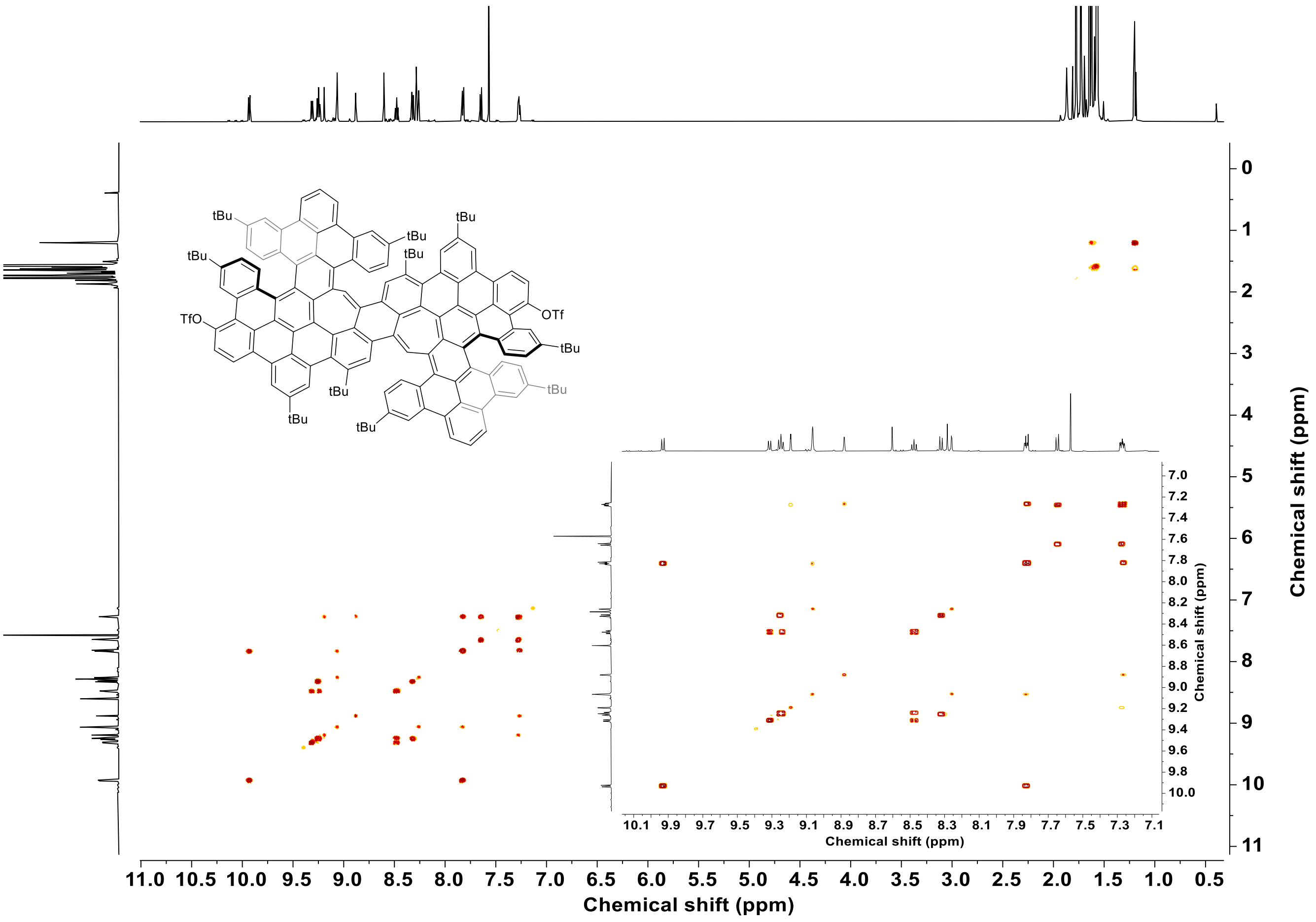

Figure S49. ^1H-^1H COSY NMR spectrum (600/600 MHz, $CDCl_3$, 298 K) of compound **11**.

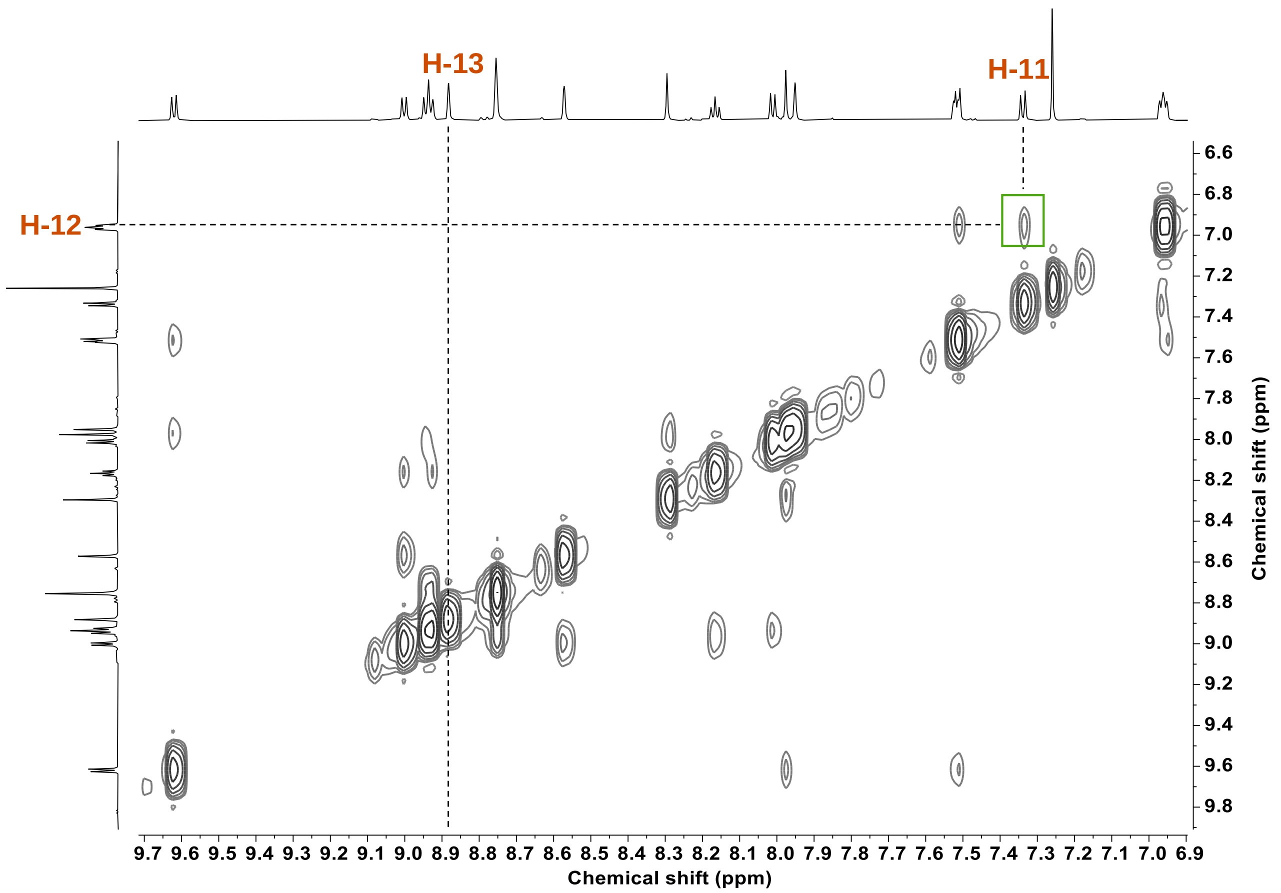

Figure S50. ^{1}H-^{1}H NOESY NMR spectrum (600/600 MHz, $CDCl_3$, 298 K) of compound **11**.

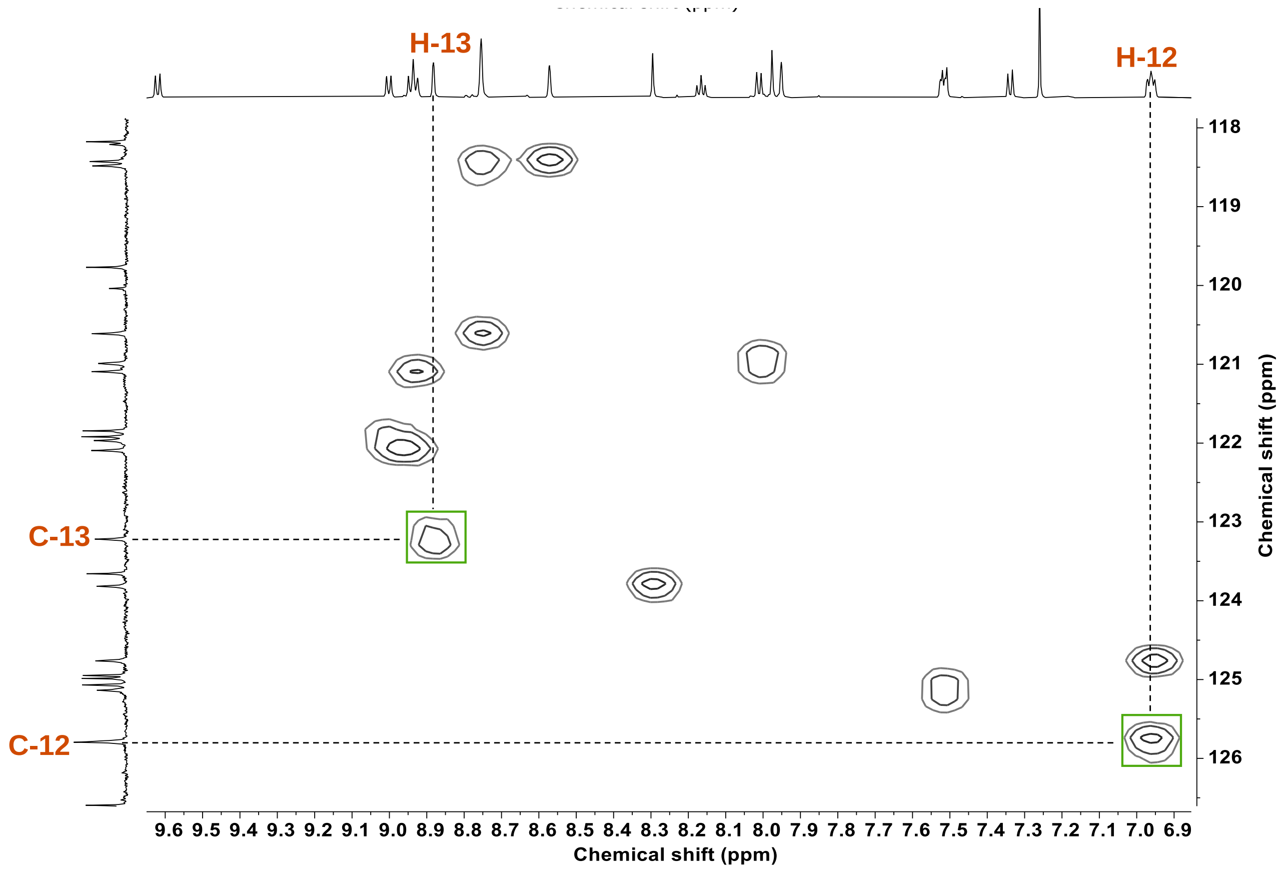

Figure S51. ^{1}H-^{13}C HSQC NMR spectrum (600/151 MHz, $CDCl_3$, 298 K) of compound 11.

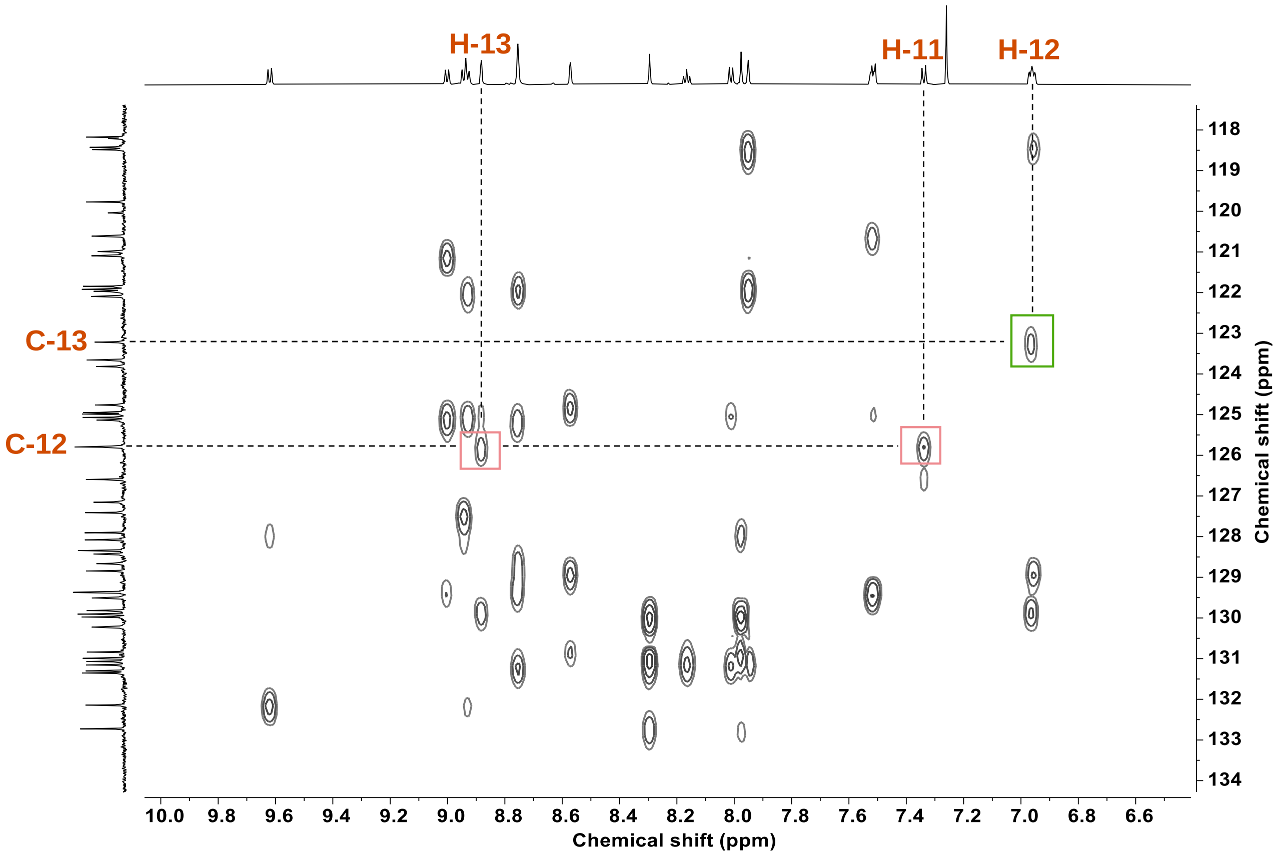

Figure S52. ¹H-¹³C HMBC NMR spectrum (600/151 MHz, $CDCl_3$, 298 K) of compound **11**.

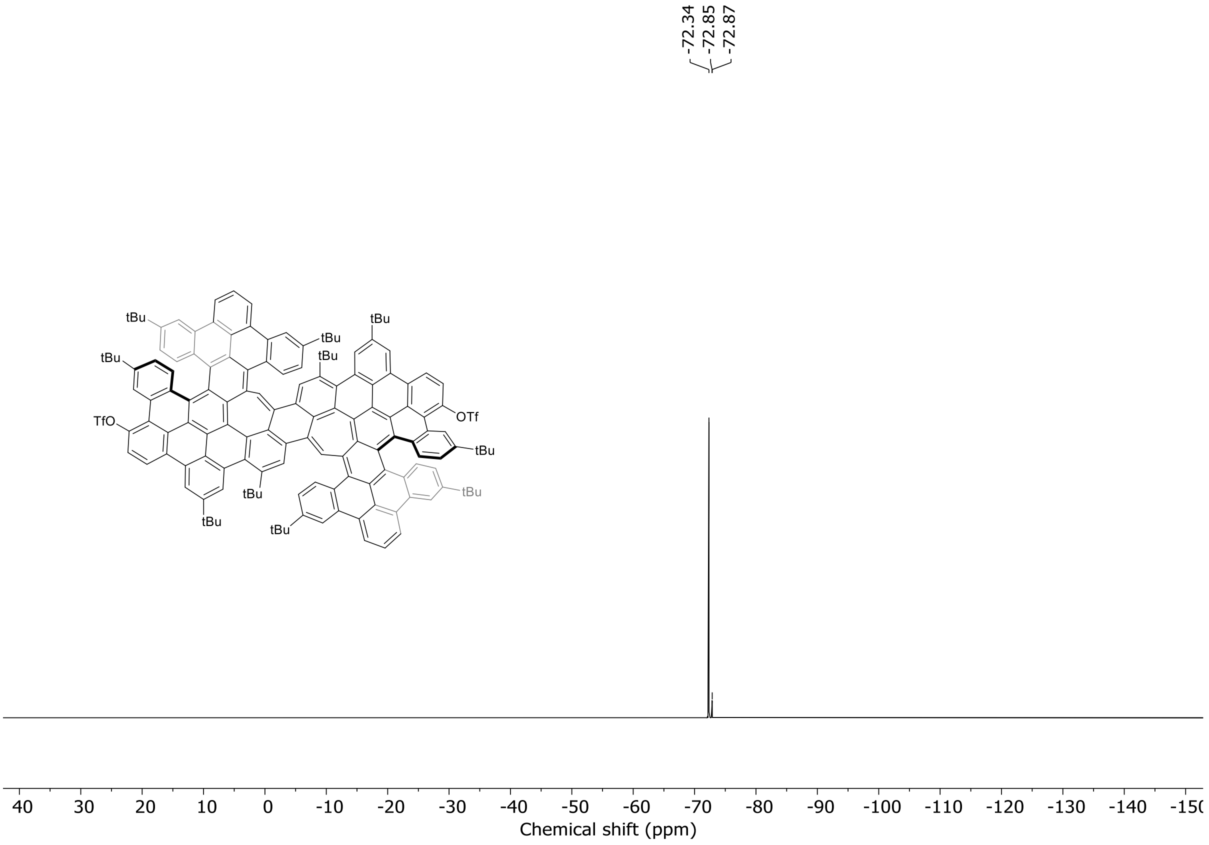

Figure S53. ¹⁹F NMR (471 MHz, $CDCl_3$, 298 K) of compound **11.**

4. FT-IR Spectra

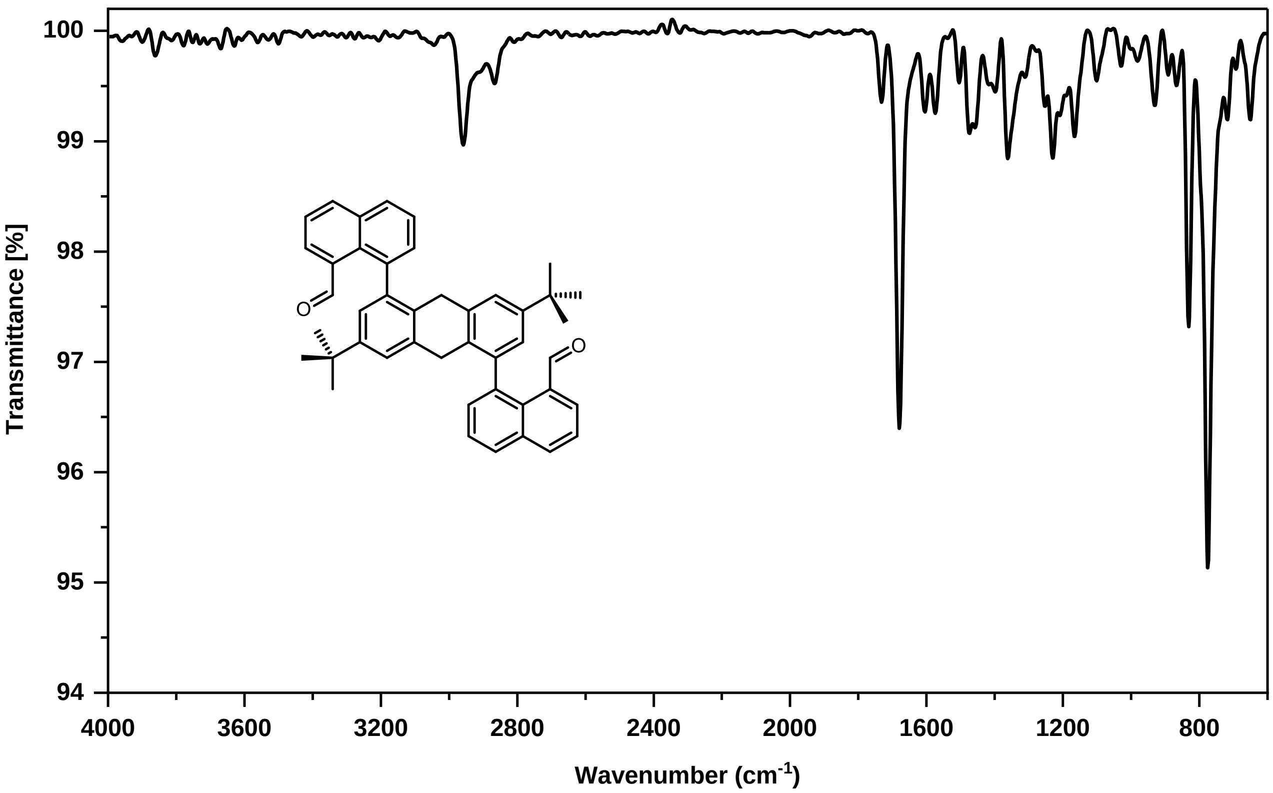

Figure S54. FT-IR spectrum (ATR, neat) of compound **4**.

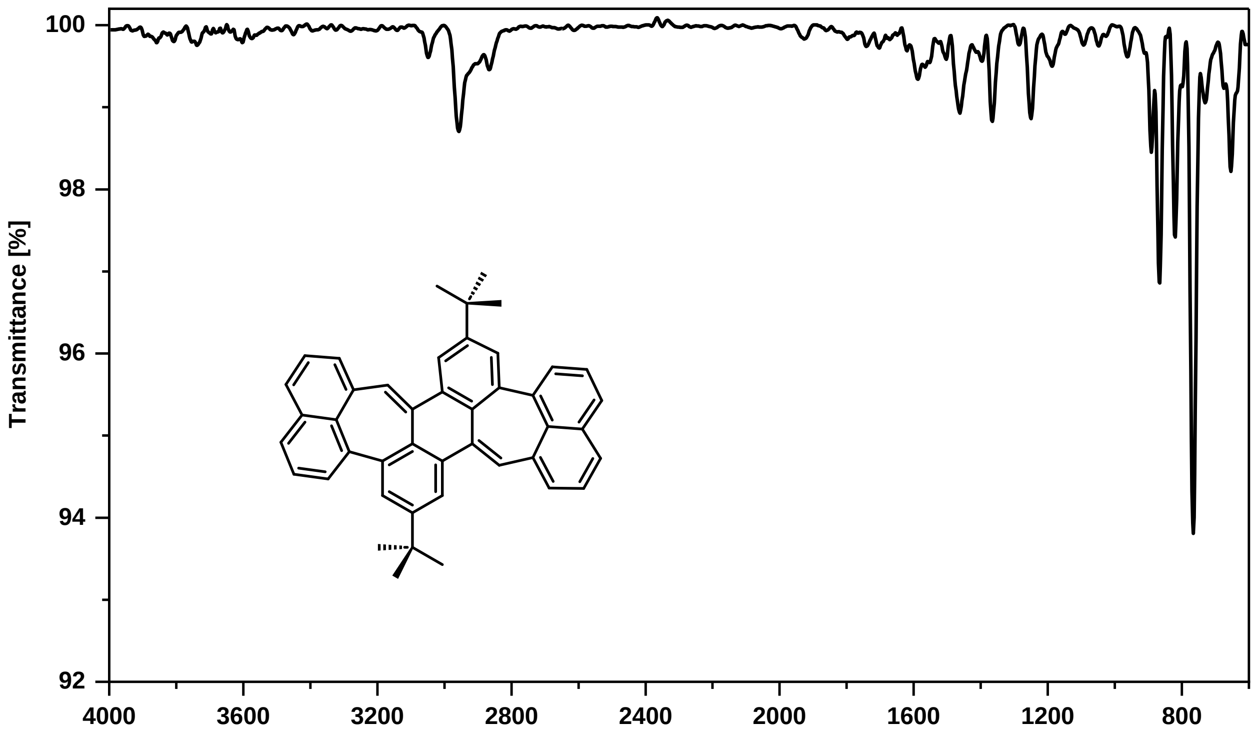

Figure S55. FT-IR spectrum (ATR, neat) of compound **5**.

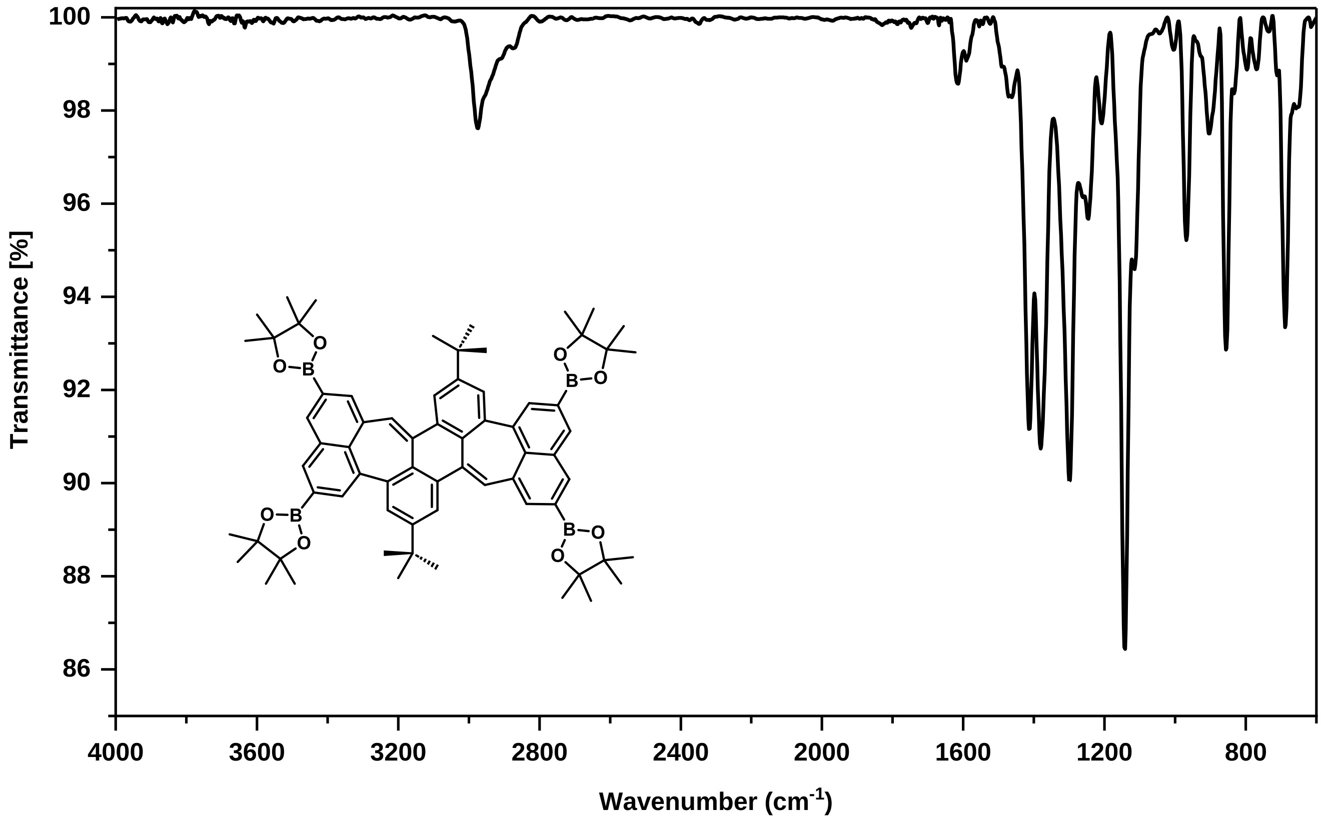

Figure S56. FT-IR spectrum (ATR, neat) of compound **6**.

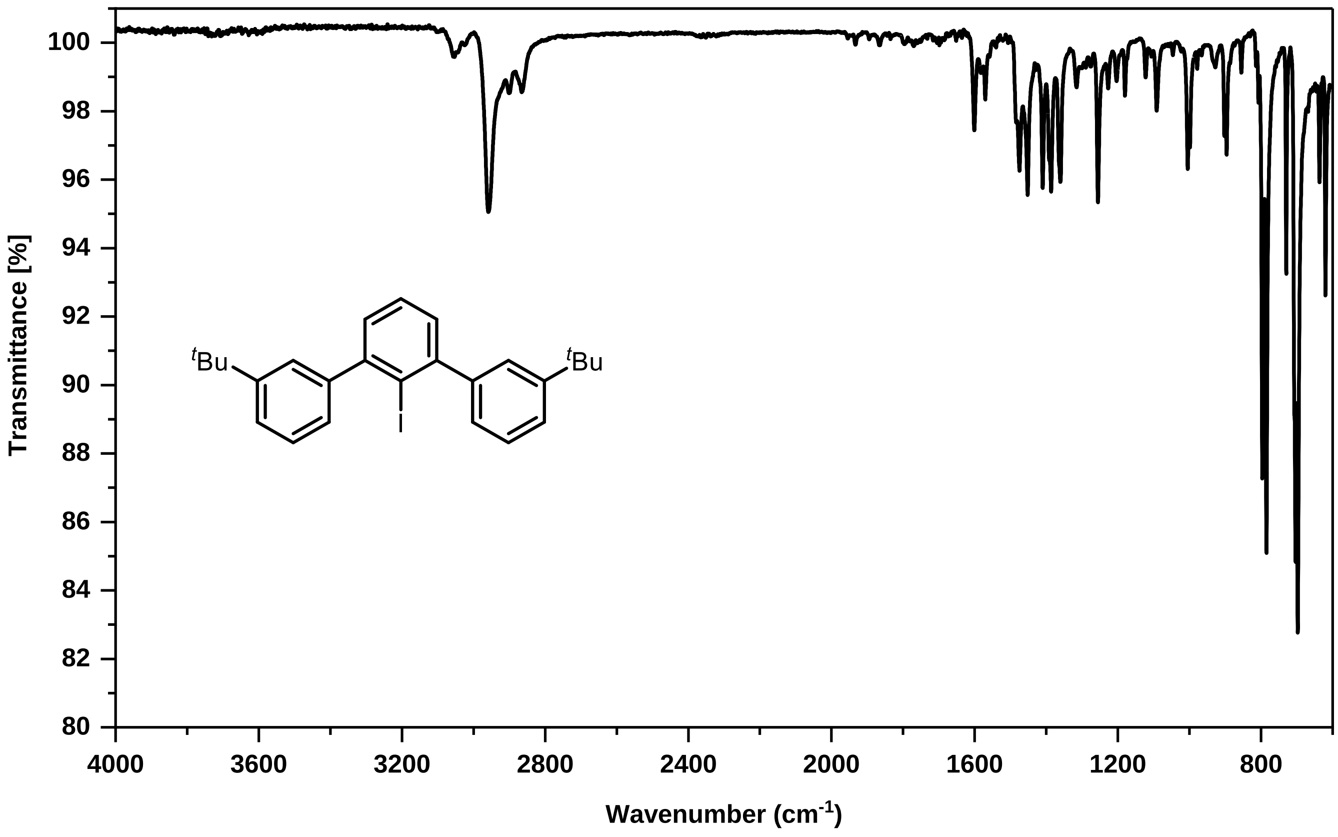

Figure S57. FT-IR spectrum (ATR, neat) of compound **6**.

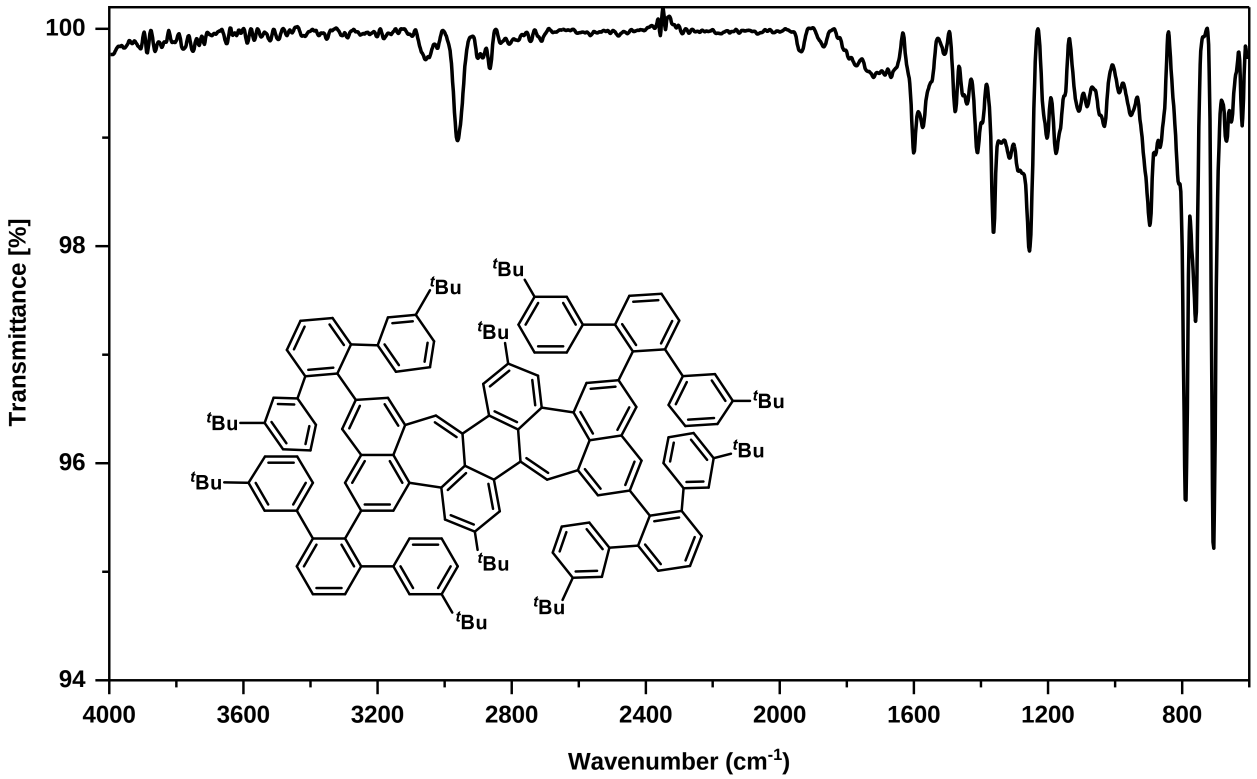

Figure S58. FT-IR spectrum (ATR, neat) of compound **7**.

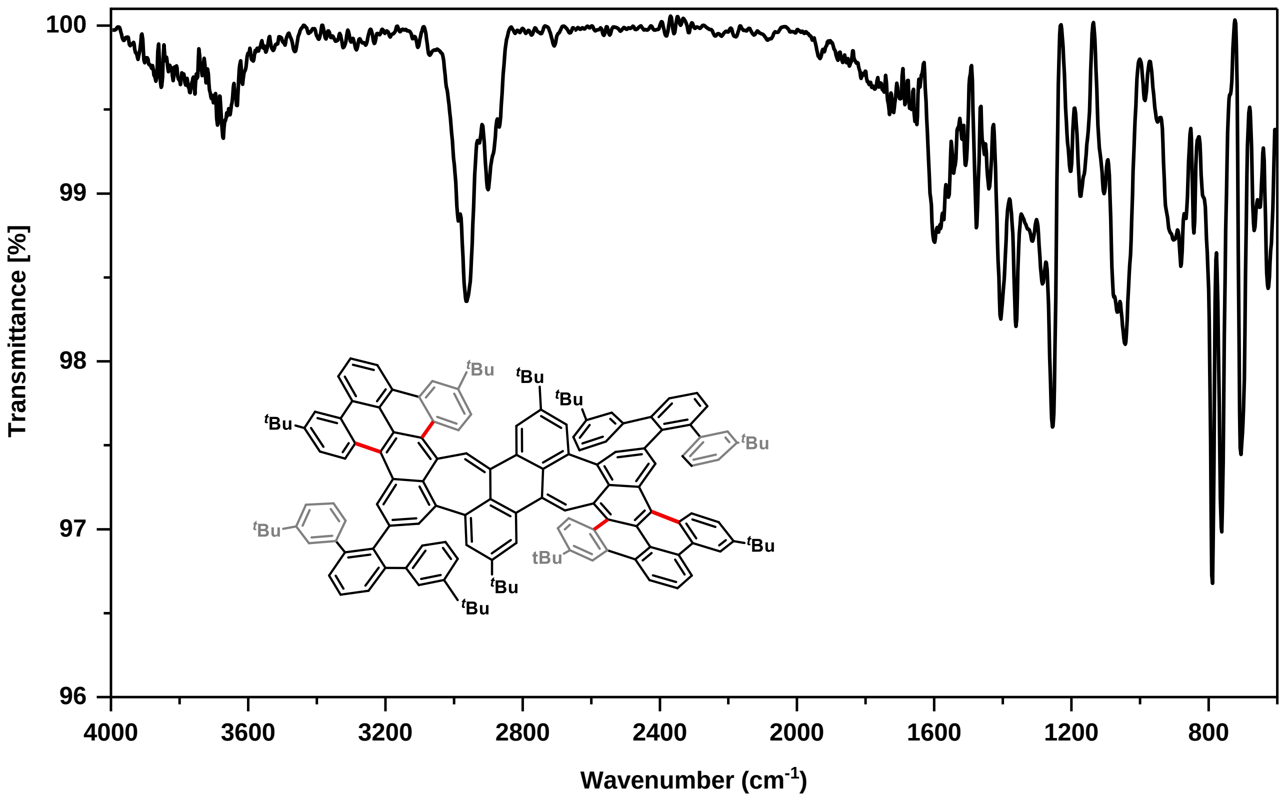

Figure S59. FT-IR spectrum (ATR, neat) of compound **8**.

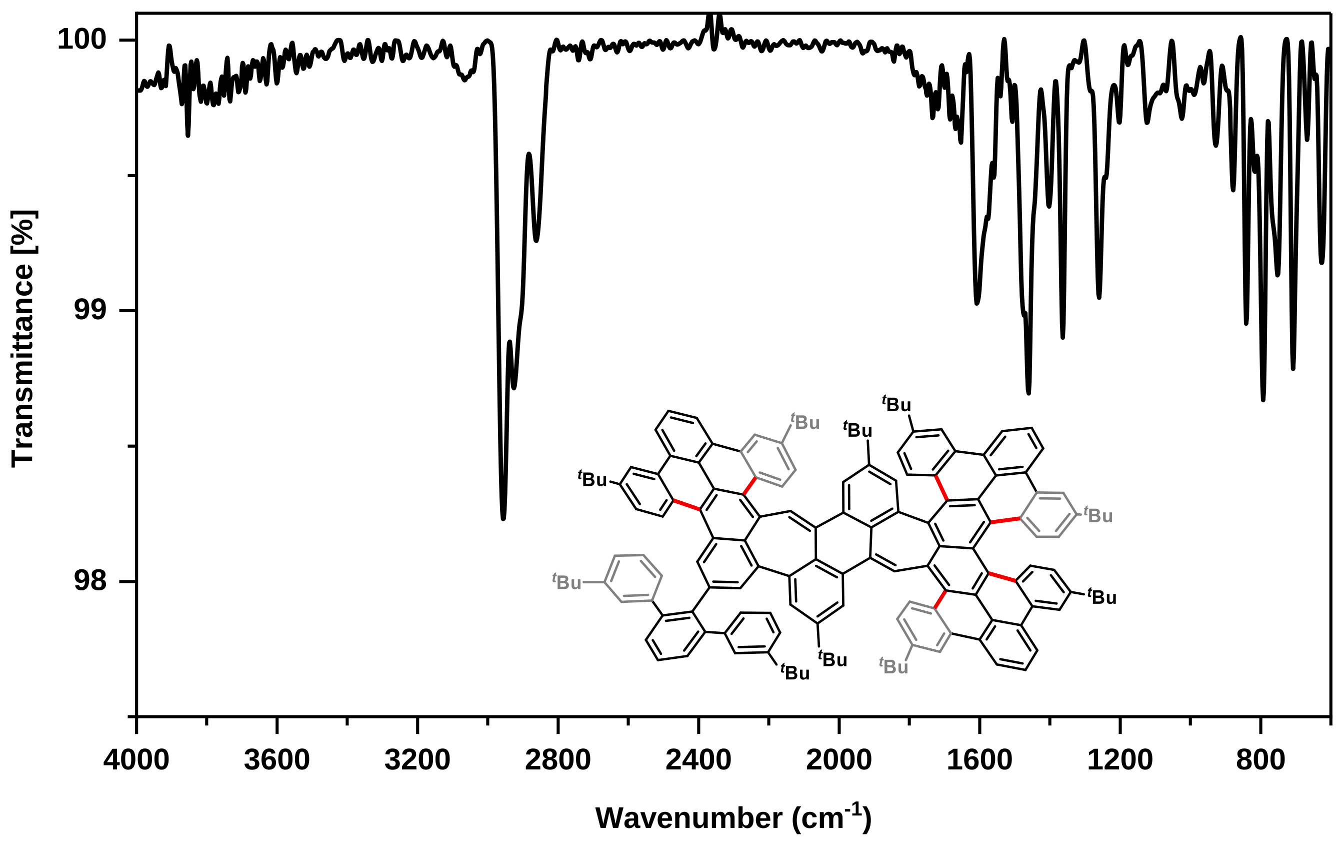

Figure S60. FT-IR spectrum (ATR, neat) of compound **9**.

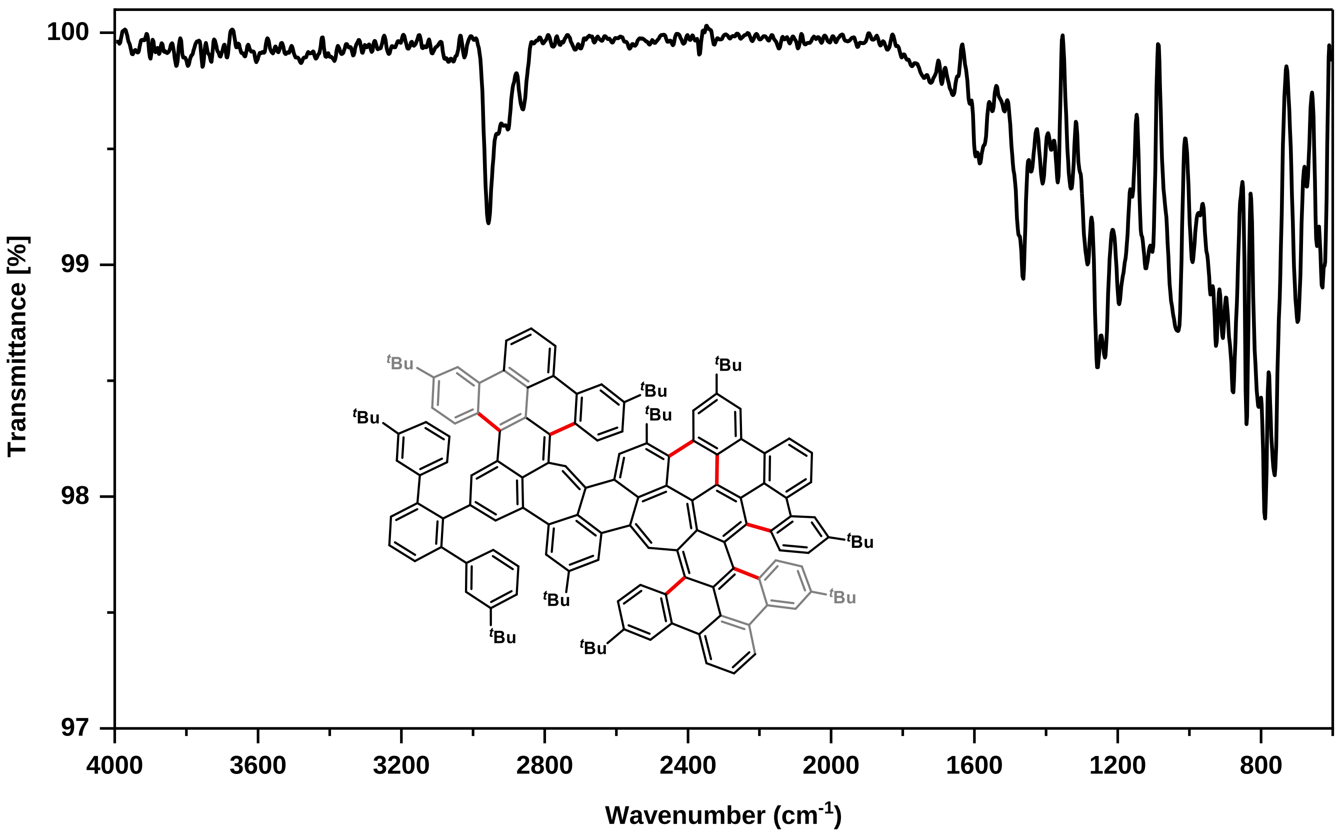

Figure S61. FT-IR spectrum (ATR, neat) of compound **10**.

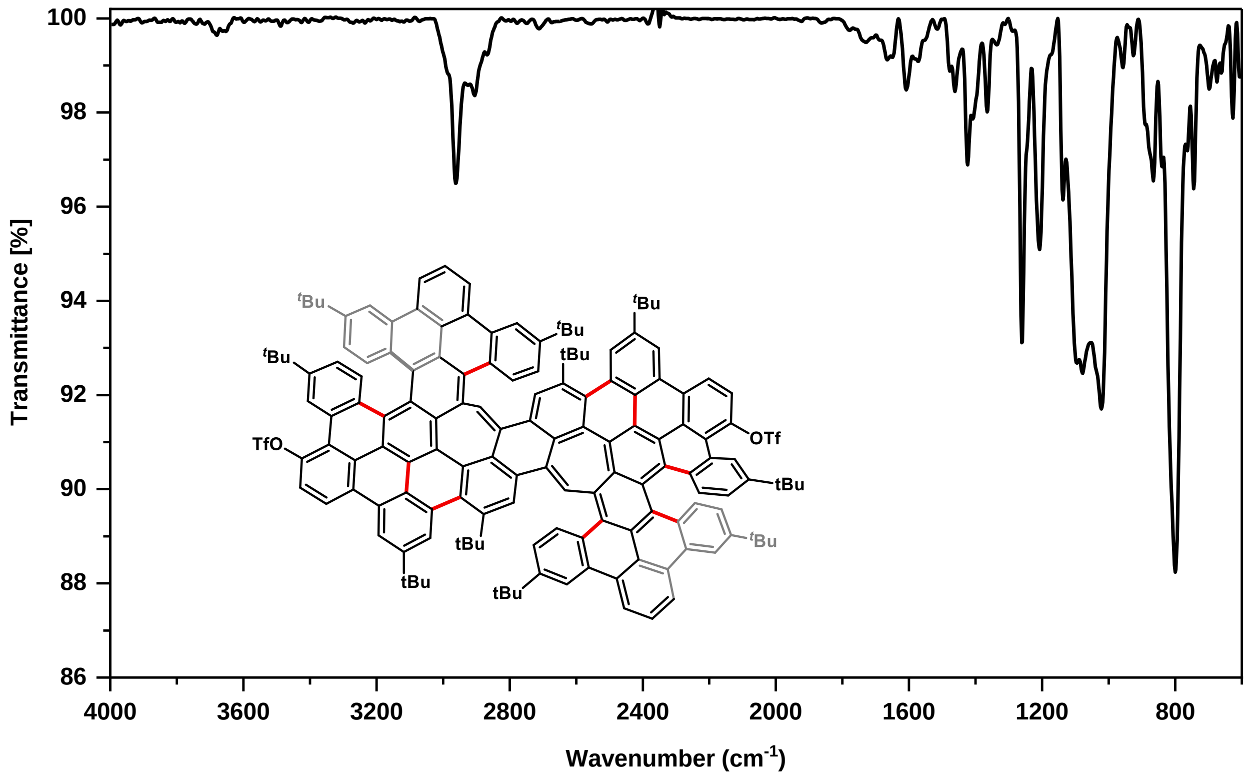

Figure S62. FT-IR spectrum (ATR, neat) of compound **11**.

5. Mass Spectra

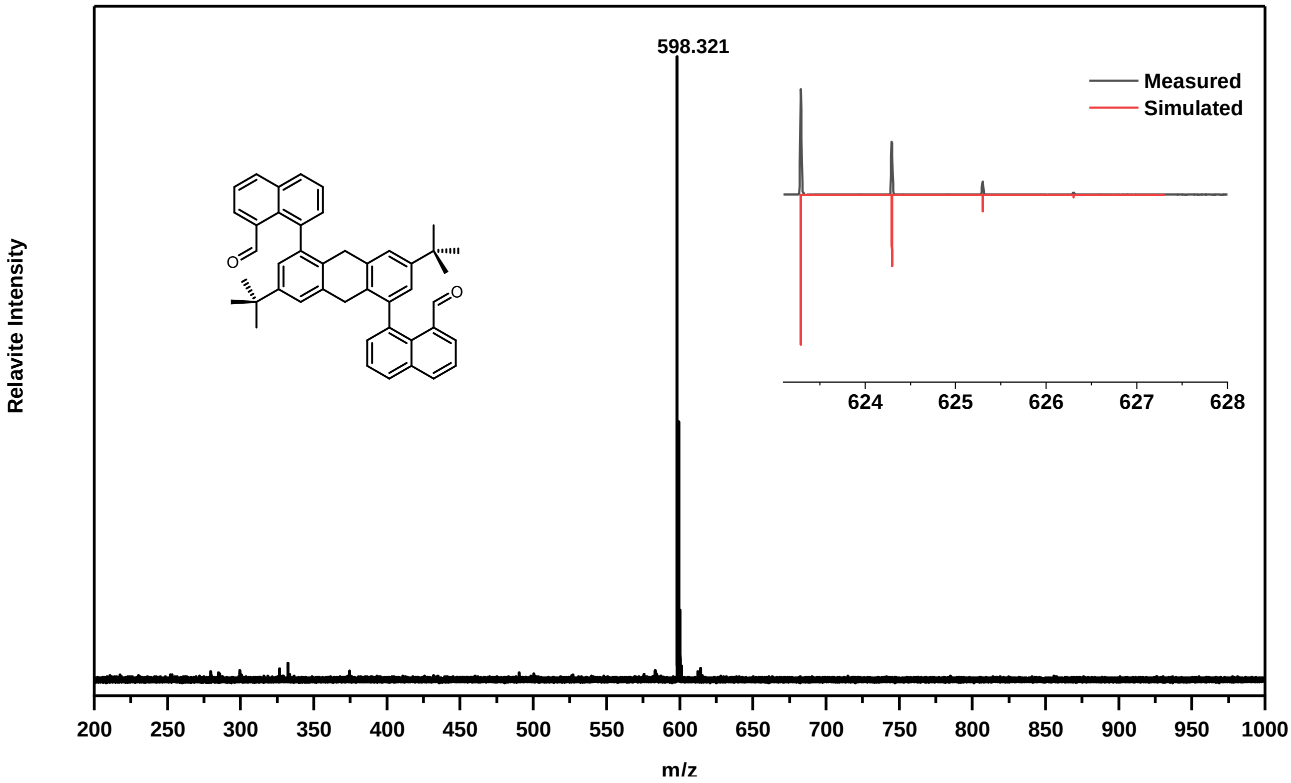

Figure S63. HR MALDI-MS of compound **4**. Outer spectrum was measured by Bruker Autoflex speed MALDI-TOF Instrument. Inset was measured by Bruker timsTOFfleX Instrument.

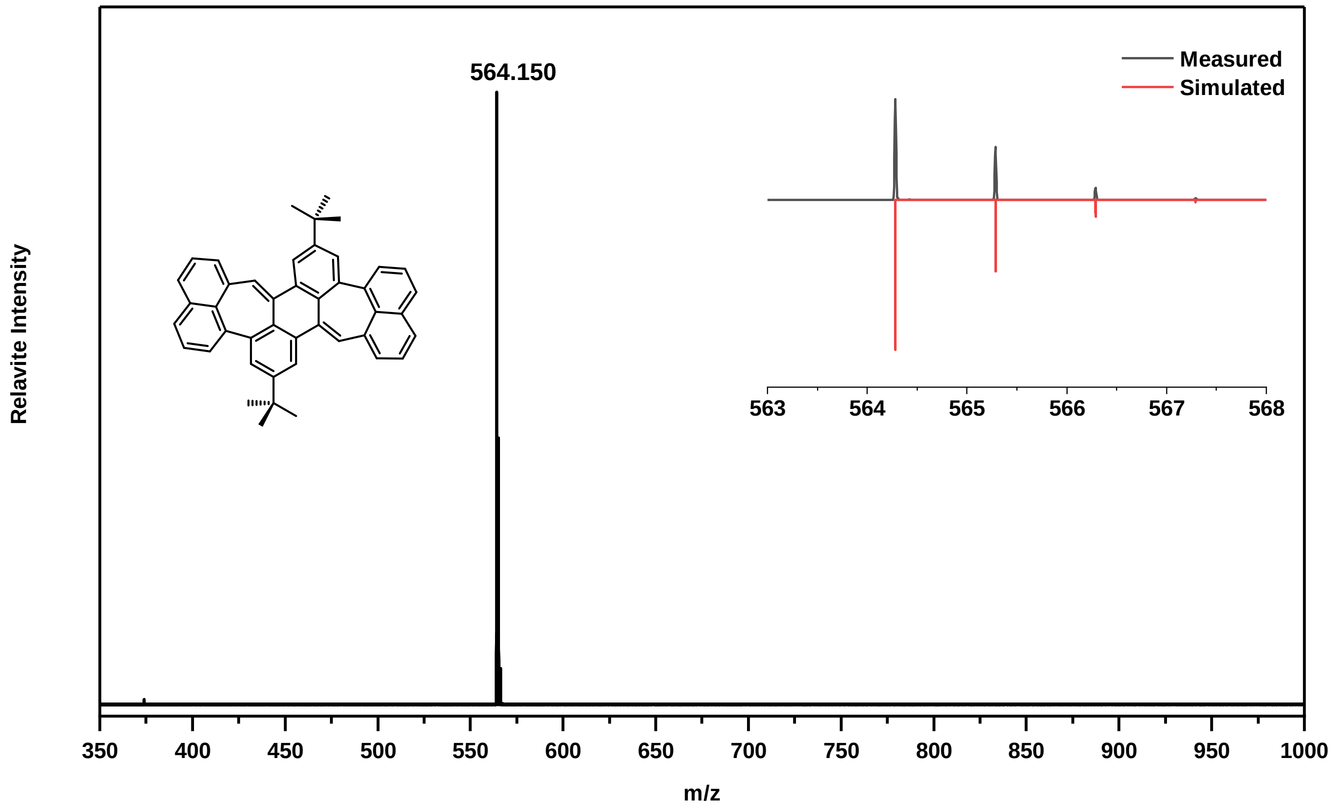

Figure S64. HR MALDI-MS of compound **5**. Outer spectrum was measured by Bruker Autoflex speed MALDI-TOF Instrument. Inset was measured by Bruker timsTOFfleX Instrument.

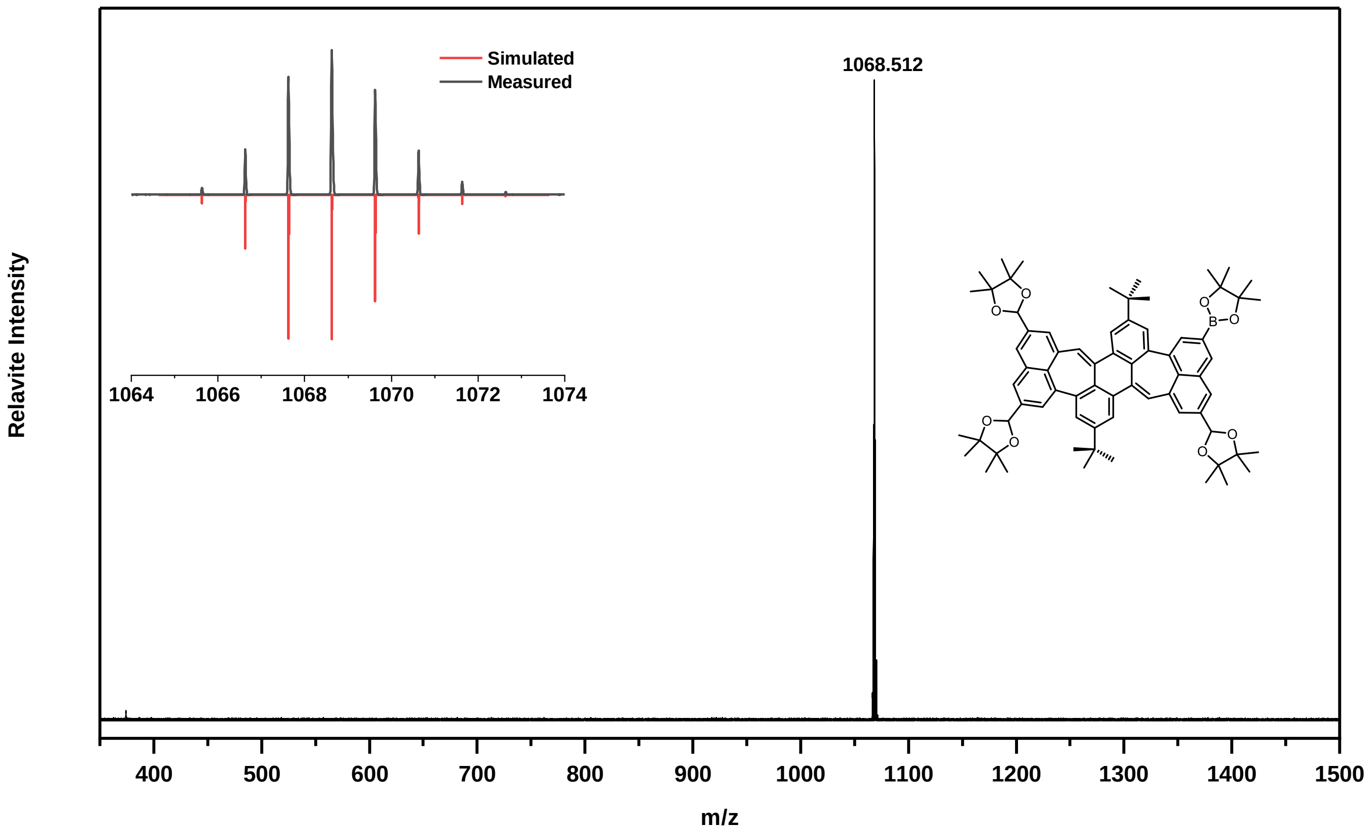

Figure S65. HR MALDI-MS of compound **6**. Outer spectrum was measured by Bruker Autoflex speed MALDI-TOF Instrument. Inset was measured by Bruker timsTOFfleX Instrument.

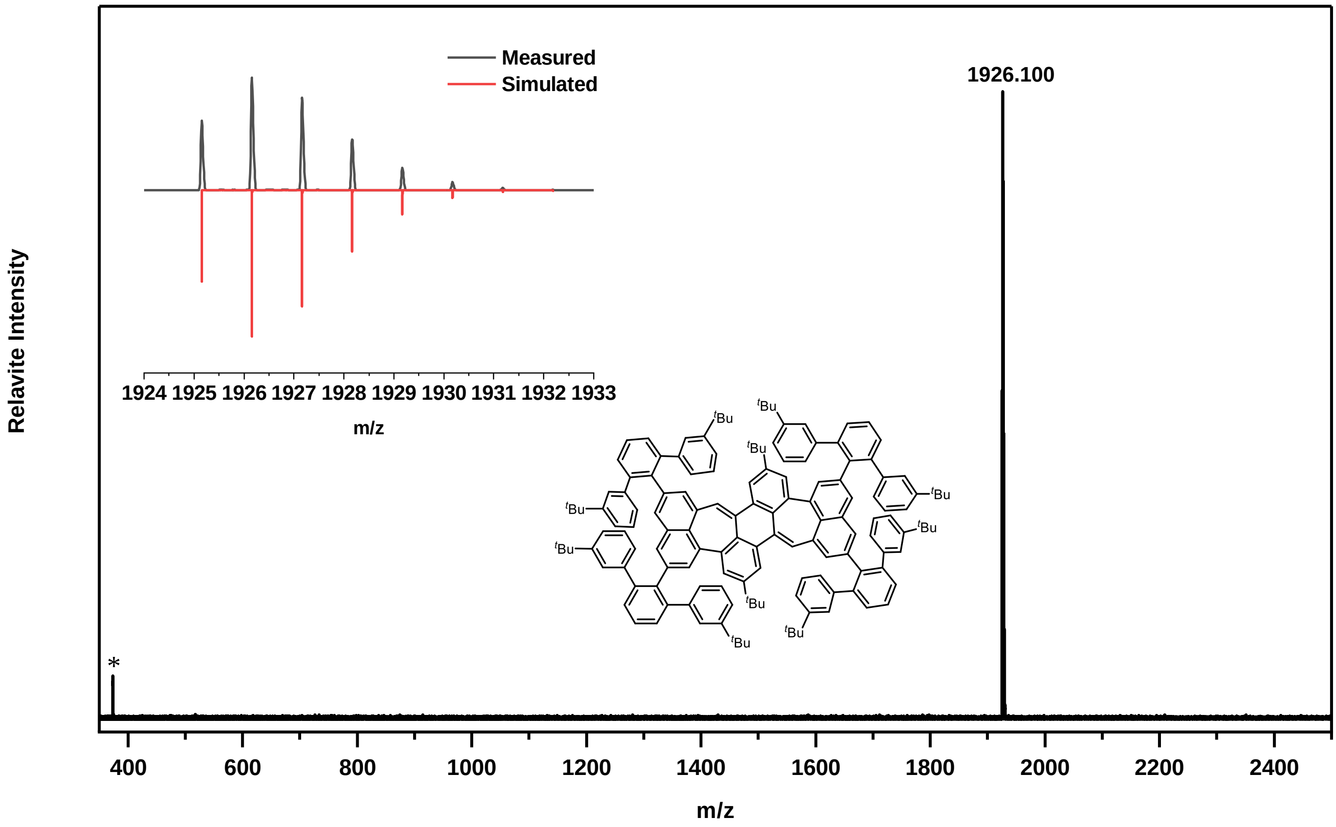

Figure S66. HR MALDI-MS of compound **7**. *: Artefact signal of the MALDI-TOF-MS measurement as proven by blank measurements. Outer spectrum was measured by Bruker Autoflex speed MALDI-TOF Instrument. Inset was measured by Bruker timsTOFfleX Instrument.

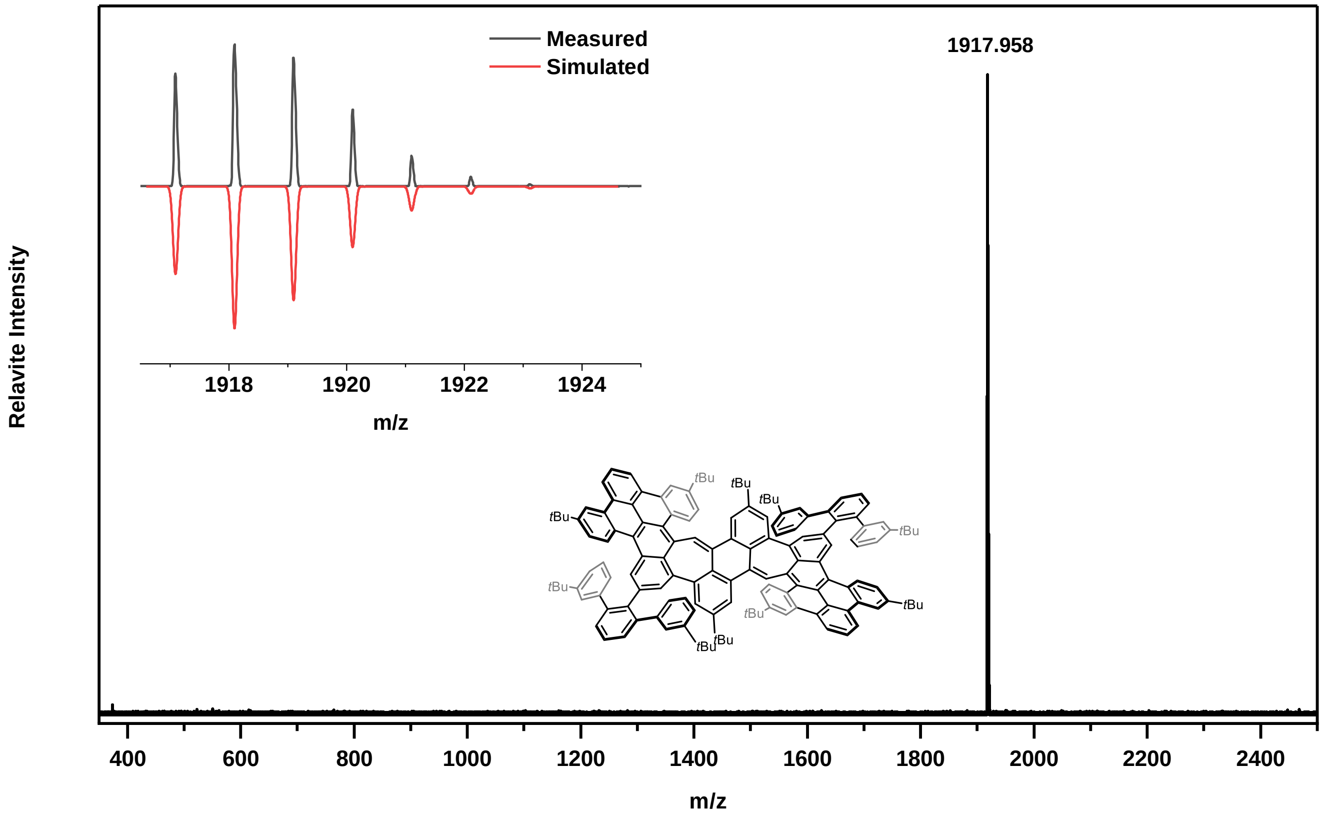

Figure S67. HR MALDI-MS of compound **8**. Outer spectrum was measured by Bruker Autoflex speed MALDI-TOF Instrument. Inset was measured by Bruker timsTOFfleX Instrument.

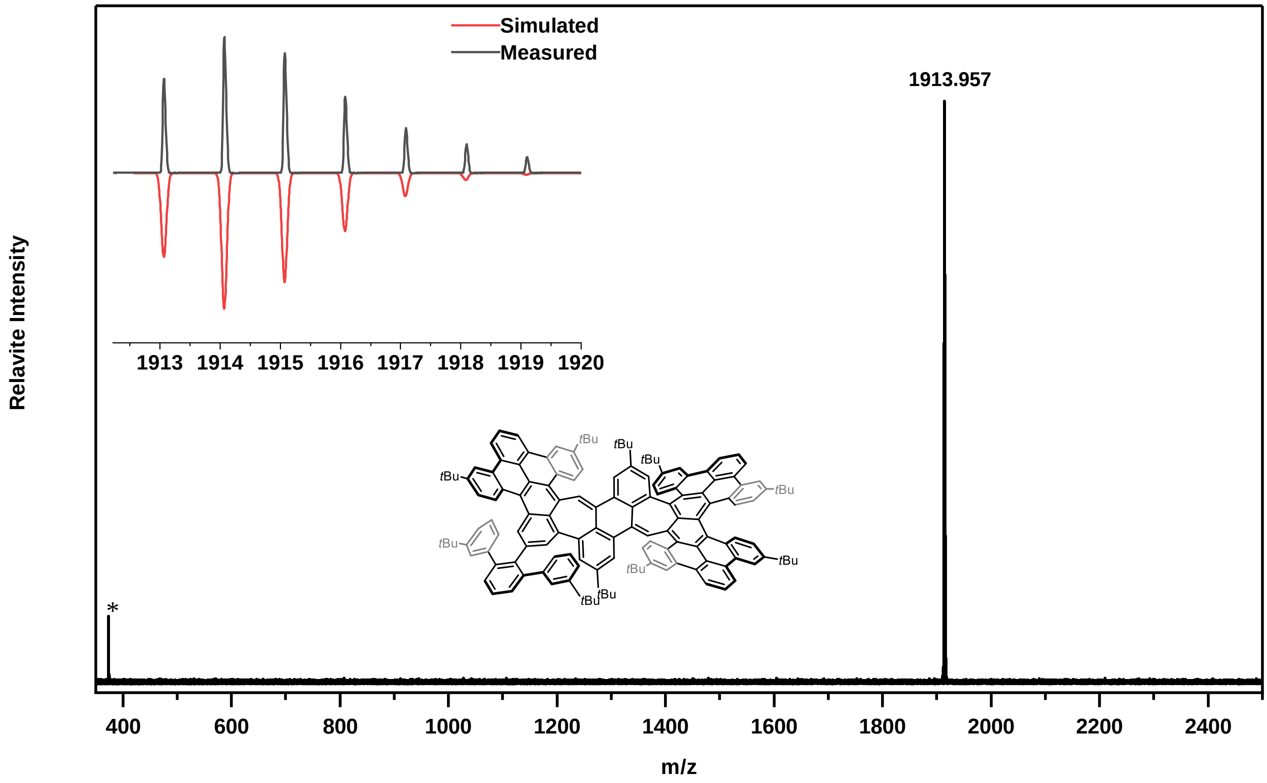

Figure S68. HR MALDI-MS of compound **9**. *: Artefact signal of the MALDI-TOF-MS measurement as proven by blank measurements. Outer spectrum was measured by Bruker Autoflex speed MALDI-TOF Instrument. Inset was measured by Bruker timsTOFfleX Instrument.

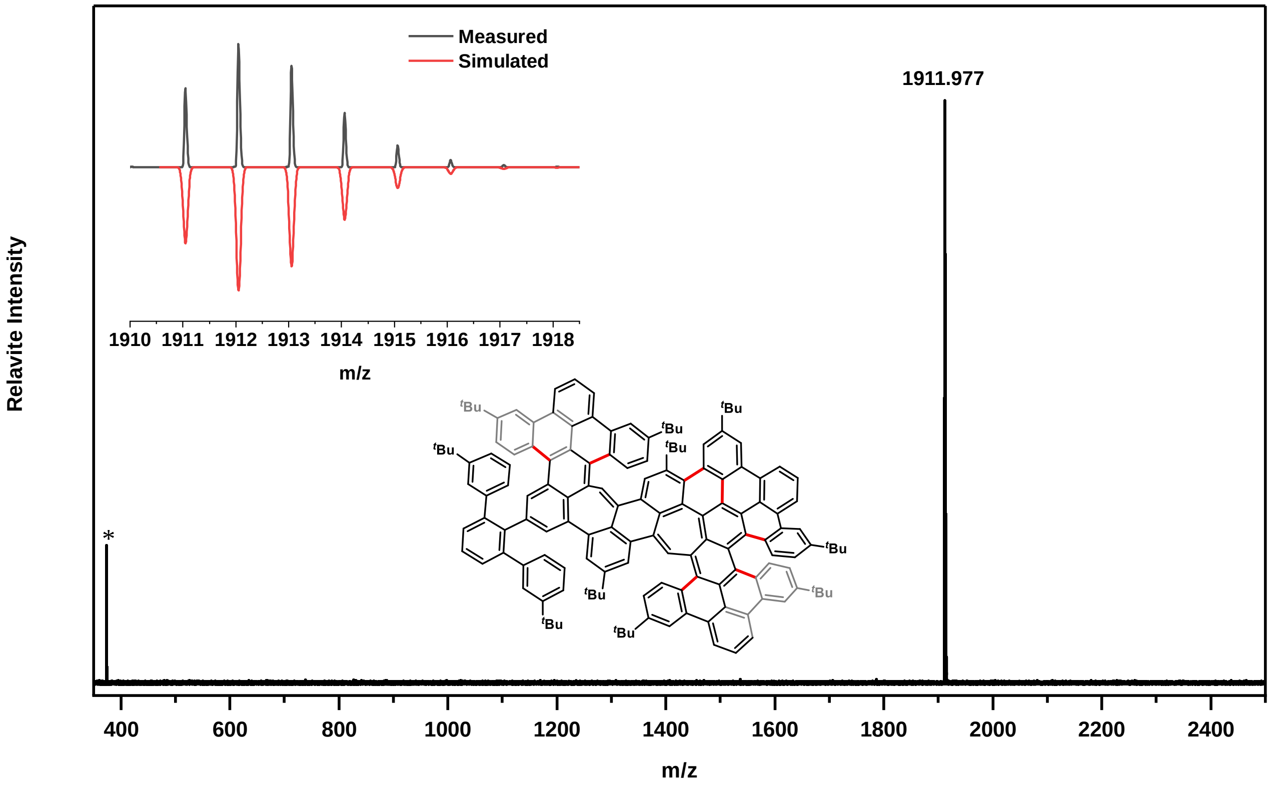

Figure S69. HR MALDI-MS of compound **10**. *: Artefact signal of the MALDI-TOF-MS measurement as proven by blank measurements. Outer spectrum was measured by Bruker Autoflex speed MALDI-TOF Instrument. Inset was measured by Bruker timsTOFfleX Instrument.

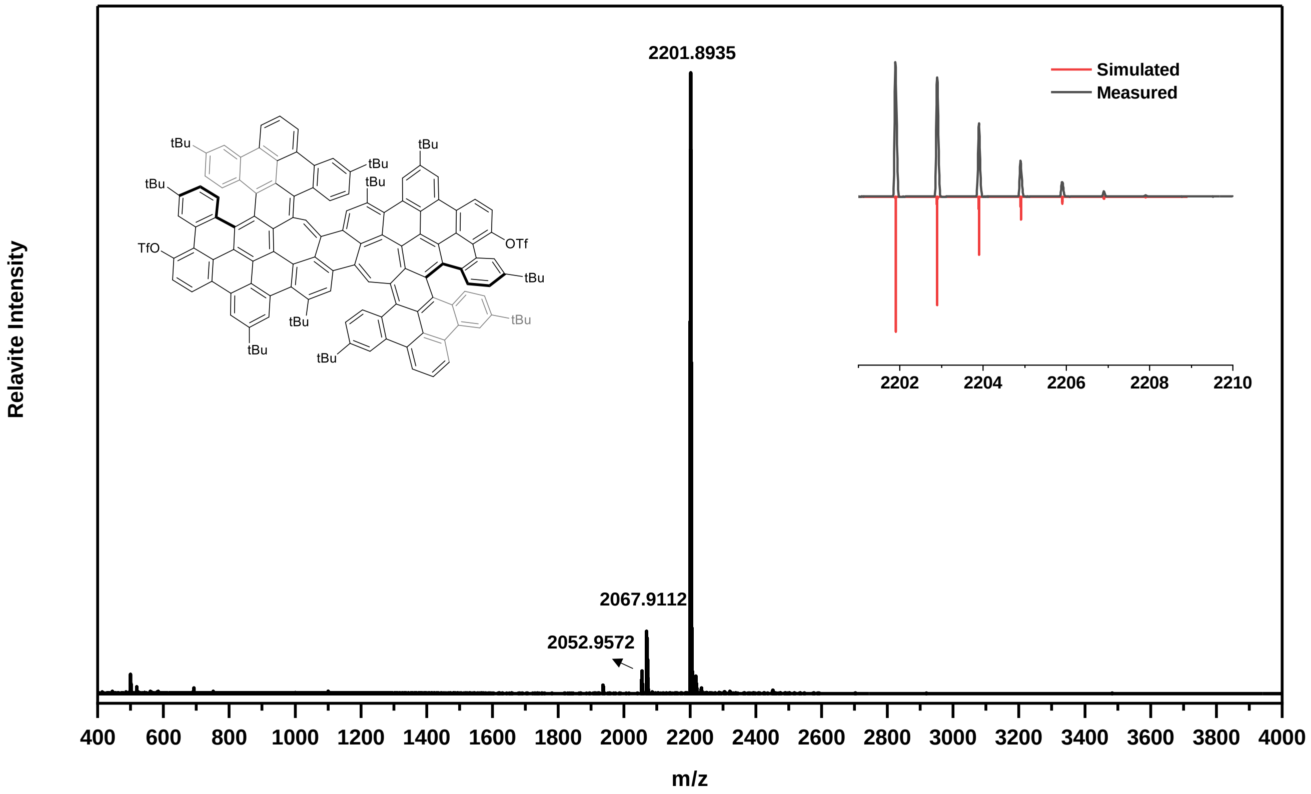

Figure S70. HR MALDI-MS of compound **11.** Measured by Bruker timsTOFfleX Instrument.

6. UV-vis and Fluorescence Spectra

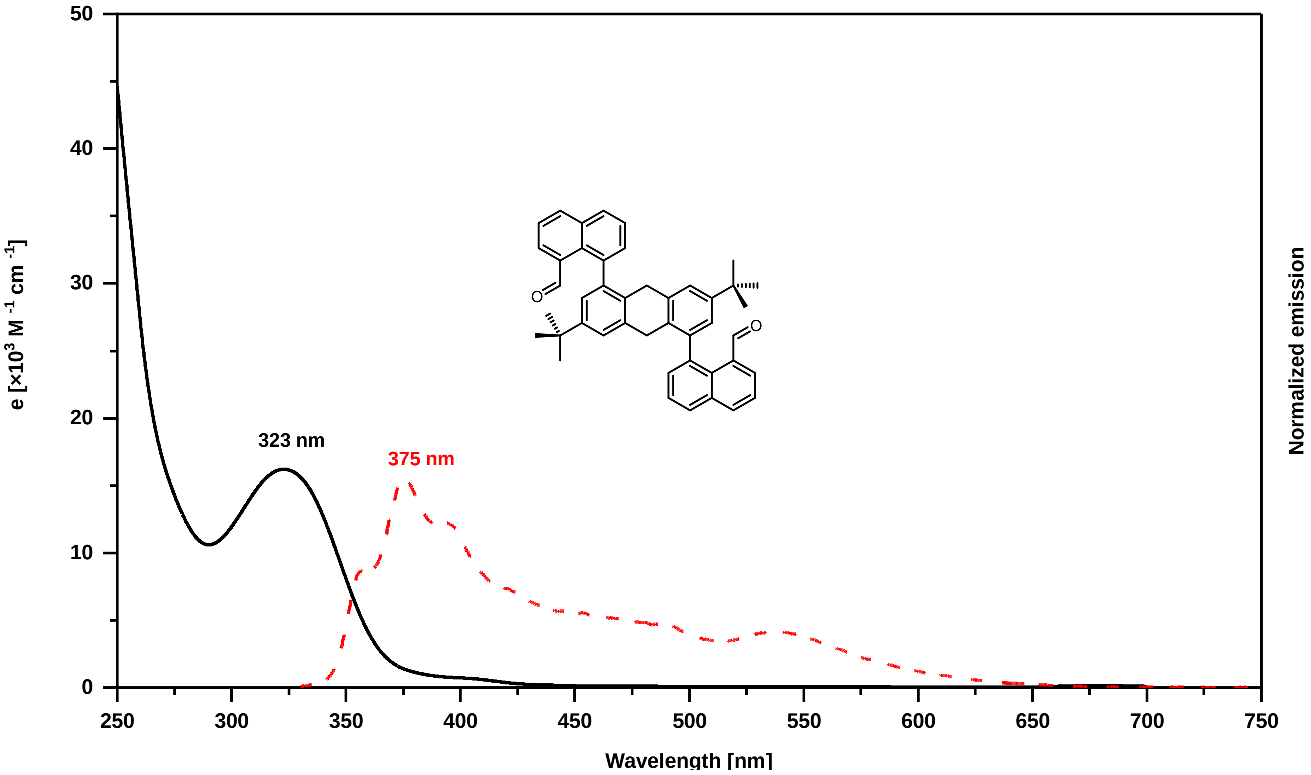

Figure S71. UV-vis (solid) and emission (λ_{ex} = 320 nm, dash) spectra of compound **4** measured in CH_2Cl_2 at room temperature.

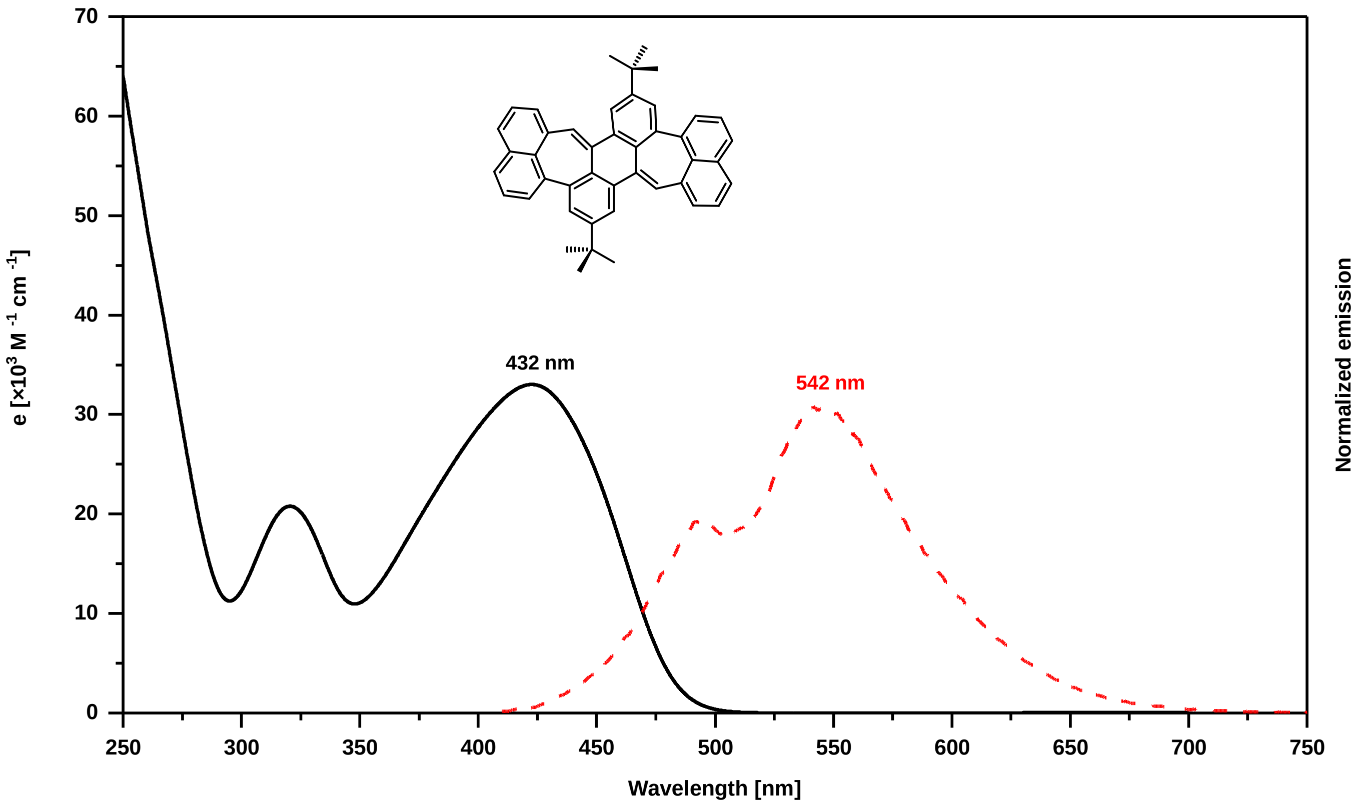

Figure S72. UV-vis (solid) and emission (λ_{ex} = 405 nm, dash) spectra of compound **5** measured in CH_2Cl_2 at room temperature.

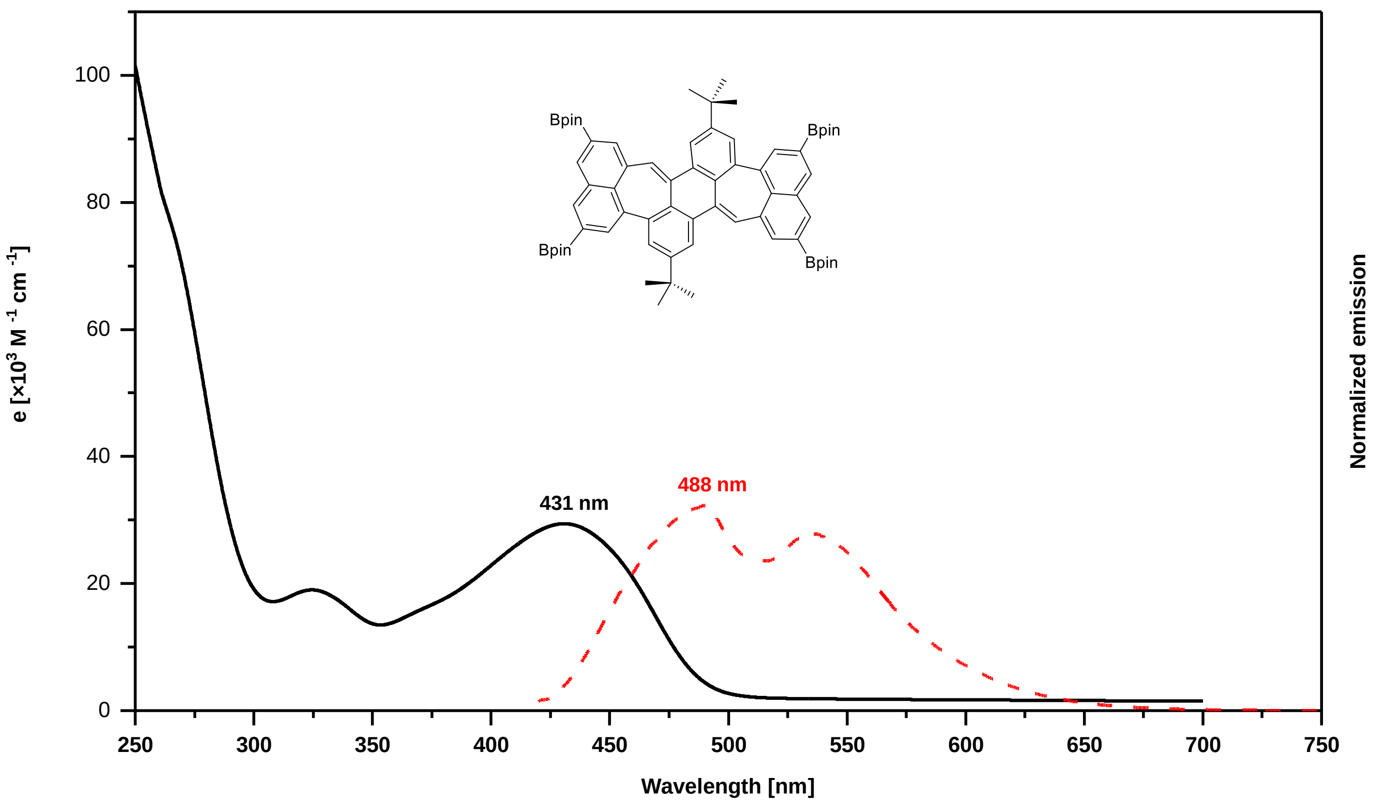

Figure S73. UV-vis (solid) and emission (λ_{ex} = 420 nm, dash) spectra of compound **6** measured in CH_2Cl_2 at room temperature.

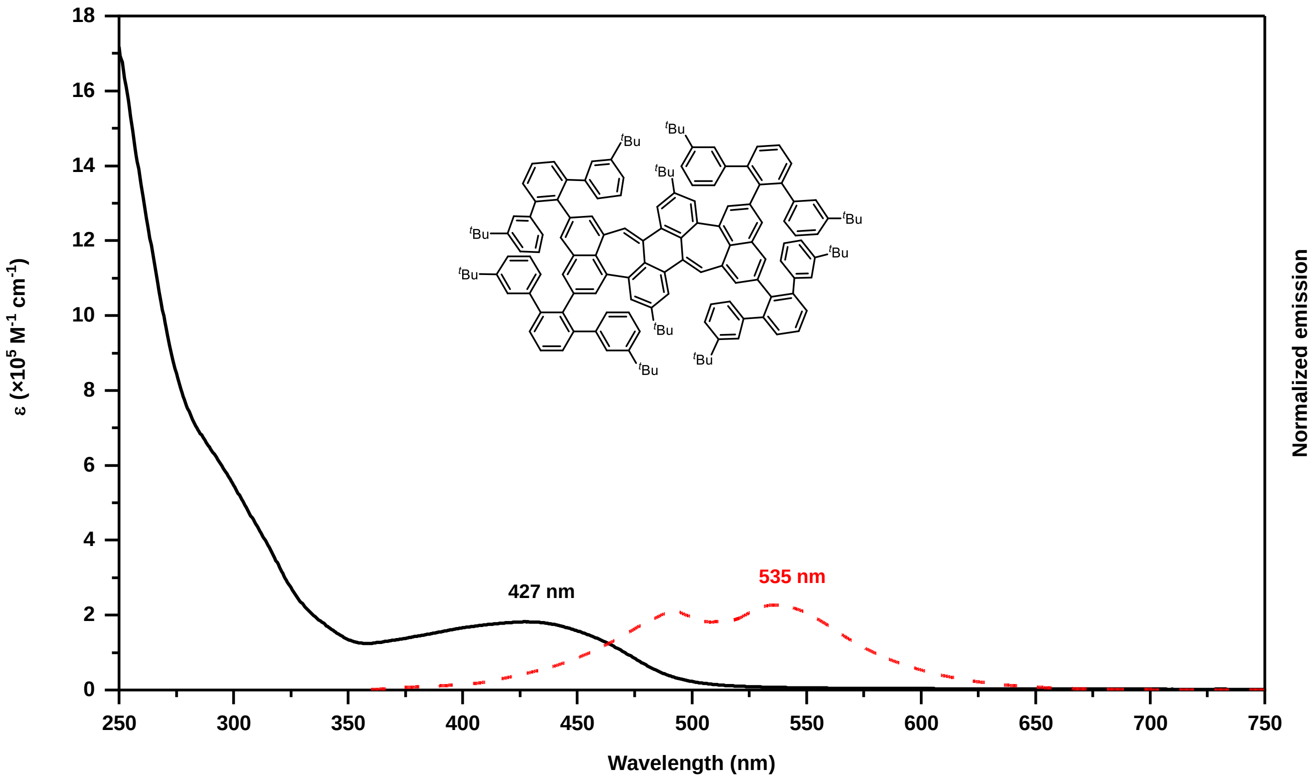

Figure S74. UV-vis (solid) and emission (λ_{ex} = 360 nm, dash) spectra of compound **7** measured in CH_2Cl_2 at room temperature.

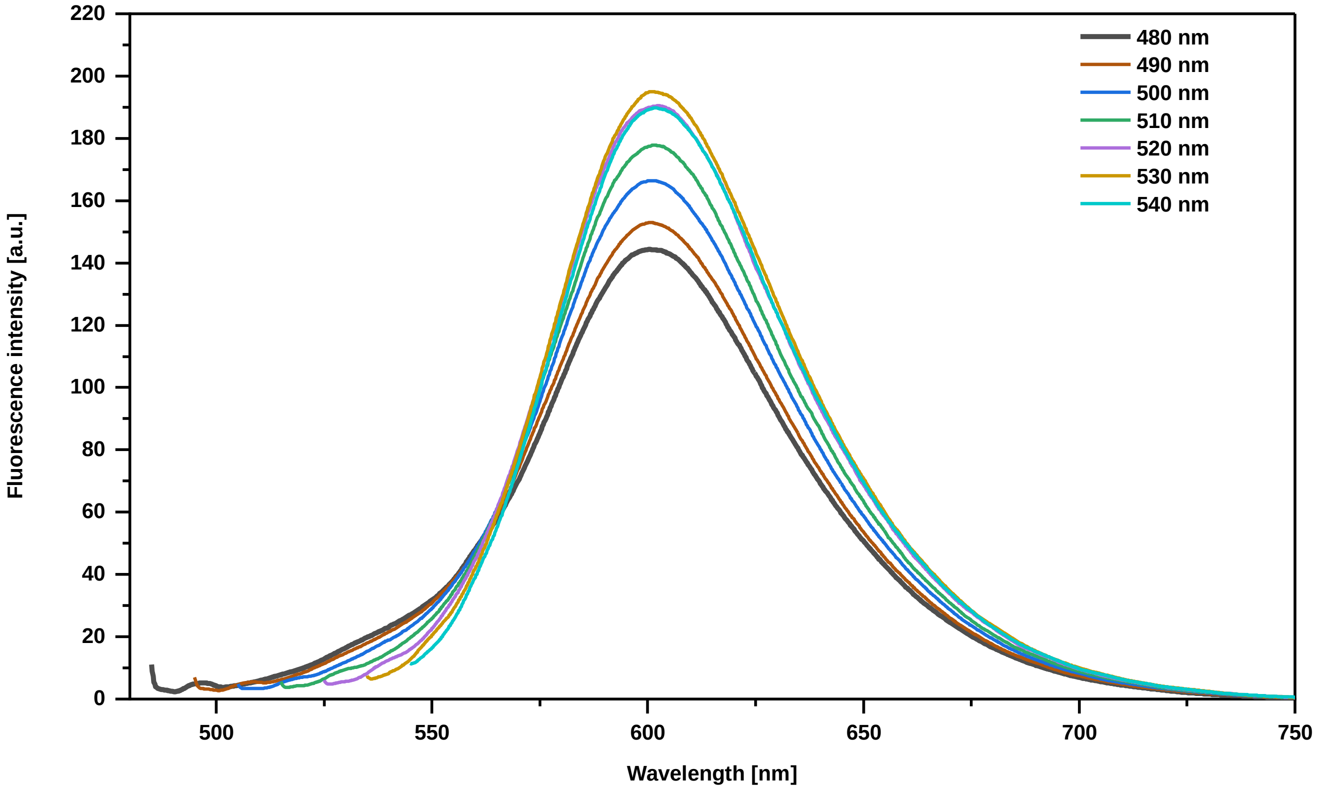

Figure S75. Excitation dependent emission spectra of compound **8** (concentration: 1.7 × 10^{-6} mol/L, absorbance = 0.096 at λ = 541 nm.). Measured in CH_2Cl_2 at room temperature.

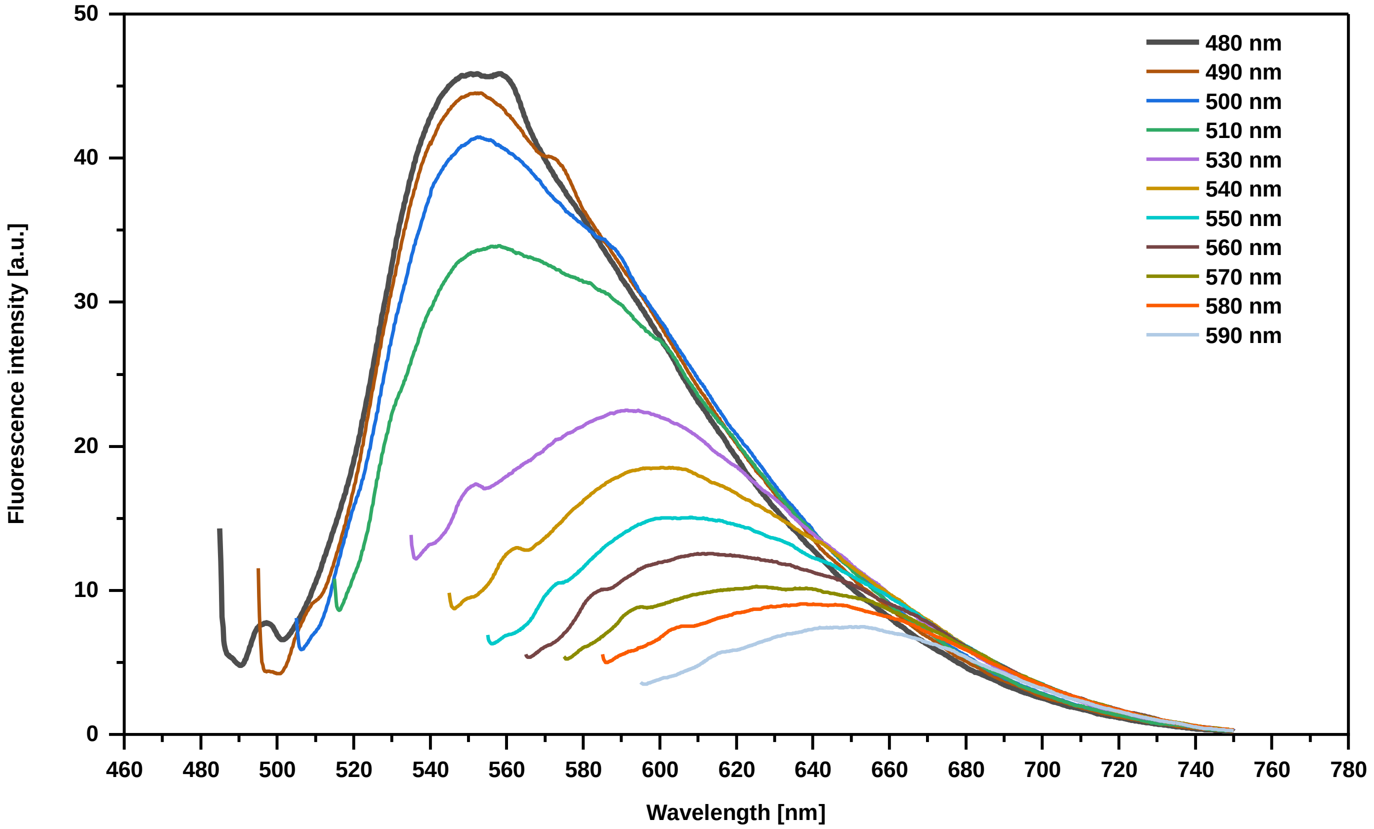

Figure S76. Excitation dependent emission spectra of compound **9** (concentration: 1.8 × 10^{-6} mol/L, absorbance = 0.084 at λ = 590 nm). Measured in CH_2Cl_2 at room temperature.

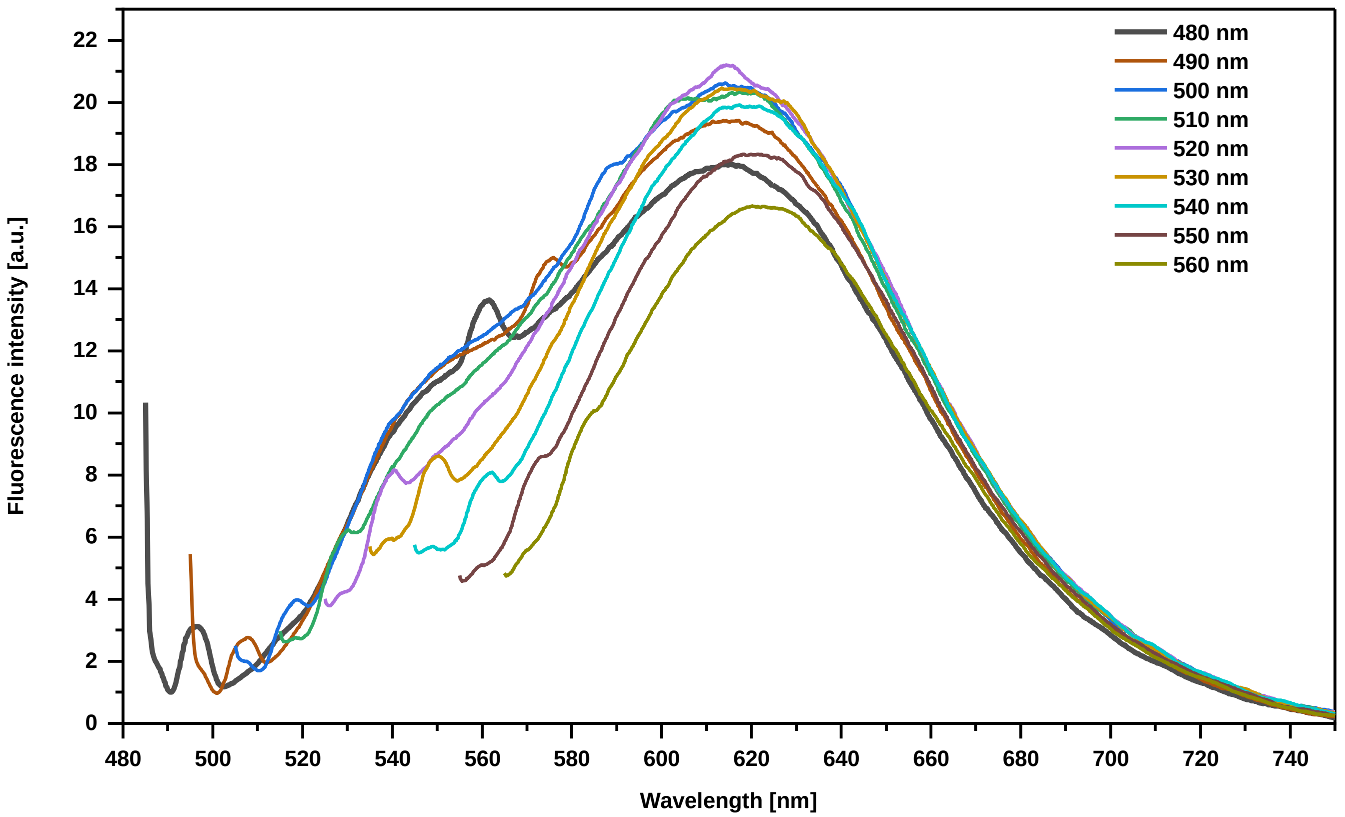

Figure S77. Excitation dependent emission spectra of compound **10** (concentration: 1.7 × 10^{-6} mol/L, absorbance = 0.075 at λ = 511 nm). Measured in CH_2Cl_2 at room temperature.

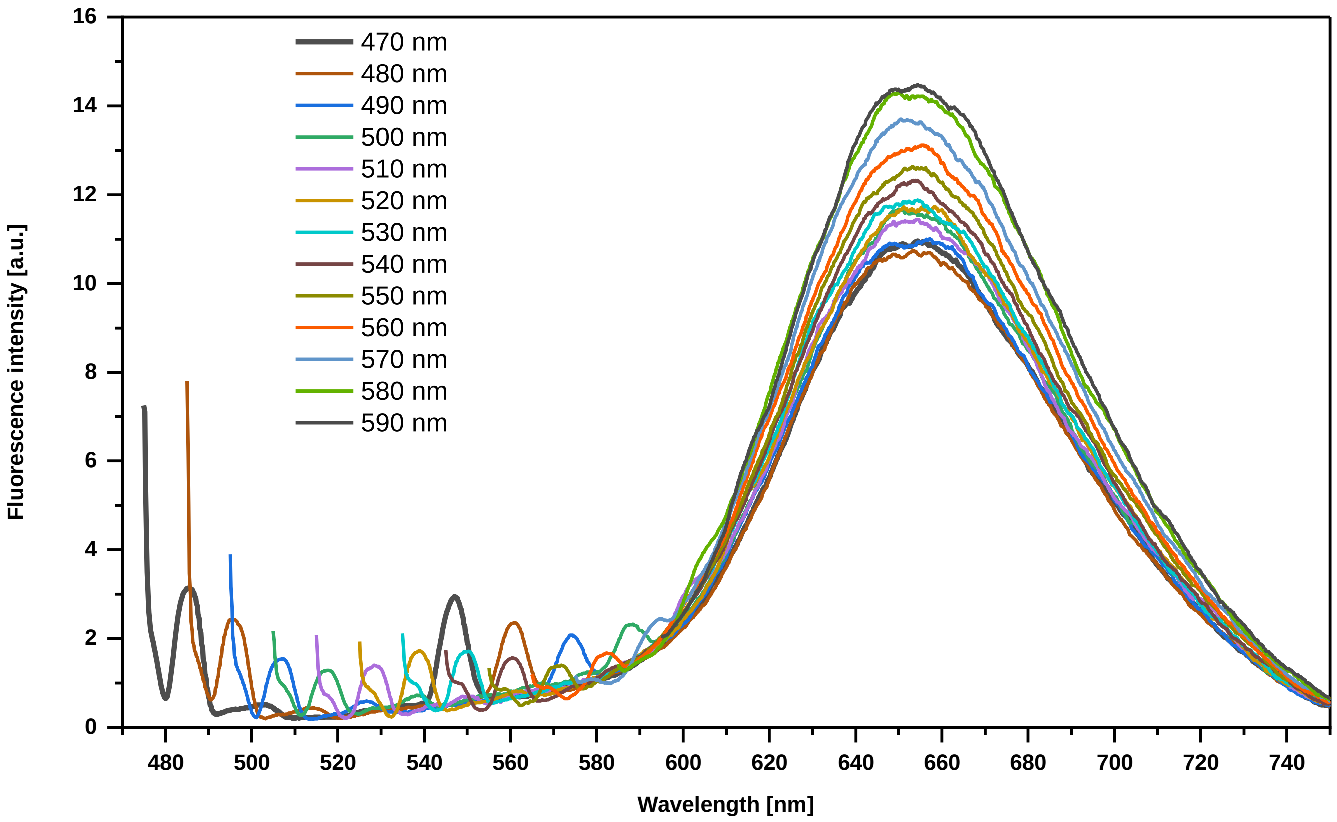

Figure S78. Excitation dependent emission spectra of compound **11** (concentration: 3.0 × 10^{-6} mol/L, absorbance = 0.096 at λ = 583 nm). Measured in CH_2Cl_2 at room temperature.

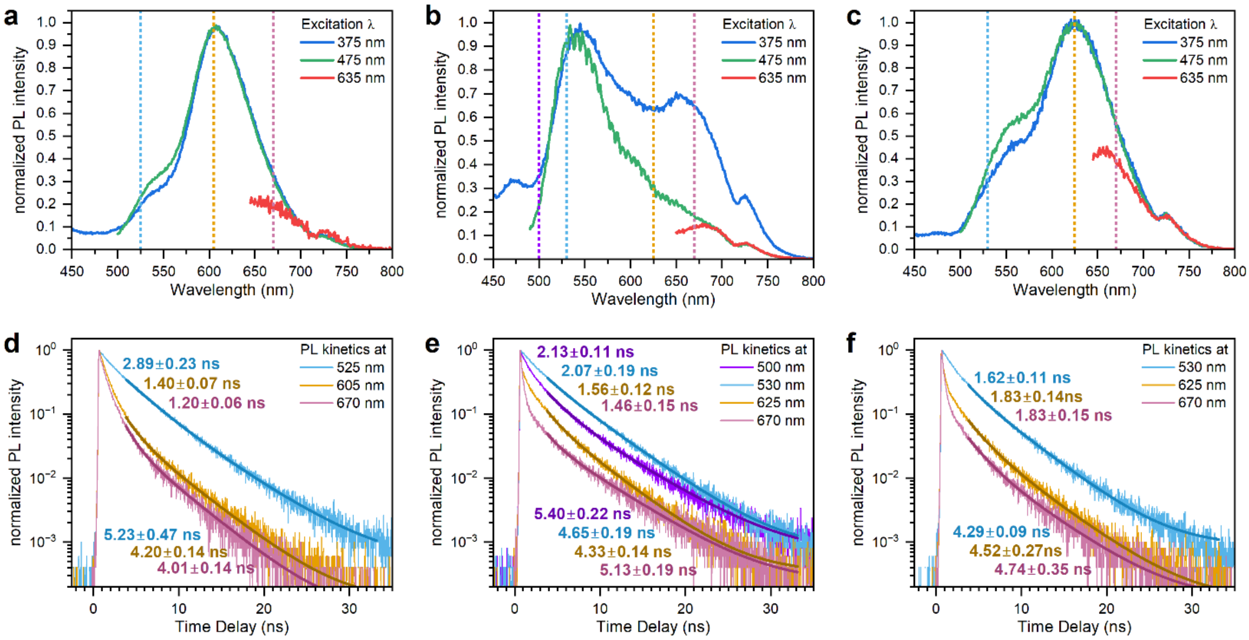

Figure S79. Normalized photoluminescence spectra (**a**) for **8**, **(b)** for **9** and **(c)** for **10** in CH_2Cl_2 solution at 20 °C upon excitation at 375, 475 and 635 nm. (**d**-**f**) corresponding kinetic traces upon excitation of 375 nm with photoluminescence lifetimes determined by biexponential fit.

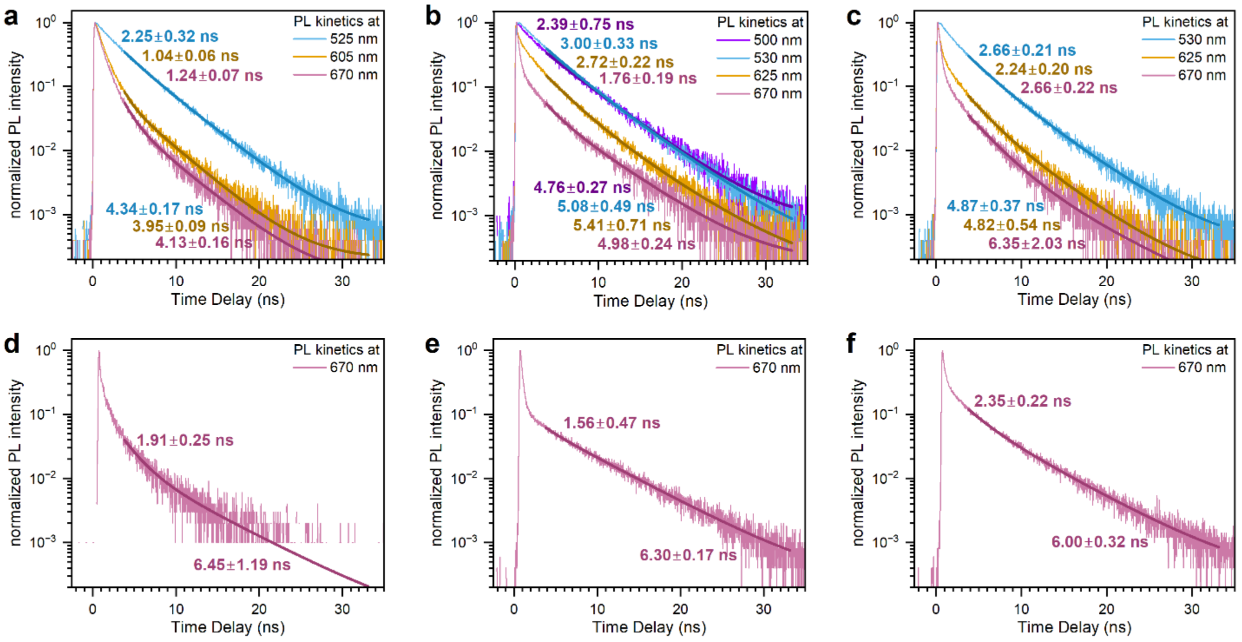

Figure S80. Kinetic traces of compounds **8**, **9** and **10** (from left to right) in CH_2Cl_2 solution at 20° C at selected wavelengths within the emission spectra upon excitation at 475 nm (**a**–**c**) and 635 nm (**d**–**f**) with photoluminescence lifetimes determined by biexponential fit.

7. Cyclic Voltammograms and Differential pulse voltammograms

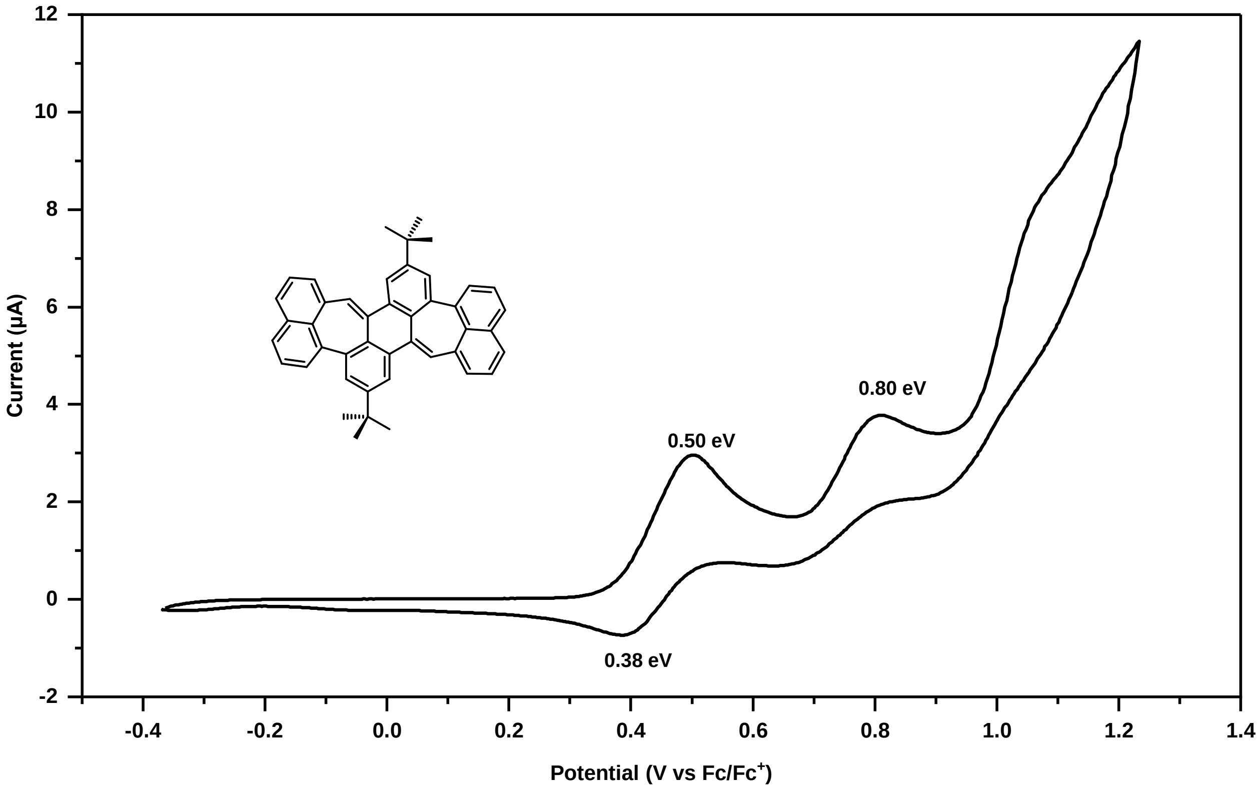

Figure S81. Cyclic voltammogram of **5** (CH_2Cl_2, 0.1 M NBu_4PF_6, scan rate: 100 mV·s^{-1}, working electrode: Glassy carbon, counter electrode: Pt, pseudo-reference electrode: Ag/Ag$^+$, room temperature) versus ferrocene as an internal reference.

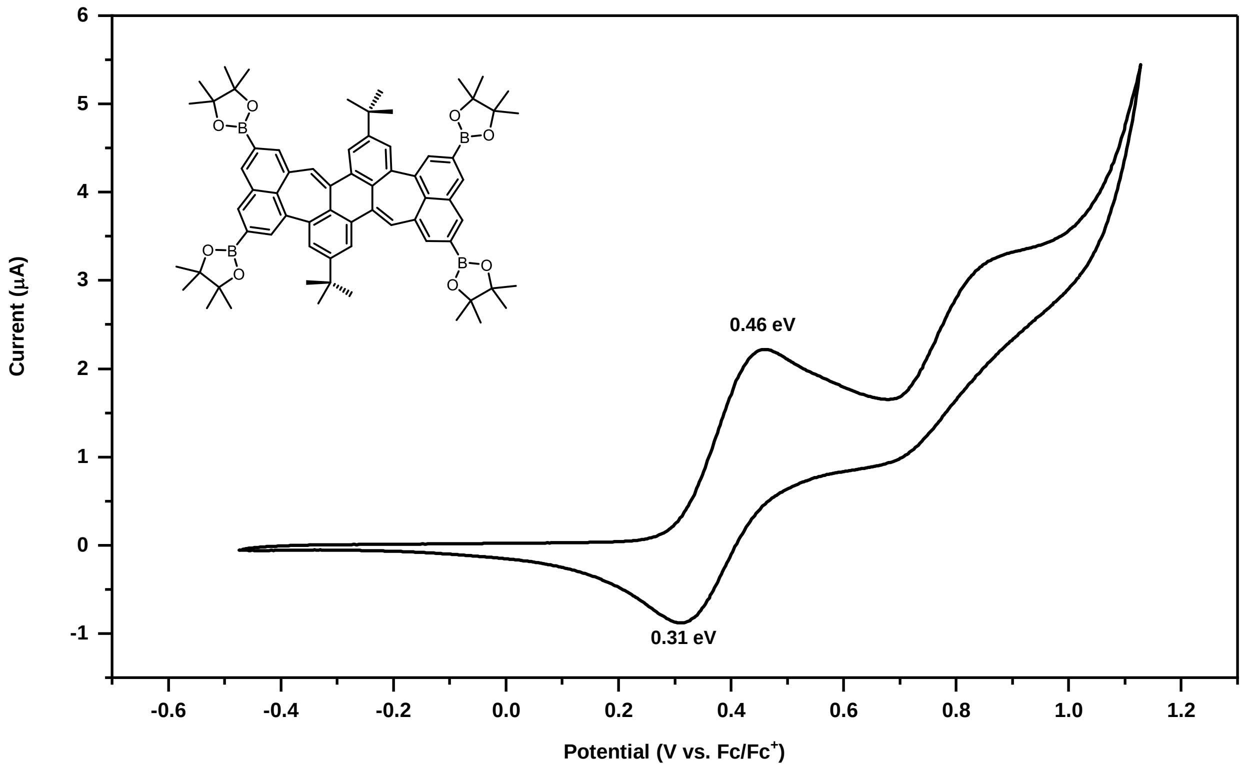

Figure S82. Cyclic voltammogram of **6** (CH_2Cl_2, 0.1 M NBu_4PF_6, scan rate: 100 mV·s^{-1}, working electrode: Glassy carbon, counter electrode: Pt, pseudo-reference electrode: Ag/Ag$^+$, room temperature) versus ferrocene as an internal reference.

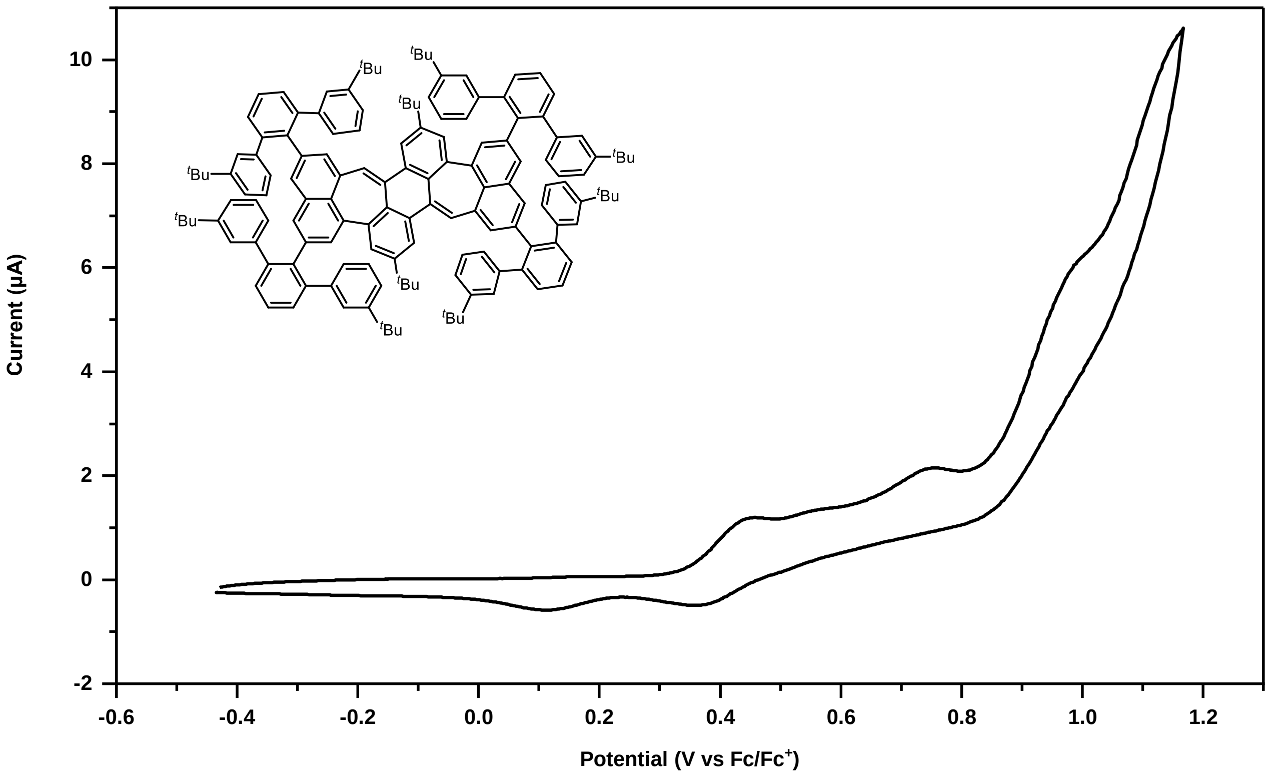

Figure S83. Cyclic voltammogram of **7** (CH_2Cl_2, 0.1 M NBu_4PF_6, scan rate: 100 mV·s^{-1}, working electrode: Glassy carbon, counter electrode: Pt, pseudo-reference electrode: Ag/Ag$^+$, room temperature) versus ferrocene as an internal reference.

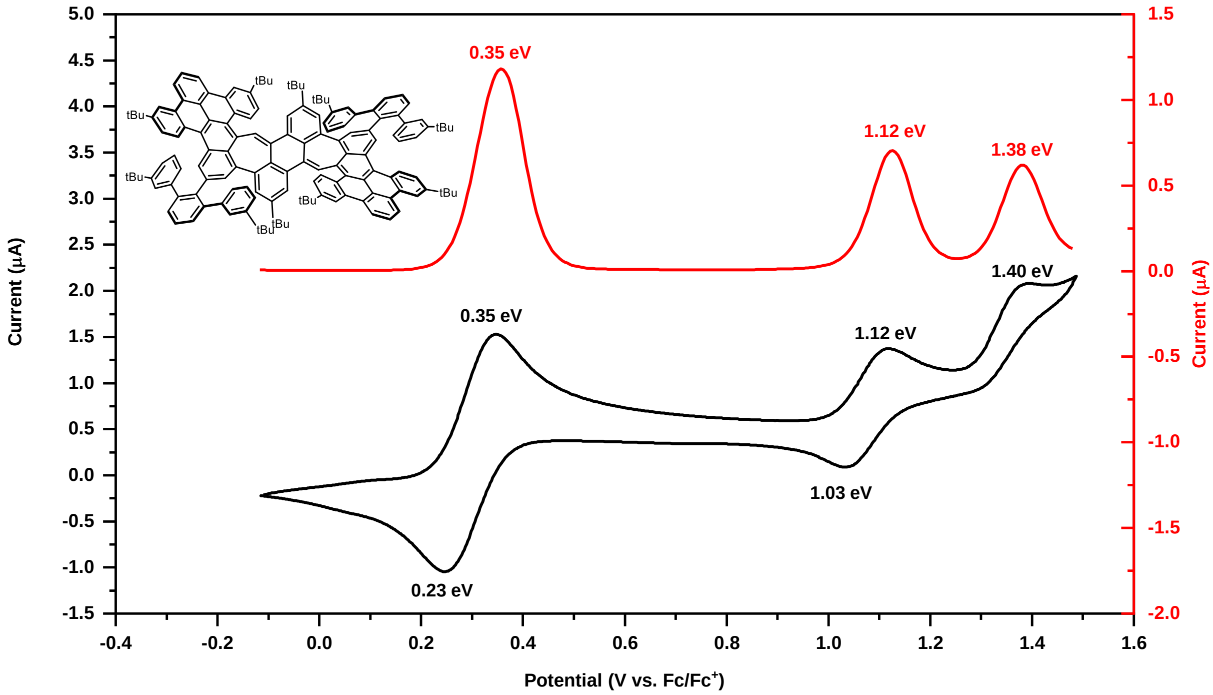

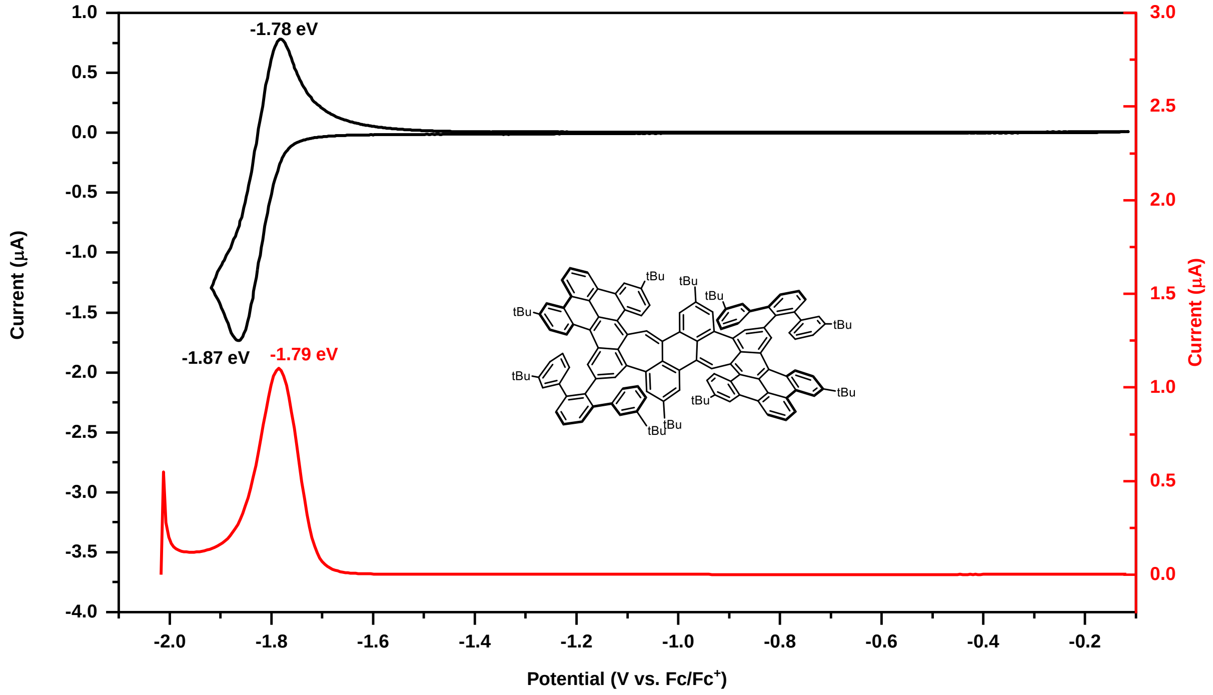

Figure S84. Cyclic voltammogram (black) and differential pulse voltammogram (red) of **8** (CH_2Cl_2, 0.1 M NBu_4PF_6, scan rate: 100 mV·s^{-1}, working electrode: Glassy carbon, counter electrode: Pt, pseudo-reference electrode: Ag/Ag^{+}, room temperature) versus ferrocene as an internal reference. For CV the scan speed was 100 mV/s. The DPV was obtained with a step size of 0.005 V, a modulation amplitude of 0.025 V a modulation time of 0.05 s and an interval time of 0.5 s.

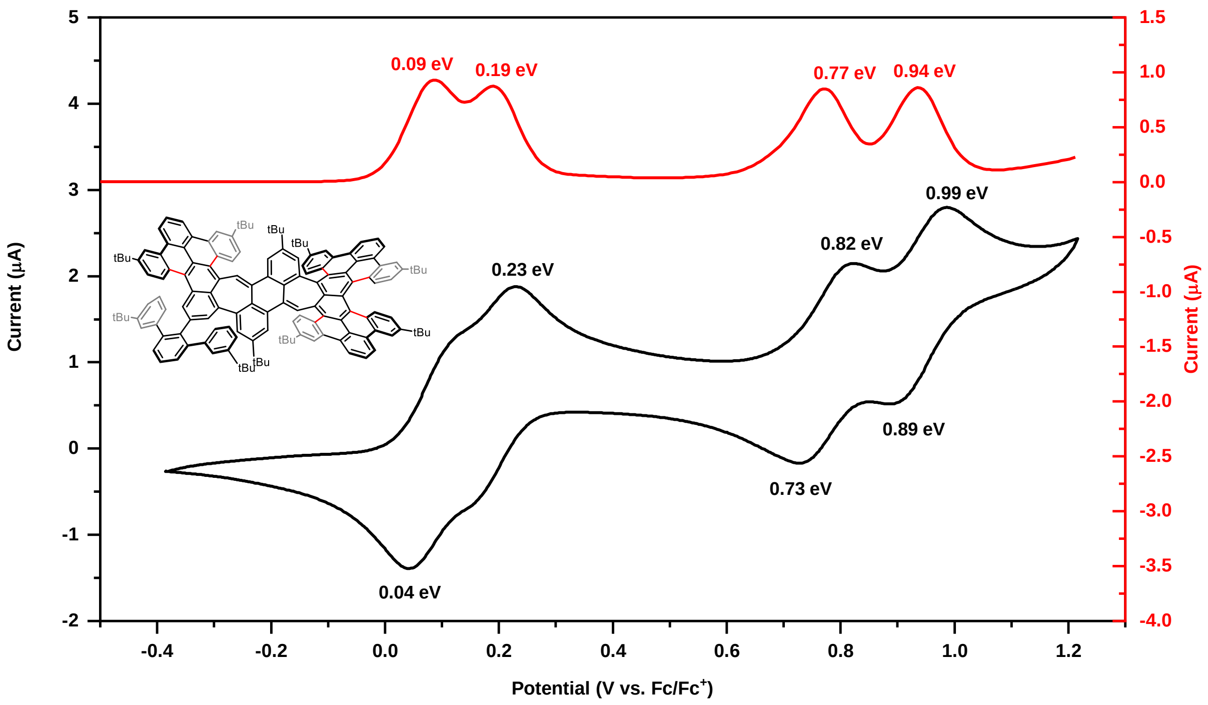

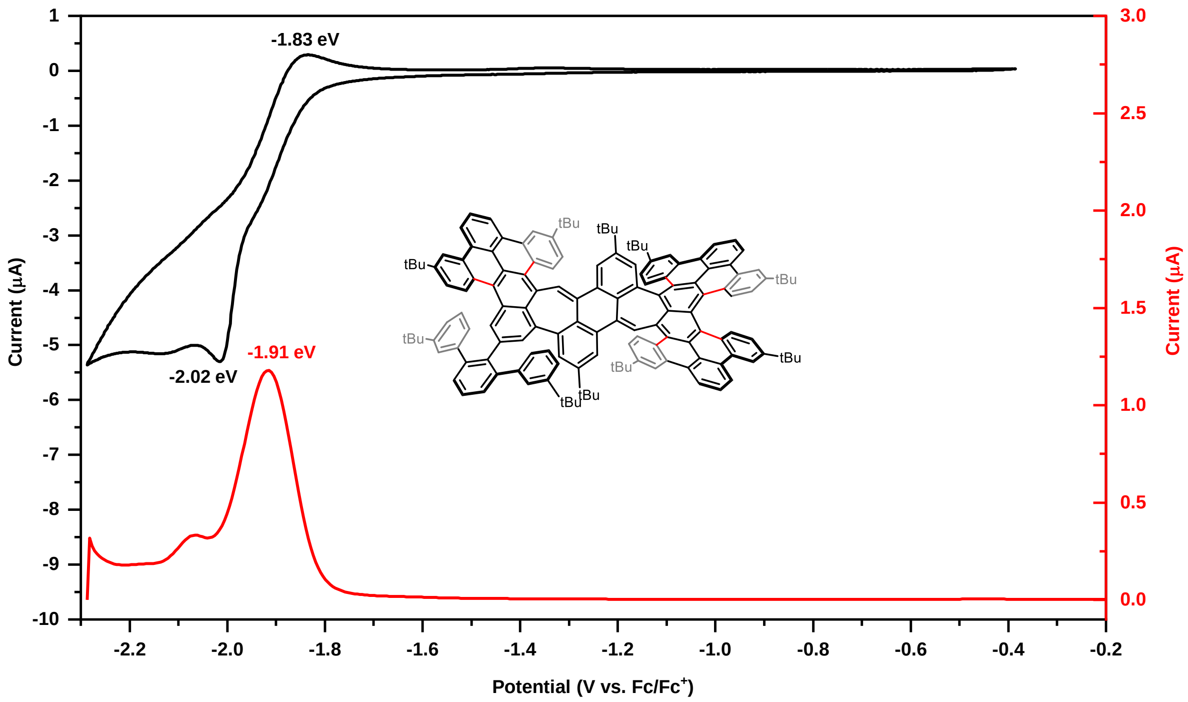

Figure S85. Cyclic voltammogram (black) and differential pulse voltammogram (red) of **9** (CH_2Cl_2, 0.1 M NBu_4PF_6, scan rate: 100 mV·s^{-1}, working electrode: Glassy carbon, counter electrode: Pt, pseudo-reference electrode: Ag/Ag$^+$, room temperature) versus ferrocene as an internal reference. For CV the scan speed was 100 mV/s. The DPV was obtained with a step size of 0.005 V, a modulation amplitude of 0.025 V a modulation time of 0.05 s and an interval time of 0.5 s.

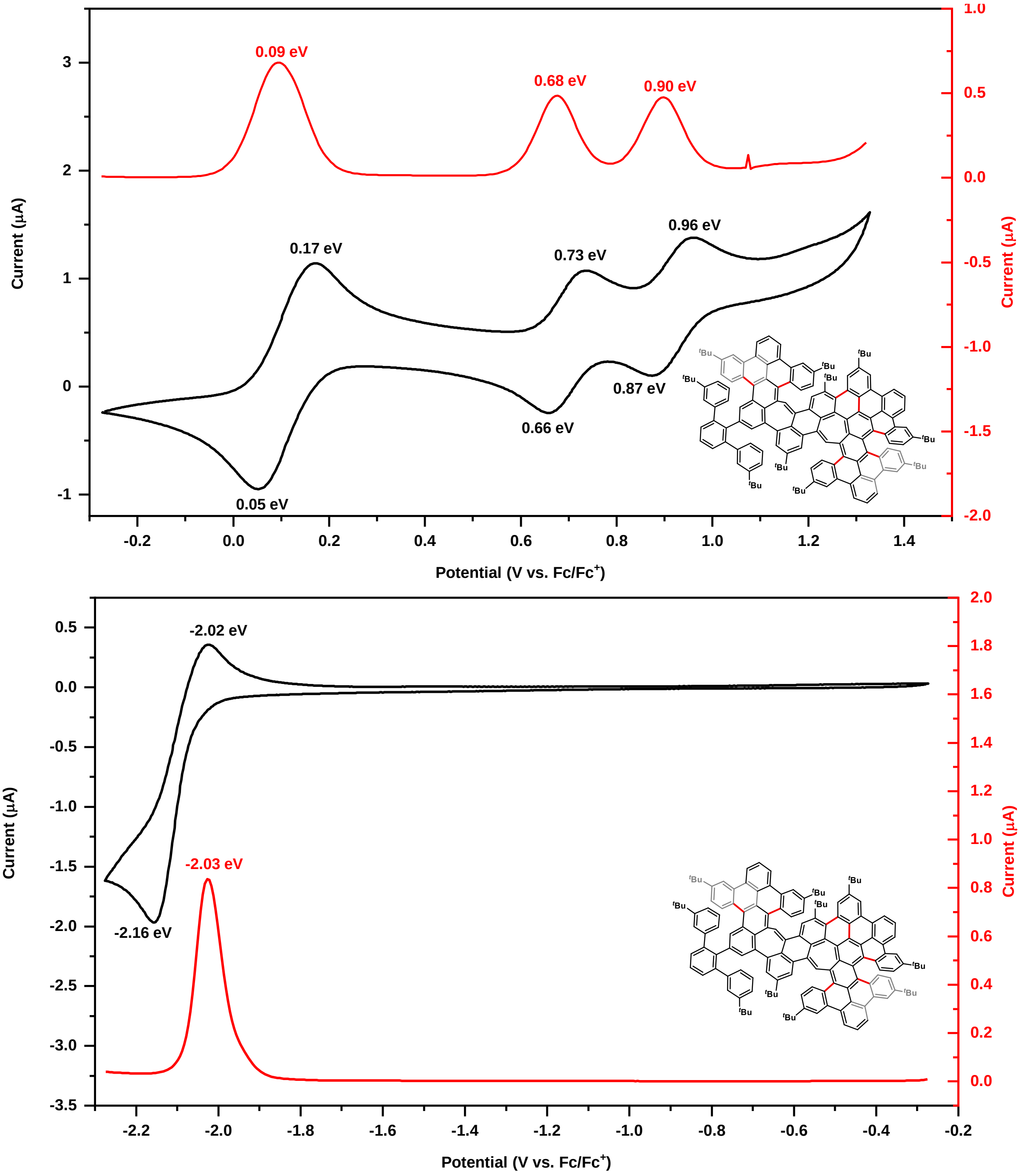

Figure S86. Cyclic voltammogram (black) and differential pulse voltammogram (red) of **10** (CH_2Cl_2, 0.1 M NBu_4PF_6, scan rate: 100 mV·s^{-1}, working electrode: Glassy carbon, counter electrode: Pt, pseudo-reference electrode: Ag/Ag^+, room temperature) versus ferrocene as an internal reference. For CV the scan speed was 100 mV/s. The DPV was obtained with a step size of 0.005 V, a modulation amplitude of 0.025 V a modulation time of 0.05 s and an interval time of 0.5 s.

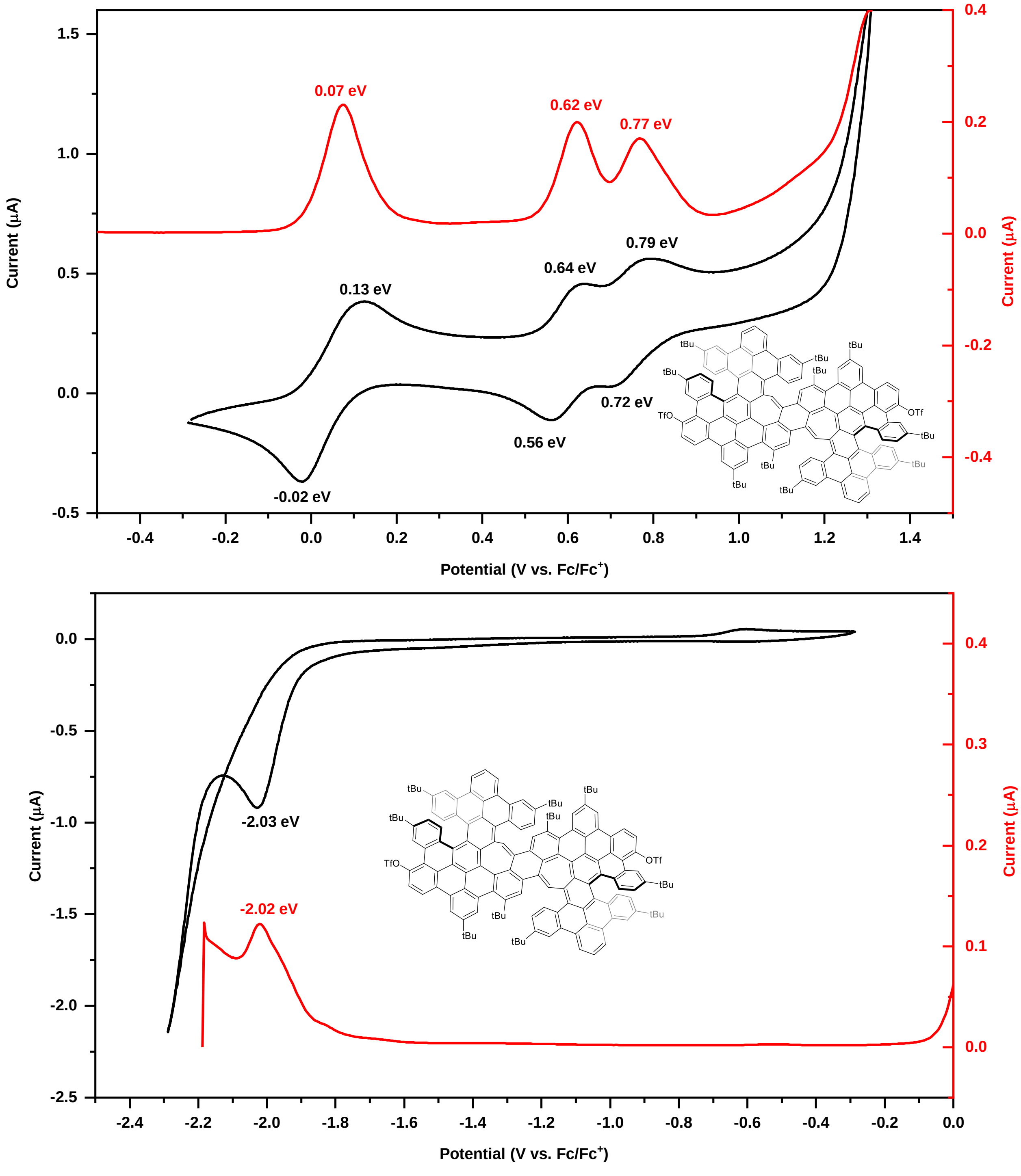

Figure S87. Cyclic voltammogram (black) and differential pulse voltammogram (red) of **11** (CH_2Cl_2, 0.1 M NBu_4PF_6, scan rate: 100 mV·s^{-1}, working electrode: Glassy carbon, counter electrode: Pt, pseudo-reference electrode: Ag/Ag$^+$, room temperature) versus ferrocene as an internal reference. For CV the scan speed was 100 mV/s. The DPV was obtained with a step size of 0.005 V, a modulation amplitude of 0.025 V a modulation time of 0.05 s and an interval time of 0.5 s.

8. Chromatograms

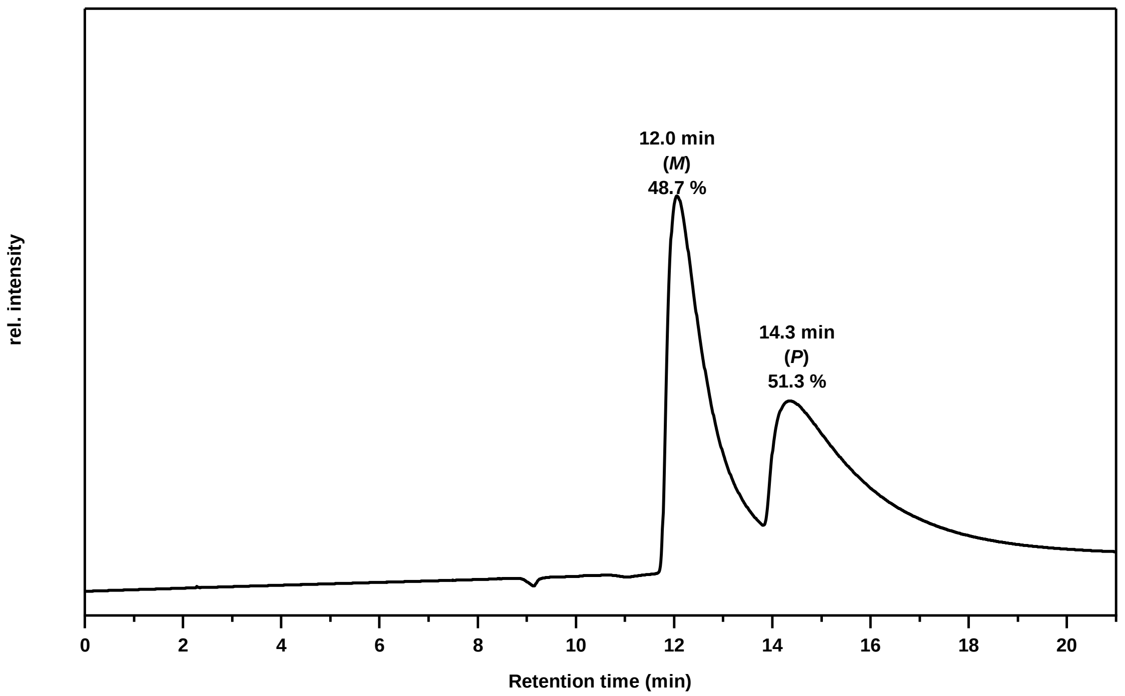

Figure S88. Preparative chromatogram of the racemic mixture of compound **9** (IC column, *n*-heptane/DCM 95:5, 10 mL min^{-1}, 254 nm).

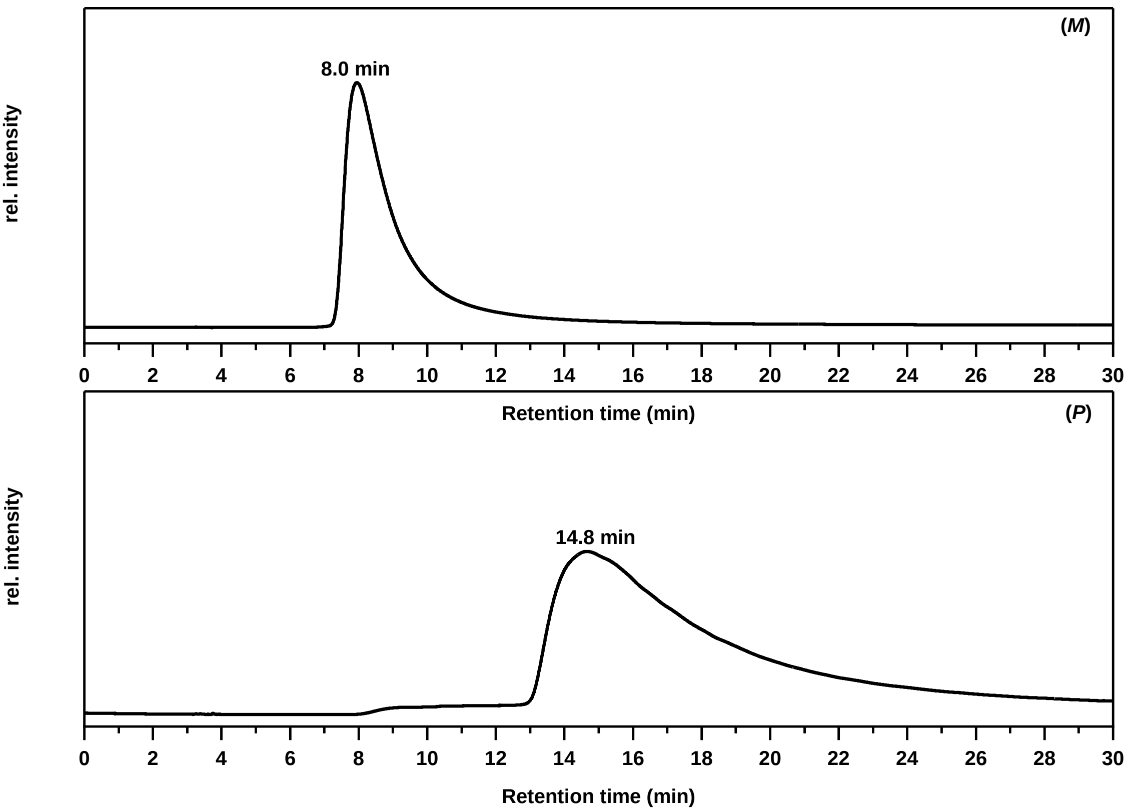

Figure S89. Analytical chromatograms of the two separated enantiomers of compound **9**. (IC column, *n*-heptane/DCM 96:4, 1 mL min^{-1}, 254 nm).

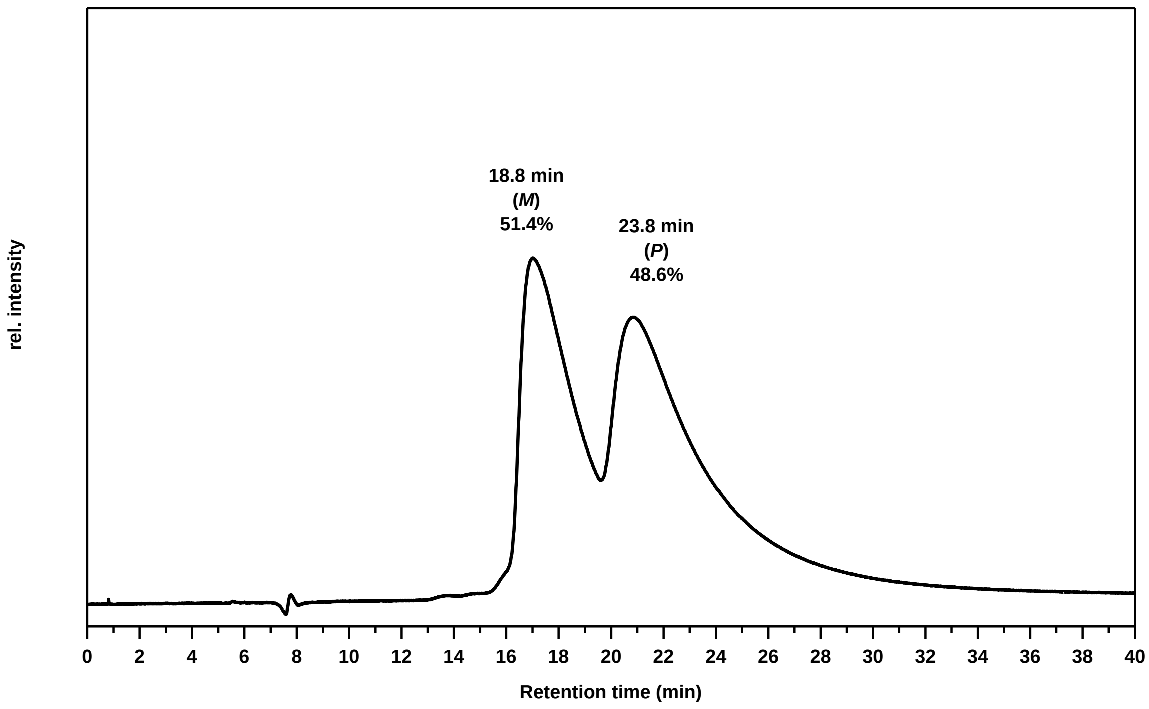

Figure S90. Preparative chromatogram of the racemic mixture of compound **10** (IA column, *n*-heptane/DCM/Et_3N 100:2:0.3, 10 mL min^{-1}, 254 nm).

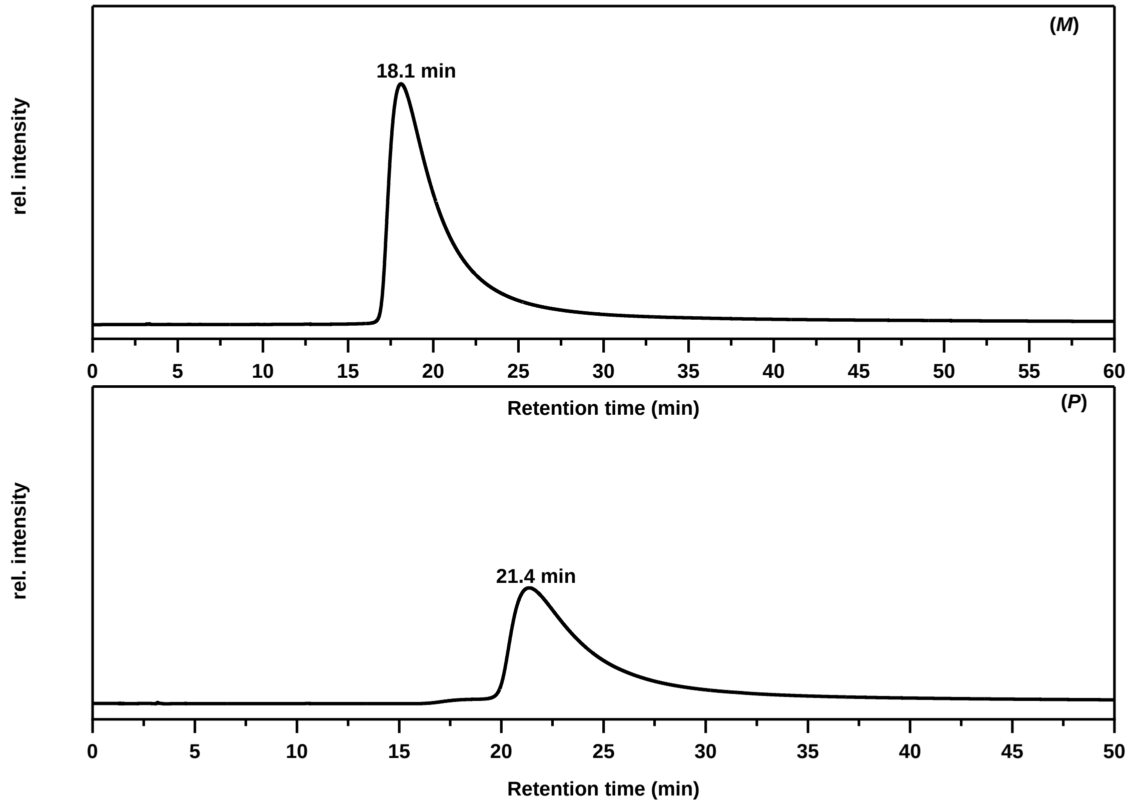

Figure S91. Analytical chromatograms of the two separated enantiomers of compound **10**. (IA column, *n*-heptane, 1 mL min^{-1}, 254 nm).

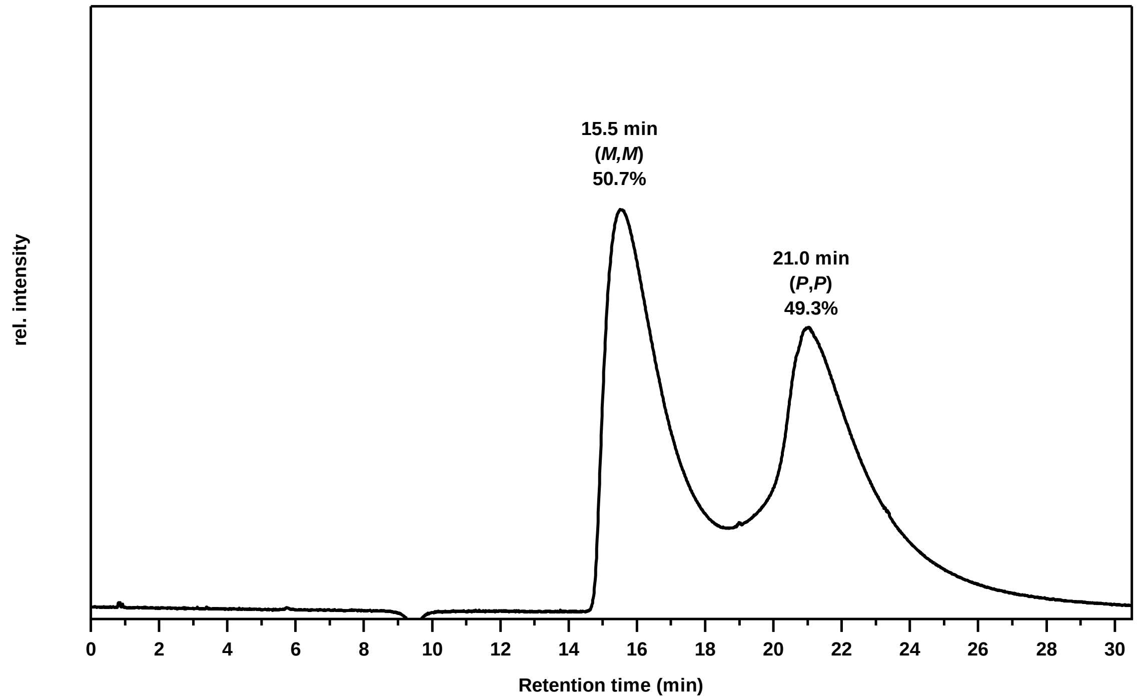

Figure S92. Preparative chromatogram of the racemic mixture of compound **11** (IA column, *n*-heptane/Et_3N 1000:3, 10 mL min^{-1}, 254 nm).

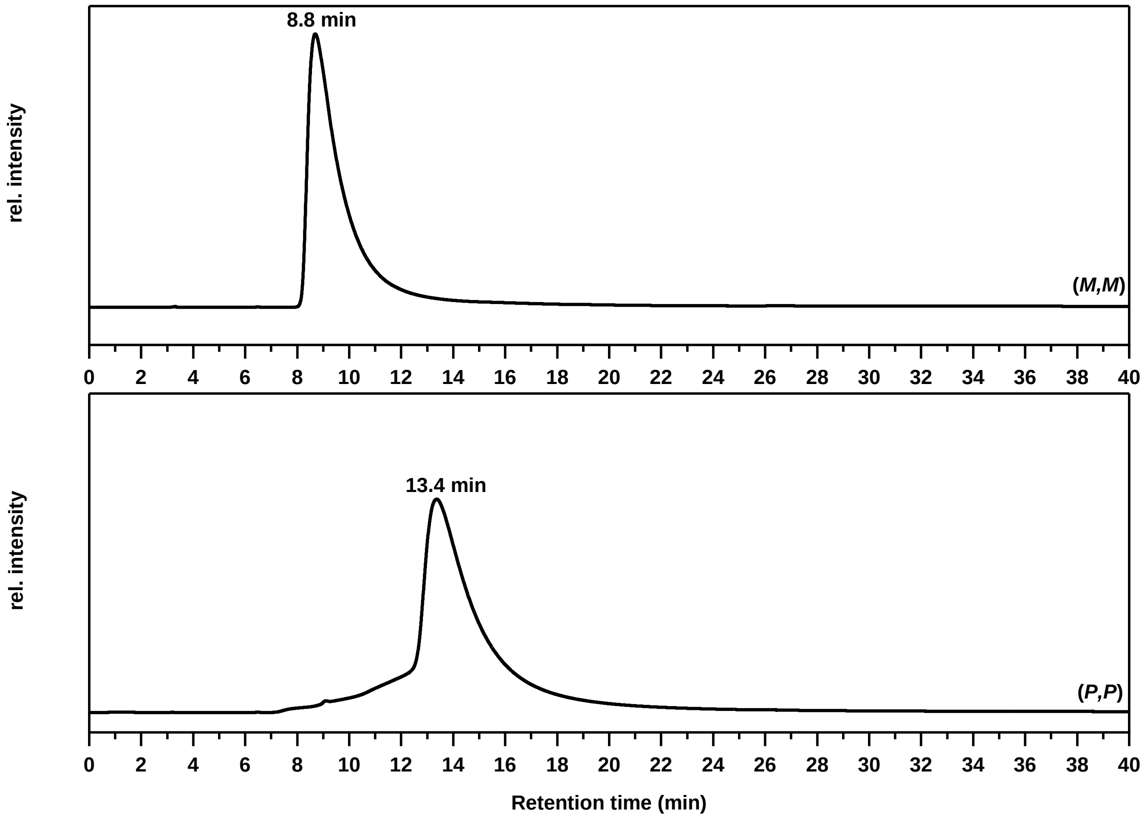

Figure S93. Analytical chromatograms of the two separated enantiomers of compound **11** (IA column, *n*-heptane, 1 mL min^{-1}, 254 nm).

9. Computational Details

9.1. Conformational Studies

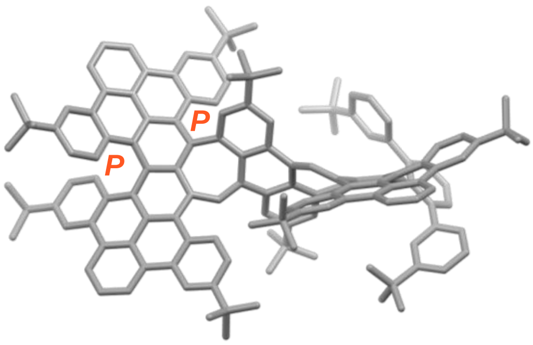

(*P*,*P*)-**9**
(0 kcal·mol^{-1})

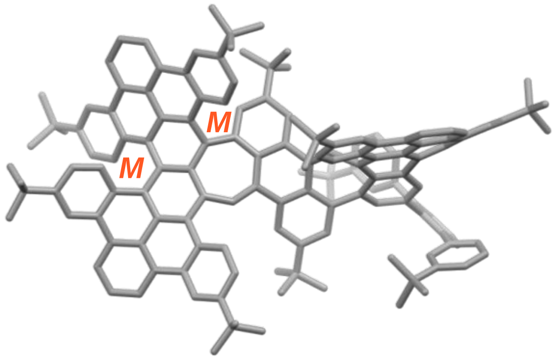

(*M*,*M*)-**9**
(19.40 kcal·mol^{-1})

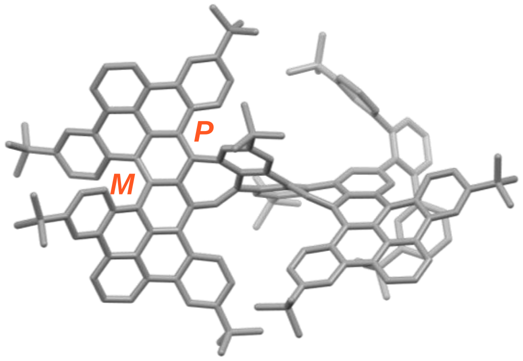

(*M*,*P*)-**9**
(6.29 kcal·mol^{-1})

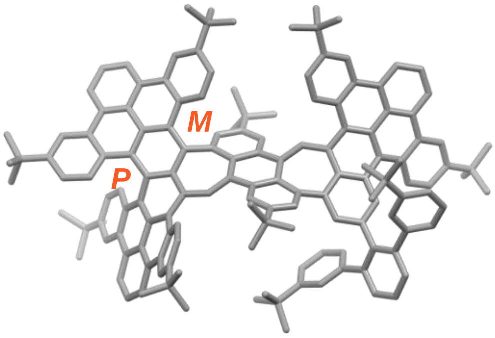

(*P*,*M*)-**9**
(14.92 kcal·mol^{-1})

Figure S94. Stereoisomers of compound **9** and their relative free energies calculated at the B3LYP/6-31G* level of theory. For simplification, only [6] and [5]-helicene units are considered during calculation. Please note here, (*P*,*P*)-**9** and (*M*,*M*)-**9** are not a pair of enantiomers due to different backbone and helicity of [4]helicenes.

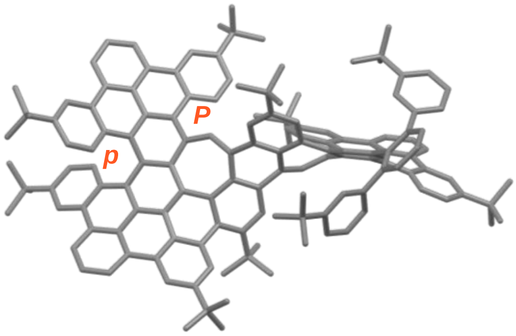

(*P*,*P*)-**10**
(0 kcal·mol^{-1})

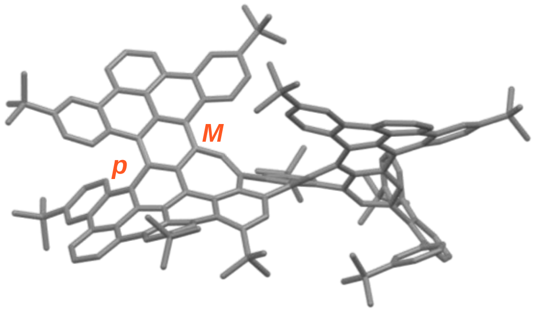

(*P*,*M*)-**10**
(8.58 kcal·mol^{-1})

Figure S95. Stereoisomers of compound **10** and their relative free energies calculated at the B3LYP/6-31G* level of theory. For simplification, only [6]- and [4]helicene units are considered during calculation.

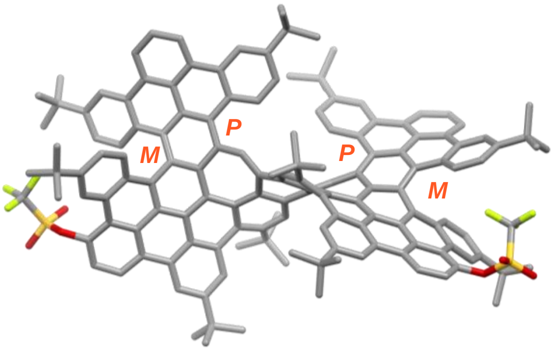

(M,PP,M)-**11**
(0 kcal·mol^{-1})

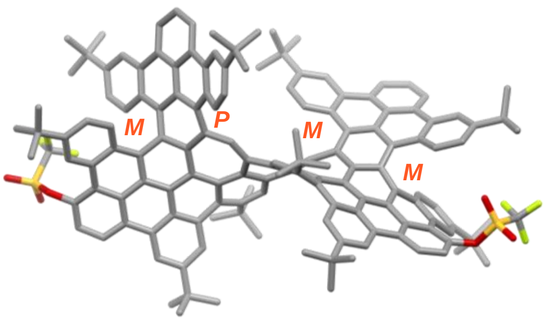

(M,PM,M)-**11**
(3.80 kcal·mol^{-1})

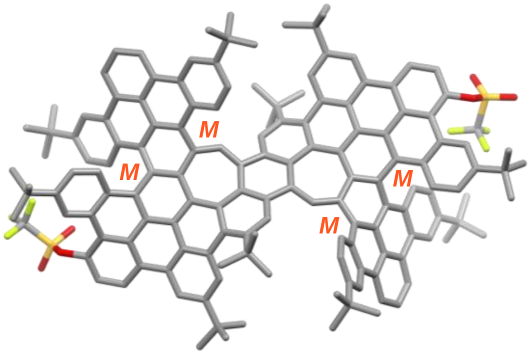

(M,MM,M)-**11**
(10.55 kcal·mol^{-1})

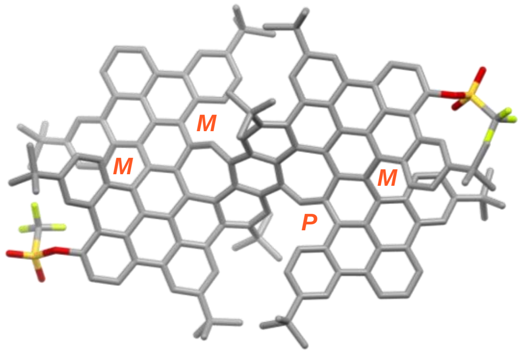

(M,MP,M)-**11**
(6.21 kcal·mol^{-1})

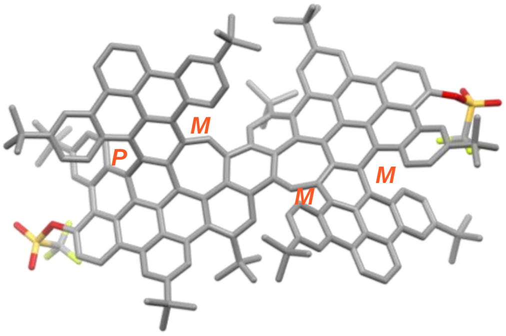

(P,MM,M)-**11**
(16.91 kcal·mol^{-1})

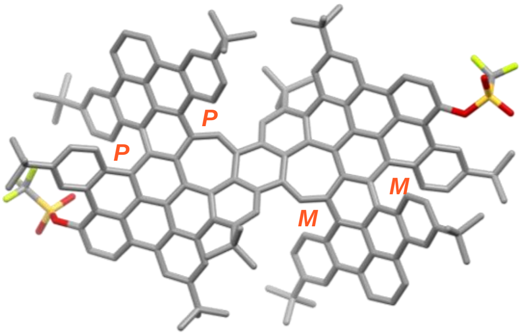

(P,PM,M)-**11**
(9.40 kcal·mol^{-1})

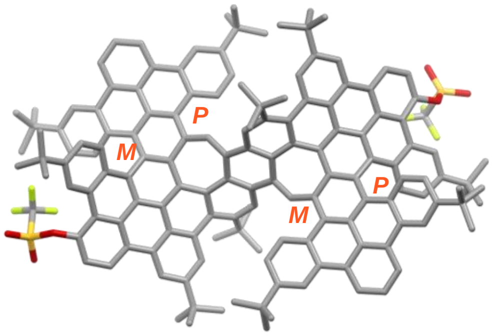

(M,PM,P)-**11**
(9.50 kcal·mol^{-1})

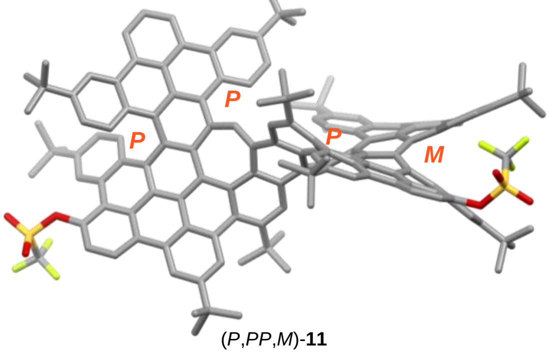

(P,PP,M)-**11**
(3.45 kcal·mol^{-1})

Figure S96. Stereoisomers of compound **11** and their relative free energies calculated at the B3LYP/6-31G* level of theory. For simplification, only [6]- and [4]helicene units are permuted.

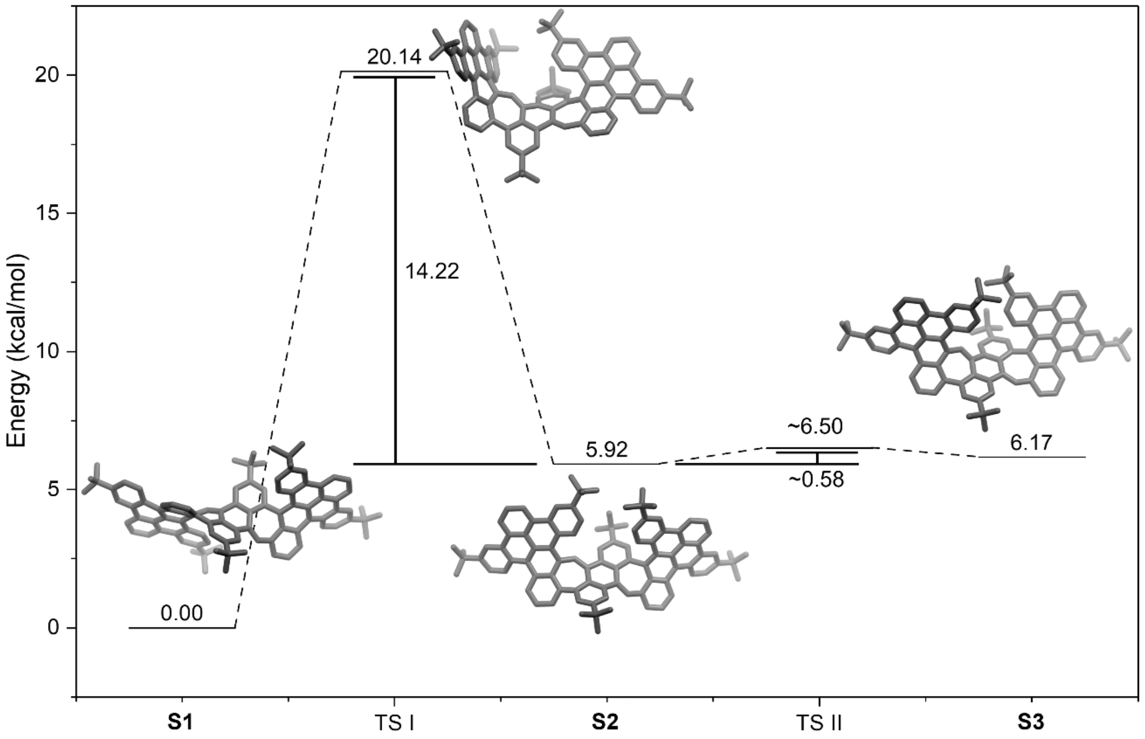

Figure S97. Energy profile (calculated at PM6 level of theory[S9]) for the interconversion of a simplified version of compound **9** (S.m.) to a conformer with inverted backbone (Flipped). Similar to the conversion from the (*P*,*P*,*P*,*P*) conformer to the (*P*,*P*,*P*,*M*) conformer of compound **9**. The transition state has an imaginary frequency of 16.1 cm^{-1}. Since the relevant direction of the isomerization is from **S2** to **S1**, this corresponds to a reaction barrier of 14.22 kcal/mol.

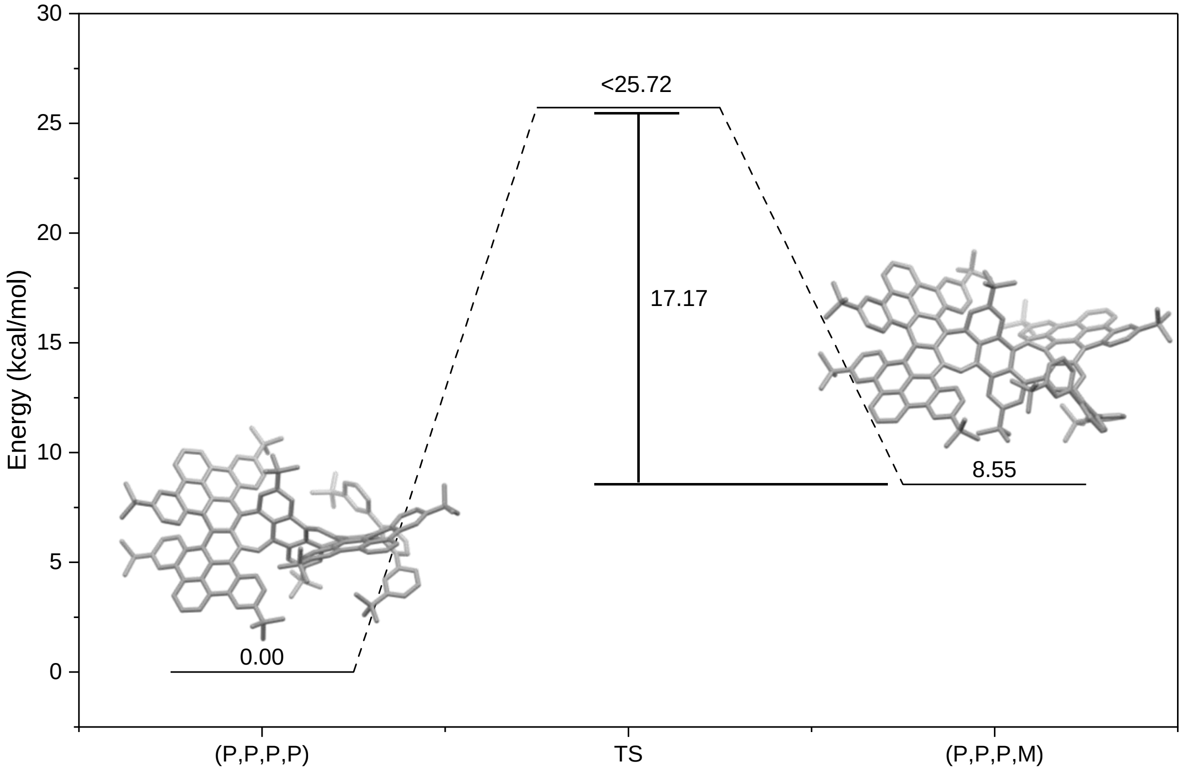

Figure S98. Energy profile (calculated at PM6 level of theory) of the interconversion of the (*P*,*P*,*P*,*P*) conformer to the (*P*,*P*,*P*,*M*) conformer of compound **9**. The energy of the transition state was estimated from a scan along a dihedral angle in the backbone of compound **9** (see also Figure S99). Since the relevant direction of the isomerization is from (*P*,*P*,*P*,*M*) to (*P*,*P*,*P*,*P*), this gives an upper bound for the reaction barrier of 17.17 kcal/mol.

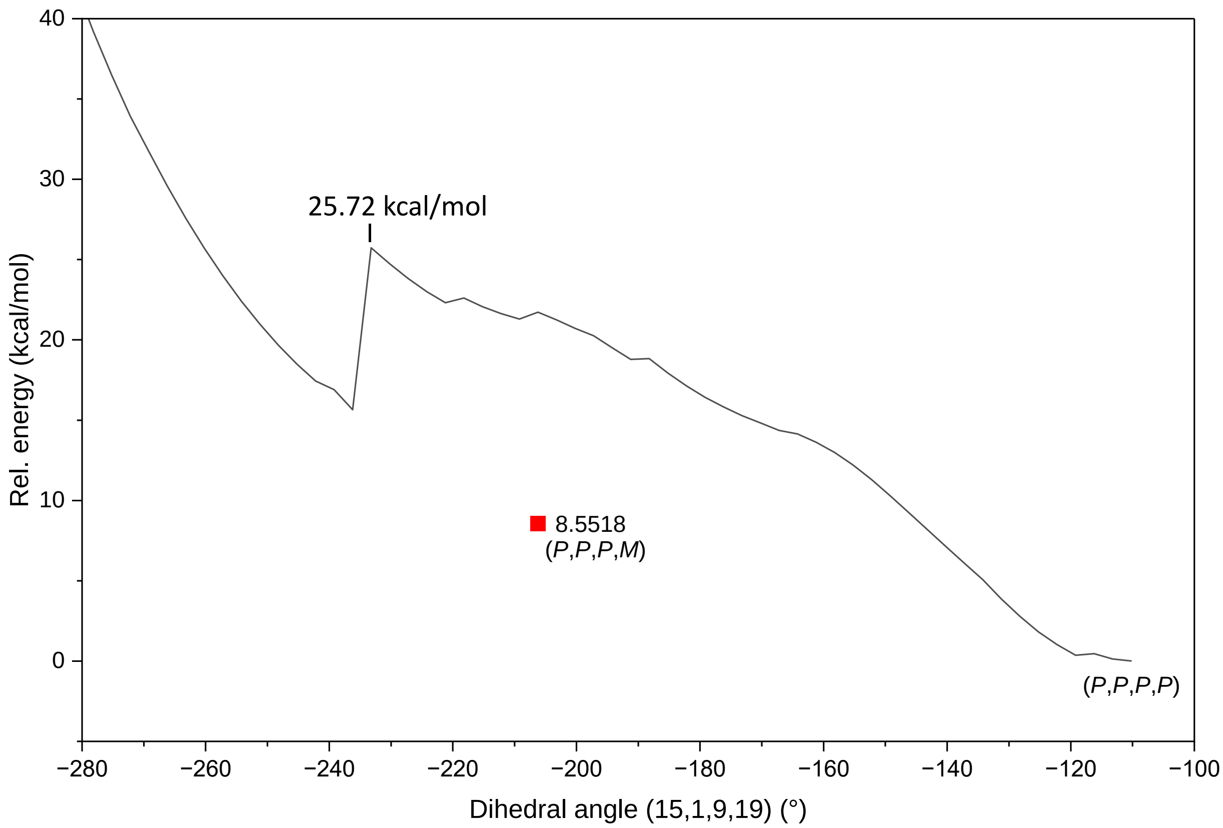

Figure S99. Energy along a scan of a dihedral angle in the backbone of compound **9** (at PM6 level of theory) starting at the (*P*,*P*,*P*,*P*) conformer. The highest energy point is marked at 25.72 kcal·mol^{-1} relative to the (*P*,*P*,*P*,*P*) conformer. If the constraint of the scan is released after the highest energy geometry, the structure optimizes into the (*P*,*P*,*P*,*M*) conformer with an inverted backbone geometry.

9.2. Frontier Molecular Orbitals

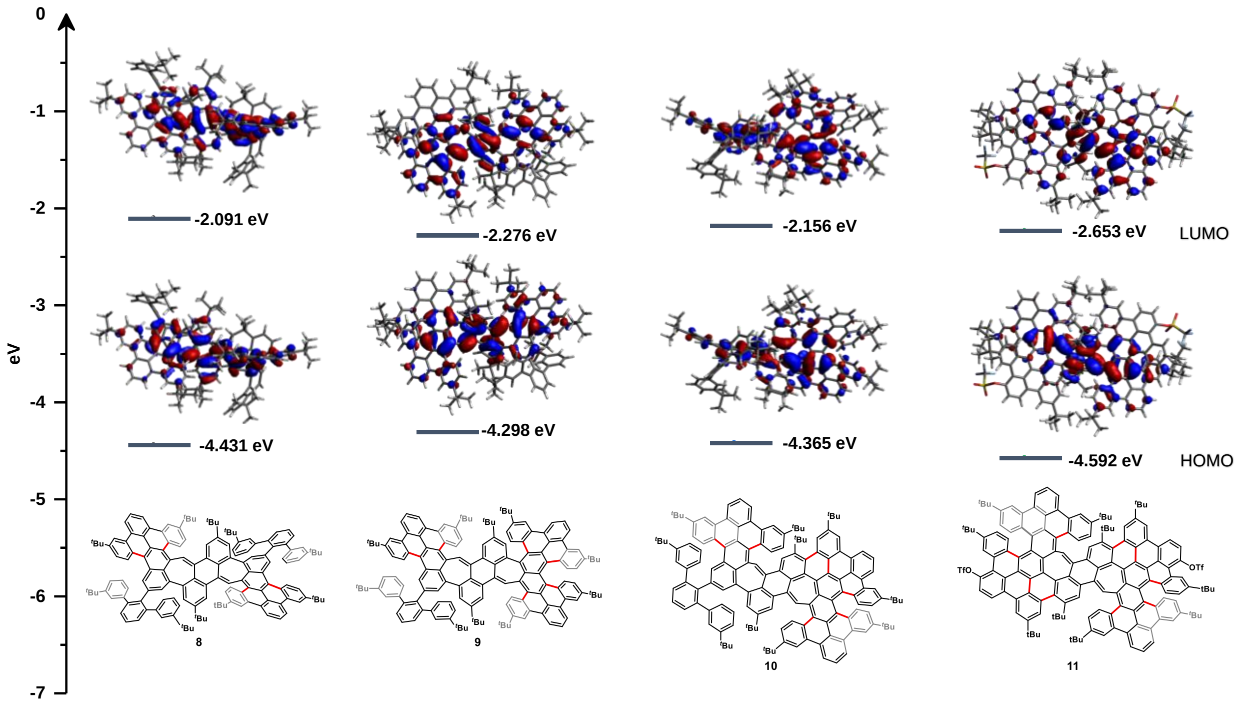

Figure S100. The frontier molecular orbitals of compounds **8**–**11** calculated at the B3LYP/6-31G* level of theory. **8**: (*P,P*)-conformer. **9**: (*P,P,P,M*)-conformer. **10**: (*P,P*)-conformer. **11**: (*M,M,P,M*)-conformer.

9.3. TD-DFT Calculations

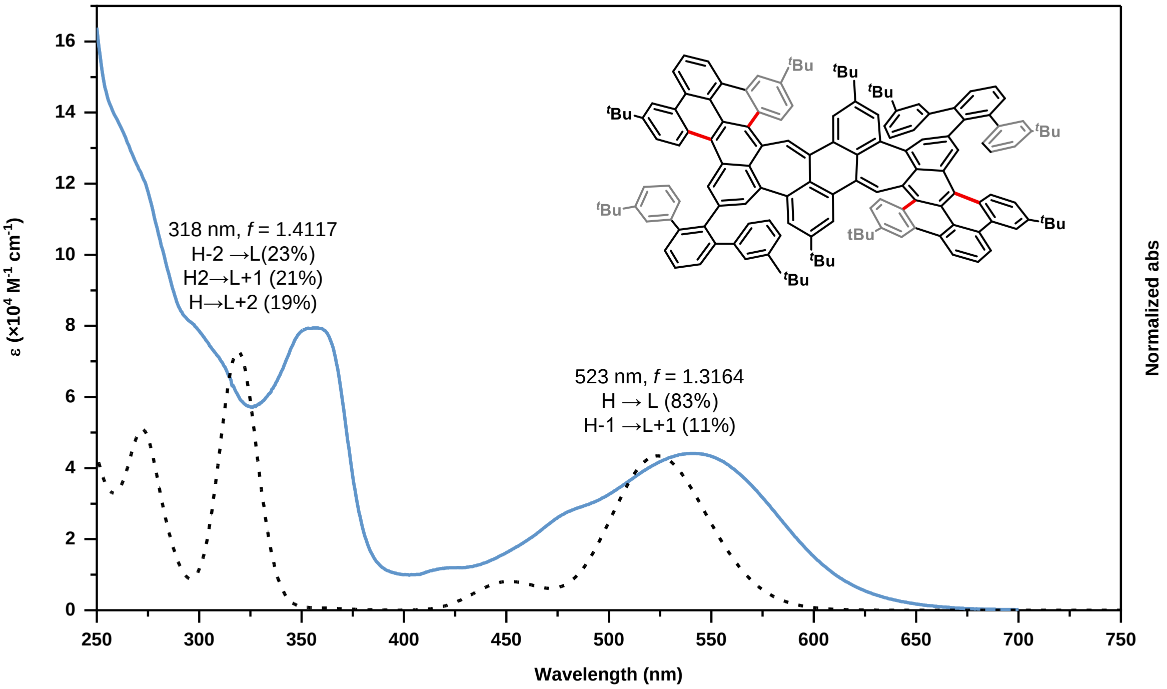

Figure S101. Comparison of UV-vis absorption spectrum measured in CH_2Cl_2 (solid) and calculated UV-vis absorption spectrum (dash) of compound **8** based on TDDFT at the CAM-B3LYP/6-31G*(SCRF- CH_2Cl_2) level of theory.

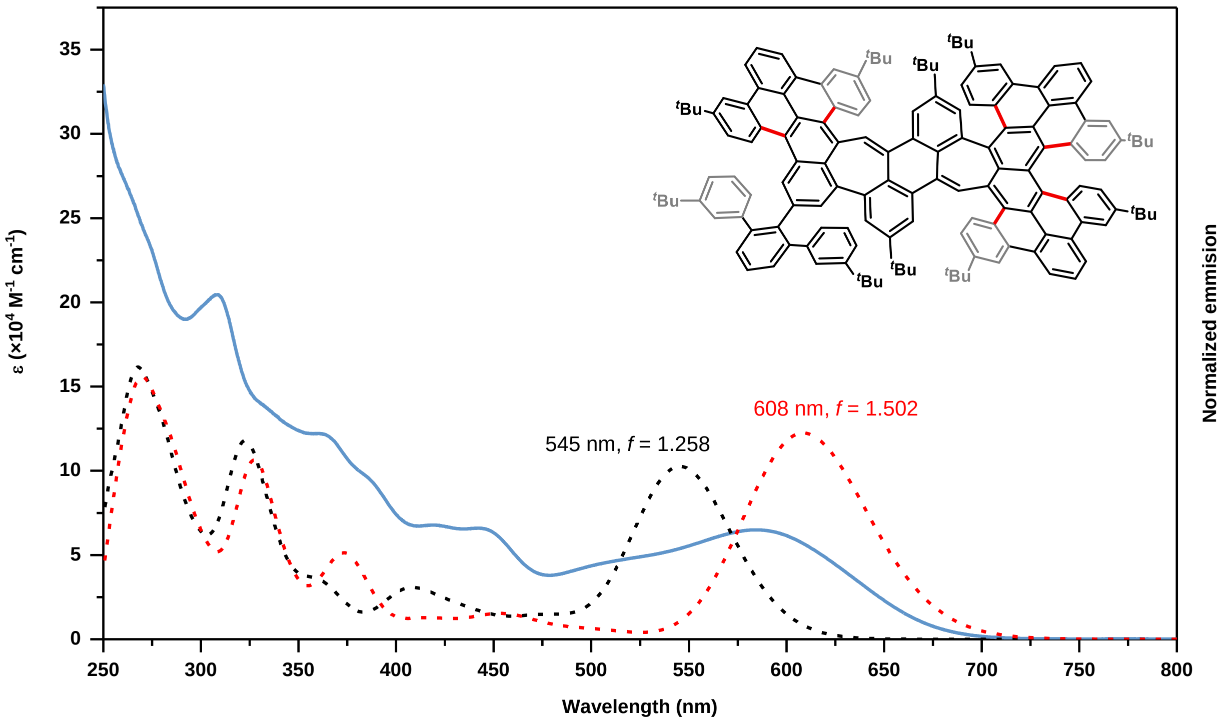

Figure S102. Comparison of UV-vis absorption spectrum measured in CH_2Cl_2 (solid) and calculated UV-vis absorption spectra (CAM-B3LYP/6-31G*, SCRF-CH_2Cl_2) of compound **9**, with a red dashed line for the (*P,P,P,M*)-conformer and a black dashed line for the (*P,P,P,P*)-conformer.

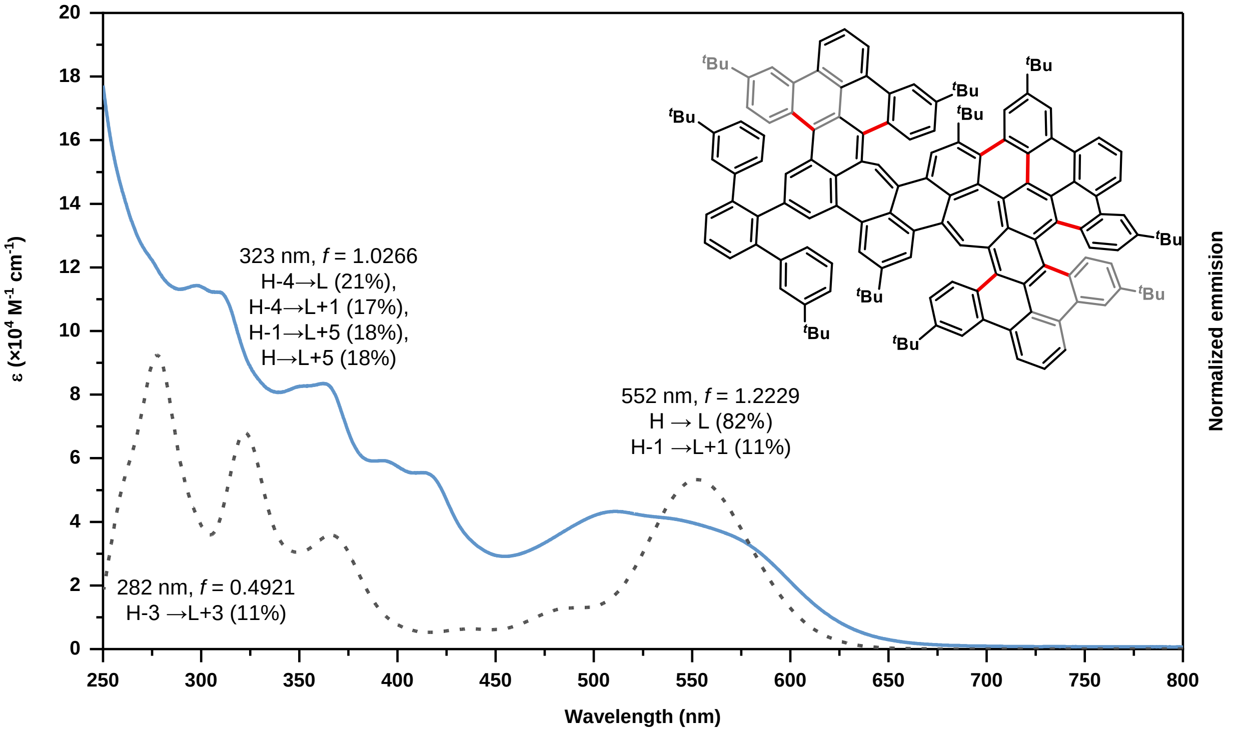

Figure S103. Comparison of experimental (solid, measured in CH_2Cl_2) and TDDFT (CAM-B3LYP/6-31G*, SCRF-CH_2Cl_2) calculated (dash) UV-vis absorption spectra of compound **10** along with assignments of key transitions. H = HOMO, L = LUMO, *f* = oscillator strength.

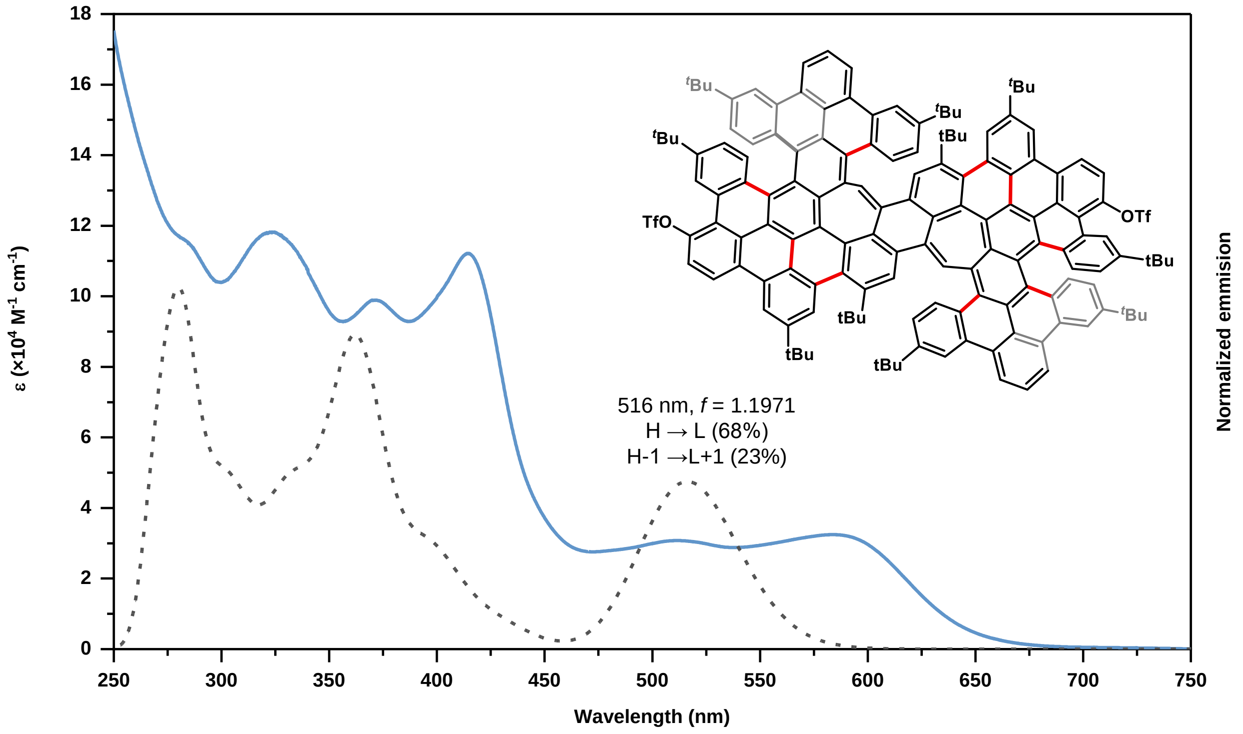

Figure S104. Comparison of UV-vis absorption spectrum measured in CH_2Cl_2 (black) and calculated UV-vis absorption spectrum (dash) of compound **11** based on TDDFT at the CAM-B3LYP/6-31G*(SCRF- CH_2Cl_2) level of theory**.**

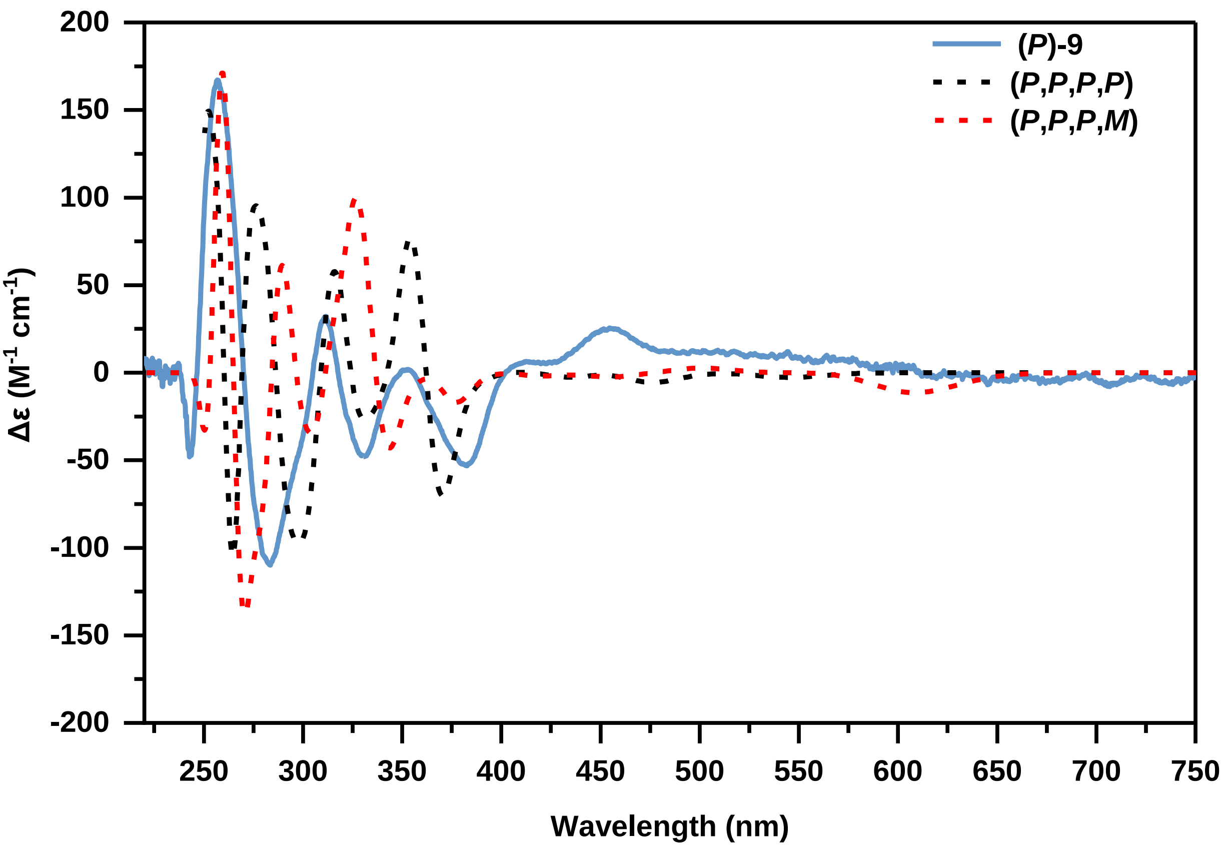

Figure S105. Comparison between the experimental CD spectrum (solid) of the second eluted fraction of **9** by chiral HPLC and calculated CD spectra (CAM-PBE1PBE/6-31G*, σ = 0.3 eV) of compound **9**, with a red dashed line for the (*P*,*P*,*P*,*M*)-conformer and a black dashed line for the (*P*,*P*,*P*,*P*)-conformer.

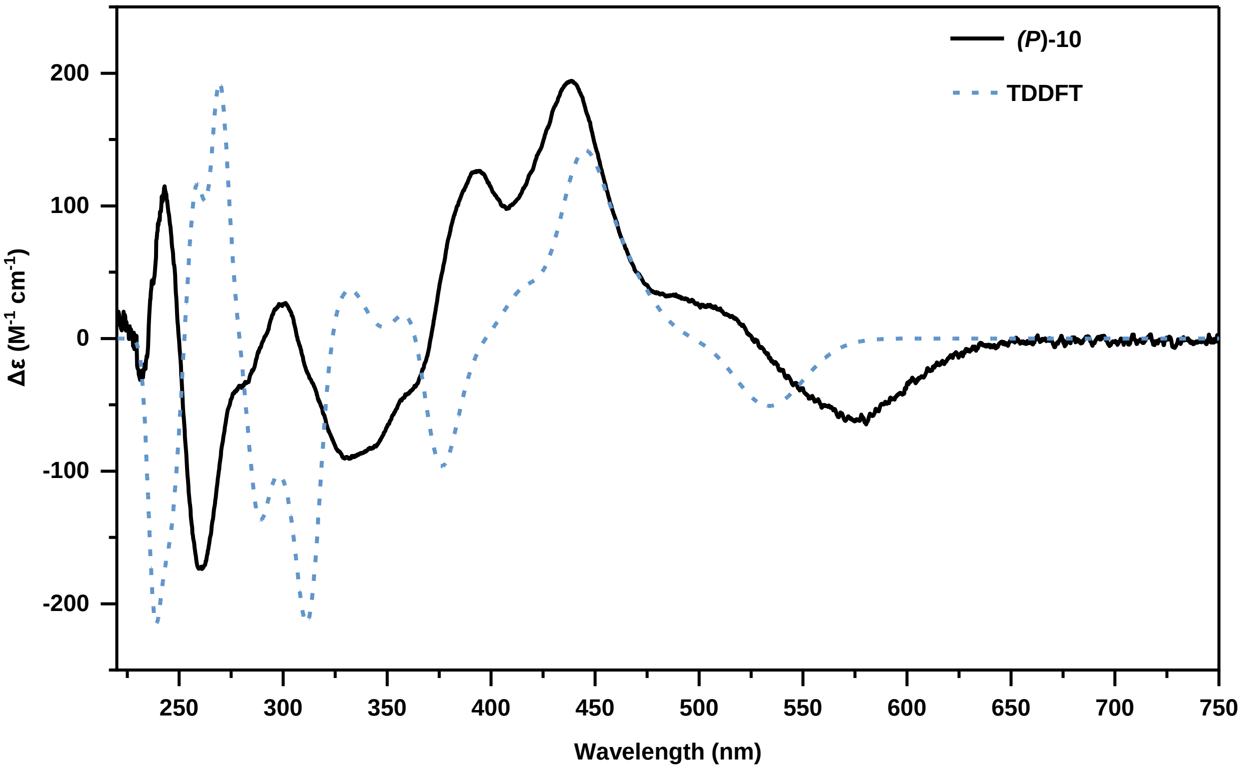

Figure S106. Comparison between the experimental CD spectrum (solid) of the second eluted fraction of **10** by chiral HPLC and calculated CD spectrum (dash, CAM-PBE1PBE/6-31G*, σ = 0.3 eV) for the *P* enantiomer of compound **10**.

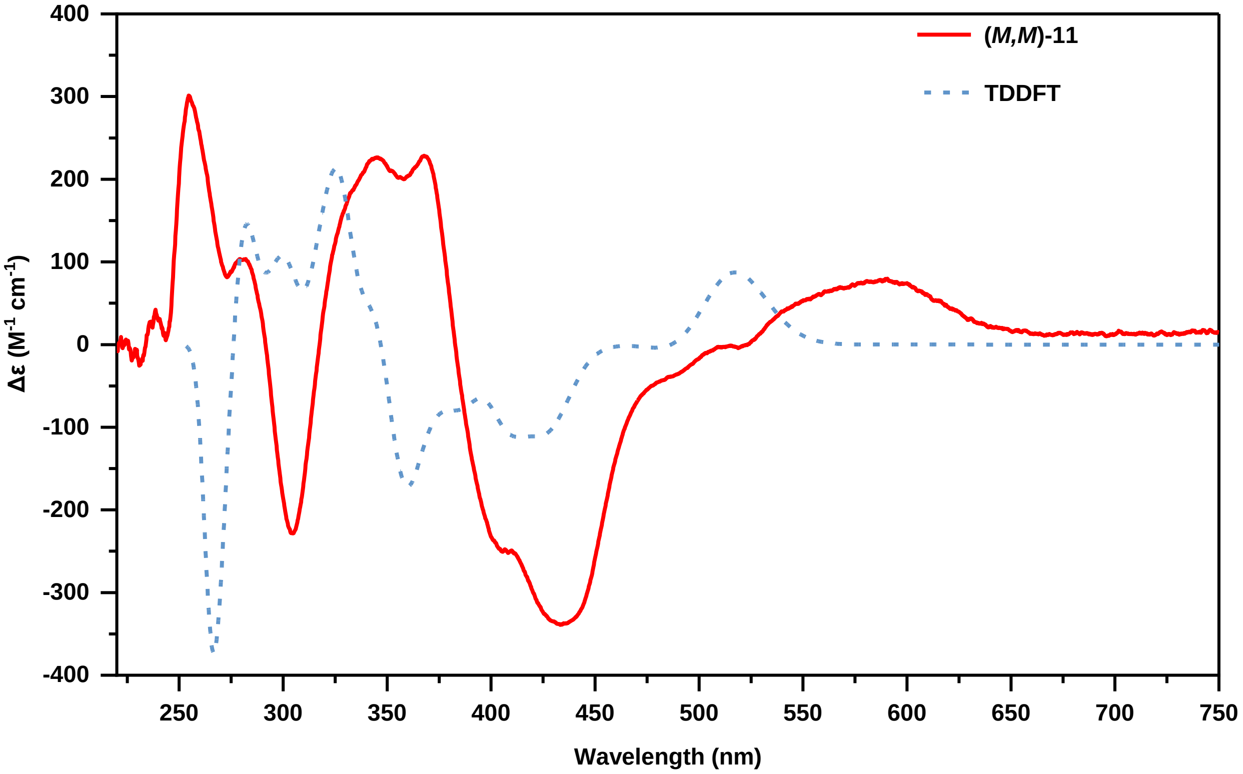

Figure S107. Comparison between the experimental CD spectrum (solid) of the first eluted fraction of **11** by chiral HPLC and calculated CD spectrum (dash, CAM-PBE1PBE/6-31G*, σ = 0.3 eV) for the (*M*,*M*) enantiomer of compound **11**.

9.4. Nucleus-independent Chemical Shift (NICS)

NICS(0), and NICS (1) and NICS(-1) values were calculated using Hartree-Fock GIAO methods (HF/6-31G*) after DFT-optimization (B3LYP/6-31G*) of the corresponding molecules level of theory. NICS(0) and NICS(±1) values were obtained from the optimized geometries by adding ghost atoms in the center and 1 Å above and below the center of the corresponding ring, respectively.

Table S1. DFT calculated NICS(0), and NICS (1) and NICS(-1) values for compound **8**. The molecular model indicates the number of each ring and from which side the NICS(+1) and NICS(-1) are defined.

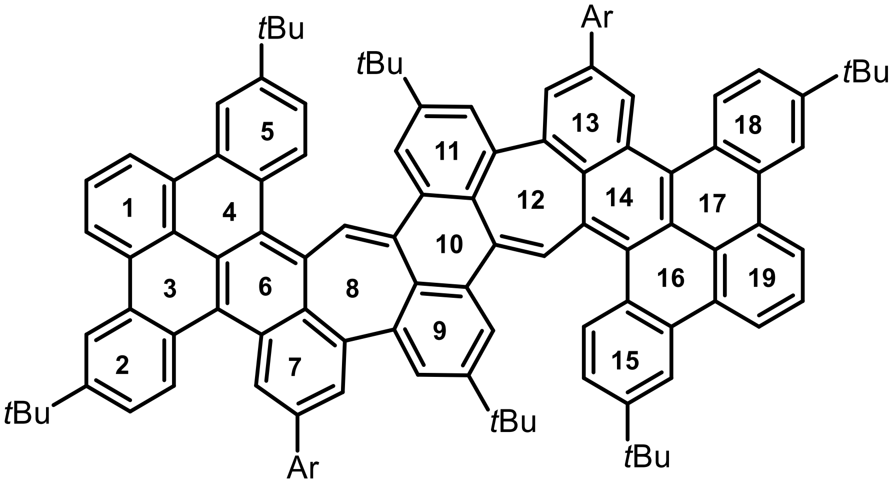

Ar = 3-*tert*-butylphenyl

Ring	NICS(0)	NICS(1)	NICS(-1)	NICS(1)av
1	-11.3	-12.8	-13.2	-13.0
2	-11.0	-13.8	-11.4	-12.6
3	-2.5	-6.8	-5.5	-6.15
4	0.3	-3.2	-4.1	-3.65
5	-9.8	-11.3	-12.0	-11.65
6	-8.6	-11.4	-10.7	-11.05
7	-9.8	-9.7	-12.8	-11.25
8	7.3	2.0	3.0	2.5
9	-9.5	-11.2	-10.7	-10.95
10	2.8	-1.3	-0.7	-1.0
11	-9.5	-11.3	-10.6	-10.95
12	6.8	1.1	2.8	1.95
13	-11.4	-9.1	-10.3	-9.7
14	-8.6	-12.3	-9.6	-10.95
15	-10.2	-11.0	-12.7	-11.85
16	1.1	-3.0	-3.4	-3.2
17	-2.1	-5.6	-6.2	-5.9
18	-11.1	-11.5	-13.9	-12.7
19	-11.1	-13.2	-12.3	-12.75

Table S2. DFT calculated NICS(0), and NICS (1) and NICS(-1) values for compound **9**. The molecular model indicates the number of each ring.

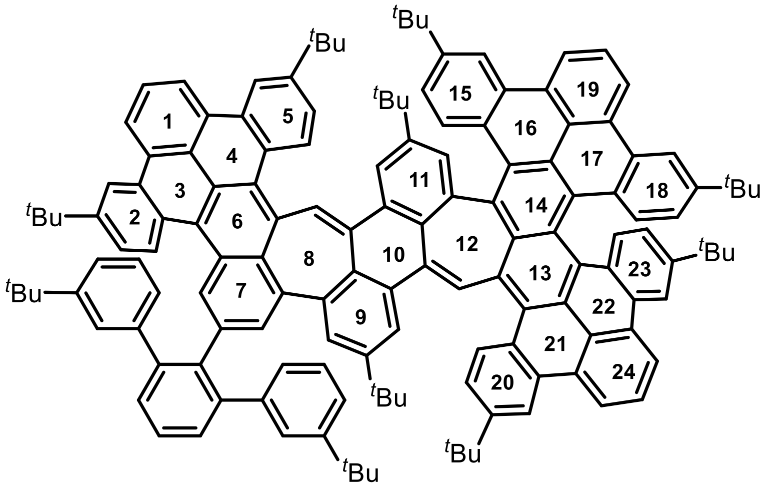

Ring	NICS(0)	NICS(1)	NICS(-1)	NICS(1)av
1	-10.4	-12.3	-12.7	-12.5
2	-9.5	-12.6	-10.4	-11.5
3	-2.5	-6.9	-5.7	-6.3
4	-0.9	-3.0	-3.7	-3.35
5	-8.8	-10.6	-11.3	-10.95
6	-8.1	-11.0	-10.4	-10.7
7	-9.3	-9.3	-12.4	-10.85
8	7.5	2.1	3.2	2.65
9	-8.3	-10.3	-9.8	-10.05
10	3.6	-0.2	-0.0	-0.1
11	-8.7	-10.1	-10.3	-10.2
12	7.5	2.7	2.4	2.55
13	-8.0	-12.0	-8.9	-10.45
14	-7.0	-8.6	-11.1	-9.85
15	-8.7	-11.3	-10.0	-10.65
16	1.7	-3.3	-1.7	-2.5
17	-1.9	-3.8	-6.8	-5.3
18	-10.2	-11.4	-13.0	-12.2
19	-9.9	-11.9	-12.1	-12.0
20	-8.7	-10.0	-11.3	-10.65
21	1.7	-1.7	-3.3	-2.5
22	-1.9	-6.8	-3.8	-5.3
23	-10.2	-13.0	-11.4	-12.2
24	-9.9	-12.1	-11.9	-12.0

Table S3. DFT calculated NICS(0), and NICS (1) and NICS(-1) values for compound **10**. The molecular model indicates the number of each.

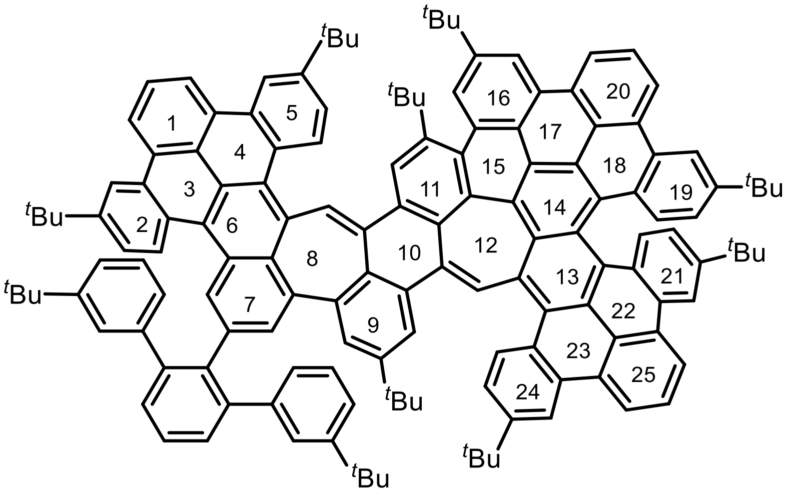

Ring	NICS(0)	NICS(1)	NICS(-1)	NICS(1)av
1	-11.2731	-12.6991	-13.1137	-12.9
2	-10.8796	-13.5160	-11.4135	-12.5
3	-2.5978	-6.7665	-5.7044	-6.2
4	0.6921	-2.8668	-3.7557	-3.3
5	-9.8891	-11.3954	-11.9829	-11.7
6	-8.4953	-11.2126	-10.6516	-10.9
7	-9.7988	-9.7071	-12.7624	-11.2
8	7.4610	1.9502	3.2837	2.6
9	-9.5253	-11.3129	-10.6151	-11.0
10	2.6508	-2.0564	-0.1281	-1.1
11	-8.2732	-10.5393	-9.7954	-10.2
12	9.6888	3.9378	5.4577	4.7
13	-8.0049	-11.9993	-9.3499	-10.7
14	-8.8608	-9.7567	-12.2411	-11.0
15	0.0656	-4.1283	-4.2289	-4.2
16	-10.9501	-13.1488	-11.8415	-12.5
17	1.9316	-2.1019	-2.8058	-2.5
18	-1.4934	-3.8834	-6.4657	-5.2
19	-10.8703	-11.7088	-13.4211	-12.6
20	-10.7615	-12.4610	-12.3337	-12.4
21	-10.8441	-13.3829	-11.7177	-12.6
22	-2.5598	-7.5191	-4.3008	-5.9
23	0.8304	-2.9281	-3.4873	-3.2
24	-9.7699	-11.1210	-11.9958	-11.6
25	-11.1018	-12.8404	-12.6286	-12.7

Table S4. DFT calculated NICS(0), and NICS (1) and NICS(-1) values for compound **11**. The molecular model indicates the number of each ring.

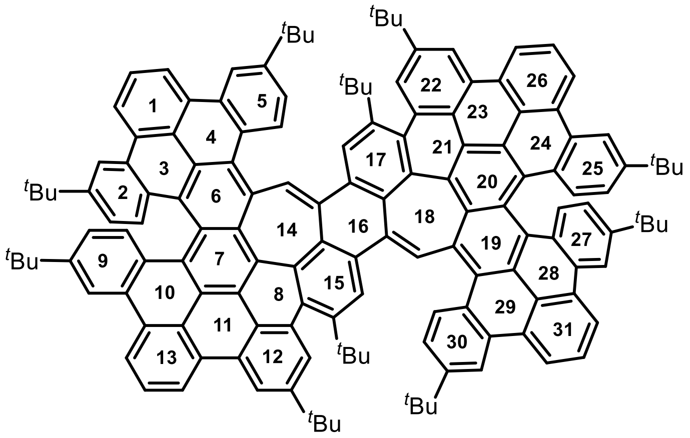

Ring	NICS(0)	NICS(1)	NICS(-1)	NICS(1)av
1	-10.0	-12.0	-12.2	-12.1
2	-10.1	-11.5	-12.8	-12.15
3	-1.9	-4.1	-6.8	-5.45
4	1.0	-3.7	-2.7	-3.2
5	-8.7	-11.6	-10.0	-10.8
6	-6.5	-8.7	-10.5	-9.6
7	-8.1	-11.8	-8.9	-10.35
8	-0.4	-4.0	-4.8	-4.4
9	-10.4	-13.3	-11.0	-12.15
10	-0.9	-6.1	-3.1	-4.6
11	2.4	-2.4	-1.9	-2.15
12	-10.1	-11.4	-12.3	-11.85
13	-11.0	-11.2	-11.8	-11.5
14	10.6	5.9	5.3	5.6
15	-6.9	-8.6	-9.3	-8.95
16	4.6	0.7	1.2	0.95
17	-7.2	-9.8	-8.9	-9.35
18	9.4	3.9	4.5	4.2
19	-6.2	-9.2	-9.1	-9.15
20	-6.1	-8.7	-9.9	-9.3
21	-1.1	-4.4	-5.3	-4.85
22	-9.9	-12.6	-10.8	-11.7
23	3.0	-1.4	-1.5	-1.45
24	-1.8	-6.6	-3.4	-5.0
25	-10.5	-13.4	-11.3	-12.35
26	-11.3	-11.7	-11.9	-11.8
27	-10.4	-11.3	-13.2	-12.25
28	-1.2	-3.9	-6.0	-4.95
29	1.4	-2.3	-3.5	-2.9
30	-9.3	-10.3	-12.4	-11.35
31	-9.9	-12.2	-11.7	-11.95

9.5. Anisotropy of the Induced Current Density (AICD)

AICD was calculated using Hartree-Fock methods (HF/6-31G*)[S23-30] after DFT optimization (B3LYP/6-31G*) of the corresponding molecules. Using multiwfn, π-orbitals were detected and the occupation of other orbitals set to zero. Visualization was achieved with an isosurface value of 0.050.[S19] The diamagnetic (clockwise) ring currents are highlighted by red arrows.

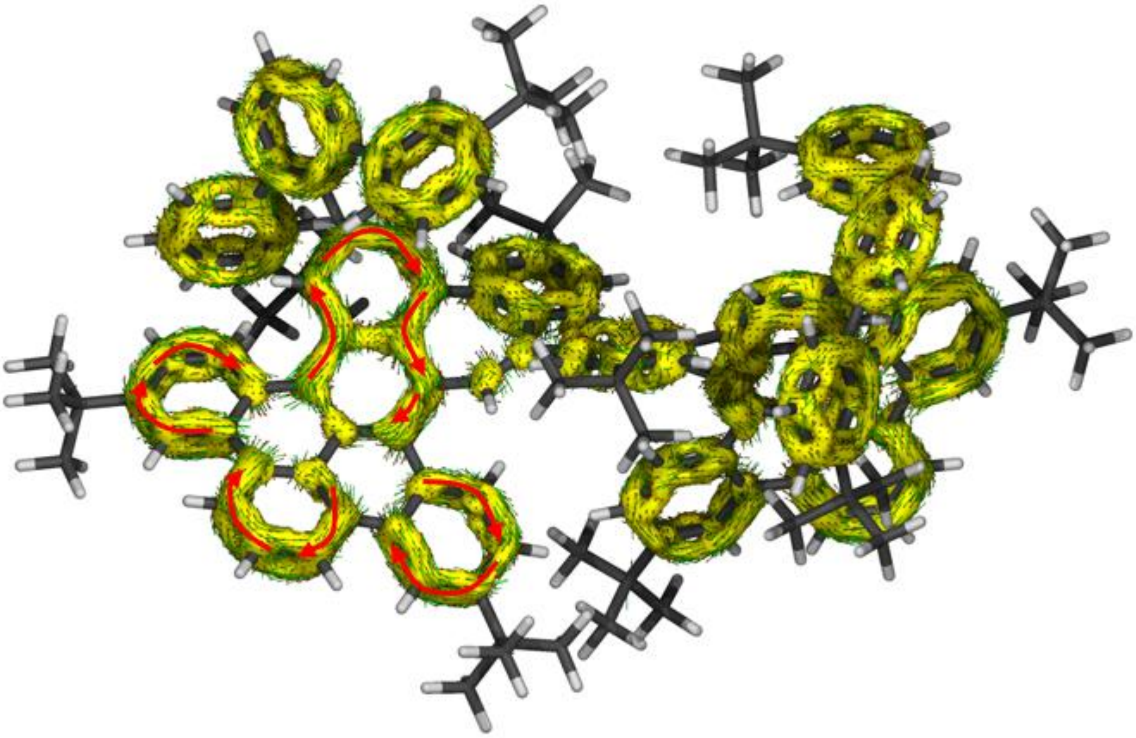

Figure S108. The calculated AICD plots for compound **8**.

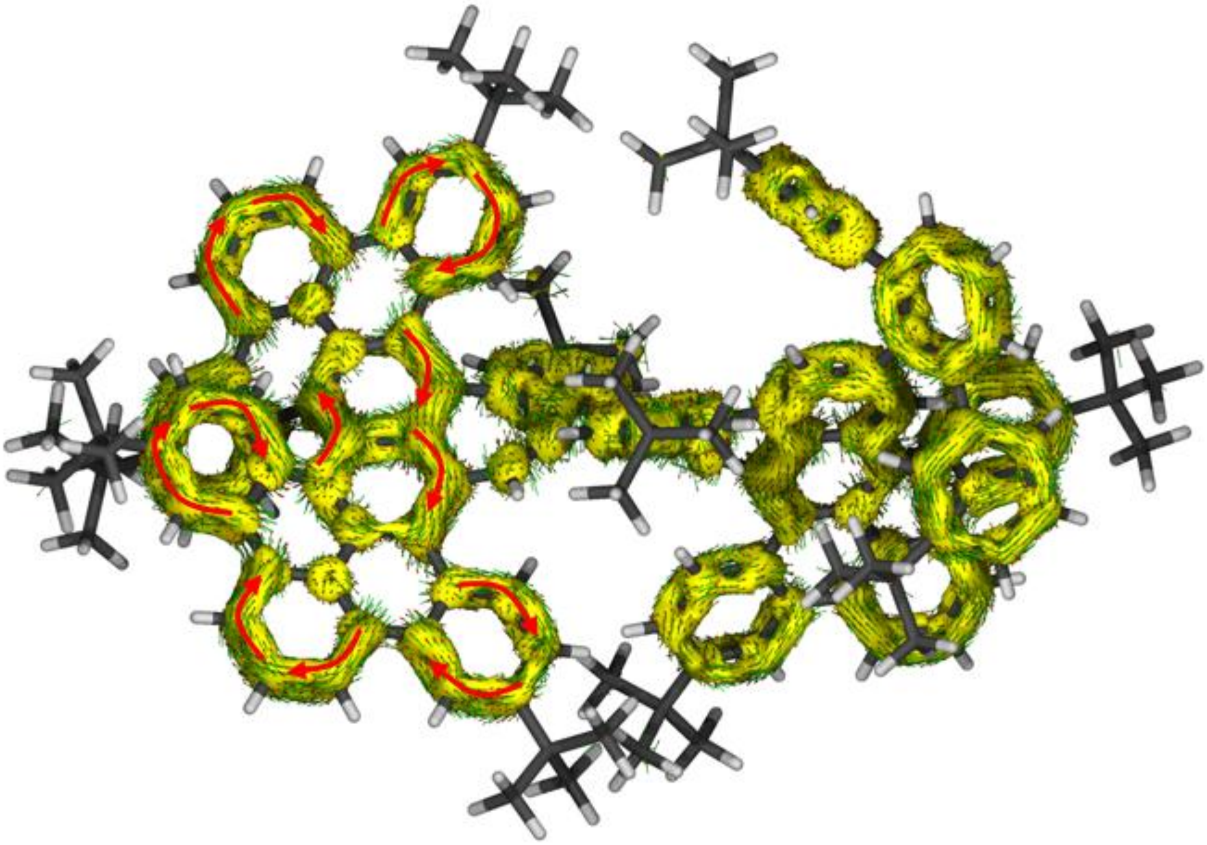

Figure S109. The calculated AICD plots for compound **9**.

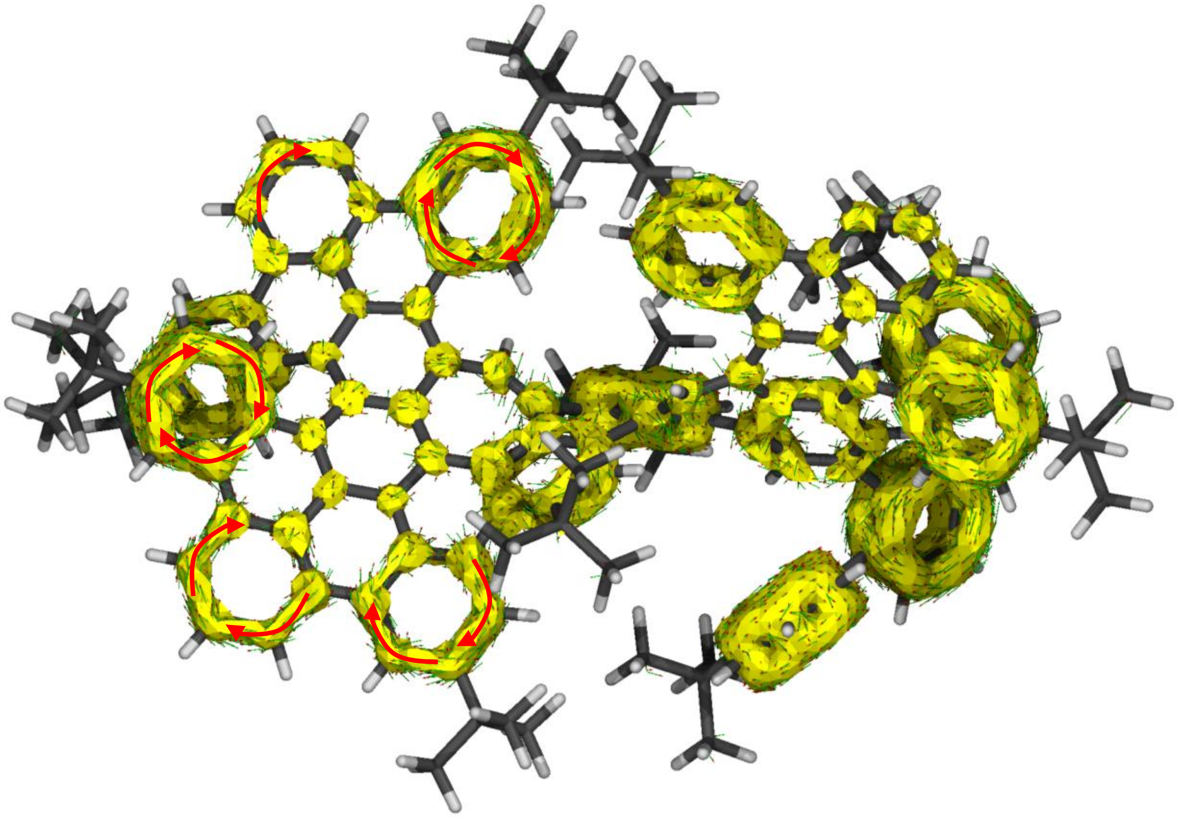

Figure S110. The calculated AICD plots for compound **10**.

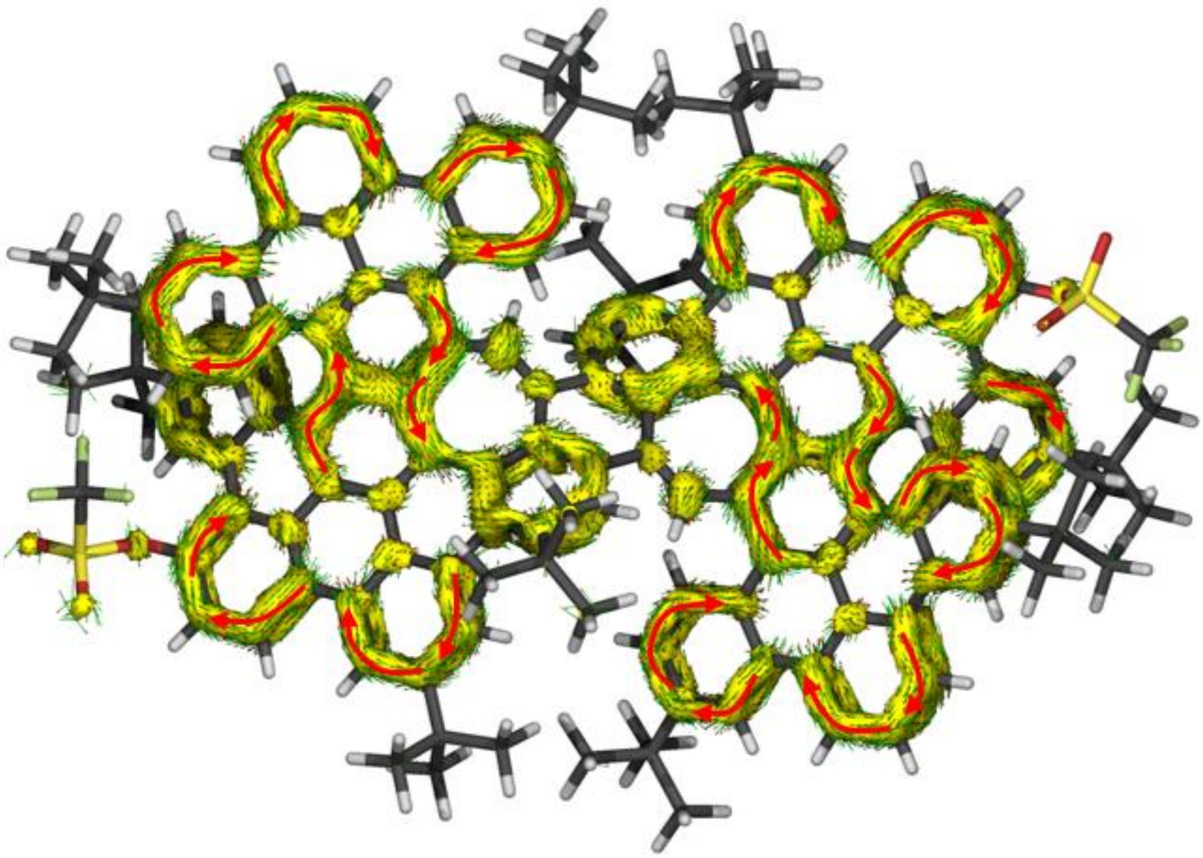

Figure S111. The calculated AICD plots for compound **11**.

10. Crystallographic Data

Compound 4 (CCDC: 2515109)

Crystals suitable for X-ray diffraction were obtained by vapor diffusion ($CHCl_3$/EtOH).

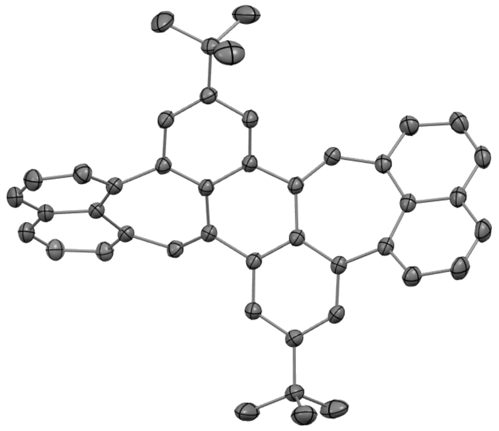

Empirical formula	$C_{44}H_{36}$	
Formula weight	564.73	
Temperature	200(2) K	
Wavelength	0.71073 Å	
Crystal system	triclinic	
Space group	P $\bar{1}$	
Z	2	
Unit cell dimensions	a =9.9123(5) Å	α =100.0095(8) deg.
	b = 11.5042(5) Å	β =92.8236(9) deg.
	c = 14.1255(6) Å	γ =104.0543(9) deg.
Volume	1531.70(12) Å^3	
Density (calculated)	1.22 g/cm^3	
Absorption coefficient	0.07 mm^{-1}	
Crystal shape	rhombic	
Crystal size	0.231 x 0.134 x 0.038 mm^3	
Crystal colour	orange	
Theta range for data collection	1.5 to 29.9 deg.	
Index ranges	$-13\leq h\leq 13$, $-15\leq k\leq 15$, $-18\leq l\leq 19$	
Reflections collected	31201	
Independent reflections	8160 (R_{int} = 0.0353)	
Observed reflections	5864 (I > 2σ(I))	
Absorption correction	Semi-empirical from equivalents	
Max. and min. transmission	0.96 and 0.93	
Refinement method	Full-matrix least-squares on F^2	
Data/restraints/parameters	8160 / 0 / 403	
Goodness-of-fit on F^2	1.04	
Final R indices (I>2sigma(I))	$R1$ = 0.055, w$R2$ = 0.146	
Largest diff. peak and hole	0.34 and -0.25 eÅ^{-3}	

Compound 5 (CCDC: 2515110)

Crystals suitable for X-ray diffraction were obtained by vapor diffusion (DCM/MeOH).

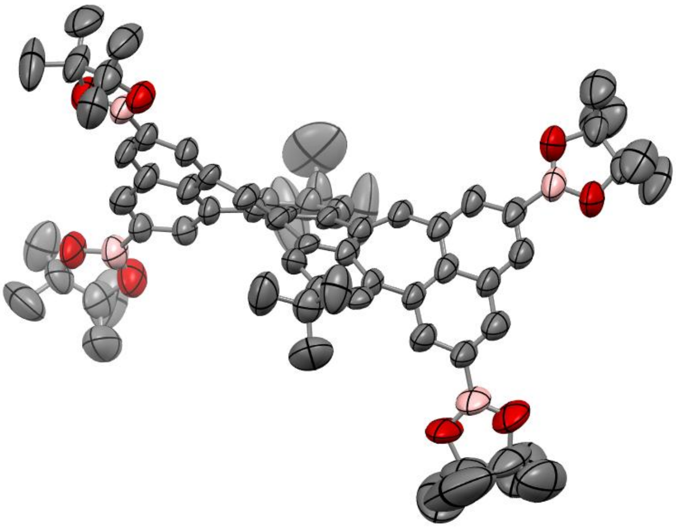

Empirical formula	$C_{68}H_{80}B_4O_8$	
Formula weight	1068.56	
Temperature	200(2) K	
Wavelength	1.54178 Å	
Crystal system	monoclinic	
Space group	$P2_1/n$	
Z	8	
Unit cell dimensions	a = 15.8110(9) Å	α = 90 deg.
	b = 28.775(3) Å	β =99.645(4) deg.
	c = 32.5966(19) Å	γ = 90 deg.
Volume	14620.6(17) $Å^3$	
Density (calculated)	0.97 g/cm^3	
Absorption coefficient	0.48 mm^{-1}	
Crystal shape	plank	
Crystal size	0.105 x 0.055 x 0.026 mm^3	
Crystal colour	orange	
Theta range for data collection	2.8 to 41.6 deg.	
Index ranges	$-13 \le h \le 13$, $-24 \le k \le 24$, $-28 \le l \le 20$	
Reflections collected	41127	
Independent reflections	9659 (R_{int} = 0.2205)	
Observed reflections	4056 (I > 2σ(I))	
Absorption correction	Semi-empirical from equivalents	
Max. and min. transmission	1.00 and 0.30	
Refinement method	Full-matrix least-squares on F^2	
Data/restraints/parameters	9659 / 4300 / 1441	
Goodness-of-fit on F^2	1.06	
Final R indices (I>2sigma(I))	$R1$ = 0.140, w$R2$ = 0.344	
Largest diff. peak and hole	0.30 and -0.22 $eÅ^{-3}$	

Compound 8 (CCDC: 2515111)

Crystals suitable for X-ray diffraction were obtained by layering technique (Hexane/MeOH 3/1).

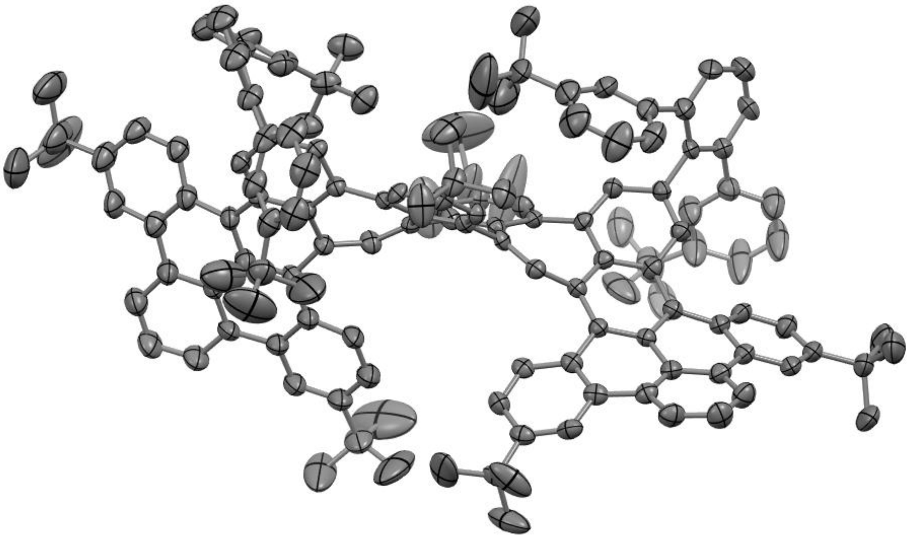

Empirical formula	$C_{148}H_{140}$	
Formula weight	1918.59	
Temperature	200(2) K	
Wavelength	1.54178 Å	
Crystal system	triclinic	
Space group	$P\bar{1}$	
Z	2	
Unit cell dimensions	a = 14.3610(8) Å	α =77.307(4) deg.
	b = 19.1601(10) Å	β =73.672(4) deg.
	c = 23.8792(14) Å	γ =83.309(4) deg.
Volume	6140.9(6) $Å^3$	
Density (calculated)	1.04 g/cm^3	
Absorption coefficient	0.44 mm^{-1}	
Crystal shape	brick	
Crystal size	0.090 x 0.064 x 0.040 mm^3	
Crystal colour	red	
Theta range for data collection	2.4 to 52.6 deg.	
Index ranges	$-14 \le h \le 14$, $-15 \le k \le 19$, $-24 \le l \le 24$	
Reflections collected	57594	
Independent reflections	14024 ($Rint$ = 0.1536)	
Observed reflections	8172 ($I > 2\sigma(I)$)	
Absorption correction	Semi-empirical from equivalents	
Max. and min. transmission	0.99 and 0.90	
Refinement method	Full-matrix least-squares on F^2	
Data/restraints/parameters	14024 / 2243 / 1397	
Goodness-of-fit on F^2	1.11	
Final R indices (I>2sigma(I))	$R1$ = 0.119, w$R2$ = 0.261	
Largest diff. peak and hole	0.67 and -0.28 $eÅ^{-3}$	

11. Reference

[S1] G. R. Fulmer, A. J. M. Miller, N. H. Sherden, H. E. Gottlieb, A. Nudelman, B. M. Stoltz, J. E. Bercaw, K. I. Goldberg, *Organometallics* **2010**, *29*, 2176–2179. DOI: 10.1021/om100106e.

[S2] G. M. Sheldrick, *Acta Crystallogr. C* **2015**, *71*, 3–8. DOI: 10.1107/S2053229614024218.

[S3] D. Kratzert, J. J. Holstein, I. Krossing, *J. Appl. Crystallogr.* **2015**, *48*, 933–938. DOI: 10.1107/S1600576715005580.

[S4] M. J. Frisch, G. W. Trucks, H. B. Schlegel, G. E. Scuseria, M. A. Robb, J. R. Cheeseman, G. Scalmani, V. Barone, B. Mennucci, G. A. Petersson, H. Nakatsuji, M. Caricato, X. Li, H. P. Hratchian, A. F. Izmaylov, J. Bloino, G. Zheng, J. L. Sonnenberg, M. Hada, M. Ehara, K. Toyota, R. Fukuda, J. Hasegawa, M. Ishida, T. Nakajima, Y. Honda, O. Kitao, H. Nakai, T. Vreven, J. A. Montgomery Jr., J. E. Peralta, F. Ogliaro, M. Bearpark, J. J. Heyd, E. Brothers, K. N. Kudin, V. N. Staroverov, K. T., R. Kobayashi, J. Normand, K. Raghavachari, A. Rendell, J. C. Burant, S. S. Iyengar, J. Tomasi, M. Cossi, N. Rega, J. M. Millam, M. Klene, J. E. Knox, J. B. Cross, V. Bakken, C. Adamo, J. Jaramillo, R. Gomperts, R. E. Stratmann, O. Yazyev, A. J. Austin, R. Cammi, C. Pomelli, J. W. Ochterski, R. L. Martin, K. Morokuma, V. G. Zakrzewski, G. A. Voth, P. Salvador, J. J. Dannenberg, S. Dapprich, A. D. Daniels, Ö. Farkas, J. B. Foresman, J. V. Ortiz, J. Cioslowski, D. J. Fox, *Gaussian 09, Revision D.01*, Gaussian, Inc., Wallingford CT, **2016**.

[S5] S. H. Vosko, L. Wilk, M. Nusair, *Can. J. Phys.* **1980**, *58*, 1200–1211. DOI: 10.1139/p80-159.

[S6] C. Lee, W. Yang, R. G. Parr, *Phys Rev B Condens Matter* **1988**, *37*, 785–789. DOI: 10.1103/physrevb.37.785.

[S7] A. D. Becke, *J. Chem. Phys.* **1993**, *98*, 5648–5652. DOI: 10.1063/1.464913.

[S8] P. J. Stephens, F. J. Devlin, C. F. Chabalowski, M. J. Frisch, *J. Phys. Chem.* **1994**, *98*, 11623–11627. DOI: 10.1021/j100096a001.

[S9] J. J. Stewart, *J. Mol. Model.* **2007**, *13*, 1173-1213. 10.1007/s00894-007-0233-4.

[S10] T. Yanai, D. P. Tew, N. C. Handy, *Chem. Phys. Lett.* **2004**, *393*, 51–57. DOI: 10.1016/j.cplett.2004.06.011.

[S11] P. v. R. Schleyer, C. Maerker, A. Dransfeld, H. Jiao, N. J. R. van Eikema Hommes, *J. Am. Chem. Soc.* **1996**, *118*, 6317–6318. DOI: 10.1021/ja960582d.

[S12] K. Wolinski, J. F. Hinton, P. Pulay, *J. Am. Chem. Soc.* **1990**, *112*, 8251–8260. DOI: 10.1021/ja00179a005.

[S13] R. McWeeny, *Phys. Rev.* **1962**, *126*, 1028–1034. DOI: 10.1103/PhysRev.126.1028.

[S14] F. London, *J. Phys. Radium* **1937**, *8*, 397–409. DOI: 10.1051/jphysrad:01937008010039700.

[S15] R. Ditchfield, *Mol. Phys.* **1974**, *27*, 789–807. DOI: 10.1080/00268977400100711.

[S16] J. R. Cheeseman, G. W. Trucks, T. A. Keith, M. J. Frisch, *J. Chem. Phys.* **1996**, *104*, 5497–5509. DOI: 10.1063/1.471789.

[S17] R. Herges, D. Geuenich, *J. Phys. Chem. A* **2001**, *105*, 3214–3220. DOI: 10.1021/jp0034426.

[S18] D. Geuenich, K. Hess, F. Köhler, R. Herges, *Chem. Rev.* **2005**, *105*, 3758–3772. DOI: 10.1021/cr0300901.

[S19] T. Lu, F. Chen, *J. Comput. Chem.* **2012**, *33*, 580–592. DOI: 10.1002/jcc.22885.

[S20] G. Zhang, F. Rominger, U. Zschieschang, H. Klauk, M. Mastalerz, *Chem. Eur. J.* **2016**, *22*, 14840–14845. DOI: 10.1002/chem.201603336.

[S21] P. J. C. R. J. Bailey, and H. Shechte, *J. Am. Chem. Soc.* **1983**, *105*, 6096–6103. DOI: 10.1021/ja00357a021.

[S22] L. Shan, Z. Liang, X. Xu, Q. Tang, Q. Miao, *Chem. Sci.* **2013**, *4*, 3294–3297. DOI: 10.1039/c3sc51158h.

[S23] J. A. Gaunt, *Mathematical Proceedings of the Cambridge Philosophical Society* **2008**, *24*, 328–342. DOI: 10.1017/s0305004100015851.

[S24] D. R. Hartree, *Mathematical Proceedings of the Cambridge Philosophical Society* **2008**, *24*, 111–132. DOI: 10.1017/s0305004100011920.

[S25] J. C. Slater, *Phys. Rev.* **1928**, *32*, 339–348. DOI: 10.1103/PhysRev.32.339.

[S26] E. H. L. B. Simon, *Communications in Mathematical Physics* **1977**, *53*, 185–194 DOI: 10.1007/BF01609845.

[S27] V. Fock, *Zeitschrift für Physik* **1930**, *62*, 795–805. DOI: 10.1007/BF01330439.

[S28] C. C. J. Roothaan, *Reviews of Modern Physics* **1951**, *23*, 69–89. DOI: 10.1103/RevModPhys.23.69.

[S29] D. Feller, *J. Comput. Chem.* **1996**, *17*, 1571–1586.

[S30] B. T. D. Karen L. Schuchardt, Todd Elsethagen, Lisong Sun, Vidhya Gurumoorthi, Jared Chase, Jun Li, and Theresa L. Windus, *J. Chem. Inf. Model.* **2007**, *47*, 1045–1052. DOI: 10.1021/ci600510j.
